# Impaired glucose tolerance and cardiovascular risk factors in relation to infertility: a Mendelian randomization analysis in the Norwegian Mother, Father, and Child Cohort Study

**DOI:** 10.1093/humrep/dead234

**Published:** 2023-11-08

**Authors:** Álvaro Hernáez, Yunsung Lee, Christian M Page, Karoline H Skåra, Siri E Håberg, Per Magnus, Pål R Njølstad, Ole A Andreassen, Elizabeth C Corfield, Alexandra Havdahl, Abigail Fraser, Stephen Burgess, Deborah A Lawlor, Maria C Magnus

**Affiliations:** Centre for Fertility and Health, Norwegian Institute of Public Health, Oslo, Norway; Blanquerna School of Health Sciences, Universitat Ramon Llull, Barcelona, Spain; Centre for Fertility and Health, Norwegian Institute of Public Health, Oslo, Norway; Centre for Fertility and Health, Norwegian Institute of Public Health, Oslo, Norway; Division for Mental and Physical Health, Department of Physical Health and Ageing, Norwegian Institute of Public Health, Oslo, Norway; Centre for Fertility and Health, Norwegian Institute of Public Health, Oslo, Norway; Department of Community Medicine and Global Health, Institute of Health and Society, University of Oslo, Oslo, Norway; Centre for Fertility and Health, Norwegian Institute of Public Health, Oslo, Norway; Centre for Fertility and Health, Norwegian Institute of Public Health, Oslo, Norway; Department of Clinical Science, Mohn Center for Diabetes Precision Medicine, University of Bergen, Bergen, Norway; Children and Youth Clinic, Haukeland University Hospital, Bergen, Norway; Division of Mental Health and Addiction, Norwegian Centre for Mental Disorders Research, NORMENT, Oslo University Hospital, Oslo, Norway; Institute of Clinical Medicine, University of Oslo, Oslo, Norway; Department of Mental Disorders, Norwegian Institute of Public Health, Oslo, Norway; Nic Waals Institute, Lovisenberg Diakonale Hospital, Oslo, Norway; Department of Mental Disorders, Norwegian Institute of Public Health, Oslo, Norway; Nic Waals Institute, Lovisenberg Diakonale Hospital, Oslo, Norway; PROMENTA Research Center, Department of Psychology, University of Oslo, Oslo, Norway; Population Health Sciences, Bristol Medical School, University of Bristol, Bristol, UK; MRC Integrative Epidemiology Unit, University of Bristol, Bristol, UK; Population Health Sciences, Bristol Medical School, University of Bristol, Bristol, UK; MRC Integrative Epidemiology Unit, University of Bristol, Bristol, UK; MRC Biostatistics Unit, University of Cambridge, Cambridge, UK; Cardiovascular Epidemiology Unit, Department of Public Health and Primary Care, University of Cambridge, Cambridge, UK; Population Health Sciences, Bristol Medical School, University of Bristol, Bristol, UK; MRC Integrative Epidemiology Unit, University of Bristol, Bristol, UK; NIHR Bristol Biomedical Research Centre, Bristol, UK; Centre for Fertility and Health, Norwegian Institute of Public Health, Oslo, Norway; Population Health Sciences, Bristol Medical School, University of Bristol, Bristol, UK; MRC Integrative Epidemiology Unit, University of Bristol, Bristol, UK

**Keywords:** fasting insulin, glucose metabolism, blood pressure, lipid profile, infertility, Mendelian randomization, MoBa

## Abstract

**STUDY QUESTION:**

Are impaired glucose tolerance (as measured by fasting glucose, glycated hemoglobin, and fasting insulin) and cardiovascular disease risk (as measured by low-density lipoprotein cholesterol, high-density lipoprotein cholesterol, triglycerides, systolic blood pressure, and diastolic blood pressure) causally related to infertility?

**SUMMARY ANSWER:**

Genetic instruments suggest that higher fasting insulin may increase infertility in women.

**WHAT IS KNOWN ALREADY:**

Observational evidence suggests a shared etiology between impaired glucose tolerance, cardiovascular risk, and fertility problems.

**STUDY DESIGN, SIZE, DURATION:**

This study included two-sample Mendelian randomization (MR) analyses, in which we used genome-wide association summary data that were publicly available for the biomarkers of impaired glucose tolerance and cardiovascular disease, and sex-specific genome-wide association studies (GWASs) of infertility conducted in the Norwegian Mother, Father, and Child Cohort Study.

**PARTICIPANTS/MATERIALS, SETTING, METHODS:**

There were 68 882 women (average age 30, involved in 81 682 pregnancies) and 47 474 of their male partners (average age 33, 55 744 pregnancies) who had available genotype data and who provided self-reported information on time-to-pregnancy and use of ARTs. Of couples, 12% were infertile (having tried to conceive for ≥12 months or used ARTs to conceive). We applied the inverse variance weighted method with random effects to pool data across variants and a series of sensitivity analyses to explore genetic instrument validity. (We checked the robustness of genetic instruments and the lack of unbalanced horizontal pleiotropy, and we used methods that are robust to population stratification.) Findings were corrected for multiple comparisons by the Bonferroni method (eight exposures: *P*-value < 0.00625).

**MAIN RESULTS AND THE ROLE OF CHANCE:**

In women, increases in genetically determined fasting insulin levels were associated with greater odds of infertility (+1 log(pmol/l): odds ratio 1.60, 95% CI 1.17 to 2.18, *P*-value = 0.003). The results were robust in the sensitivity analyses exploring the validity of MR assumptions and the role of pleiotropy of other cardiometabolic risk factors. There was also evidence of higher glucose and glycated hemoglobin causing infertility in women, but the findings were imprecise and did not pass our *P*-value threshold for multiple testing. The results for lipids and blood pressure were close to the null, suggesting that these did not cause infertility.

**LIMITATIONS, REASONS FOR CAUTION:**

We did not know if underlying causes of infertility were in the woman, man, or both. Our analyses only involved couples who had conceived. We did not have data on circulating levels of cardiometabolic risk factors, and we opted to conduct an MR analysis using GWAS summary statistics. No sex-specific genetic instruments on cardiometabolic risk factors were available. Our results may be affected by selection and misclassification bias. Finally, the characteristics of our study sample limit the generalizability of our results to populations of non-European ancestry.

**WIDER IMPLICATIONS OF THE FINDINGS:**

Treatments for lower fasting insulin levels may reduce the risk of infertility in women.

**STUDY FUNDING/COMPETING INTEREST(S):**

The MoBa Cohort Study is supported by the Norwegian Ministry of Health and Care Services and the Norwegian Ministry of Education and Research. This work was supported by the European Research Council [grant numbers 947684, 101071773, 293574, 101021566], the Research Council of Norway [grant numbers 262700, 320656, 274611], the South-Eastern Norway Regional Health Authority [grant numbers 2020022, 2021045], and the British Heart Foundation [grant numbers CH/F/20/90003, AA/18/1/34219]. Open Access funding was provided by the Norwegian Institute of Public Health. The funders had no role in the study design; the collection, analysis, and interpretation of data; the writing of the report; or the decision to submit the article for publication. D.A.L. has received research support from National and International government and charitable bodies, Roche Diagnostics and Medtronic for research unrelated to the current work. O.A.A. has been a consultant to HealthLytix. The rest of the authors declare that no competing interests exist.

**TRIAL REGISTRATION NUMBER:**

N/A.

## Introduction

Impaired glucose tolerance and other cardiovascular risk factors are related to fertility problems in women (Mulder *et al.*, 2020; Vatier *et al.*, 2022) and men (Alves *et al.*, 2013; Keihani *et al.*, 2019). However, these associations could be explained by residual confounding, as other exposures related to these risk factors may be causing fertility problems (Davey Smith and Hemani, 2014). Mendelian randomization (MR), a method based on genetic instrumental variables, can be used to obtain potential causal effects that are unaffected by this source of confounding (Davey Smith and Hemani, 2014). Using this methodology, we have previously described that high and low body weight increase the risk of infertility in both sexes (Hernaez *et al.*, 2021), while the previously reported association between smoking and infertility may not be causal (Hernáez *et al.*, 2022). However, the role of other cardiometabolic risk factors on infertility according to MR remains to be elucidated.

The aim of this study was to investigate whether biomarkers of impaired glucose tolerance (fasting glucose, glycated hemoglobin, and fasting insulin) and cardiovascular disease risk factors (low-density lipoprotein cholesterol (LDL-C), high-density lipoprotein cholesterol (HDL-C), triglycerides, systolic blood pressure (SBP), and diastolic blood pressure (DBP)) influence the risk of infertility using an MR-based approach.

## Materials and methods

### Population

Our study involved data from the Norwegian Mother, Father, and Child Cohort Study (MoBa) (Magnus *et al.*, 2016). MoBa is a population-based pregnancy cohort study administered by the Norwegian Institute of Public Health. Pregnant women and their partners were recruited at ∼18 gestational weeks across Norway between 1999 and 2008. The cohort is composed of 114 500 children, 95 200 mothers, and 75 200 fathers. Blood samples were obtained from both parents during pregnancy (Paltiel *et al.*, 2014). Details of the MoBa genotyping data and quality control have been previously described (Corfield *et al.*, 2022). This work is based on a subsample of parents involved in singleton pregnancies with available genotype and infertility information (68 882 women and 47 474 men) from version 12 of the quality-assured data files released on 11 May 2022. Our study is presented following the STrengthening the Reporting of OBservational studies in Epidemiology (STROBE) guidelines for reporting MR and cohort studies.

### Genetic variants and instrumental variables

We extracted independent single-nucleotide polymorphisms (SNPs) as genetic instruments from the most recent genome-wide association studies (GWASs) on glucose metabolism (Chen *et al.*, 2021), lipid profile (Graham *et al.*, 2021), and blood pressure (Evangelou *et al.*, 2018). Independent SNPs were not in linkage disequilibrium (pairwise *r*^2^ < 0.01) and were associated with their phenotypes according to genome-wide significance thresholds. MoBa was not part of any of these GWASs. We used SNPs individually as genetic instruments (Burgess *et al.*, 2019). SNPs that were used in the analyses and the magnitude of their association with the exposure and the outcome are described in Supplementary Tables S1, S2, S3, S4, S5, S6, S7, and S8. The number of SNPs used in each approach is described in Supplementary Table S9.

### Infertility

Women were asked if the pregnancy was planned and how many months it took them to conceive (options: ‘<1 month’, ‘1–2 months’, or ‘≥3 months’) (Magnus *et al.*, 2016). If they answered ‘≥3 months’, they were further asked to specify the exact number of months. An individual was defined as infertile whether they reported trying to conceive for ≥12 months or having used ARTs in any of their MoBa pregnancies (Hernáez *et al.*, 2022). Information on use of ARTs was available through linkage to the birth registry. The reference group comprised participants who conceived spontaneously within 12 months. Couples with unplanned pregnancies were excluded.

### Other variables

We obtained information from both parents on their age at delivery (continuous), years of education (continuous), BMI (continuous), tobacco use (having ever smoked or not), and number of previous deliveries (continuous). We report the maximum values for all continuous variables for those parents who participated in more than one pregnancy in MoBa.

### Ethical approval

The MoBa study follows the Declaration of Helsinki for Medical Research involving human subjects. The establishment of MoBa and its initial data collection were based on a license from the Norwegian Data Protection Agency and approved by Regional Committees for Medical and Health Research Ethics. The MoBa cohort is now based on the regulations in the Norwegian Health Registry Act. Participants provided written informed consent before joining the cohort. This project was approved by the Regional Committee for Medical and Health Research Ethics of South/East Norway (reference: 2017/1362).

### Statistical analyses

We described normally distributed continuous variables by means and SDs, non-normally distributed continuous variables by medians and 1st–3rd quartiles, and categorical variables by proportions. We assessed differences in baseline characteristics between parents with and without infertility and between MoBa participants included and not included in this study (due to a lack of genotype data), by unpaired *t*-tests (normally distributed variables), Mann–Whitney *U*-tests (non-normally distributed variables), and chi-squared tests (categorical variables).

We first performed sex-specific GWASs of infertility in MoBa using Scalable and Accurate Implementation of GEneralized mixed models (accounting for kinship and adjusted for the first 20 genetic principal components, genotype batch, and age at reported infertility). We then searched for the SNPs linked to the cardiometabolic risk factors in the summary statistics of the infertility GWASs. Palindromic SNPs with allele frequencies close to 0.5 were excluded. After data harmonization, we estimated the association between the risk factor of interest and infertility using a random effects inverse variance weighted method (Burgess *et al.*, 2019). We corrected our findings for multiple comparisons (eight exposures) by the Bonferroni method (*P*-value threshold < 0.00625).

There are three key assumptions in MR: (i) genetic instruments are robustly related to the exposure, (ii) there is no confounding of the genetic instrument–outcome associations, and (iii) genetic instruments are exclusively linked to the outcome through the exposure of interest. If violated, MR conclusions may be invalid (Burgess *et al.*, 2019). In relation to the first assumption, we estimated the robustness of our genetic instruments as the mean *F*-statistic (average beta^2^/standard error^2^ across all SNPs; *F*-statistics < 10 are indicative of a weak genetic instrument (Burgess *et al.*, 2019)). Regarding the second assumption, we reduced the potential confounding of the genetic instrument–outcome association due to population stratification by: (i) selecting our genetic instruments from GWASs performed in European population; (ii) adjusting the one-sample MR analyses for the first 20 ancestry-informative genetic principal components; and (iii) conducting two-sample MR analyses using GWASs adjusted for genetic principal components. Finally, the third MR assumption is violated by genetic instruments that are related to other risk factors for the outcome different to the exposure of interest (horizontal pleiotropy) (Davey Smith and Hemani, 2014). We checked the presence of horizontal pleiotropy by several procedures based on sensitivity two-sample MR methods (MR-Egger, weighted median, and weighted mode) (Hemani *et al.*, 2018). It can be identified if: (i) a non-zero intercept is present in the MR-Egger method; (ii) there is no concordance among MR estimates in inverse variance weighted and the alternative methods; and (iii) between SNP heterogeneity is found according to the Cochran’s *Q* and the Rücker’s *Q’* (Hemani *et al.*, 2018). Several associations were affected by horizontal pleiotropy, possibly due to the high inter-relation among cardiometabolic risk factors. Thus, we also used the Steiger filtering, which allows one to determine which SNPs explain more variation in our exposure of interest than in the rest of variables related to it (which are less susceptible of biasing findings due to horizontal pleiotropy) (Hemani *et al.*, 2018). For example, 71 of the SNPs for fasting glucose explained more variability in fasting glucose than in the other risk factors considered (glycated hemoglobin, fasting insulin, LDL-C, HDL-C, triglycerides, SBP, DBP, BMI, and ever smoking) and were retained after the filtering. As sensitivity analyses, we ran two-sample MR using exclusively the SNPs that passed the filtering process.

Analyses were conducted in R Software v. 4.1.2. Code for data management and analysis is available at https://github.com/alvarohernaez/MR_cardiometabolic_infertility_MoBa.

## Results

### Study population

**Figure 1.**
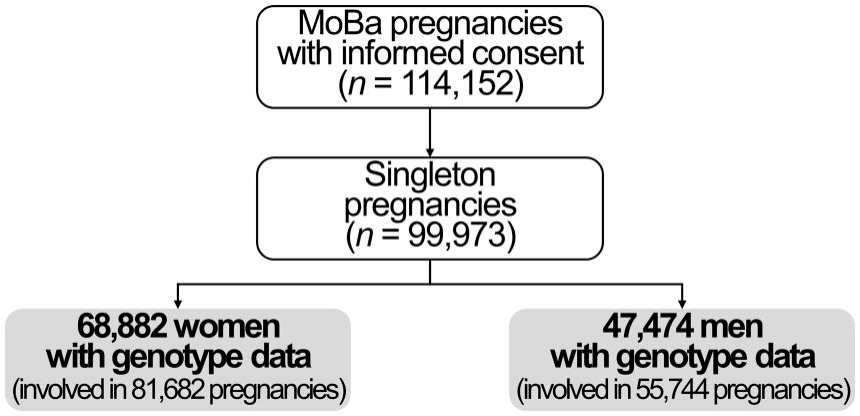
Study flow chart.

Our study population consisted of 68 882 women (a total of 81 682 pregnancies) and 47 474 men (55 744 pregnancies) (Fig. 1). Infertility was reported in 12% of the couples. Participants with infertility were older, had lower educational attainment, greater mean BMI values, were more likely to have smoked, and were more likely to have a first time pregnancy (Table 1). MoBa participants with genotype information had higher educational attainment, were less likely to have smoked, and were more likely to report infertility, compared to those without genotype information (Supplementary Table S10).

**Table 1.** Population characteristics.

	Women			Men		
	All	No infertility reported	Infertility reported	All	No infertility reported	Infertility reported
	(n = 68 882)	(n = 60 532)	(n = 8350)	(n = 47 474)	(n = 41 624)	(n = 5850)
Age at delivery, years (mean ± SD)	30.5 ± 4.61	30.3 ± 4.58	32.1 ± 4.47	32.9 ± 5.27	32.6 ± 5.21	34.6 ± 5.38
Education years (mean ± SD)	17.1 ± 3.35	17.1 ± 3.34	16.9 ± 3.39	16.4 ± 3.55	16.4 ± 3.54	16.1 ± 3.57
BMI, kg/m^2^ (median, 1st–3rd quartile)	23.2 (21.2–26.1)	23.1 (21.1–26.0)	23.9 (21.5–27.4)	25.5 (23.7–27.8)	25.5 (23.7–27.7)	25.9 (24.1–28.3)
Ever smokers (*n*, %)	35 632 (52.3%)	31 247 (52.3%)	4385 (53.0%)	24 299 (51.2%)	21 232 (51.0%)	3067 (52.4%)
Trying for a first pregnancy (*n*, %)	27 030 (39.3%)	23 290 (38.6%)	3740 (44.9%)	20 026 (42.3%)	17 212 (41.4%)	2814 (48.2%)

### Cardiometabolic risk factors and infertility

**Figure 2.**
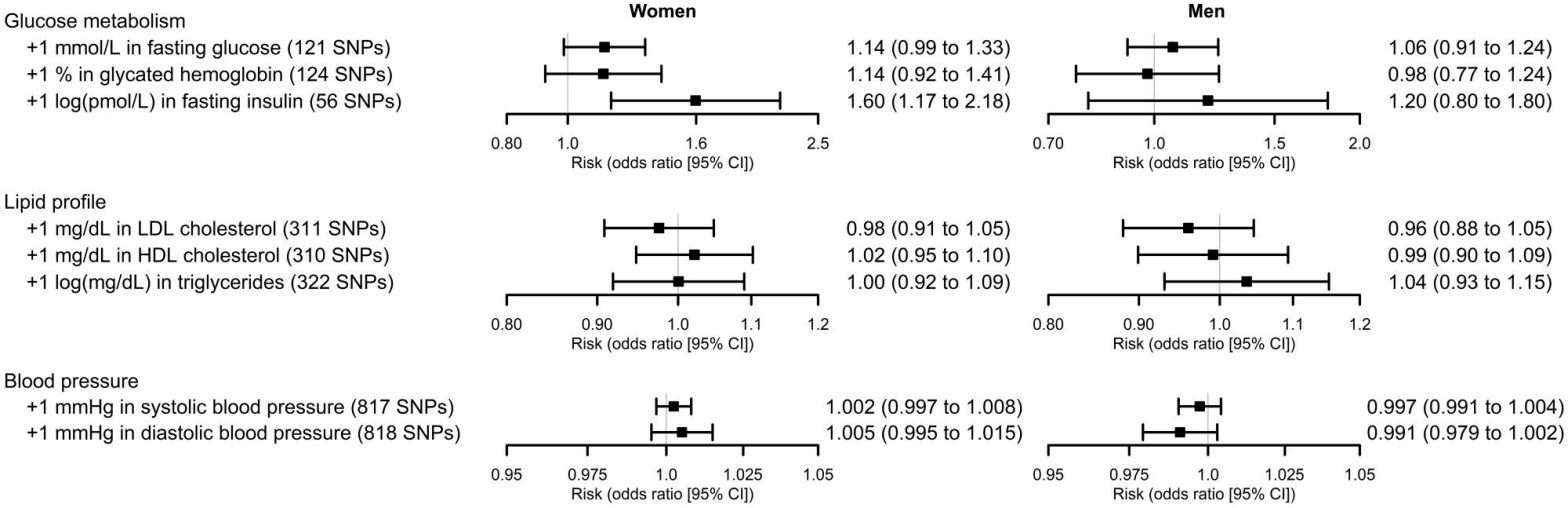
**Mendelian randomization (MR) analyses on the relationship of cardiometabolic risk factors with infertility in women and men.** The units differ for each exposure, so the results are not directly comparable in magnitude.

In women, increases in the genetically determined levels of fasting insulin were related to greater odds of infertility (+1 log(pmol/l): odds ratio 1.60, 95% CI 1.17 to 2.18, *P*-value = 0.003) (Fig. 2). We found little evidence that this result was affected by weak instrument bias (estimated *F*-statistic: 52.5) or unbalanced horizontal pleiotropy (MR-Egger intercept test *P*-value = 0.194; no evidence of between-SNP heterogeneity (Cochran’s *Q* = 55.8, *P*-value = 0.445; Rücker’s *Q’* = 54.1, *P*-value = 0.472); consistency in directionality of estimates in MR sensitivity methods; and consistency between main analyses and those only using the genetic variants that were unrelated to other risk factors according to the Steiger filtering) (Supplementary Table S11). Associations of higher maternal fasting glucose and glycated hemoglobin in women with greater infertility odds were suggested. However, these relationships were imprecise and did not pass our multiple testing *P*-value threshold. Lipid and blood pressure associations with infertility were close to the null (Fig. 2, Supplementary Table S11).

## Discussion

In this MR analysis, genetic instruments suggested that higher concentrations of fasting insulin increase the risk of infertility in women. An association between higher fasting glucose and glycated hemoglobin in women and higher risk of infertility was also suggested but analyses in larger numbers of participants are needed to confirm this.

Hyperinsulinemia, a marker of insulin resistance, and glucose intolerance are commonly present in women with polycystic ovary syndrome, which are in turn at higher risk of being infertile (Zhang *et al.*, 2019). Hyperinsulinemia is also linked to follicular dysplasia and impaired synthesis of sexual hormones in the follicle (less progesterone, lower availability of FSH, and more androgens) (Foong *et al.*, 2006; Xu *et al.*, 2019). Our findings support hyperinsulinemia as a cause of infertility in women. Our other findings, such as the suggested associations between higher genetically determined levels of fasting glucose and glycosylated hemoglobin and infertility, are also in agreement with this observation. The lack of robust statistical evidence for an effect of lipids and blood pressure on infertility suggests that previous observational results (Keihani *et al.*, 2019; Mulder *et al.*, 2020; Vatier *et al.*, 2022) may have been influenced by residual confounding.

Our work has several strengths. MR studies exploring the relationship between cardiometabolic risk factors and a clinical definition of fertility have been lacking. Our findings appear robust because our population was relatively homogeneous, and we thoroughly evaluated the compliance with MR assumptions. However, our study has some limitations.

First, infertility is a couple-dependent measure. Thus, we could not establish whether underlying causes of infertility were in the woman, man, or both. In addition, we lacked information on potential causes of infertility (polycystic ovary syndrome, blocked tubes, anovulation due to clomiphene use, etc.). We were also unable to suggest a particular cause in most of the infertility cases. In our data, ICSI, which is more often linked to male factor infertility, was reported in 7.7% of cases of male infertility (92.3% of male infertility cases remained unexplained), and IVF, which is less often related to male factor infertility, was reported in 10.6% of cases of female infertility (89.4% of female infertility cases were unexplained).

Second, MoBa only recruited couples who had conceived. Cardiometabolic risk factors may be associated with more severe forms of infertility, such as sterility (which is absolute inability to conceive). Therefore, our findings should be validated in studies also involving sterile couples.

Third, we did not have data on circulating levels of cardiometabolic risk factors in MoBa and there was no GWAS of infertility with available summary data across the genome. We opted to undertake a GWAS of infertility in MoBa (with no independent replication) and a two-sample MR using summary results from publicly available GWAS of the exposures and those from our MoBa GWAS. In addition, our outcome GWAS was relatively small. Thus, our findings should be replicated using summary statistics of a larger GWAS on infertility. It would also be advisable to replicate the results in a large independent sample, with information on circulating levels of cardiometabolic risk factors and infertility, in which a one-sample MR analysis could be undertaken. Besides, due to the limited size of our GWAS on infertility, we decided to highlight some results that were clinically relevant but did not pass multiple testing (i.e. the associations between the genetically predicted levels of fasting glucose and glycated hemoglobin in women and greater odds of infertility). We decided to highlight these findings because the other indicator of impaired glucose tolerance (fasting insulin) was positively associated with the odds of infertility in our data, and this therefore suggests a plausible relationship between impaired glucose tolerance as a whole and infertility.

Fourth, no sex-specific genetic instruments were available in the largest GWASs on cardiometabolic risk factors, and we had to assume that there were no sex differences in the genetic instruments for exposures. If this is not the case, our results could be biased.

Fifth, MoBa participants with genotype information had higher educational attainment, were less likely to have ever smoked, and were more likely to report being infertile. These factors may introduce selection bias into our findings and, as a result, decrease our internal and external validity. However, previous studies comparing MoBa participants with the general Norwegian population have shown that selection bias had a minimal impact on pregnancy outcomes (Nilsen *et al.*, 2009).

Sixth, we might not have been able to detect all cases of infertility in our population (e.g. an individual who may have taken ≥12 months to conceive in pregnancies outside of the MoBa study), which could have affected our ability to find associations in our study and introduced misclassification bias.

Finally, the characteristics of our study sample limit the generalizability of our results to other populations, particularly populations of genetic ancestries other than European.

In conclusion, genetic instruments suggest a role of hyperinsulinemia and possibly glucose intolerance on infertility in women. If further research in larger samples and with sex-specific exposure instruments supports our findings, preventing hyperinsulinemia and impaired glucose tolerance in women of childbearing age (through strategies such as low-glycemic index diets, regular physical activity, weight management, proper sleep patterns, or glucose-lowering medication if needed) (Davies *et al.*, 2022) may be a means of reducing fertility problems in this population.

## Supplementary Material

dead234_Supplementary_Table_S1

dead234_Supplementary_Table_S2

dead234_Supplementary_Table_S3

dead234_Supplementary_Table_S4

dead234_Supplementary_Table_S5

dead234_Supplementary_Table_S6

dead234_Supplementary_Table_S7

dead234_Supplementary_Table_S8

dead234_Supplementary_Table_S9

dead234_Supplementary_Table_S10

dead234_Supplementary_Table_S11

## Data Availability

The consent given by the participants does not allow for storage of individual data in repositories or journals. Researchers who want access to data sets for replication should apply through datatilgang@fhi.no. This process requires approval from the Regional Committee for Medical and Health Research Ethics in Norway and an agreement with MoBa. GWAS summary statistics on fasting glucose, glycated hemoglobin, and fasting insulin have been contributed by the Meta-Analyses of Glucose and Insulin-related traits Consortium investigators and have been downloaded from https://magicinvestigators.org/downloads/ (menu: ‘Trans-ancestry and single-ancestry meta-analysis summary statistics of four glycemic traits’). GWAS summary statistics on lipid profile traits have been contributed by the Global Lipids Genetics Consortium investigators and have been downloaded from http://csg.sph.umich.edu/willer/public/glgc-lipids2021/ (menu: ‘Ancestry-specific GWAS summary statistics for HDL-C, LDL-C, non-HDL-C, TC and TG’). Finally, GWAS summary statistics on blood pressure have been contributed by Evangelou *et al.* (2018) and have been downloaded from the Supplementary Tables of the article (https://www.nature.com/articles/s41588-018-0205-x#MOESM3).
