# Supplementary material for: Impaired glucose tolerance and cardiovascular risk factors in relation to infertility: a Mendelian randomization analysis in the Norwegian Mother, Father, and Child Cohort Study
Source: Hum Reprod. 2023 Nov 8;39(2):436–41. doi: 10.1093/humrep/dead234 (PMC10833082; doi:10.1093/humrep/dead234)
Supplement: dead234_Supplementary_Table_S1 [file dead234_supplementary_table_s1.docx]

**Supplementary Table S1.** SNPs included in fasting glucose-related analyses.

| **RSID** | **Chrom.** | **Position** | **Used in MR** | **Used in MR**  **+ Steiger filt.** | **Effect**  **allele** | **Other**  **allele** | **Effect**  **allele**  **freq.** | **Exposure:**  **beta** | **Exposure:**  **SE** | **Outcome**  **(women):**  **beta** | **Outcome**  **(women):**  **SE** | **Outcome**  **(men):**  **beta** | **Outcome**  **(men):**  **SE** |
| --- | --- | --- | --- | --- | --- | --- | --- | --- | --- | --- | --- | --- | --- |
| rs6662924 | 1 | 100894419 | Yes | Yes | A | C | 0.19 | 0.014 | 0.002 | 0.002 | 0.021 | 0.025 | 0.025 |
| rs78132593 | 1 | 150868102 | Yes | No | A | C | 0.21 | -0.015 | 0.002 | 0.005 | 0.021 | -0.016 | 0.025 |
| rs267738 | 1 | 150940625 | Yes | No | T | G | 0.79 | 0.013 | 0.002 | -0.001 | 0.021 | 0.015 | 0.024 |
| rs2075423 | 1 | 214154719 | Yes | Yes | T | G | 0.37 | -0.016 | 0.002 | -0.076 | 0.018 | -0.013 | 0.021 |
| rs340874 | 1 | 214159256 | Yes | Yes | T | C | 0.47 | -0.015 | 0.002 | -0.053 | 0.017 | -0.015 | 0.02 |
| rs348330 | 1 | 229672955 | No | No | - | - | - | - | - | - | - | - | - |
| rs877273 | 2 | 27140022 | Yes | Yes | T | C | 0.38 | 0.013 | 0.002 | -0.026 | 0.017 | -0.022 | 0.021 |
| rs1371614 | 2 | 27152874 | Yes | Yes | T | C | 0.25 | 0.016 | 0.002 | -0.034 | 0.019 | -0.005 | 0.022 |
| rs1260326 | 2 | 27730940 | Yes | Yes | T | C | 0.39 | -0.028 | 0.002 | -0.001 | 0.018 | -0.003 | 0.021 |
| rs780093 | 2 | 27742603 | Yes | Yes | T | C | 0.38 | -0.028 | 0.002 | 0.001 | 0.018 | -0.011 | 0.022 |
| rs115640879 | 2 | 28268742 | Yes | No | T | C | 0.08 | -0.024 | 0.004 | 0.003 | 0.043 | 0.009 | 0.052 |
| rs183381538 | 2 | 43775309 | No | No | - | - | - | - | - | - | - | - | - |
| rs189548 | 2 | 54941112 | Yes | Yes | A | G | 0.73 | -0.012 | 0.002 | 0.013 | 0.019 | -0.006 | 0.023 |
| rs78996387 | 2 | 169238040 | Yes | No | T | C | 0.08 | -0.023 | 0.004 | 0.031 | 0.034 | 0.025 | 0.04 |
| rs72883292 | 2 | 169710158 | Yes | No | T | C | 0.02 | -0.009 | 0.007 | -0.035 | 0.06 | 0.006 | 0.071 |
| rs150171632 | 2 | 169748691 | No | No | - | - | - | - | - | - | - | - | - |
| rs111485380 | 2 | 169754123 | No | No | - | - | - | - | - | - | - | - | - |
| rs540524 | 2 | 169756930 | Yes | Yes | A | G | 0.62 | -0.011 | 0.002 | 0.025 | 0.017 | -0.035 | 0.021 |
| rs1402837 | 2 | 169757354 | Yes | Yes | T | C | 0.22 | 0.056 | 0.002 | 0.008 | 0.02 | -0.029 | 0.024 |
| rs573225 | 2 | 169757541 | Yes | Yes | A | G | 0.68 | 0.069 | 0.002 | -0.018 | 0.018 | 0.009 | 0.022 |
| rs560887 | 2 | 169763148 | Yes | Yes | T | C | 0.3 | -0.075 | 0.002 | 0.022 | 0.019 | -0.014 | 0.022 |
| rs492594 | 2 | 169764176 | No | No | - | - | - | - | - | - | - | - | - |
| rs145353824 | 2 | 169765277 | No | No | - | - | - | - | - | - | - | - | - |
| rs17539351 | 2 | 169766560 | Yes | Yes | T | C | 0.12 | -0.068 | 0.003 | 0.036 | 0.028 | 0.009 | 0.033 |
| rs13430620 | 2 | 169768891 | No | No | - | - | - | - | - | - | - | - | - |
| rs563694 | 2 | 169774071 | Yes | Yes | A | C | 0.65 | 0.066 | 0.002 | -0.015 | 0.018 | 0.01 | 0.021 |
| rs114764002 | 2 | 169776141 | No | No | - | - | - | - | - | - | - | - | - |
| rs191257736 | 2 | 169778471 | No | No | - | - | - | - | - | - | - | - | - |
| rs56100844 | 2 | 169786707 | Yes | No | T | G | 0.98 | 0.118 | 0.01 | -0.006 | 0.072 | -0.005 | 0.085 |
| rs3755158 | 2 | 169792188 | Yes | Yes | C | G | 0.89 | -0.04 | 0.003 | 0.027 | 0.027 | -0.008 | 0.032 |
| rs508741 | 2 | 169795288 | Yes | Yes | T | C | 0.64 | -0.013 | 0.002 | 0.012 | 0.018 | -0.036 | 0.021 |
| rs199666154 | 2 | 169800434 | No | No | - | - | - | - | - | - | - | - | - |
| rs114691375 | 2 | 169813318 | Yes | No | A | T | 0.03 | 0.045 | 0.006 | -0.032 | 0.056 | -0.004 | 0.065 |
| rs6731931 | 2 | 173593726 | Yes | No | T | C | 0.8 | -0.012 | 0.002 | 0.013 | 0.022 | 0.036 | 0.026 |
| rs11708067 | 3 | 123065778 | Yes | Yes | A | G | 0.77 | 0.028 | 0.002 | 0.037 | 0.02 | -0.012 | 0.024 |
| rs75098673 | 3 | 141094338 | Yes | No | T | C | 0.05 | -0.035 | 0.005 | -0.025 | 0.037 | 0.009 | 0.044 |
| rs16851397 | 3 | 141134818 | Yes | Yes | A | G | 0.95 | 0.033 | 0.004 | 0.023 | 0.037 | 0.004 | 0.044 |
| rs17437560 | 3 | 152180329 | Yes | Yes | T | C | 0.11 | -0.018 | 0.003 | 0.022 | 0.029 | 0.008 | 0.034 |
| rs1604038 | 3 | 170709193 | Yes | Yes | T | C | 0.29 | -0.02 | 0.002 | -0.007 | 0.018 | 0.027 | 0.022 |
| rs3215234 | 3 | 170724091 | No | No | - | - | - | - | - | - | - | - | - |
| rs7631557 | 3 | 185513646 | Yes | No | C | G | 0.3 | 0.011 | 0.002 | -0.013 | 0.019 | -0.025 | 0.022 |
| rs35188816 | 3 | 185526108 | No | No | - | - | - | - | - | - | - | - | - |
| rs6808574 | 3 | 187740523 | Yes | Yes | T | C | 0.39 | -0.013 | 0.002 | 0.006 | 0.017 | -0.02 | 0.021 |
| rs4862423 | 4 | 185726548 | Yes | Yes | T | C | 0.4 | 0.012 | 0.002 | 0.002 | 0.017 | 0.011 | 0.021 |
| rs157512 | 5 | 55809127 | Yes | No | T | C | 0.76 | 0.013 | 0.002 | 0.033 | 0.019 | 0.031 | 0.023 |
| rs7708285 | 5 | 76425867 | Yes | Yes | A | G | 0.73 | -0.013 | 0.002 | 0.01 | 0.019 | -0.037 | 0.023 |
| rs6878122 | 5 | 76427311 | Yes | No | A | G | 0.72 | -0.013 | 0.002 | 0.009 | 0.019 | -0.028 | 0.022 |
| rs1820176 | 5 | 95696585 | Yes | No | T | C | 0.71 | 0.025 | 0.002 | 0.014 | 0.019 | 0.008 | 0.022 |
| rs6876986 | 5 | 95703329 | Yes | No | C | G | 0.29 | -0.024 | 0.002 | -0.014 | 0.018 | -0.008 | 0.022 |
| rs7729395 | 5 | 102100576 | Yes | Yes | T | C | 0.05 | -0.023 | 0.005 | -0.019 | 0.039 | -0.002 | 0.046 |
| rs9379084 | 6 | 7231843 | Yes | Yes | A | G | 0.13 | -0.012 | 0.003 | -0.014 | 0.03 | 0.02 | 0.035 |
| rs3778321 | 6 | 7250270 | Yes | Yes | A | G | 0.19 | -0.019 | 0.002 | 0.032 | 0.021 | -0.018 | 0.025 |
| rs9348441 | 6 | 20680678 | Yes | No | A | T | 0.28 | 0.018 | 0.002 | 0.007 | 0.019 | -0.025 | 0.023 |
| rs10305457 | 6 | 39034095 | Yes | No | T | C | 0.09 | 0.024 | 0.003 | -0.027 | 0.03 | 0.004 | 0.036 |
| rs10305492 | 6 | 39046794 | Yes | Yes | A | G | 0.014 | -0.076 | 0.01 | 0.055 | 0.062 | 0.015 | 0.074 |
| rs12055786 | 6 | 153431125 | Yes | Yes | T | C | 0.42 | 0.012 | 0.002 | 0.015 | 0.017 | -0.002 | 0.02 |
| rs10281892 | 7 | 14919852 | Yes | No | A | G | 0.82 | -0.028 | 0.002 | -0.013 | 0.022 | 0.048 | 0.027 |
| rs2191346 | 7 | 15053878 | No | No | - | - | - | - | - | - | - | - | - |
| rs10487796 | 7 | 15063430 | No | No | - | - | - | - | - | - | - | - | - |
| rs10228796 | 7 | 15064190 | No | No | - | - | - | - | - | - | - | - | - |
| rs55841377 | 7 | 44145178 | Yes | No | C | G | 0.78 | 0.009 | 0.002 | 0.03 | 0.021 | -0.009 | 0.025 |
| rs2971671 | 7 | 44211337 | Yes | Yes | T | C | 0.77 | -0.043 | 0.002 | -0.041 | 0.021 | -0.019 | 0.025 |
| rs2971670 | 7 | 44226101 | Yes | No | T | C | 0.17 | 0.061 | 0.002 | 0.013 | 0.023 | 0.022 | 0.028 |
| rs1799884 | 7 | 44229068 | Yes | Yes | T | C | 0.17 | 0.062 | 0.002 | 0.013 | 0.023 | 0.023 | 0.028 |
| rs3757840 | 7 | 44231216 | Yes | Yes | T | G | 0.49 | 0.042 | 0.002 | 0.024 | 0.017 | 0.012 | 0.02 |
| rs6975024 | 7 | 44231886 | Yes | Yes | T | C | 0.84 | -0.062 | 0.002 | -0.011 | 0.023 | -0.024 | 0.028 |
| rs2908286 | 7 | 44234737 | Yes | No | T | C | 0.17 | 0.061 | 0.002 | 0.013 | 0.024 | 0.022 | 0.028 |
| rs138917529 | 7 | 44235694 | Yes | No | A | T | 0.98 | 0.06 | 0.007 | 0.018 | 0.067 | 0.118 | 0.079 |
| rs878521 | 7 | 44255643 | Yes | No | A | G | 0.24 | 0.055 | 0.002 | 0.016 | 0.02 | 0.014 | 0.024 |
| rs2108349 | 7 | 50786663 | Yes | Yes | A | G | 0.66 | -0.016 | 0.002 | -0.021 | 0.018 | -0.023 | 0.021 |
| rs58925536 | 7 | 75654574 | Yes | No | T | C | 0.03 | 0.031 | 0.005 | -0.057 | 0.049 | 0.034 | 0.058 |
| rs67070387 | 7 | 75836023 | Yes | No | T | C | 0.03 | 0.031 | 0.006 | -0.068 | 0.049 | 0.042 | 0.058 |
| rs13242882 | 7 | 89800053 | No | No | - | - | - | - | - | - | - | - | - |
| rs194520 | 7 | 89854446 | Yes | Yes | T | G | 0.45 | -0.009 | 0.002 | -0.02 | 0.017 | -0.025 | 0.02 |
| rs7012637 | 8 | 9173209 | Yes | No | A | G | 0.47 | -0.018 | 0.002 | -0.007 | 0.017 | 0.014 | 0.02 |
| rs9987289 | 8 | 9183358 | Yes | No | A | G | 0.1 | 0.028 | 0.003 | 0.005 | 0.027 | -0.043 | 0.033 |
| rs4841132 | 8 | 9183596 | Yes | No | A | G | 0.1 | 0.028 | 0.003 | 0.005 | 0.027 | -0.044 | 0.033 |
| rs12541643 | 8 | 81076874 | Yes | No | T | C | 0.48 | 0.012 | 0.002 | 0.004 | 0.017 | 0.023 | 0.02 |
| rs896854 | 8 | 95960511 | Yes | Yes | T | C | 0.5 | 0.01 | 0.002 | -0.004 | 0.017 | 0.016 | 0.02 |
| rs13266634 | 8 | 118184783 | Yes | Yes | T | C | 0.32 | -0.029 | 0.002 | -0.016 | 0.018 | -0.018 | 0.022 |
| rs9650069 | 8 | 118204020 | Yes | No | T | C | 0.32 | -0.029 | 0.002 | -0.013 | 0.018 | -0.021 | 0.022 |
| rs1574285 | 9 | 4283137 | Yes | Yes | T | G | 0.59 | -0.019 | 0.002 | -0.004 | 0.017 | 0.033 | 0.02 |
| rs57884925 | 9 | 4285119 | No | No | - | - | - | - | - | - | - | - | - |
| rs10974438 | 9 | 4291928 | Yes | Yes | A | C | 0.64 | -0.02 | 0.002 | 0.011 | 0.017 | 0.011 | 0.021 |
| rs10217762 | 9 | 22133645 | Yes | No | T | C | 0.56 | -0.003 | 0.002 | -0.027 | 0.017 | -0.012 | 0.02 |
| rs10811660 | 9 | 22134068 | Yes | No | A | G | 0.18 | -0.022 | 0.002 | 0.034 | 0.023 | 0.034 | 0.027 |
| rs16913693 | 9 | 111680359 | Yes | Yes | T | G | 0.97 | 0.039 | 0.005 | -0.084 | 0.039 | -0.059 | 0.047 |
| rs507666 | 9 | 136149399 | Yes | No | A | G | 0.2 | 0.016 | 0.002 | 0.022 | 0.02 | 0.01 | 0.024 |
| rs3829109 | 9 | 139256766 | Yes | Yes | A | G | 0.3 | -0.016 | 0.002 | -0.006 | 0.018 | -0.007 | 0.022 |
| rs10781511 | 9 | 139280766 | Yes | No | A | G | 0.27 | -0.011 | 0.002 | -0.002 | 0.019 | 0 | 0.023 |
| rs2839671 | 10 | 26505822 | Yes | Yes | A | G | 0.17 | -0.016 | 0.002 | -0.013 | 0.023 | 0.016 | 0.027 |
| rs7095788 | 10 | 95384152 | Yes | Yes | T | C | 0.35 | -0.011 | 0.002 | -0.02 | 0.018 | -0.009 | 0.021 |
| rs35011531 | 10 | 113032095 | Yes | No | T | C | 0.09 | -0.033 | 0.003 | -0.04 | 0.032 | -0.008 | 0.039 |
| rs12784552 | 10 | 113036354 | Yes | Yes | A | G | 0.91 | 0.033 | 0.003 | 0.039 | 0.032 | 0.008 | 0.038 |
| rs11195538 | 10 | 113117650 | Yes | Yes | T | C | 0.93 | 0.019 | 0.004 | 0.019 | 0.031 | -0.075 | 0.037 |
| rs61875120 | 10 | 114753259 | Yes | No | T | C | 0.79 | -0.027 | 0.002 | 0.016 | 0.021 | -0.028 | 0.025 |
| rs34872471 | 10 | 114754071 | Yes | No | T | C | 0.72 | -0.026 | 0.002 | 0.017 | 0.019 | -0.002 | 0.023 |
| rs7903146 | 10 | 114758349 | Yes | Yes | T | C | 0.27 | 0.026 | 0.002 | -0.014 | 0.019 | 0.006 | 0.023 |
| rs3842753 | 11 | 2181060 | Yes | No | T | G | 0.28 | 0.013 | 0.002 | -0.012 | 0.019 | 0.011 | 0.022 |
| rs689 | 11 | 2182224 | Yes | No | A | T | 0.28 | 0.013 | 0.002 | -0.009 | 0.019 | 0.008 | 0.022 |
| rs4930011 | 11 | 2856658 | Yes | No | C | G | 0.59 | -0.009 | 0.002 | -0.016 | 0.017 | -0.021 | 0.02 |
| rs2168101 | 11 | 8255408 | No | No | - | - | - | - | - | - | - | - | - |
| rs10838524 | 11 | 45870177 | Yes | Yes | A | G | 0.45 | 0.024 | 0.002 | -0.011 | 0.017 | -0.026 | 0.02 |
| rs7115753 | 11 | 45912013 | Yes | No | A | G | 0.46 | 0.024 | 0.002 | -0.017 | 0.017 | -0.02 | 0.02 |
| rs8914 | 11 | 46699124 | Yes | Yes | A | G | 0.11 | -0.02 | 0.003 | 0.046 | 0.027 | 0.015 | 0.033 |
| rs10501320 | 11 | 47293799 | Yes | Yes | C | G | 0.25 | -0.022 | 0.002 | 0.038 | 0.02 | 0.012 | 0.024 |
| rs10717442 | 11 | 47340680 | No | No | - | - | - | - | - | - | - | - | - |
| rs34228231 | 11 | 47820241 | Yes | No | A | G | 0.23 | -0.018 | 0.002 | 0.024 | 0.021 | -0.015 | 0.025 |
| rs1483121 | 11 | 48333360 | Yes | Yes | A | G | 0.14 | -0.016 | 0.003 | 0.008 | 0.024 | -0.029 | 0.029 |
| rs10769572 | 11 | 49313235 | Yes | Yes | A | G | 0.25 | 0.011 | 0.002 | -0.003 | 0.019 | 0.005 | 0.023 |
| rs174583 | 11 | 61609750 | Yes | No | T | C | 0.36 | -0.017 | 0.002 | 0.001 | 0.017 | 0.01 | 0.021 |
| rs77464186 | 11 | 72460398 | Yes | No | A | C | 0.82 | 0.023 | 0.002 | 0.063 | 0.022 | 0.027 | 0.026 |
| rs11603349 | 11 | 72460694 | Yes | No | T | C | 0.82 | 0.024 | 0.002 | 0.063 | 0.022 | 0.027 | 0.026 |
| rs7113297 | 11 | 92671744 | No | No | - | - | - | - | - | - | - | - | - |
| rs11523890 | 11 | 92679778 | Yes | Yes | T | C | 0.34 | 0.052 | 0.002 | 0.034 | 0.018 | -0.001 | 0.022 |
| rs10466351 | 11 | 92697981 | Yes | No | T | C | 0.38 | 0.056 | 0.002 | 0.025 | 0.017 | -0.003 | 0.021 |
| rs10830962 | 11 | 92698427 | Yes | Yes | C | G | 0.6 | -0.048 | 0.002 | -0.032 | 0.017 | 0.003 | 0.021 |
| rs10830963 | 11 | 92708710 | Yes | Yes | C | G | 0.71 | -0.077 | 0.002 | -0.028 | 0.019 | 0.024 | 0.023 |
| rs79354397 | 11 | 92708961 | Yes | No | T | C | 0.97 | -0.081 | 0.006 | -0.02 | 0.05 | 0.038 | 0.059 |
| rs2657879 | 12 | 56865338 | Yes | Yes | A | G | 0.82 | -0.012 | 0.002 | -0.028 | 0.024 | 0.034 | 0.028 |
| rs12315434 | 12 | 57780936 | Yes | No | A | C | 0.77 | 0.011 | 0.002 | -0.021 | 0.019 | -0.028 | 0.023 |
| rs6538804 | 12 | 97848910 | Yes | Yes | C | G | 0.61 | 0.014 | 0.002 | 0 | 0.017 | 0.009 | 0.021 |
| rs6489811 | 12 | 121893626 | Yes | Yes | A | G | 0.47 | -0.011 | 0.002 | -0.043 | 0.017 | -0.009 | 0.02 |
| rs7962128 | 12 | 121907336 | Yes | No | A | G | 0.44 | -0.011 | 0.002 | -0.036 | 0.017 | 0.002 | 0.02 |
| rs11610045 | 12 | 133063768 | Yes | No | A | G | 0.5 | 0.014 | 0.002 | 0.028 | 0.017 | -0.019 | 0.02 |
| rs11619319 | 13 | 28487599 | Yes | Yes | A | G | 0.78 | -0.017 | 0.002 | 0.007 | 0.021 | -0.014 | 0.025 |
| rs576674 | 13 | 33554302 | Yes | Yes | A | G | 0.84 | -0.018 | 0.002 | 0.043 | 0.025 | 0.058 | 0.029 |
| rs8003022 | 14 | 90038821 | Yes | Yes | A | G | 0.61 | -0.013 | 0.002 | -0.003 | 0.017 | -0.026 | 0.021 |
| rs35889227 | 14 | 90055468 | Yes | No | T | G | 0.62 | -0.013 | 0.002 | -0.002 | 0.017 | -0.02 | 0.021 |
| rs12888855 | 14 | 100830818 | Yes | Yes | A | C | 0.21 | -0.014 | 0.002 | 0.005 | 0.02 | -0.003 | 0.024 |
| rs7163757 | 15 | 62391608 | Yes | Yes | T | C | 0.45 | -0.022 | 0.002 | -0.001 | 0.017 | 0.034 | 0.02 |
| rs12594062 | 15 | 75102851 | Yes | No | T | C | 0.36 | 0.01 | 0.002 | 0.003 | 0.018 | -0.05 | 0.021 |
| rs11630478 | 15 | 75102923 | Yes | No | T | G | 0.64 | -0.01 | 0.002 | -0.003 | 0.018 | 0.049 | 0.021 |
| rs7178572 | 15 | 77747190 | Yes | Yes | A | G | 0.3 | -0.012 | 0.002 | 0.009 | 0.019 | -0.027 | 0.022 |
| rs11633054 | 15 | 77747276 | Yes | Yes | A | G | 0.3 | -0.013 | 0.002 | 0.009 | 0.019 | -0.027 | 0.022 |
| rs6598541 | 15 | 99271135 | Yes | Yes | A | G | 0.36 | 0.011 | 0.002 | 0.005 | 0.018 | -0.003 | 0.021 |
| rs2238435 | 16 | 4014282 | Yes | No | C | G | 0.4 | 0.011 | 0.002 | -0.051 | 0.017 | -0.006 | 0.021 |
| rs2302593 | 19 | 46196634 | No | No | - | - | - | - | - | - | - | - | - |
| rs6113722 | 20 | 22557099 | Yes | Yes | A | G | 0.04 | -0.042 | 0.004 | -0.029 | 0.045 | -0.078 | 0.054 |
| rs3833331 | 20 | 22562326 | No | No | - | - | - | - | - | - | - | - | - |
| rs1337918 | 20 | 22567608 | Yes | No | A | C | 0.04 | -0.046 | 0.005 | -0.079 | 0.05 | -0.089 | 0.059 |
| rs17265513 | 20 | 39832628 | Yes | Yes | T | C | 0.79 | -0.016 | 0.002 | -0.008 | 0.021 | -0.045 | 0.025 |
| rs39713 | 22 | 30343186 | Yes | Yes | T | C | 0.09 | -0.017 | 0.003 | 0.006 | 0.032 | 0.024 | 0.038 |
