# Supplementary material for: Impaired glucose tolerance and cardiovascular risk factors in relation to infertility: a Mendelian randomization analysis in the Norwegian Mother, Father, and Child Cohort Study
Source: Hum Reprod. 2023 Nov 8;39(2):436–41. doi: 10.1093/humrep/dead234 (PMC10833082; doi:10.1093/humrep/dead234)
Supplement: dead234_Supplementary_Table_S2 [file dead234_supplementary_table_s2.docx]

**Supplementary Table S2.** SNPs included in glycated hemoglobin-related analyses.

| **RSID** | **Chrom.** | **Position** | **Used in MR** | **Used in MR**  **+ Steiger filt.** | **Effect**  **allele** | **Other**  **allele** | **Effect**  **allele**  **freq.** | **Exposure:**  **beta** | **Exposure:**  **SE** | **Outcome**  **(women):**  **beta** | **Outcome**  **(women):**  **SE** | **Outcome**  **(men):**  **beta** | **Outcome**  **(men):**  **SE** |
| --- | --- | --- | --- | --- | --- | --- | --- | --- | --- | --- | --- | --- | --- |
| rs6674544 | 1 | 219628973 | No | No | - | - | - | - | - | - | - | - | - |
| rs77935490 | 2 | 630902 | No | No | - | - | - | - | - | - | - | - | - |
| rs1260326 | 2 | 27730940 | Yes | No | T | C | 0.39 | -0.023 | 0.002 | -0.001 | 0.018 | -0.003 | 0.021 |
| rs6713419 | 2 | 165508300 | Yes | Yes | T | C | 0.63 | 0.019 | 0.002 | 0.005 | 0.017 | 0.024 | 0.021 |
| rs75265117 | 2 | 165518799 | Yes | No | C | G | 0.88 | 0.028 | 0.003 | 0.026 | 0.026 | 0.028 | 0.031 |
| rs1128249 | 2 | 165528624 | Yes | Yes | T | G | 0.39 | -0.02 | 0.002 | -0.011 | 0.017 | -0.009 | 0.02 |
| rs13389219 | 2 | 165528876 | Yes | Yes | T | C | 0.39 | -0.02 | 0.002 | -0.011 | 0.017 | -0.008 | 0.02 |
| rs2943646 | 2 | 227099534 | Yes | Yes | A | G | 0.37 | -0.025 | 0.002 | -0.001 | 0.017 | -0.033 | 0.021 |
| rs2972145 | 2 | 227101309 | Yes | No | T | C | 0.37 | -0.025 | 0.002 | -0.001 | 0.017 | -0.032 | 0.021 |
| rs308971 | 3 | 12116620 | Yes | Yes | A | G | 0.87 | -0.022 | 0.003 | 0.017 | 0.025 | -0.044 | 0.029 |
| rs11712037 | 3 | 12344730 | Yes | No | C | G | 0.87 | 0.028 | 0.003 | 0.044 | 0.024 | 0.047 | 0.029 |
| rs35000407 | 3 | 12351521 | Yes | No | T | G | 0.86 | 0.026 | 0.003 | 0.041 | 0.024 | 0.047 | 0.029 |
| rs9819511 | 3 | 49884261 | Yes | No | T | C | 0.3 | 0.014 | 0.002 | -0.014 | 0.018 | -0.002 | 0.022 |
| rs17331151 | 3 | 52844534 | Yes | Yes | T | C | 0.11 | -0.016 | 0.003 | 0.002 | 0.027 | -0.077 | 0.032 |
| rs11708067 | 3 | 123065778 | Yes | No | A | G | 0.78 | -0.014 | 0.002 | 0.037 | 0.02 | -0.012 | 0.024 |
| rs62271373 | 3 | 150066540 | Yes | No | A | T | 0.06 | 0.026 | 0.005 | 0.056 | 0.04 | -0.091 | 0.047 |
| rs2276936 | 4 | 89726283 | Yes | Yes | A | C | 0.5 | -0.012 | 0.002 | 0.006 | 0.017 | 0.03 | 0.02 |
| rs3775380 | 4 | 89739808 | Yes | Yes | A | G | 0.5 | -0.012 | 0.002 | 0.005 | 0.017 | 0.026 | 0.02 |
| rs9884482 | 4 | 106081636 | Yes | Yes | T | C | 0.61 | -0.013 | 0.002 | -0.014 | 0.017 | 0.01 | 0.021 |
| rs10010325 | 4 | 106106353 | Yes | Yes | A | C | 0.48 | 0.012 | 0.002 | 0.004 | 0.017 | -0.002 | 0.02 |
| rs11727676 | 4 | 145659064 | Yes | Yes | T | C | 0.91 | -0.02 | 0.004 | -0.039 | 0.03 | 0.004 | 0.036 |
| rs6855363 | 4 | 157670537 | Yes | Yes | T | C | 0.68 | 0.013 | 0.002 | 0.023 | 0.018 | 0.029 | 0.021 |
| rs4865796 | 5 | 53272664 | Yes | Yes | A | G | 0.68 | 0.017 | 0.002 | 0.04 | 0.018 | 0.014 | 0.022 |
| rs459193 | 5 | 55806751 | Yes | Yes | A | G | 0.27 | -0.018 | 0.002 | -0.023 | 0.019 | -0.031 | 0.023 |
| rs3936511 | 5 | 55860781 | Yes | Yes | A | G | 0.82 | -0.019 | 0.003 | -0.031 | 0.023 | 0.043 | 0.028 |
| rs116141873 | 6 | 34222201 | Yes | No | T | G | 0.04 | 0.043 | 0.006 | 0.071 | 0.043 | 0.079 | 0.051 |
| rs2780215 | 6 | 34236973 | No | No | - | - | - | - | - | - | - | - | - |
| rs998584 | 6 | 43757896 | Yes | No | A | C | 0.49 | 0.012 | 0.002 | 0.01 | 0.017 | -0.008 | 0.02 |
| rs9472135 | 6 | 43809802 | Yes | Yes | T | C | 0.7 | 0.011 | 0.002 | 0.038 | 0.018 | -0.013 | 0.022 |
| rs1474696 | 6 | 127449246 | Yes | No | A | G | 0.49 | -0.015 | 0.002 | 0.011 | 0.017 | 0.001 | 0.02 |
| rs2745353 | 6 | 127452935 | Yes | Yes | T | C | 0.52 | 0.015 | 0.002 | -0.011 | 0.017 | -0.001 | 0.02 |
| rs73013411 | 6 | 164126233 | Yes | No | A | C | 0.13 | -0.018 | 0.003 | -0.012 | 0.025 | -0.029 | 0.03 |
| rs4709746 | 6 | 164133001 | Yes | No | T | C | 0.13 | -0.018 | 0.003 | -0.012 | 0.025 | -0.031 | 0.03 |
| rs2108349 | 7 | 50786663 | Yes | No | A | G | 0.66 | -0.012 | 0.002 | -0.021 | 0.018 | -0.023 | 0.021 |
| rs13234269 | 7 | 130429186 | No | No | - | - | - | - | - | - | - | - | - |
| rs972283 | 7 | 130466854 | Yes | No | A | G | 0.47 | -0.011 | 0.002 | -0.006 | 0.017 | 0.026 | 0.02 |
| rs330945 | 8 | 9021933 | Yes | No | T | C | 0.63 | 0.014 | 0.002 | 0.012 | 0.018 | 0.037 | 0.021 |
| rs7012637 | 8 | 9173209 | Yes | No | A | G | 0.48 | -0.022 | 0.002 | -0.007 | 0.017 | 0.014 | 0.02 |
| rs7012814 | 8 | 9173358 | Yes | No | A | G | 0.48 | -0.022 | 0.002 | -0.007 | 0.017 | 0.013 | 0.02 |
| rs4841132 | 8 | 9183596 | Yes | No | A | G | 0.11 | 0.026 | 0.003 | 0.005 | 0.027 | -0.044 | 0.033 |
| rs13258890 | 8 | 23615445 | Yes | Yes | T | C | 0.77 | 0.013 | 0.003 | -0.004 | 0.02 | -0.021 | 0.024 |
| rs75179845 | 9 | 136132954 | Yes | No | T | C | 0.91 | -0.022 | 0.004 | -0.027 | 0.035 | 0.057 | 0.042 |
| rs8176693 | 9 | 136137657 | Yes | Yes | T | C | 0.1 | 0.02 | 0.003 | 0.023 | 0.035 | -0.055 | 0.042 |
| rs118164457 | 10 | 89680631 | Yes | No | T | C | 0.96 | -0.035 | 0.006 | 0.095 | 0.046 | 0.076 | 0.054 |
| rs12769346 | 10 | 89764490 | Yes | Yes | T | G | 0.86 | -0.015 | 0.003 | 0.042 | 0.024 | 0.016 | 0.029 |
| rs7903146 | 10 | 114758349 | Yes | No | T | C | 0.27 | -0.012 | 0.002 | -0.014 | 0.019 | 0.006 | 0.023 |
| rs2845885 | 11 | 63869062 | Yes | Yes | T | C | 0.93 | -0.02 | 0.004 | 0.048 | 0.035 | 0.06 | 0.042 |
| rs2054435 | 12 | 21696684 | Yes | Yes | A | G | 0.22 | -0.015 | 0.003 | -0.03 | 0.021 | 0.009 | 0.026 |
| rs6487237 | 12 | 21699928 | Yes | Yes | A | C | 0.78 | 0.015 | 0.003 | 0.03 | 0.021 | -0.007 | 0.026 |
| rs111264094 | 12 | 48202696 | Yes | No | C | G | 0.97 | 0.057 | 0.009 | 0.124 | 0.071 | -0.107 | 0.084 |
| rs1351394 | 12 | 66351826 | Yes | Yes | T | C | 0.49 | -0.011 | 0.002 | 0.026 | 0.017 | 0.035 | 0.02 |
| rs7968682 | 12 | 66371880 | Yes | Yes | T | G | 0.51 | 0.011 | 0.002 | -0.02 | 0.017 | -0.026 | 0.02 |
| rs1402013 | 12 | 102740822 | Yes | Yes | A | G | 0.35 | -0.009 | 0.002 | 0.014 | 0.018 | 0.02 | 0.022 |
| rs860598 | 12 | 102898446 | Yes | Yes | A | G | 0.82 | 0.018 | 0.003 | 0.029 | 0.023 | -0.01 | 0.028 |
| rs35747 | 12 | 102912558 | Yes | Yes | A | G | 0.82 | 0.017 | 0.003 | 0.03 | 0.023 | -0.012 | 0.028 |
| rs7133378 | 12 | 124409502 | Yes | No | A | G | 0.32 | -0.013 | 0.002 | -0.005 | 0.018 | -0.012 | 0.022 |
| rs7975482 | 12 | 124481690 | Yes | No | A | G | 0.67 | 0.012 | 0.002 | 0.011 | 0.018 | 0.018 | 0.021 |
| rs12454712 | 18 | 60845884 | No | No | - | - | - | - | - | - | - | - | - |
| rs10422861 | 19 | 33894846 | Yes | No | T | C | 0.67 | -0.013 | 0.002 | 0.006 | 0.018 | -0.016 | 0.021 |
| rs731839 | 19 | 33899065 | Yes | Yes | A | G | 0.66 | -0.012 | 0.002 | 0.006 | 0.018 | -0.015 | 0.021 |
| rs1999536 | 20 | 45581777 | No | No | - | - | - | - | - | - | - | - | - |
| rs1206760 | 20 | 45582472 | Yes | Yes | A | G | 0.54 | -0.011 | 0.002 | -0.014 | 0.017 | -0.015 | 0.02 |
| rs200678953 | 21 | 24493294 | No | No | - | - | - | - | - | - | - | - | - |
