# Supplementary material for: Impaired glucose tolerance and cardiovascular risk factors in relation to infertility: a Mendelian randomization analysis in the Norwegian Mother, Father, and Child Cohort Study
Source: Hum Reprod. 2023 Nov 8;39(2):436–41. doi: 10.1093/humrep/dead234 (PMC10833082; doi:10.1093/humrep/dead234)
Supplement: dead234_Supplementary_Table_S3 [file dead234_supplementary_table_s3.docx]

**Supplementary Table S3.** SNPs included in fasting insulin-related analyses.

| **RSID** | **Chrom.** | **Position** | **Used in MR** | **Used in MR**  **+ Steiger filt.** | **Effect**  **allele** | **Other**  **allele** | **Effect**  **allele**  **freq.** | **Exposure:**  **beta** | **Exposure:**  **SE** | **Outcome**  **(women):**  **beta** | **Outcome**  **(women):**  **SE** | **Outcome**  **(men):**  **beta** | **Outcome**  **(men):**  **SE** |
| --- | --- | --- | --- | --- | --- | --- | --- | --- | --- | --- | --- | --- | --- |
| rs1175549 | 1 | 3691727 | Yes | Yes | A | C | 0.75 | 0.01 | 0.002 | 0.011 | 0.021 | 0.04 | 0.025 |
| rs2375278 | 1 | 25529038 | Yes | Yes | A | G | 0.17 | 0.011 | 0.002 | -0.027 | 0.022 | -0.021 | 0.026 |
| rs644592 | 1 | 25703156 | Yes | No | T | C | 0.15 | 0.013 | 0.002 | -0.026 | 0.022 | -0.037 | 0.027 |
| rs267738 | 1 | 150940625 | Yes | Yes | T | G | 0.79 | 0.011 | 0.002 | -0.001 | 0.021 | 0.015 | 0.024 |
| rs7534795 | 1 | 155275553 | No | No | - | - | - | - | - | - | - | - | - |
| rs10796941 | 1 | 155284261 | Yes | No | T | C | 0.25 | 0.01 | 0.002 | -0.037 | 0.02 | -0.007 | 0.024 |
| rs857725 | 1 | 158607935 | Yes | Yes | T | G | 0.73 | -0.021 | 0.001 | 0.005 | 0.019 | -0.033 | 0.023 |
| rs857676 | 1 | 158615702 | Yes | Yes | A | G | 0.57 | 0.01 | 0.001 | 0.003 | 0.017 | 0.027 | 0.02 |
| rs7547793 | 1 | 203653544 | Yes | No | A | C | 0.13 | -0.012 | 0.002 | 0.01 | 0.026 | -0.027 | 0.031 |
| rs10900585 | 1 | 203654024 | Yes | No | T | G | 0.88 | 0.012 | 0.002 | -0.011 | 0.026 | 0.028 | 0.031 |
| rs340882 | 1 | 214145731 | Yes | No | C | G | 0.41 | -0.008 | 0.001 | -0.067 | 0.017 | -0.019 | 0.021 |
| rs12612492 | 2 | 24093756 | Yes | Yes | T | C | 0.12 | 0.019 | 0.002 | -0.001 | 0.026 | -0.013 | 0.031 |
| rs6545222 | 2 | 24235704 | Yes | Yes | A | G | 0.75 | 0.01 | 0.002 | 0.047 | 0.019 | 0.009 | 0.023 |
| rs1367173 | 2 | 43449385 | Yes | Yes | T | C | 0.12 | -0.015 | 0.002 | -0.028 | 0.027 | -0.044 | 0.033 |
| rs6723441 | 2 | 48123915 | Yes | Yes | A | T | 0.81 | -0.009 | 0.002 | 0.002 | 0.021 | 0.013 | 0.025 |
| rs17037289 | 2 | 48587198 | Yes | No | A | G | 0.75 | -0.009 | 0.002 | -0.027 | 0.019 | -0.006 | 0.022 |
| rs150171632 | 2 | 169748691 | No | No | - | - | - | - | - | - | - | - | - |
| rs540524 | 2 | 169756930 | Yes | No | A | G | 0.63 | -0.002 | 0.001 | 0.025 | 0.017 | -0.035 | 0.021 |
| rs560887 | 2 | 169763148 | Yes | No | T | C | 0.3 | -0.031 | 0.001 | 0.022 | 0.019 | -0.014 | 0.022 |
| rs13419326 | 2 | 169771420 | Yes | No | A | C | 0.98 | -0.032 | 0.005 | 0.109 | 0.06 | 0.001 | 0.072 |
| rs557462 | 2 | 169777595 | Yes | No | T | C | 0.65 | 0.027 | 0.001 | -0.017 | 0.018 | 0.009 | 0.021 |
| rs56100844 | 2 | 169786707 | Yes | No | T | G | 0.98 | 0.058 | 0.007 | -0.006 | 0.072 | -0.005 | 0.085 |
| rs17256082 | 2 | 175292364 | Yes | Yes | T | C | 0.67 | -0.007 | 0.001 | 0.001 | 0.018 | 0.007 | 0.021 |
| rs4674280 | 2 | 219141458 | No | No | - | - | - | - | - | - | - | - | - |
| rs13427681 | 2 | 219167563 | No | No | - | - | - | - | - | - | - | - | - |
| rs1822534 | 3 | 12266804 | Yes | No | A | G | 0.62 | 0.008 | 0.001 | -0.029 | 0.018 | 0.018 | 0.021 |
| rs12491937 | 3 | 12268244 | Yes | No | A | G | 0.58 | 0.009 | 0.001 | -0.023 | 0.017 | 0.011 | 0.021 |
| rs9818758 | 3 | 49382925 | Yes | Yes | A | G | 0.18 | 0.013 | 0.002 | 0.034 | 0.022 | 0.01 | 0.026 |
| rs66499923 | 3 | 52887861 | No | No | - | - | - | - | - | - | - | - | - |
| rs11719201 | 3 | 123068744 | Yes | No | T | C | 0.24 | -0.013 | 0.002 | -0.042 | 0.02 | 0.008 | 0.024 |
| rs1604038 | 3 | 170709193 | Yes | No | T | C | 0.29 | -0.011 | 0.001 | -0.007 | 0.018 | 0.027 | 0.022 |
| rs4894769 | 3 | 171516306 | No | No | - | - | - | - | - | - | - | - | - |
| rs13089972 | 3 | 171798694 | No | No | - | - | - | - | - | - | - | - | - |
| rs7632281 | 3 | 171812293 | Yes | No | T | G | 0.43 | -0.011 | 0.001 | 0.001 | 0.017 | -0.002 | 0.02 |
| rs13134327 | 4 | 144659795 | Yes | Yes | A | G | 0.33 | 0.014 | 0.001 | -0.004 | 0.018 | 0.013 | 0.022 |
| rs13129993 | 4 | 144684229 | Yes | Yes | A | T | 0.32 | 0.015 | 0.002 | -0.003 | 0.018 | 0.013 | 0.022 |
| rs112578089 | 4 | 145128105 | No | No | - | - | - | - | - | - | - | - | - |
| rs77909720 | 4 | 145222284 | No | No | - | - | - | - | - | - | - | - | - |
| rs6877043 | 5 | 154048367 | Yes | Yes | T | C | 0.64 | 0.009 | 0.001 | -0.006 | 0.017 | -0.006 | 0.021 |
| rs1948759 | 5 | 156442657 | Yes | No | A | G | 0.17 | -0.01 | 0.002 | -0.023 | 0.022 | -0.01 | 0.027 |
| rs3778321 | 6 | 7250270 | Yes | No | A | G | 0.19 | -0.011 | 0.002 | 0.032 | 0.021 | -0.018 | 0.025 |
| rs34499031 | 6 | 20676414 | No | No | - | - | - | - | - | - | - | - | - |
| rs35612982 | 6 | 20682622 | Yes | No | T | C | 0.82 | -0.01 | 0.002 | -0.036 | 0.023 | 0.025 | 0.027 |
| rs6931514 | 6 | 20703952 | Yes | No | A | G | 0.73 | -0.01 | 0.001 | -0.008 | 0.019 | 0.024 | 0.023 |
| rs12193223 | 6 | 24978511 | Yes | Yes | C | G | 0.94 | 0.02 | 0.003 | 0.039 | 0.036 | 0.092 | 0.044 |
| rs75580845 | 6 | 25578433 | Yes | No | T | C | 0.92 | 0.02 | 0.002 | 0.032 | 0.03 | 0.031 | 0.036 |
| rs1799945 | 6 | 26091179 | Yes | Yes | C | G | 0.86 | 0.025 | 0.002 | 0.005 | 0.027 | -0.058 | 0.032 |
| rs1800562 | 6 | 26093141 | Yes | Yes | A | G | 0.06 | -0.038 | 0.003 | -0.013 | 0.033 | -0.05 | 0.04 |
| rs13194491 | 6 | 27037080 | No | No | - | - | - | - | - | - | - | - | - |
| rs13214703 | 6 | 27941387 | Yes | Yes | T | C | 0.93 | 0.02 | 0.003 | 0.041 | 0.031 | 0.053 | 0.036 |
| rs34979126 | 6 | 28449380 | Yes | No | A | G | 0.07 | -0.018 | 0.003 | -0.047 | 0.031 | -0.034 | 0.036 |
| rs116735744 | 6 | 28984755 | No | No | - | - | - | - | - | - | - | - | - |
| rs6929796 | 6 | 31522669 | Yes | Yes | A | G | 0.17 | -0.009 | 0.002 | -0.011 | 0.021 | -0.013 | 0.026 |
| rs9376090 | 6 | 135411228 | Yes | Yes | T | C | 0.73 | 0.025 | 0.001 | 0.025 | 0.019 | 0 | 0.022 |
| rs9389268 | 6 | 135419631 | Yes | Yes | A | G | 0.73 | 0.023 | 0.002 | 0.024 | 0.019 | -0.006 | 0.022 |
| rs10231021 | 7 | 15060429 | No | No | - | - | - | - | - | - | - | - | - |
| rs2191349 | 7 | 15064309 | Yes | No | T | G | 0.53 | 0.008 | 0.001 | -0.008 | 0.017 | -0.027 | 0.02 |
| rs10259649 | 7 | 44219705 | Yes | No | T | C | 0.77 | -0.024 | 0.002 | -0.037 | 0.021 | -0.021 | 0.025 |
| rs2971670 | 7 | 44226101 | Yes | No | T | C | 0.17 | 0.032 | 0.002 | 0.013 | 0.023 | 0.022 | 0.028 |
| rs1799884 | 7 | 44229068 | Yes | No | T | C | 0.17 | 0.032 | 0.002 | 0.013 | 0.023 | 0.023 | 0.028 |
| rs3757840 | 7 | 44231216 | Yes | No | T | G | 0.5 | 0.022 | 0.001 | 0.024 | 0.017 | 0.012 | 0.02 |
| rs2908286 | 7 | 44234737 | Yes | No | T | C | 0.17 | 0.032 | 0.002 | 0.013 | 0.024 | 0.022 | 0.028 |
| rs35332062 | 7 | 73012042 | Yes | No | A | G | 0.12 | 0.011 | 0.002 | 0.012 | 0.025 | -0.064 | 0.029 |
| rs13234131 | 7 | 73025975 | Yes | No | A | G | 0.87 | -0.011 | 0.002 | -0.004 | 0.025 | 0.069 | 0.03 |
| rs4731113 | 7 | 123283949 | Yes | No | T | C | 0.96 | 0.02 | 0.004 | 0.021 | 0.043 | -0.016 | 0.052 |
| rs4317621 | 8 | 41516581 | Yes | No | A | G | 0.42 | -0.004 | 0.001 | -0.012 | 0.017 | 0.013 | 0.02 |
| rs34664882 | 8 | 41543675 | Yes | No | A | G | 0.03 | -0.049 | 0.004 | -0.06 | 0.051 | 0.027 | 0.062 |
| rs4737009 | 8 | 41630405 | Yes | Yes | A | G | 0.24 | 0.023 | 0.002 | -0.03 | 0.02 | 0.001 | 0.024 |
| rs6980507 | 8 | 42383084 | Yes | Yes | A | G | 0.4 | 0.011 | 0.001 | -0.004 | 0.017 | -0.008 | 0.021 |
| rs11558471 | 8 | 118185733 | Yes | No | A | G | 0.68 | 0.015 | 0.001 | 0.023 | 0.018 | 0.02 | 0.021 |
| rs35859536 | 8 | 118191475 | Yes | No | T | C | 0.32 | -0.015 | 0.001 | -0.015 | 0.018 | -0.018 | 0.022 |
| rs2954021 | 8 | 126482077 | Yes | No | A | G | 0.49 | -0.007 | 0.001 | 0.008 | 0.017 | -0.014 | 0.02 |
| rs10811661 | 9 | 22134094 | Yes | No | T | C | 0.82 | 0.013 | 0.002 | -0.034 | 0.023 | -0.034 | 0.027 |
| rs10811662 | 9 | 22134253 | Yes | No | A | G | 0.18 | -0.013 | 0.002 | 0.035 | 0.023 | 0.033 | 0.027 |
| rs7847351 | 9 | 79977312 | Yes | No | A | G | 0.81 | -0.013 | 0.002 | 0.029 | 0.021 | 0.049 | 0.025 |
| rs12351997 | 9 | 80015424 | Yes | Yes | T | C | 0.8 | -0.013 | 0.002 | 0.024 | 0.021 | 0.051 | 0.025 |
| rs61750929 | 9 | 91495135 | Yes | No | T | C | 0.06 | -0.028 | 0.003 | -0.008 | 0.037 | 0.012 | 0.045 |
| rs7042939 | 9 | 110511408 | Yes | Yes | A | G | 0.4 | 0.01 | 0.001 | 0.018 | 0.017 | -0.007 | 0.02 |
| rs1467311 | 9 | 110536932 | Yes | Yes | A | G | 0.66 | -0.011 | 0.001 | -0.015 | 0.018 | 0.001 | 0.021 |
| rs649129 | 9 | 136154304 | Yes | No | T | C | 0.22 | 0.011 | 0.002 | 0.021 | 0.02 | 0.022 | 0.023 |
| rs3829109 | 9 | 139256766 | Yes | No | A | G | 0.3 | -0.009 | 0.002 | -0.006 | 0.018 | -0.007 | 0.022 |
| rs11257655 | 10 | 12307894 | Yes | Yes | T | C | 0.22 | 0.011 | 0.002 | -0.005 | 0.02 | 0.016 | 0.024 |
| rs5785903 | 10 | 71002040 | No | No | - | - | - | - | - | - | - | - | - |
| rs4745982 | 10 | 71089843 | Yes | Yes | T | G | 0.92 | 0.074 | 0.003 | 0.021 | 0.032 | -0.04 | 0.038 |
| rs150705486 | 10 | 71093216 | Yes | No | A | G | 0.02 | -0.114 | 0.006 | -0.116 | 0.074 | -0.039 | 0.087 |
| rs17476364 | 10 | 71094504 | Yes | No | T | C | 0.9 | 0.086 | 0.002 | 0.018 | 0.027 | -0.013 | 0.032 |
| rs72805692 | 10 | 71099109 | Yes | No | A | G | 0.89 | 0.079 | 0.002 | 0.022 | 0.026 | -0.005 | 0.031 |
| rs200572185 | 10 | 71118821 | No | No | - | - | - | - | - | - | - | - | - |
| rs7903146 | 10 | 114758349 | Yes | No | T | C | 0.28 | 0.013 | 0.001 | -0.014 | 0.019 | 0.006 | 0.023 |
| rs4980325 | 11 | 234451 | Yes | No | T | G | 0.53 | 0.011 | 0.001 | 0.023 | 0.017 | 0.004 | 0.021 |
| rs3842753 | 11 | 2181060 | Yes | No | T | G | 0.28 | 0.008 | 0.002 | -0.012 | 0.019 | 0.011 | 0.022 |
| rs373894 | 11 | 9763094 | Yes | Yes | A | C | 0.72 | 0.008 | 0.002 | 0.023 | 0.019 | -0.053 | 0.023 |
| rs360140 | 11 | 9776567 | Yes | Yes | A | C | 0.66 | -0.008 | 0.001 | -0.001 | 0.018 | 0.036 | 0.021 |
| rs117706710 | 11 | 10508903 | Yes | No | T | G | 0.009 | 0.046 | 0.009 | 0.02 | 0.075 | -0.075 | 0.091 |
| rs11039154 | 11 | 47278502 | Yes | No | T | C | 0.28 | -0.009 | 0.001 | 0.032 | 0.02 | 0.012 | 0.024 |
| rs10838696 | 11 | 47363285 | Yes | No | A | G | 0.35 | -0.007 | 0.001 | 0.007 | 0.018 | 0.008 | 0.022 |
| rs174559 | 11 | 61581656 | Yes | No | A | G | 0.27 | -0.011 | 0.001 | -0.009 | 0.019 | 0.009 | 0.022 |
| rs174584 | 11 | 61610750 | Yes | No | A | G | 0.35 | -0.009 | 0.001 | 0.001 | 0.017 | 0.009 | 0.021 |
| rs10466351 | 11 | 92697981 | Yes | No | T | C | 0.38 | 0.015 | 0.001 | 0.025 | 0.017 | -0.003 | 0.021 |
| rs10830963 | 11 | 92708710 | Yes | No | C | G | 0.72 | -0.02 | 0.002 | -0.028 | 0.019 | 0.024 | 0.023 |
| rs11224302 | 11 | 100456604 | Yes | Yes | T | C | 0.1 | -0.016 | 0.002 | -0.062 | 0.029 | 0.005 | 0.034 |
| rs117233107 | 12 | 4328521 | Yes | No | A | G | 0.02 | -0.047 | 0.007 | 0.021 | 0.065 | 0.03 | 0.077 |
| rs2110073 | 12 | 7075882 | Yes | Yes | T | C | 0.1 | 0.012 | 0.002 | 0.015 | 0.028 | -0.068 | 0.033 |
| rs76261711 | 12 | 48486696 | Yes | No | A | T | 0.9 | 0.014 | 0.002 | 0.03 | 0.027 | -0.013 | 0.033 |
| rs4760682 | 12 | 48512285 | Yes | No | A | C | 0.8 | 0.016 | 0.002 | -0.028 | 0.022 | 0.008 | 0.026 |
| rs10774624 | 12 | 111833788 | Yes | No | A | G | 0.52 | 0.009 | 0.001 | 0.012 | 0.017 | 0.035 | 0.02 |
| rs17696736 | 12 | 112486818 | Yes | No | A | G | 0.57 | 0.009 | 0.001 | -0.001 | 0.017 | 0.024 | 0.021 |
| rs4766971 | 12 | 113018479 | Yes | No | A | G | 0.09 | 0.01 | 0.002 | 0.011 | 0.028 | 0.012 | 0.034 |
| rs76533333 | 13 | 113352916 | Yes | No | A | G | 0.92 | -0.027 | 0.003 | 0.016 | 0.03 | 0.042 | 0.035 |
| rs1278769 | 13 | 113536627 | Yes | Yes | A | G | 0.24 | -0.009 | 0.002 | -0.033 | 0.02 | 0.025 | 0.024 |
| rs7994900 | 13 | 114553134 | Yes | No | T | C | 0.27 | 0.011 | 0.002 | 0.002 | 0.019 | 0.014 | 0.023 |
| rs2273475 | 14 | 65268605 | Yes | Yes | A | G | 0.9 | -0.013 | 0.002 | 0.007 | 0.032 | 0.019 | 0.038 |
| rs147727276 | 14 | 73601025 | Yes | No | A | G | 0.1 | -0.013 | 0.002 | 0.023 | 0.03 | 0.044 | 0.036 |
| rs10151436 | 14 | 73616095 | Yes | Yes | A | T | 0.89 | 0.013 | 0.002 | -0.015 | 0.028 | -0.042 | 0.033 |
| rs1535464 | 14 | 100793431 | Yes | No | A | G | 0.23 | -0.009 | 0.002 | 0.011 | 0.02 | 0 | 0.024 |
| rs33952550 | 14 | 100798141 | No | No | - | - | - | - | - | - | - | - | - |
| rs452306 | 15 | 65822777 | Yes | No | T | C | 0.59 | -0.01 | 0.001 | 0.025 | 0.017 | -0.007 | 0.021 |
| rs368777 | 15 | 65849552 | Yes | No | A | G | 0.57 | -0.009 | 0.001 | 0.026 | 0.017 | -0.005 | 0.021 |
| rs11248914 | 16 | 293562 | Yes | Yes | T | C | 0.66 | 0.011 | 0.001 | -0.015 | 0.018 | 0.027 | 0.021 |
| rs9926463 | 16 | 11437607 | Yes | No | A | C | 0.31 | 0.008 | 0.002 | -0.003 | 0.019 | -0.003 | 0.022 |
| rs11643024 | 16 | 11443183 | Yes | No | A | G | 0.31 | 0.008 | 0.002 | -0.01 | 0.018 | -0.005 | 0.022 |
| rs4787458 | 16 | 28531287 | Yes | No | A | G | 0.62 | -0.008 | 0.001 | 0.022 | 0.017 | -0.016 | 0.021 |
| rs7190771 | 16 | 28590030 | No | No | - | - | - | - | - | - | - | - | - |
| rs7198799 | 16 | 68818390 | Yes | Yes | T | C | 0.29 | 0.008 | 0.001 | 0.008 | 0.019 | 0.018 | 0.022 |
| rs1862748 | 16 | 68832943 | Yes | Yes | T | C | 0.3 | 0.008 | 0.001 | 0.013 | 0.019 | 0.02 | 0.022 |
| rs4238685 | 16 | 88775220 | Yes | Yes | T | C | 0.1 | 0.006 | 0.002 | 0.004 | 0.028 | -0.008 | 0.033 |
| rs837763 | 16 | 88853729 | Yes | Yes | T | C | 0.56 | 0.018 | 0.001 | -0.001 | 0.017 | -0.017 | 0.02 |
| rs8075153 | 17 | 17622666 | Yes | Yes | T | C | 0.43 | -0.007 | 0.001 | -0.005 | 0.017 | 0.01 | 0.02 |
| rs9914988 | 17 | 27183104 | Yes | Yes | A | G | 0.8 | 0.013 | 0.002 | -0.034 | 0.022 | -0.101 | 0.026 |
| rs2748427 | 17 | 76121864 | No | No | - | - | - | - | - | - | - | - | - |
| rs2748424 | 17 | 76124865 | No | No | - | - | - | - | - | - | - | - | - |
| rs200350733 | 17 | 76125194 | No | No | - | - | - | - | - | - | - | - | - |
| rs72634341 | 17 | 80682778 | Yes | No | A | G | 0.72 | -0.031 | 0.002 | 0.016 | 0.019 | 0.001 | 0.023 |
| rs9909940 | 17 | 80689036 | Yes | No | T | C | 0.31 | 0.032 | 0.001 | -0.018 | 0.019 | -0.004 | 0.022 |
| rs62076520 | 17 | 80695406 | Yes | No | A | G | 0.37 | -0.026 | 0.001 | 0.01 | 0.017 | 0.013 | 0.021 |
| rs113373052 | 17 | 80697458 | Yes | No | T | C | 0.3 | 0.033 | 0.001 | -0.019 | 0.019 | -0.004 | 0.022 |
| rs12452572 | 17 | 80702963 | No | No | - | - | - | - | - | - | - | - | - |
| rs191734192 | 17 | 80778724 | No | No | - | - | - | - | - | - | - | - | - |
| rs141786856 | 17 | 80950648 | No | No | - | - | - | - | - | - | - | - | - |
| rs28671200 | 18 | 43774444 | No | No | - | - | - | - | - | - | - | - | - |
| rs12982956 | 19 | 17238413 | Yes | No | A | G | 0.72 | 0.007 | 0.002 | -0.012 | 0.018 | 0.033 | 0.022 |
| rs17533945 | 19 | 17257802 | Yes | Yes | T | C | 0.6 | -0.013 | 0.001 | 0.022 | 0.017 | -0.011 | 0.021 |
| rs12978547 | 19 | 33037212 | Yes | Yes | C | G | 0.97 | 0.028 | 0.004 | 0.036 | 0.044 | 0.028 | 0.053 |
| rs10405535 | 19 | 33072085 | Yes | No | A | G | 0.3 | 0.012 | 0.002 | -0.019 | 0.019 | 0.01 | 0.022 |
| rs4499344 | 19 | 33073431 | Yes | Yes | A | G | 0.31 | -0.011 | 0.002 | 0.013 | 0.018 | -0.02 | 0.022 |
| rs737092 | 20 | 55990405 | Yes | Yes | T | C | 0.51 | -0.007 | 0.001 | 0.009 | 0.017 | 0.027 | 0.02 |
| rs855791 | 22 | 37462936 | Yes | Yes | A | G | 0.43 | 0.019 | 0.001 | -0.007 | 0.017 | 0.008 | 0.02 |
| rs2143923 | 22 | 43141907 | Yes | No | A | G | 0.48 | 0.007 | 0.001 | 0.005 | 0.017 | -0.004 | 0.02 |
