# Supplementary material for: Impaired glucose tolerance and cardiovascular risk factors in relation to infertility: a Mendelian randomization analysis in the Norwegian Mother, Father, and Child Cohort Study
Source: Hum Reprod. 2023 Nov 8;39(2):436–41. doi: 10.1093/humrep/dead234 (PMC10833082; doi:10.1093/humrep/dead234)
Supplement: dead234_Supplementary_Table_S4 [file dead234_supplementary_table_s4.docx]

**Supplementary Table S4.** SNPs included in LDL cholesterol-related analyses.

| **RSID** | **Chrom.** | **Position** | **Used in MR** | **Used in MR**  **+ Steiger filt.** | **Effect**  **allele** | **Other**  **allele** | **Effect**  **allele**  **freq.** | **Exposure:**  **beta** | **Exposure:**  **SE** | **Outcome**  **(women):**  **beta** | **Outcome**  **(women):**  **SE** | **Outcome**  **(men):**  **beta** | **Outcome**  **(men):**  **SE** |
| --- | --- | --- | --- | --- | --- | --- | --- | --- | --- | --- | --- | --- | --- |
| rs1123571 | 1 | 2326009 | Yes | Yes | A | G | 0.469 | -0.011 | 0 | -0.002 | 0.017 | -0.02 | 0.02 |
| rs1497406 | 1 | 16505320 | Yes | Yes | G | A | 0.584 | 0.014 | 0 | 0.016 | 0.017 | 0.019 | 0.021 |
| rs2992752 | 1 | 18808526 | Yes | No | C | A | 0.63 | -0.01 | 0 | 0.019 | 0.018 | -0.011 | 0.021 |
| rs7551124 | 1 | 23785760 | Yes | No | T | C | 0.874 | 0.017 | 0 | 0.002 | 0.024 | 0.008 | 0.029 |
| rs10903129 | 1 | 25768937 | Yes | Yes | G | A | 0.548 | 0.026 | 0 | -0.021 | 0.017 | -0.014 | 0.02 |
| rs41311276 | 1 | 26387625 | Yes | No | C | A | 0.175 | -0.014 | 0 | 0.03 | 0.022 | 0.066 | 0.026 |
| rs114165349 | 1 | 27021913 | Yes | No | C | G | 0.023 | 0.084 | 0 | 0.012 | 0.049 | -0.002 | 0.058 |
| rs41309280 | 1 | 27688814 | Yes | No | T | C | 0.037 | 0.039 | 0 | 0.033 | 0.038 | -0.037 | 0.046 |
| rs17853159 | 1 | 45810865 | Yes | No | A | G | 0.078 | -0.022 | 0 | -0.014 | 0.032 | 0.014 | 0.038 |
| rs143064733 | 1 | 49204135 | No | No | - | - | - | - | - | - | - | - | - |
| rs570553136 | 1 | 52262405 | No | No | - | - | - | - | - | - | - | - | - |
| rs140559632 | 1 | 53367652 | No | No | - | - | - | - | - | - | - | - | - |
| rs191875604 | 1 | 54246772 | No | No | - | - | - | - | - | - | - | - | - |
| rs11591147 | 1 | 55505647 | No | No | - | - | - | - | - | - | - | - | - |
| rs557402933 | 1 | 56339701 | No | No | - | - | - | - | - | - | - | - | - |
| rs186538116 | 1 | 56840574 | No | No | - | - | - | - | - | - | - | - | - |
| rs754633424 | 1 | 57390571 | No | No | - | - | - | - | - | - | - | - | - |
| rs145440120 | 1 | 58137322 | No | No | - | - | - | - | - | - | - | - | - |
| rs17121798 | 1 | 61675857 | Yes | No | G | C | 0.074 | -0.018 | 0 | 0.017 | 0.033 | 0.03 | 0.039 |
| rs598253 | 1 | 62911341 | Yes | No | C | T | 0.338 | -0.042 | 0 | 0.02 | 0.018 | 0.009 | 0.021 |
| rs566523636 | 1 | 63421381 | No | No | - | - | - | - | - | - | - | - | - |
| rs2391159 | 1 | 92971951 | Yes | No | C | T | 0.793 | 0.023 | 0 | 0.021 | 0.021 | 0.002 | 0.025 |
| rs111232683 | 1 | 107566149 | No | No | - | - | - | - | - | - | - | - | - |
| rs115292790 | 1 | 109310728 | Yes | No | A | G | 0.014 | -0.056 | 0 | -0.051 | 0.069 | -0.024 | 0.082 |
| rs12740374 | 1 | 109817590 | Yes | Yes | T | G | 0.219 | -0.148 | 0 | 0.001 | 0.02 | 0.018 | 0.024 |
| rs140266316 | 1 | 110326545 | Yes | No | A | G | 0.016 | -0.058 | 0 | 0.135 | 0.064 | -0.053 | 0.076 |
| rs267733 | 1 | 150958836 | Yes | No | G | A | 0.156 | -0.019 | 0 | -0.01 | 0.023 | -0.007 | 0.028 |
| rs184980867 | 1 | 151658543 | No | No | - | - | - | - | - | - | - | - | - |
| rs4390169 | 1 | 155106054 | Yes | No | G | A | 0.515 | -0.012 | 0 | 0.013 | 0.017 | 0.008 | 0.02 |
| rs12045893 | 1 | 158530416 | Yes | No | T | C | 0.248 | 0.011 | 0 | -0.001 | 0.02 | 0.024 | 0.023 |
| rs115383270 | 1 | 161531340 | Yes | No | A | G | 0.073 | 0.019 | 0 | -0.009 | 0.033 | -0.047 | 0.04 |
| rs76900682 | 1 | 174064560 | Yes | No | A | G | 0.154 | 0.013 | 0 | 0.017 | 0.022 | 0.03 | 0.027 |
| rs6682862 | 1 | 177938437 | Yes | No | A | G | 0.164 | -0.014 | 0 | -0.025 | 0.022 | 0.001 | 0.027 |
| rs1689801 | 1 | 182165484 | Yes | No | A | G | 0.325 | 0.014 | 0 | -0.019 | 0.018 | -0.005 | 0.022 |
| rs2296288 | 1 | 183072590 | Yes | Yes | C | T | 0.563 | 0.011 | 0 | -0.009 | 0.017 | 0.008 | 0.02 |
| rs1434282 | 1 | 199010721 | Yes | No | T | C | 0.723 | 0.011 | 0 | 0.002 | 0.019 | -0.002 | 0.023 |
| rs2642438 | 1 | 220970028 | Yes | No | G | A | 0.706 | 0.027 | 0 | -0.006 | 0.019 | 0.008 | 0.022 |
| rs7519734 | 1 | 224549524 | Yes | No | C | T | 0.223 | -0.011 | 0 | -0.001 | 0.02 | 0.019 | 0.024 |
| rs553427 | 1 | 234852760 | Yes | Yes | T | C | 0.527 | 0.04 | 0 | -0.017 | 0.017 | 0.012 | 0.02 |
| rs3935011 | 1 | 246891269 | Yes | Yes | C | T | 0.48 | 0.009 | 0 | 0.012 | 0.017 | -0.05 | 0.02 |
| rs3820897 | 2 | 3642361 | Yes | No | C | T | 0.808 | -0.016 | 0 | 0.012 | 0.021 | 0.022 | 0.025 |
| rs67269656 | 2 | 8720038 | Yes | No | T | C | 0.265 | -0.01 | 0 | 0.035 | 0.019 | -0.039 | 0.022 |
| rs7556983 | 2 | 17955255 | Yes | Yes | A | G | 0.104 | -0.015 | 0 | -0.002 | 0.027 | -0.014 | 0.032 |
| rs79279183 | 2 | 20370641 | No | No | - | - | - | - | - | - | - | - | - |
| rs934197 | 2 | 21267461 | Yes | No | A | G | 0.33 | 0.096 | 0 | 0.003 | 0.018 | 0.02 | 0.021 |
| rs191450029 | 2 | 21942500 | No | No | - | - | - | - | - | - | - | - | - |
| rs541138574 | 2 | 22812173 | No | No | - | - | - | - | - | - | - | - | - |
| rs150520162 | 2 | 23541654 | No | No | - | - | - | - | - | - | - | - | - |
| rs142787485 | 2 | 26358156 | Yes | No | G | A | 0.036 | -0.029 | 0 | -0.001 | 0.043 | 0.02 | 0.051 |
| rs13394970 | 2 | 26929282 | Yes | No | G | T | 0.602 | 0.01 | 0 | -0.018 | 0.017 | -0.005 | 0.021 |
| rs1260326 | 2 | 27730940 | Yes | No | C | T | 0.61 | -0.031 | 0 | 0.001 | 0.018 | 0.003 | 0.021 |
| rs141647229 | 2 | 43473222 | No | No | - | - | - | - | - | - | - | - | - |
| rs4299376 | 2 | 44072576 | Yes | Yes | T | G | 0.691 | -0.07 | 0 | 0.014 | 0.018 | -0.009 | 0.022 |
| rs140879085 | 2 | 44663302 | Yes | No | T | C | 0.011 | -0.097 | 0 | -0.008 | 0.079 | 0.019 | 0.094 |
| rs12712955 | 2 | 46166321 | Yes | No | G | A | 0.503 | -0.011 | 0 | 0.008 | 0.017 | -0.003 | 0.02 |
| rs4671050 | 2 | 62988169 | Yes | Yes | T | G | 0.32 | -0.022 | 0 | 0.014 | 0.018 | 0.002 | 0.022 |
| rs954680 | 2 | 64913045 | Yes | Yes | G | C | 0.702 | 0.012 | 0 | 0.019 | 0.019 | 0.015 | 0.023 |
| rs10206764 | 2 | 70549961 | Yes | No | C | T | 0.407 | 0.011 | 0 | 0.014 | 0.017 | -0.011 | 0.02 |
| rs11887443 | 2 | 85879041 | No | No | - | - | - | - | - | - | - | - | - |
| rs2970901 | 2 | 88428834 | Yes | Yes | T | G | 0.459 | 0.01 | 0 | -0.02 | 0.017 | 0.055 | 0.02 |
| rs10185855 | 2 | 101642260 | Yes | Yes | G | A | 0.361 | -0.013 | 0 | 0.014 | 0.018 | -0.02 | 0.021 |
| rs1992172 | 2 | 109093836 | Yes | Yes | G | A | 0.194 | 0.02 | 0 | 0 | 0.021 | -0.037 | 0.025 |
| rs6734238 | 2 | 113841030 | Yes | No | G | A | 0.407 | -0.009 | 0 | 0.005 | 0.017 | 0.014 | 0.021 |
| rs115626828 | 2 | 118224104 | Yes | No | C | G | 0.021 | -0.041 | 0 | 0.078 | 0.065 | -0.074 | 0.077 |
| rs150474434 | 2 | 118845121 | Yes | No | A | G | 0.095 | -0.042 | 0 | 0.015 | 0.031 | 0.053 | 0.037 |
| rs17050272 | 2 | 121306440 | No | No | - | - | - | - | - | - | - | - | - |
| rs10928512 | 2 | 135451302 | Yes | No | T | G | 0.601 | -0.011 | 0 | -0.003 | 0.018 | -0.041 | 0.021 |
| rs1375131 | 2 | 135954797 | No | No | - | - | - | - | - | - | - | - | - |
| rs12614487 | 2 | 158434569 | Yes | No | T | C | 0.074 | -0.027 | 0 | -0.039 | 0.031 | -0.033 | 0.038 |
| rs10184004 | 2 | 165508389 | Yes | No | T | C | 0.413 | -0.01 | 0 | -0.006 | 0.017 | -0.008 | 0.02 |
| rs10184673 | 2 | 169827796 | Yes | No | A | G | 0.595 | -0.022 | 0 | 0.032 | 0.017 | -0.002 | 0.02 |
| rs12693968 | 2 | 203302627 | Yes | Yes | A | G | 0.258 | 0.021 | 0 | -0.011 | 0.019 | -0.009 | 0.023 |
| rs1250259 | 2 | 216300482 | Yes | No | A | T | 0.736 | 0.018 | 0 | -0.015 | 0.019 | 0.04 | 0.023 |
| rs78058190 | 2 | 219699999 | No | No | - | - | - | - | - | - | - | - | - |
| rs6431630 | 2 | 234677386 | Yes | Yes | A | G | 0.105 | 0.024 | 0 | 0.039 | 0.028 | -0.014 | 0.034 |
| rs13076933 | 3 | 12327431 | Yes | No | G | T | 0.257 | -0.025 | 0 | 0.025 | 0.019 | 0.019 | 0.023 |
| rs6792725 | 3 | 24520283 | No | No | - | - | - | - | - | - | - | - | - |
| rs9837622 | 3 | 32514647 | Yes | Yes | A | T | 0.073 | -0.037 | 0 | -0.027 | 0.036 | 0.026 | 0.042 |
| rs13621 | 3 | 52558133 | Yes | No | C | T | 0.462 | 0.011 | 0 | -0.04 | 0.017 | 0.009 | 0.02 |
| rs71311871 | 3 | 58420613 | Yes | No | G | A | 0.081 | -0.038 | 0 | 0.01 | 0.032 | 0.067 | 0.038 |
| rs55921103 | 3 | 69810294 | Yes | No | T | G | 0.641 | 0.013 | 0 | -0.024 | 0.018 | -0.019 | 0.021 |
| rs3732359 | 3 | 119536429 | Yes | No | A | G | 0.77 | -0.015 | 0 | 0.006 | 0.02 | -0.021 | 0.024 |
| rs68028452 | 3 | 122285218 | No | No | - | - | - | - | - | - | - | - | - |
| rs2011442 | 3 | 124959834 | Yes | Yes | T | C | 0.661 | 0.012 | 0 | 0.028 | 0.018 | 0.001 | 0.021 |
| rs9862203 | 3 | 126058362 | Yes | Yes | G | A | 0.76 | 0.013 | 0 | 0.024 | 0.02 | 0 | 0.023 |
| rs62264113 | 3 | 127292333 | Yes | No | A | G | 0.11 | 0.014 | 0 | -0.002 | 0.026 | 0.053 | 0.031 |
| rs56299595 | 3 | 129278182 | Yes | No | G | A | 0.125 | 0.018 | 0 | 0.026 | 0.028 | 0.005 | 0.033 |
| rs74341202 | 3 | 132183991 | Yes | No | A | G | 0.05 | -0.043 | 0 | -0.019 | 0.037 | -0.072 | 0.045 |
| rs523118 | 3 | 135965888 | Yes | No | G | T | 0.789 | 0.014 | 0 | 0.046 | 0.02 | 0.037 | 0.024 |
| rs9653945 | 3 | 142660706 | Yes | No | A | G | 0.347 | -0.013 | 0 | -0.016 | 0.018 | -0.027 | 0.021 |
| rs76440173 | 3 | 155546124 | No | No | - | - | - | - | - | - | - | - | - |
| rs6441313 | 3 | 160036400 | Yes | Yes | G | A | 0.537 | -0.013 | 0 | -0.015 | 0.017 | -0.028 | 0.02 |
| rs56118251 | 3 | 171534525 | Yes | No | G | A | 0.157 | 0.014 | 0 | 0.03 | 0.025 | 0.079 | 0.03 |
| rs397726270 | 3 | 195942080 | No | No | - | - | - | - | - | - | - | - | - |
| rs13108218 | 4 | 3443931 | Yes | No | G | A | 0.615 | -0.018 | 0 | -0.009 | 0.017 | -0.027 | 0.021 |
| rs4689653 | 4 | 7223319 | Yes | No | G | T | 0.617 | 0.011 | 0 | 0.027 | 0.017 | -0.014 | 0.021 |
| rs79623641 | 4 | 39999404 | Yes | No | A | G | 0.068 | -0.018 | 0 | -0.009 | 0.031 | -0.016 | 0.038 |
| rs112575086 | 4 | 54429890 | Yes | No | T | C | 0.12 | -0.014 | 0 | -0.002 | 0.025 | 0.004 | 0.03 |
| rs4860987 | 4 | 69491284 | No | No | - | - | - | - | - | - | - | - | - |
| rs187479321 | 4 | 70264840 | No | No | - | - | - | - | - | - | - | - | - |
| rs530082542 | 4 | 71474948 | No | No | - | - | - | - | - | - | - | - | - |
| rs938075289 | 4 | 72754788 | No | No | - | - | - | - | - | - | - | - | - |
| rs537526354 | 4 | 73491622 | No | No | - | - | - | - | - | - | - | - | - |
| rs187918276 | 4 | 74033564 | No | No | - | - | - | - | - | - | - | - | - |
| rs181372486 | 4 | 74793806 | No | No | - | - | - | - | - | - | - | - | - |
| rs189087436 | 4 | 75416194 | No | No | - | - | - | - | - | - | - | - | - |
| rs60117481 | 4 | 77420598 | No | No | - | - | - | - | - | - | - | - | - |
| rs342467 | 4 | 88052219 | Yes | No | C | T | 0.601 | 0.01 | 0 | 0.013 | 0.017 | 0.012 | 0.02 |
| rs144198753 | 4 | 99713350 | No | No | - | - | - | - | - | - | - | - | - |
| rs28497720 | 4 | 100487370 | Yes | No | T | C | 0.251 | -0.018 | 0 | 0.002 | 0.02 | -0.021 | 0.024 |
| rs13107325 | 4 | 103188709 | Yes | No | T | C | 0.064 | -0.025 | 0 | -0.037 | 0.04 | -0.011 | 0.048 |
| rs17617028 | 4 | 106064683 | Yes | No | A | G | 0.217 | 0.013 | 0 | 0.005 | 0.019 | -0.012 | 0.023 |
| rs28471982 | 4 | 110918687 | Yes | No | G | A | 0.372 | -0.009 | 0 | -0.016 | 0.018 | -0.029 | 0.021 |
| rs138204164 | 4 | 120123417 | Yes | No | G | C | 0.132 | -0.015 | 0 | 0.027 | 0.024 | 0.032 | 0.029 |
| rs2085723 | 4 | 151206057 | Yes | No | T | G | 0.649 | 0.01 | 0 | -0.032 | 0.018 | -0.025 | 0.021 |
| rs41280463 | 4 | 154191226 | Yes | No | A | G | 0.167 | -0.015 | 0 | 0.019 | 0.021 | -0.026 | 0.025 |
| rs6054 | 4 | 155489608 | No | No | - | - | - | - | - | - | - | - | - |
| rs72701754 | 4 | 185251995 | Yes | No | T | A | 0.404 | 0.009 | 0 | 0.01 | 0.017 | 0.004 | 0.021 |
| rs116734477 | 5 | 52095024 | Yes | No | T | C | 0.038 | -0.051 | 0 | 0.071 | 0.042 | -0.015 | 0.05 |
| rs13173241 | 5 | 55861359 | Yes | No | A | G | 0.195 | 0.016 | 0 | 0.028 | 0.023 | -0.031 | 0.027 |
| rs3010275 | 5 | 72014761 | Yes | No | G | T | 0.21 | -0.018 | 0 | 0.062 | 0.02 | 0.018 | 0.025 |
| rs12916 | 5 | 74656539 | Yes | Yes | C | T | 0.402 | 0.07 | 0 | -0.003 | 0.017 | -0.007 | 0.02 |
| rs139733342 | 5 | 75241150 | No | No | - | - | - | - | - | - | - | - | - |
| rs6869845 | 5 | 122833703 | Yes | No | T | C | 0.549 | 0.017 | 0 | -0.01 | 0.017 | 0.016 | 0.02 |
| rs11745587 | 5 | 131796922 | Yes | Yes | A | G | 0.355 | 0.016 | 0 | -0.007 | 0.017 | -0.009 | 0.021 |
| rs10037298 | 5 | 139567696 | Yes | No | G | T | 0.213 | -0.013 | 0 | 0.013 | 0.02 | -0.022 | 0.024 |
| rs11167778 | 5 | 141888881 | Yes | Yes | T | C | 0.107 | 0.022 | 0 | -0.001 | 0.028 | 0.032 | 0.033 |
| rs17053386 | 5 | 155709100 | Yes | No | A | G | 0.023 | 0.033 | 0 | 0.01 | 0.051 | -0.057 | 0.061 |
| rs12657266 | 5 | 156396003 | Yes | No | T | C | 0.635 | 0.035 | 0 | -0.017 | 0.018 | 0.011 | 0.021 |
| rs352942 | 5 | 176528386 | Yes | No | A | G | 0.252 | -0.01 | 0 | 0.012 | 0.02 | 0.005 | 0.023 |
| rs59408219 | 6 | 11850754 | No | No | - | - | - | - | - | - | - | - | - |
| rs2235215 | 6 | 16131156 | Yes | No | C | T | 0.321 | -0.032 | 0 | 0.018 | 0.017 | -0.015 | 0.021 |
| rs6940342 | 6 | 21381568 | Yes | Yes | C | T | 0.625 | 0.009 | 0 | 0.004 | 0.017 | -0.027 | 0.021 |
| rs113760175 | 6 | 22343592 | Yes | No | A | G | 0.065 | -0.02 | 0 | 0.004 | 0.037 | -0.093 | 0.045 |
| rs116272812 | 6 | 25452783 | Yes | No | C | T | 0.121 | -0.023 | 0 | -0.008 | 0.026 | 0.003 | 0.031 |
| rs1800562 | 6 | 26093141 | Yes | No | A | G | 0.068 | -0.057 | 0 | -0.013 | 0.033 | -0.05 | 0.04 |
| rs35657082 | 6 | 27067657 | No | No | - | - | - | - | - | - | - | - | - |
| rs3118362 | 6 | 28785085 | No | No | - | - | - | - | - | - | - | - | - |
| rs2517671 | 6 | 29937977 | Yes | No | G | A | 0.407 | 0.015 | 0 | 0.033 | 0.017 | -0.02 | 0.021 |
| rs1265097 | 6 | 31106459 | Yes | No | A | C | 0.095 | 0.029 | 0 | 0.003 | 0.03 | -0.012 | 0.037 |
| rs7774197 | 6 | 32046275 | Yes | Yes | C | A | 0.064 | 0.035 | 0 | -0.017 | 0.035 | -0.028 | 0.042 |
| rs6689 | 6 | 32627700 | Yes | No | G | A | 0.203 | 0.039 | 0 | -0.015 | 0.021 | 0.01 | 0.026 |
| rs3800461 | 6 | 34616322 | Yes | No | C | G | 0.119 | -0.024 | 0 | -0.032 | 0.026 | -0.008 | 0.03 |
| rs11754612 | 6 | 35476562 | Yes | No | A | T | 0.115 | -0.015 | 0 | -0.009 | 0.026 | 0.027 | 0.031 |
| rs11380614 | 6 | 37037818 | No | No | - | - | - | - | - | - | - | - | - |
| rs11759627 | 6 | 39238772 | Yes | No | T | C | 0.32 | 0.01 | 0 | 0.006 | 0.018 | -0.006 | 0.022 |
| rs2395943 | 6 | 42940673 | Yes | No | G | A | 0.586 | -0.013 | 0 | 0.02 | 0.017 | -0.024 | 0.021 |
| rs9472125 | 6 | 43756169 | No | No | - | - | - | - | - | - | - | - | - |
| rs17665178 | 6 | 52435243 | Yes | No | G | C | 0.302 | -0.016 | 0 | 0.016 | 0.018 | 0.023 | 0.021 |
| rs12662589 | 6 | 53509035 | Yes | Yes | C | G | 0.259 | 0.015 | 0 | 0.01 | 0.019 | 0.032 | 0.023 |
| rs9496567 | 6 | 100602753 | Yes | Yes | A | G | 0.236 | -0.021 | 0 | -0.038 | 0.02 | -0.002 | 0.024 |
| rs62419249 | 6 | 101447338 | Yes | No | A | G | 0.478 | 0.012 | 0 | -0.01 | 0.017 | 0.003 | 0.02 |
| rs4946713 | 6 | 106374015 | Yes | Yes | A | C | 0.447 | -0.011 | 0 | -0.018 | 0.017 | -0.027 | 0.02 |
| rs11153143 | 6 | 109300661 | Yes | No | T | C | 0.126 | -0.014 | 0 | -0.044 | 0.026 | -0.027 | 0.031 |
| rs1556857 | 6 | 116325559 | Yes | Yes | C | T | 0.407 | -0.017 | 0 | 0.018 | 0.018 | 0.022 | 0.021 |
| rs9388498 | 6 | 126873423 | No | No | - | - | - | - | - | - | - | - | - |
| rs67822704 | 6 | 130386212 | No | No | - | - | - | - | - | - | - | - | - |
| rs9399137 | 6 | 135419018 | Yes | No | C | T | 0.262 | -0.026 | 0 | -0.026 | 0.019 | 0.002 | 0.022 |
| rs72974722 | 6 | 139331419 | Yes | No | C | A | 0.177 | 0.021 | 0 | -0.011 | 0.021 | 0.023 | 0.026 |
| rs635769 | 6 | 139831981 | Yes | No | C | T | 0.619 | -0.01 | 0 | -0.027 | 0.017 | 0.012 | 0.021 |
| rs9480534 | 6 | 151015562 | Yes | No | G | A | 0.092 | -0.017 | 0 | -0.009 | 0.03 | 0.041 | 0.035 |
| rs1871859 | 6 | 151898506 | Yes | Yes | T | C | 0.13 | 0.018 | 0 | -0.028 | 0.024 | -0.027 | 0.029 |
| rs9347330 | 6 | 159984784 | Yes | No | A | T | 0.337 | 0.01 | 0 | -0.013 | 0.018 | -0.018 | 0.021 |
| rs2297359 | 6 | 160492613 | Yes | No | C | T | 0.015 | 0.118 | 0 | -0.1 | 0.075 | -0.021 | 0.091 |
| rs10455872 | 6 | 161010118 | Yes | Yes | G | A | 0.069 | 0.114 | 0 | 0.005 | 0.032 | -0.006 | 0.039 |
| rs148349043 | 6 | 161659369 | Yes | No | G | C | 0.014 | 0.085 | 0 | 0 | 0.075 | -0.032 | 0.089 |
| rs10272002 | 7 | 1047615 | Yes | No | G | A | 0.21 | -0.022 | 0 | 0.006 | 0.02 | -0.005 | 0.024 |
| rs144787122 | 7 | 2296552 | No | No | - | - | - | - | - | - | - | - | - |
| rs55696093 | 7 | 21605973 | Yes | No | G | A | 0.211 | 0.038 | 0 | 0.02 | 0.02 | -0.016 | 0.024 |
| rs4722551 | 7 | 25991826 | Yes | Yes | C | T | 0.163 | 0.036 | 0 | 0.02 | 0.023 | 0.056 | 0.027 |
| rs12533280 | 7 | 36171953 | Yes | Yes | T | C | 0.195 | 0.018 | 0 | 0.004 | 0.021 | -0.01 | 0.025 |
| rs7808613 | 7 | 41746922 | Yes | Yes | G | C | 0.245 | 0.011 | 0 | -0.025 | 0.02 | 0.021 | 0.023 |
| rs28658444 | 7 | 44006213 | Yes | No | C | G | 0.014 | 0.039 | 0 | 0.043 | 0.069 | 0.062 | 0.083 |
| rs17725246 | 7 | 44581986 | Yes | Yes | C | T | 0.195 | 0.043 | 0 | 0.009 | 0.021 | 0.023 | 0.025 |
| rs799157 | 7 | 73020301 | Yes | No | C | T | 0.964 | -0.032 | 0 | -0.028 | 0.049 | 0.031 | 0.057 |
| rs2302434 | 7 | 75630183 | Yes | Yes | T | C | 0.174 | 0.015 | 0 | 0.013 | 0.022 | -0.005 | 0.026 |
| rs1014283 | 7 | 87076587 | Yes | Yes | A | C | 0.183 | -0.014 | 0 | -0.007 | 0.021 | -0.005 | 0.025 |
| rs6967728 | 7 | 97915637 | Yes | No | G | A | 0.817 | 0.018 | 0 | 0.003 | 0.021 | -0.032 | 0.025 |
| rs564449 | 7 | 100321138 | Yes | No | G | T | 0.885 | -0.027 | 0 | -0.004 | 0.024 | 0.019 | 0.029 |
| rs10248717 | 7 | 107056795 | Yes | No | G | A | 0.309 | 0.013 | 0 | -0.024 | 0.019 | 0.002 | 0.022 |
| rs62621812 | 7 | 127015083 | Yes | No | A | G | 0.024 | 0.03 | 0 | 0.03 | 0.051 | 0.066 | 0.062 |
| rs1838931 | 7 | 130455588 | Yes | No | T | C | 0.312 | 0.011 | 0 | 0.001 | 0.019 | -0.028 | 0.022 |
| rs4374942 | 7 | 155026807 | Yes | Yes | C | T | 0.081 | 0.02 | 0 | 0.006 | 0.032 | -0.002 | 0.038 |
| rs2928576 | 8 | 6600349 | Yes | No | C | T | 0.672 | 0.012 | 0 | -0.007 | 0.018 | -0.03 | 0.022 |
| rs13256216 | 8 | 8577883 | Yes | No | T | C | 0.25 | 0.013 | 0 | -0.022 | 0.019 | -0.06 | 0.023 |
| rs9987289 | 8 | 9183358 | Yes | No | G | A | 0.908 | 0.06 | 0 | -0.005 | 0.027 | 0.043 | 0.033 |
| rs4645567 | 8 | 9723964 | Yes | No | A | T | 0.786 | -0.014 | 0 | -0.008 | 0.02 | 0.023 | 0.024 |
| rs11250076 | 8 | 10647823 | Yes | No | G | A | 0.571 | -0.01 | 0 | 0.015 | 0.017 | 0.027 | 0.02 |
| rs9692668 | 8 | 11789933 | Yes | No | A | C | 0.556 | 0.01 | 0 | 0.006 | 0.017 | -0.049 | 0.02 |
| rs1495741 | 8 | 18272881 | Yes | No | A | G | 0.779 | -0.022 | 0 | 0.025 | 0.02 | -0.001 | 0.024 |
| rs769111033 | 8 | 19855858 | No | No | - | - | - | - | - | - | - | - | - |
| rs900776 | 8 | 21918089 | Yes | No | C | A | 0.17 | -0.02 | 0 | 0.006 | 0.023 | 0.019 | 0.027 |
| rs117139027 | 8 | 29024943 | No | No | - | - | - | - | - | - | - | - | - |
| rs9298506 | 8 | 55437524 | Yes | Yes | G | A | 0.208 | 0.025 | 0 | 0.012 | 0.02 | 0.004 | 0.024 |
| rs9297994 | 8 | 59392324 | Yes | Yes | A | G | 0.662 | -0.036 | 0 | 0 | 0.018 | 0.014 | 0.022 |
| rs12114596 | 8 | 61518399 | No | No | - | - | - | - | - | - | - | - | - |
| rs62509311 | 8 | 74907295 | No | No | - | - | - | - | - | - | - | - | - |
| rs400824 | 8 | 81357702 | Yes | No | T | C | 0.29 | 0.01 | 0 | -0.009 | 0.019 | 0.007 | 0.023 |
| rs2737245 | 8 | 116658583 | Yes | Yes | T | G | 0.277 | -0.025 | 0 | 0.028 | 0.018 | 0.027 | 0.022 |
| rs28601761 | 8 | 126500031 | No | No | - | - | - | - | - | - | - | - | - |
| rs11787335 | 8 | 145044104 | Yes | No | T | C | 0.355 | 0.023 | 0 | -0.029 | 0.018 | -0.007 | 0.022 |
| rs3780181 | 9 | 2640759 | Yes | Yes | G | A | 0.07 | -0.036 | 0 | 0 | 0.035 | -0.024 | 0.042 |
| rs28498684 | 9 | 16900695 | Yes | No | G | A | 0.6 | -0.013 | 0 | -0.033 | 0.017 | -0.018 | 0.02 |
| rs12551960 | 9 | 19267440 | Yes | No | T | C | 0.079 | 0.035 | 0 | 0.011 | 0.029 | -0.05 | 0.036 |
| rs10757273 | 9 | 22090301 | No | No | - | - | - | - | - | - | - | - | - |
| rs7864568 | 9 | 78212428 | Yes | Yes | G | A | 0.684 | 0.016 | 0 | -0.014 | 0.018 | -0.011 | 0.022 |
| rs1571791 | 9 | 78729213 | Yes | No | C | T | 0.62 | -0.014 | 0 | -0.008 | 0.018 | 0.005 | 0.021 |
| rs142550358 | 9 | 91392686 | No | No | - | - | - | - | - | - | - | - | - |
| rs2740488 | 9 | 107661742 | Yes | No | C | A | 0.261 | -0.025 | 0 | -0.001 | 0.019 | 0.045 | 0.023 |
| rs56294298 | 9 | 117133524 | Yes | No | A | G | 0.085 | -0.018 | 0 | -0.034 | 0.033 | 0.053 | 0.039 |
| rs13289095 | 9 | 131466489 | No | No | - | - | - | - | - | - | - | - | - |
| rs2519093 | 9 | 136141870 | Yes | No | T | C | 0.188 | 0.072 | 0 | 0.022 | 0.02 | 0.01 | 0.024 |
| rs13301660 | 9 | 139340802 | Yes | No | T | C | 0.27 | -0.016 | 0 | -0.011 | 0.019 | 0.008 | 0.023 |
| rs7903259 | 10 | 17259642 | Yes | No | G | C | 0.416 | 0.016 | 0 | -0.004 | 0.017 | 0.001 | 0.021 |
| rs41274050 | 10 | 52573772 | No | No | - | - | - | - | - | - | - | - | - |
| rs10761750 | 10 | 65128619 | Yes | No | A | G | 0.483 | 0.014 | 0 | -0.057 | 0.017 | 0.011 | 0.02 |
| rs17476364 | 10 | 71094504 | Yes | No | C | T | 0.099 | -0.027 | 0 | -0.018 | 0.027 | 0.013 | 0.032 |
| rs11000443 | 10 | 74665640 | Yes | No | A | C | 0.043 | -0.021 | 0 | -0.022 | 0.042 | -0.008 | 0.049 |
| rs1870140 | 10 | 82246749 | Yes | No | G | A | 0.843 | 0.012 | 0 | -0.01 | 0.023 | -0.008 | 0.027 |
| rs477418 | 10 | 89805410 | Yes | No | A | C | 0.033 | -0.025 | 0 | -0.02 | 0.056 | -0.025 | 0.067 |
| rs2068888 | 10 | 94839642 | Yes | No | A | G | 0.451 | -0.018 | 0 | -0.003 | 0.017 | -0.001 | 0.02 |
| rs61886346 | 10 | 96101364 | Yes | No | T | C | 0.061 | -0.019 | 0 | -0.025 | 0.035 | -0.028 | 0.041 |
| rs603424 | 10 | 102075479 | Yes | No | A | G | 0.169 | 0.016 | 0 | 0.017 | 0.025 | 0.009 | 0.03 |
| rs2792751 | 10 | 113940329 | Yes | No | C | T | 0.719 | -0.024 | 0 | 0.027 | 0.019 | 0.005 | 0.023 |
| rs7079542 | 10 | 115794898 | Yes | No | G | A | 0.12 | -0.016 | 0 | 0.014 | 0.026 | 0.023 | 0.031 |
| rs2301179 | 10 | 118404620 | Yes | Yes | G | A | 0.508 | 0.016 | 0 | -0.019 | 0.017 | -0.02 | 0.02 |
| rs7904973 | 10 | 124693587 | Yes | No | T | G | 0.576 | 0.021 | 0 | 0.014 | 0.017 | 0.017 | 0.02 |
| rs151191319 | 11 | 1081287 | Yes | No | A | G | 0.033 | -0.031 | 0 | -0.072 | 0.046 | 0.001 | 0.055 |
| rs7124487 | 11 | 2988323 | Yes | No | T | C | 0.186 | -0.012 | 0 | 0.019 | 0.023 | -0.007 | 0.027 |
| rs188513096 | 11 | 4993424 | No | No | - | - | - | - | - | - | - | - | - |
| rs11601507 | 11 | 5701074 | Yes | No | A | C | 0.07 | 0.04 | 0 | -0.017 | 0.032 | -0.007 | 0.037 |
| rs10832956 | 11 | 18645668 | Yes | Yes | C | T | 0.728 | 0.021 | 0 | 0.033 | 0.019 | 0.019 | 0.022 |
| rs61882680 | 11 | 46370636 | Yes | No | T | C | 0.03 | -0.028 | 0 | -0.081 | 0.044 | 0.002 | 0.053 |
| rs174547 | 11 | 61570783 | Yes | No | C | T | 0.346 | -0.043 | 0 | 0.001 | 0.018 | 0.009 | 0.021 |
| rs7950543 | 11 | 63599440 | Yes | No | C | T | 0.37 | 0.011 | 0 | 0.001 | 0.017 | -0.005 | 0.021 |
| rs642803 | 11 | 65560620 | Yes | No | T | C | 0.456 | 0.01 | 0 | 0.015 | 0.017 | -0.015 | 0.02 |
| rs117777720 | 11 | 66196384 | Yes | No | T | C | 0.241 | -0.015 | 0 | 0.024 | 0.019 | -0.02 | 0.023 |
| rs78643851 | 11 | 69706362 | No | No | - | - | - | - | - | - | - | - | - |
| rs11237488 | 11 | 78128775 | Yes | No | T | C | 0.128 | -0.013 | 0 | 0.017 | 0.026 | 0.053 | 0.031 |
| rs11226108 | 11 | 103870755 | Yes | No | C | G | 0.191 | -0.014 | 0 | 0.022 | 0.021 | 0.016 | 0.025 |
| rs964184 | 11 | 116648917 | Yes | No | C | G | 0.865 | -0.055 | 0 | 0.033 | 0.025 | 0.003 | 0.03 |
| rs116987336 | 11 | 117175658 | Yes | No | A | G | 0.027 | 0.036 | 0 | -0.092 | 0.053 | -0.091 | 0.064 |
| rs4639966 | 11 | 118573519 | Yes | Yes | C | T | 0.241 | 0.015 | 0 | -0.015 | 0.02 | -0.021 | 0.024 |
| rs3862606 | 11 | 121330087 | Yes | No | G | A | 0.448 | 0.01 | 0 | 0.014 | 0.017 | -0.008 | 0.02 |
| rs10790519 | 11 | 122535408 | Yes | No | C | T | 0.385 | 0.016 | 0 | 0.003 | 0.017 | -0.012 | 0.021 |
| rs112771035 | 11 | 126225876 | Yes | No | G | C | 0.069 | 0.063 | 0 | -0.023 | 0.031 | 0.055 | 0.037 |
| rs35882350 | 12 | 623129 | No | No | - | - | - | - | - | - | - | - | - |
| rs76895963 | 12 | 4384844 | No | No | - | - | - | - | - | - | - | - | - |
| rs145920606 | 12 | 7627311 | Yes | No | G | A | 0.081 | -0.017 | 0 | 0.037 | 0.03 | -0.045 | 0.036 |
| rs149871778 | 12 | 9098995 | No | No | - | - | - | - | - | - | - | - | - |
| rs75667995 | 12 | 25409070 | Yes | No | C | T | 0.068 | -0.029 | 0 | -0.024 | 0.035 | -0.068 | 0.041 |
| rs11175540 | 12 | 40586295 | Yes | No | A | T | 0.066 | 0.026 | 0 | 0.017 | 0.033 | 0.008 | 0.039 |
| rs2250751 | 12 | 51106178 | Yes | No | A | G | 0.341 | -0.017 | 0 | 0.048 | 0.018 | 0.017 | 0.021 |
| rs61177162 | 12 | 51779544 | No | No | - | - | - | - | - | - | - | - | - |
| rs74090765 | 12 | 53792914 | Yes | No | G | T | 0.173 | 0.014 | 0 | -0.024 | 0.021 | -0.056 | 0.025 |
| rs11172134 | 12 | 57645789 | Yes | No | A | T | 0.208 | -0.015 | 0 | 0.025 | 0.02 | 0.026 | 0.023 |
| rs61754230 | 12 | 72179446 | No | No | - | - | - | - | - | - | - | - | - |
| rs12306780 | 12 | 89921860 | Yes | No | T | A | 0.342 | 0.012 | 0 | -0.029 | 0.018 | -0.018 | 0.021 |
| rs1515565 | 12 | 92818786 | Yes | No | G | A | 0.518 | 0.009 | 0 | 0.004 | 0.017 | -0.011 | 0.02 |
| rs11837065 | 12 | 100859983 | Yes | Yes | T | C | 0.366 | -0.011 | 0 | -0.015 | 0.018 | 0.02 | 0.021 |
| rs978458 | 12 | 102802239 | Yes | No | C | T | 0.737 | -0.012 | 0 | -0.012 | 0.019 | 0.016 | 0.023 |
| rs1196760 | 12 | 105606068 | Yes | Yes | C | G | 0.908 | -0.02 | 0 | -0.031 | 0.029 | -0.008 | 0.034 |
| rs11114055 | 12 | 109169137 | Yes | No | C | T | 0.311 | 0.013 | 0 | -0.011 | 0.018 | -0.029 | 0.022 |
| rs4766519 | 12 | 111370980 | Yes | No | T | C | 0.521 | 0.011 | 0 | -0.002 | 0.017 | 0.031 | 0.02 |
| rs3184504 | 12 | 111884608 | Yes | No | C | T | 0.527 | 0.023 | 0 | 0.006 | 0.017 | 0.028 | 0.02 |
| rs11066412 | 12 | 113218868 | Yes | No | A | G | 0.27 | -0.014 | 0 | -0.006 | 0.02 | -0.055 | 0.023 |
| rs2516079 | 12 | 120859188 | Yes | No | G | T | 0.287 | 0.01 | 0 | 0.041 | 0.019 | 0.005 | 0.022 |
| rs1169288 | 12 | 121416650 | Yes | Yes | C | A | 0.322 | 0.036 | 0 | 0.015 | 0.018 | 0.023 | 0.022 |
| rs2247139 | 12 | 122380274 | Yes | No | G | A | 0.868 | -0.013 | 0 | -0.064 | 0.024 | 0.032 | 0.029 |
| rs28461471 | 12 | 123856974 | No | No | - | - | - | - | - | - | - | - | - |
| rs2451322 | 12 | 124545515 | Yes | No | A | G | 0.595 | 0.011 | 0 | 0.028 | 0.017 | 0.009 | 0.021 |
| rs11057840 | 12 | 125316055 | Yes | No | C | A | 0.138 | 0.022 | 0 | 0.014 | 0.025 | -0.036 | 0.03 |
| rs75588192 | 12 | 133048600 | Yes | No | A | G | 0.14 | 0.016 | 0 | 0.003 | 0.024 | 0.014 | 0.029 |
| rs368213791 | 13 | 32976656 | No | No | - | - | - | - | - | - | - | - | - |
| rs17532371 | 13 | 41635301 | Yes | No | G | C | 0.071 | -0.019 | 0 | -0.023 | 0.034 | 0.103 | 0.04 |
| rs9592980 | 13 | 74855425 | Yes | Yes | A | G | 0.585 | 0.01 | 0 | -0.023 | 0.017 | 0.022 | 0.021 |
| rs7330899 | 13 | 95223400 | Yes | Yes | A | G | 0.244 | 0.014 | 0 | 0.001 | 0.02 | 0.059 | 0.024 |
| rs4771674 | 13 | 111039070 | Yes | No | G | A | 0.619 | 0.014 | 0 | 0.006 | 0.017 | -0.01 | 0.021 |
| rs6602909 | 13 | 114551993 | Yes | No | C | T | 0.33 | 0.021 | 0 | -0.003 | 0.018 | 0.005 | 0.022 |
| rs11621792 | 14 | 24871926 | Yes | No | T | C | 0.449 | 0.022 | 0 | 0.028 | 0.017 | -0.007 | 0.02 |
| rs139262716 | 14 | 31725111 | Yes | No | A | G | 0.019 | -0.039 | 0 | -0.026 | 0.066 | -0.006 | 0.079 |
| rs11846704 | 14 | 35186694 | Yes | No | T | C | 0.267 | -0.014 | 0 | 0.022 | 0.019 | -0.018 | 0.023 |
| rs12897637 | 14 | 64239351 | Yes | No | C | T | 0.16 | 0.016 | 0 | -0.013 | 0.023 | 0.016 | 0.027 |
| rs7157399 | 14 | 70846954 | Yes | Yes | C | T | 0.86 | 0.025 | 0 | -0.009 | 0.024 | 0.05 | 0.028 |
| rs13379043 | 14 | 74250126 | Yes | No | C | T | 0.271 | -0.017 | 0 | 0.035 | 0.019 | 0.013 | 0.023 |
| rs2058919 | 14 | 75310812 | Yes | Yes | C | G | 0.61 | -0.01 | 0 | -0.004 | 0.017 | 0.022 | 0.02 |
| rs35802157 | 14 | 90066634 | Yes | No | C | T | 0.627 | -0.01 | 0 | 0.007 | 0.017 | -0.009 | 0.021 |
| rs17580 | 14 | 94847262 | Yes | Yes | A | T | 0.039 | 0.05 | 0 | 0.051 | 0.05 | -0.039 | 0.059 |
| rs2413926 | 15 | 49336477 | Yes | Yes | A | T | 0.375 | -0.013 | 0 | -0.001 | 0.018 | 0.008 | 0.021 |
| rs62023490 | 15 | 53079024 | Yes | No | A | G | 0.105 | -0.015 | 0 | 0 | 0.027 | -0.045 | 0.031 |
| rs79391862 | 15 | 53739426 | Yes | No | C | A | 0.02 | -0.064 | 0 | 0.063 | 0.044 | 0.056 | 0.052 |
| rs72749499 | 15 | 57295579 | Yes | No | G | A | 0.07 | 0.029 | 0 | 0.036 | 0.033 | 0.081 | 0.04 |
| rs35609148 | 15 | 58681533 | No | No | - | - | - | - | - | - | - | - | - |
| rs11636087 | 15 | 63788267 | Yes | No | C | T | 0.298 | 0.014 | 0 | 0.019 | 0.019 | -0.014 | 0.023 |
| rs12917376 | 15 | 75116167 | Yes | No | T | C | 0.577 | 0.013 | 0 | 0.006 | 0.017 | 0.038 | 0.02 |
| rs34631529 | 15 | 101845482 | No | No | - | - | - | - | - | - | - | - | - |
| rs12445804 | 16 | 11706100 | Yes | Yes | A | G | 0.076 | 0.033 | 0 | -0.017 | 0.033 | -0.023 | 0.039 |
| rs35468353 | 16 | 31056433 | Yes | No | G | A | 0.377 | 0.012 | 0 | 0 | 0.017 | -0.025 | 0.021 |
| rs247617 | 16 | 56990716 | Yes | No | A | C | 0.321 | -0.037 | 0 | 0.019 | 0.018 | -0.01 | 0.021 |
| rs181501802 | 16 | 70067440 | Yes | No | A | G | 0.06 | 0.024 | 0 | 0.035 | 0.032 | 0.062 | 0.038 |
| rs56212732 | 16 | 70930370 | No | No | - | - | - | - | - | - | - | - | - |
| rs34042070 | 16 | 72101525 | Yes | No | G | C | 0.191 | 0.057 | 0 | 0.034 | 0.021 | 0.016 | 0.025 |
| rs7404072 | 16 | 73096750 | No | No | - | - | - | - | - | - | - | - | - |
| rs1121985 | 16 | 79363079 | Yes | Yes | C | A | 0.63 | 0.011 | 0 | -0.013 | 0.018 | 0.003 | 0.021 |
| rs67890964 | 16 | 83979317 | No | No | - | - | - | - | - | - | - | - | - |
| rs74035509 | 16 | 88567333 | Yes | No | T | C | 0.074 | 0.023 | 0 | -0.002 | 0.034 | 0.015 | 0.041 |
| rs34460487 | 17 | 4685228 | No | No | - | - | - | - | - | - | - | - | - |
| rs571475342 | 17 | 5207882 | No | No | - | - | - | - | - | - | - | - | - |
| rs186021206 | 17 | 7069412 | No | No | - | - | - | - | - | - | - | - | - |
| rs9894946 | 17 | 7571080 | Yes | No | G | A | 0.841 | -0.017 | 0 | -0.018 | 0.023 | -0.016 | 0.028 |
| rs2270445 | 17 | 8219478 | Yes | Yes | G | A | 0.483 | 0.014 | 0 | 0.023 | 0.017 | -0.019 | 0.02 |
| rs28811342 | 17 | 18125845 | Yes | No | C | T | 0.197 | 0.013 | 0 | 0.032 | 0.02 | -0.006 | 0.025 |
| rs704 | 17 | 26694861 | Yes | Yes | A | G | 0.477 | 0.019 | 0 | 0.028 | 0.017 | 0.006 | 0.02 |
| rs56336338 | 17 | 27645258 | Yes | No | A | G | 0.134 | -0.019 | 0 | 0.016 | 0.025 | 0.039 | 0.029 |
| rs111919672 | 17 | 28583498 | Yes | No | G | C | 0.073 | -0.02 | 0 | 0.055 | 0.029 | -0.006 | 0.034 |
| rs12945088 | 17 | 29397852 | Yes | No | A | G | 0.693 | 0.018 | 0 | -0.005 | 0.018 | 0 | 0.021 |
| rs640306 | 17 | 37067261 | Yes | No | C | T | 0.656 | -0.01 | 0 | -0.001 | 0.018 | -0.013 | 0.021 |
| rs12943633 | 17 | 37970365 | Yes | Yes | T | C | 0.081 | -0.018 | 0 | 0.006 | 0.031 | -0.009 | 0.037 |
| rs2354155 | 17 | 40546652 | Yes | No | A | G | 0.527 | -0.01 | 0 | 0.004 | 0.017 | 0.012 | 0.02 |
| rs72836561 | 17 | 41926126 | Yes | No | T | C | 0.03 | -0.03 | 0 | 0.025 | 0.049 | 0.034 | 0.058 |
| rs12603290 | 17 | 45650196 | Yes | No | C | T | 0.505 | -0.027 | 0 | 0.015 | 0.017 | -0.003 | 0.02 |
| rs2288278 | 17 | 46661292 | Yes | Yes | A | G | 0.65 | 0.014 | 0 | 0.009 | 0.017 | 0.04 | 0.021 |
| rs1292061 | 17 | 57911230 | Yes | No | G | A | 0.549 | 0.01 | 0 | -0.011 | 0.017 | -0.01 | 0.02 |
| rs1801689 | 17 | 64210580 | Yes | No | C | A | 0.026 | 0.091 | 0 | 0.021 | 0.058 | 0.105 | 0.068 |
| rs9332408 | 17 | 65108987 | Yes | Yes | C | T | 0.472 | 0.012 | 0 | 0.011 | 0.017 | -0.008 | 0.02 |
| rs78186330 | 17 | 66004715 | Yes | No | A | G | 0.208 | 0.016 | 0 | 0.024 | 0.021 | 0.002 | 0.024 |
| rs77542162 | 17 | 67081278 | Yes | No | G | A | 0.02 | 0.183 | 0 | -0.001 | 0.062 | -0.015 | 0.073 |
| rs75910690 | 17 | 67668868 | Yes | No | T | C | 0.027 | 0.053 | 0 | -0.004 | 0.053 | -0.014 | 0.064 |
| rs9890133 | 17 | 68169005 | Yes | No | G | A | 0.119 | 0.016 | 0 | 0.029 | 0.025 | -0.02 | 0.03 |
| rs4485425 | 17 | 73767437 | Yes | Yes | G | A | 0.714 | 0.018 | 0 | 0.004 | 0.019 | 0.023 | 0.023 |
| rs11657987 | 17 | 76387363 | Yes | No | T | G | 0.498 | 0.018 | 0 | 0.001 | 0.017 | 0.027 | 0.02 |
| rs12950377 | 17 | 81011652 | Yes | No | C | T | 0.151 | 0.013 | 0 | 0.024 | 0.025 | -0.013 | 0.03 |
| rs150513143 | 18 | 3451539 | No | No | - | - | - | - | - | - | - | - | - |
| rs2840354 | 18 | 9545961 | Yes | Yes | T | C | 0.191 | 0.012 | 0 | -0.029 | 0.022 | -0.035 | 0.026 |
| rs538969346 | 18 | 46545976 | No | No | - | - | - | - | - | - | - | - | - |
| rs77960347 | 18 | 47109955 | Yes | No | G | A | 0.013 | 0.07 | 0 | -0.05 | 0.07 | -0.004 | 0.081 |
| rs12968116 | 18 | 55322502 | Yes | Yes | T | C | 0.126 | 0.014 | 0 | 0.005 | 0.024 | -0.032 | 0.028 |
| rs402348 | 18 | 61034420 | No | No | - | - | - | - | - | - | - | - | - |
| rs4807570 | 19 | 1123652 | Yes | No | A | G | 0.21 | -0.012 | 0 | 0.031 | 0.021 | 0.003 | 0.025 |
| rs1736177 | 19 | 2791624 | Yes | No | T | C | 0.307 | 0.016 | 0 | -0.005 | 0.018 | -0.05 | 0.022 |
| rs34054295 | 19 | 4478062 | Yes | No | G | A | 0.617 | -0.012 | 0 | 0.008 | 0.017 | -0.052 | 0.021 |
| rs708686 | 19 | 5840619 | Yes | No | T | C | 0.278 | 0.011 | 0 | -0.024 | 0.018 | 0.015 | 0.022 |
| rs571497 | 19 | 7827830 | Yes | Yes | A | G | 0.153 | -0.015 | 0 | 0.031 | 0.023 | 0.022 | 0.028 |
| rs1042540024 | 19 | 9901805 | No | No | - | - | - | - | - | - | - | - | - |
| rs12720359 | 19 | 10464138 | Yes | No | G | C | 0.033 | -0.057 | 0 | -0.045 | 0.053 | 0.062 | 0.064 |
| rs73015024 | 19 | 11197598 | Yes | No | T | G | 0.112 | -0.215 | 0 | 0.005 | 0.028 | 0.032 | 0.034 |
| rs553321524 | 19 | 11723655 | No | No | - | - | - | - | - | - | - | - | - |
| rs753931089 | 19 | 12295949 | No | No | - | - | - | - | - | - | - | - | - |
| rs560788989 | 19 | 13250023 | No | No | - | - | - | - | - | - | - | - | - |
| rs36014207 | 19 | 14172728 | Yes | No | T | C | 0.407 | -0.011 | 0 | 0.029 | 0.017 | 0.026 | 0.021 |
| rs660247 | 19 | 15796507 | Yes | Yes | G | A | 0.531 | 0.01 | 0 | -0.021 | 0.017 | 0.014 | 0.02 |
| rs11668883 | 19 | 17527370 | Yes | No | A | G | 0.811 | -0.013 | 0 | -0.022 | 0.021 | -0.009 | 0.025 |
| rs4808766 | 19 | 18335715 | Yes | No | C | G | 0.258 | 0.014 | 0 | 0.015 | 0.02 | 0.003 | 0.024 |
| rs58542926 | 19 | 19379549 | Yes | No | T | C | 0.075 | -0.111 | 0 | 0.054 | 0.029 | 0.049 | 0.034 |
| rs75916629 | 19 | 20213781 | Yes | No | G | A | 0.025 | -0.058 | 0 | 0.003 | 0.043 | 0.008 | 0.051 |
| rs187551406 | 19 | 20737538 | Yes | No | T | C | 0.024 | -0.037 | 0 | 0.009 | 0.048 | 0.096 | 0.057 |
| rs147791730 | 19 | 33864260 | Yes | No | A | G | 0.028 | -0.029 | 0 | 0.031 | 0.05 | 0.012 | 0.061 |
| rs28659908 | 19 | 35693120 | Yes | No | C | T | 0.402 | -0.01 | 0 | 0.007 | 0.017 | -0.028 | 0.021 |
| rs4803525 | 19 | 38319269 | Yes | No | G | A | 0.581 | -0.009 | 0 | 0.019 | 0.017 | -0.013 | 0.02 |
| rs56113850 | 19 | 41353107 | No | No | - | - | - | - | - | - | - | - | - |
| rs768815028 | 19 | 41921372 | No | No | - | - | - | - | - | - | - | - | - |
| rs562494568 | 19 | 42649075 | No | No | - | - | - | - | - | - | - | - | - |
| rs538657595 | 19 | 43234281 | No | No | - | - | - | - | - | - | - | - | - |
| rs76989105 | 19 | 44388711 | Yes | No | C | G | 0.013 | -0.076 | 0 | -0.181 | 0.055 | -0.035 | 0.065 |
| rs62116778 | 19 | 44894070 | Yes | No | T | G | 0.035 | -0.116 | 0 | 0.015 | 0.047 | 0.057 | 0.058 |
| rs7412 | 19 | 45412079 | Yes | No | T | C | 0.075 | -0.516 | 0 | 0.002 | 0.033 | 0.08 | 0.039 |
| rs540662190 | 19 | 45913250 | No | No | - | - | - | - | - | - | - | - | - |
| rs150262789 | 19 | 46436564 | Yes | No | T | C | 0.017 | -0.095 | 0 | -0.02 | 0.063 | -0.088 | 0.074 |
| rs574825898 | 19 | 47034252 | No | No | - | - | - | - | - | - | - | - | - |
| rs681343 | 19 | 49206462 | Yes | No | T | C | 0.494 | 0.029 | 0 | 0.035 | 0.017 | 0.04 | 0.02 |
| rs142385484 | 19 | 50016759 | Yes | No | T | C | 0.153 | -0.019 | 0 | 0.011 | 0.024 | -0.019 | 0.029 |
| rs10406304 | 19 | 57010947 | Yes | No | G | T | 0.72 | -0.012 | 0 | -0.015 | 0.018 | -0.024 | 0.022 |
| rs181976063 | 19 | 57969606 | No | No | - | - | - | - | - | - | - | - | - |
| rs35081008 | 19 | 58662235 | Yes | No | T | C | 0.155 | -0.031 | 0 | -0.017 | 0.024 | 0.049 | 0.028 |
| rs73075609 | 20 | 5580789 | Yes | No | T | C | 0.024 | 0.048 | 0 | 0.028 | 0.047 | 0.002 | 0.055 |
| rs438568 | 20 | 12958687 | Yes | No | G | A | 0.608 | 0.014 | 0 | -0.021 | 0.017 | 0.021 | 0.02 |
| rs2618566 | 20 | 17844684 | No | No | - | - | - | - | - | - | - | - | - |
| rs1044573 | 20 | 25206654 | Yes | No | G | A | 0.495 | 0.01 | 0 | 0.015 | 0.017 | 0.017 | 0.02 |
| rs7261820 | 20 | 34160840 | Yes | No | A | G | 0.137 | -0.03 | 0 | -0.007 | 0.026 | -0.026 | 0.031 |
| rs1883711 | 20 | 39179822 | Yes | No | C | G | 0.032 | 0.131 | 0 | 0.03 | 0.037 | 0.015 | 0.043 |
| rs6029549 | 20 | 39754695 | No | No | - | - | - | - | - | - | - | - | - |
| rs1800961 | 20 | 43042364 | Yes | No | T | C | 0.034 | -0.055 | 0 | 0.02 | 0.039 | -0.075 | 0.047 |
| rs3843763 | 20 | 44548193 | Yes | No | T | C | 0.265 | -0.012 | 0 | -0.009 | 0.019 | -0.021 | 0.023 |
| rs2295027 | 20 | 47582884 | Yes | No | A | G | 0.322 | 0.012 | 0 | -0.015 | 0.018 | 0.007 | 0.021 |
| rs6022851 | 20 | 52536425 | Yes | Yes | T | C | 0.446 | 0.012 | 0 | -0.033 | 0.017 | 0.02 | 0.02 |
| rs3746778 | 20 | 61341472 | No | No | - | - | - | - | - | - | - | - | - |
| rs6090040 | 20 | 62692060 | Yes | No | C | A | 0.517 | -0.014 | 0 | 0.033 | 0.017 | -0.004 | 0.02 |
| rs12106385 | 21 | 16586682 | Yes | No | A | T | 0.019 | -0.036 | 0 | 0.025 | 0.066 | 0.137 | 0.078 |
| rs2833487 | 21 | 33087863 | Yes | Yes | G | A | 0.049 | 0.031 | 0 | 0.004 | 0.037 | 0.019 | 0.043 |
| rs1963676 | 21 | 40709960 | Yes | No | T | C | 0.576 | 0.014 | 0 | 0.013 | 0.017 | 0.022 | 0.021 |
| rs5746498 | 22 | 18435794 | No | No | - | - | - | - | - | - | - | - | - |
| rs165722 | 22 | 19949013 | Yes | No | T | C | 0.529 | 0.009 | 0 | -0.017 | 0.017 | -0.02 | 0.02 |
| rs5754102 | 22 | 21916272 | No | No | - | - | - | - | - | - | - | - | - |
| rs5752963 | 22 | 30203833 | Yes | No | A | G | 0.036 | 0.027 | 0 | -0.019 | 0.046 | 0.095 | 0.056 |
| rs5755688 | 22 | 35685048 | Yes | No | G | A | 0.641 | -0.014 | 0 | -0.034 | 0.017 | -0.003 | 0.021 |
| rs132646 | 22 | 36550067 | Yes | Yes | C | T | 0.834 | -0.013 | 0 | -0.016 | 0.022 | 0.014 | 0.027 |
| rs760719 | 22 | 37460449 | Yes | No | T | C | 0.428 | 0.01 | 0 | -0.023 | 0.017 | -0.001 | 0.02 |
| rs138352 | 22 | 41268925 | Yes | Yes | G | T | 0.654 | -0.014 | 0 | 0.008 | 0.018 | 0.001 | 0.021 |
| rs3747207 | 22 | 44324855 | Yes | No | A | G | 0.222 | -0.014 | 0 | 0.002 | 0.021 | 0.011 | 0.025 |
| rs13268 | 22 | 45996298 | Yes | Yes | G | A | 0.024 | -0.038 | 0 | -0.034 | 0.059 | 0.038 | 0.071 |
| rs146471363 | 22 | 46730338 | No | No | - | - | - | - | - | - | - | - | - |
| rs9616822 | 22 | 50840573 | Yes | No | A | G | 0.359 | 0.011 | 0 | -0.026 | 0.017 | -0.007 | 0.021 |
