# Supplementary material for: Impaired glucose tolerance and cardiovascular risk factors in relation to infertility: a Mendelian randomization analysis in the Norwegian Mother, Father, and Child Cohort Study
Source: Hum Reprod. 2023 Nov 8;39(2):436–41. doi: 10.1093/humrep/dead234 (PMC10833082; doi:10.1093/humrep/dead234)
Supplement: dead234_Supplementary_Table_S5 [file dead234_supplementary_table_s5.docx]

**Supplementary Table S5.** SNPs included in HDL cholesterol-related analyses.

| **RSID** | **Chrom.** | **Position** | **Used in MR** | **Used in MR**  **+ Steiger filt.** | **Effect**  **allele** | **Other**  **allele** | **Effect**  **allele**  **freq.** | **Exposure:**  **beta** | **Exposure:**  **SE** | **Outcome**  **(women):**  **beta** | **Outcome**  **(women):**  **SE** | **Outcome**  **(men):**  **beta** | **Outcome**  **(men):**  **SE** |
| --- | --- | --- | --- | --- | --- | --- | --- | --- | --- | --- | --- | --- | --- |
| rs13303368 | 1 | 914852 | Yes | No | C | G | 0.591 | -0.011 | 0 | -0.007 | 0.017 | -0.003 | 0.02 |
| rs10753556 | 1 | 23732762 | Yes | No | G | A | 0.874 | 0.02 | 0 | 0.01 | 0.024 | 0.04 | 0.029 |
| rs75460349 | 1 | 27180088 | Yes | No | C | A | 0.024 | -0.087 | 0 | 0.024 | 0.048 | 0.013 | 0.058 |
| rs41309280 | 1 | 27688814 | Yes | No | T | C | 0.037 | -0.03 | 0 | 0.033 | 0.038 | -0.037 | 0.046 |
| rs546355 | 1 | 28364156 | Yes | Yes | C | T | 0.312 | -0.013 | 0 | -0.038 | 0.018 | -0.008 | 0.021 |
| rs4660446 | 1 | 39438889 | Yes | No | C | T | 0.656 | -0.012 | 0 | -0.016 | 0.018 | 0.018 | 0.021 |
| rs3768321 | 1 | 40035928 | Yes | No | T | G | 0.193 | -0.044 | 0 | -0.011 | 0.021 | 0.026 | 0.026 |
| rs12140153 | 1 | 62579891 | Yes | No | T | G | 0.091 | 0.019 | 0 | -0.004 | 0.026 | 0.082 | 0.031 |
| rs1168124 | 1 | 63139730 | Yes | No | T | C | 0.663 | 0.012 | 0 | -0.022 | 0.018 | -0.011 | 0.021 |
| rs6663226 | 1 | 66164748 | No | No | - | - | - | - | - | - | - | - | - |
| rs650194 | 1 | 71414863 | Yes | No | G | A | 0.662 | 0.011 | 0 | 0.005 | 0.018 | 0.016 | 0.022 |
| rs2613496 | 1 | 72754788 | Yes | No | C | T | 0.816 | -0.014 | 0 | -0.023 | 0.022 | -0.049 | 0.026 |
| rs4847419 | 1 | 93845655 | Yes | No | A | T | 0.638 | -0.026 | 0 | -0.015 | 0.018 | -0.007 | 0.021 |
| rs646776 | 1 | 109818530 | Yes | No | T | C | 0.78 | -0.035 | 0 | -0.002 | 0.02 | -0.015 | 0.024 |
| rs333947 | 1 | 110470764 | Yes | Yes | A | G | 0.15 | -0.027 | 0 | 0.018 | 0.025 | -0.012 | 0.03 |
| rs181473381 | 1 | 145472741 | No | No | - | - | - | - | - | - | - | - | - |
| rs72692849 | 1 | 149995966 | Yes | No | T | C | 0.089 | 0.025 | 0 | 0.044 | 0.028 | 0.008 | 0.034 |
| rs267738 | 1 | 150940625 | Yes | No | G | T | 0.213 | 0.022 | 0 | 0.001 | 0.021 | -0.015 | 0.024 |
| rs3806415 | 1 | 156698265 | Yes | No | T | C | 0.325 | 0.011 | 0 | -0.003 | 0.018 | -0.034 | 0.021 |
| rs9425299 | 1 | 172359100 | Yes | No | T | C | 0.57 | 0.012 | 0 | -0.023 | 0.017 | 0.014 | 0.02 |
| rs10913570 | 1 | 178523126 | Yes | Yes | C | A | 0.519 | -0.018 | 0 | -0.01 | 0.017 | -0.02 | 0.02 |
| rs1689797 | 1 | 182150978 | Yes | Yes | A | C | 0.326 | -0.026 | 0 | -0.018 | 0.018 | -0.006 | 0.022 |
| rs13303359 | 1 | 203518873 | Yes | No | C | A | 0.522 | 0.012 | 0 | -0.003 | 0.017 | 0.007 | 0.02 |
| rs3862948 | 1 | 205099959 | Yes | No | A | C | 0.234 | -0.012 | 0 | 0.003 | 0.02 | 0.007 | 0.024 |
| rs3747973 | 1 | 205677148 | Yes | No | G | A | 0.588 | 0.016 | 0 | -0.003 | 0.017 | -0.016 | 0.02 |
| rs59770119 | 1 | 219664627 | Yes | No | A | G | 0.577 | -0.017 | 0 | 0.024 | 0.017 | -0.043 | 0.02 |
| rs2642438 | 1 | 220970028 | Yes | No | G | A | 0.706 | 0.027 | 0 | -0.006 | 0.019 | 0.008 | 0.022 |
| rs56105022 | 1 | 224544746 | Yes | No | A | G | 0.035 | -0.03 | 0 | 0.009 | 0.046 | -0.004 | 0.055 |
| rs10127775 | 1 | 230295789 | Yes | No | T | A | 0.6 | 0.051 | 0 | -0.001 | 0.017 | -0.022 | 0.021 |
| rs553427 | 1 | 234852760 | Yes | No | T | C | 0.527 | 0.017 | 0 | -0.017 | 0.017 | 0.012 | 0.02 |
| rs17713729 | 2 | 249092 | Yes | Yes | C | A | 0.352 | 0.016 | 0 | -0.006 | 0.017 | 0.031 | 0.021 |
| rs6542680 | 2 | 3640142 | Yes | No | T | C | 0.808 | -0.023 | 0 | 0.012 | 0.021 | 0.02 | 0.025 |
| rs1107850 | 2 | 20371772 | Yes | No | A | G | 0.485 | -0.013 | 0 | -0.017 | 0.017 | 0.032 | 0.02 |
| rs676210 | 2 | 21231524 | Yes | No | A | G | 0.214 | 0.06 | 0 | 0.005 | 0.021 | -0.061 | 0.025 |
| rs13389106 | 2 | 30480750 | Yes | No | G | T | 0.195 | -0.012 | 0 | -0.039 | 0.021 | -0.031 | 0.024 |
| rs17326656 | 2 | 48962291 | Yes | No | T | G | 0.231 | -0.016 | 0 | 0.041 | 0.02 | 0.039 | 0.024 |
| rs805317 | 2 | 54135004 | No | No | - | - | - | - | - | - | - | - | - |
| rs17049712 | 2 | 58961136 | Yes | No | T | C | 0.295 | -0.012 | 0 | -0.009 | 0.018 | -0.011 | 0.022 |
| rs2540949 | 2 | 65284231 | Yes | No | T | A | 0.383 | 0.016 | 0 | 0.001 | 0.017 | -0.011 | 0.021 |
| rs11883967 | 2 | 66673862 | No | No | - | - | - | - | - | - | - | - | - |
| rs7586605 | 2 | 85561141 | Yes | No | T | C | 0.73 | 0.012 | 0 | -0.015 | 0.019 | -0.02 | 0.022 |
| rs4851250 | 2 | 100729293 | Yes | No | C | T | 0.172 | 0.015 | 0 | -0.009 | 0.023 | -0.014 | 0.027 |
| rs11688682 | 2 | 121347612 | No | No | - | - | - | - | - | - | - | - | - |
| rs6758044 | 2 | 135592245 | Yes | No | C | T | 0.387 | 0.011 | 0 | -0.008 | 0.018 | 0.036 | 0.022 |
| rs3739020 | 2 | 136602491 | Yes | No | G | T | 0.216 | 0.018 | 0 | -0.012 | 0.023 | -0.003 | 0.028 |
| rs13389219 | 2 | 165528876 | Yes | No | T | C | 0.4 | 0.029 | 0 | -0.011 | 0.017 | -0.008 | 0.02 |
| rs1866631 | 2 | 174075761 | Yes | No | A | G | 0.584 | -0.011 | 0 | -0.017 | 0.017 | 0.007 | 0.021 |
| rs7581601 | 2 | 203497786 | Yes | No | C | A | 0.315 | -0.018 | 0 | -0.022 | 0.018 | -0.011 | 0.021 |
| rs1047891 | 2 | 211540507 | Yes | No | A | C | 0.308 | -0.016 | 0 | 0.003 | 0.018 | -0.039 | 0.022 |
| rs78058190 | 2 | 219699999 | No | No | - | - | - | - | - | - | - | - | - |
| rs2943645 | 2 | 227099180 | Yes | No | T | C | 0.636 | -0.037 | 0 | 0.001 | 0.017 | 0.033 | 0.021 |
| rs11694828 | 2 | 228565656 | Yes | No | A | G | 0.451 | 0.015 | 0 | -0.027 | 0.017 | -0.002 | 0.021 |
| rs4675812 | 2 | 242395674 | Yes | Yes | A | G | 0.59 | 0.013 | 0 | 0.012 | 0.017 | -0.008 | 0.021 |
| rs12485478 | 3 | 12351223 | Yes | No | G | A | 0.027 | -0.054 | 0 | -0.041 | 0.052 | 0.057 | 0.063 |
| rs2455850 | 3 | 15782711 | Yes | No | C | A | 0.684 | 0.014 | 0 | -0.003 | 0.018 | -0.011 | 0.022 |
| rs2044754 | 3 | 24292940 | Yes | No | G | A | 0.109 | -0.017 | 0 | -0.034 | 0.026 | -0.026 | 0.031 |
| rs11706926 | 3 | 36978647 | Yes | No | G | A | 0.552 | 0.013 | 0 | -0.007 | 0.017 | 0.045 | 0.02 |
| rs2290547 | 3 | 47061183 | Yes | No | A | G | 0.172 | -0.02 | 0 | -0.016 | 0.022 | 0.002 | 0.026 |
| rs7634917 | 3 | 50049299 | Yes | No | G | T | 0.478 | 0.022 | 0 | -0.009 | 0.017 | 0.027 | 0.02 |
| rs138789058 | 3 | 52124388 | Yes | No | T | C | 0.018 | -0.04 | 0 | 0.015 | 0.055 | -0.005 | 0.065 |
| rs2240921 | 3 | 52830764 | Yes | No | T | C | 0.116 | 0.026 | 0 | 0.011 | 0.027 | -0.077 | 0.032 |
| rs13066793 | 3 | 87037543 | No | No | - | - | - | - | - | - | - | - | - |
| rs4516580 | 3 | 114509447 | Yes | No | C | T | 0.833 | -0.015 | 0 | -0.01 | 0.023 | -0.017 | 0.028 |
| rs3732356 | 3 | 119529113 | Yes | No | T | G | 0.932 | -0.03 | 0 | -0.025 | 0.034 | -0.01 | 0.04 |
| rs11708067 | 3 | 123065778 | Yes | No | G | A | 0.227 | -0.013 | 0 | -0.037 | 0.02 | 0.012 | 0.024 |
| rs2811468 | 3 | 129296847 | Yes | No | G | A | 0.195 | 0.014 | 0 | -0.007 | 0.023 | 0.024 | 0.027 |
| rs1154988 | 3 | 135925191 | Yes | No | A | T | 0.78 | -0.026 | 0 | 0.041 | 0.02 | 0.034 | 0.024 |
| rs62271373 | 3 | 150066540 | Yes | No | A | T | 0.053 | -0.037 | 0 | 0.056 | 0.04 | -0.091 | 0.047 |
| rs13082076 | 3 | 152085277 | Yes | No | C | T | 0.296 | 0.012 | 0 | 0.008 | 0.019 | 0.019 | 0.023 |
| rs10049088 | 3 | 156797648 | Yes | No | T | C | 0.384 | 0.021 | 0 | 0.004 | 0.017 | -0.017 | 0.021 |
| rs1516725 | 3 | 185824004 | Yes | No | C | T | 0.866 | 0.03 | 0 | -0.029 | 0.027 | -0.028 | 0.033 |
| rs1051613 | 4 | 951179 | Yes | No | A | G | 0.538 | 0.013 | 0 | -0.016 | 0.017 | 0.029 | 0.02 |
| rs4450871 | 4 | 4990298 | No | No | - | - | - | - | - | - | - | - | - |
| rs7694606 | 4 | 18028987 | Yes | No | A | G | 0.149 | -0.014 | 0 | 0.025 | 0.025 | -0.031 | 0.03 |
| rs73243877 | 4 | 26047616 | Yes | No | G | A | 0.163 | -0.021 | 0 | 0.015 | 0.023 | 0.003 | 0.028 |
| rs150361490 | 4 | 55530748 | No | No | - | - | - | - | - | - | - | - | - |
| rs35662434 | 4 | 69339933 | Yes | No | G | A | 0.221 | 0.015 | 0 | 0.007 | 0.02 | -0.003 | 0.024 |
| rs573930512 | 4 | 74512280 | No | No | - | - | - | - | - | - | - | - | - |
| rs6831339 | 4 | 83906408 | Yes | No | T | C | 0.248 | -0.012 | 0 | 0.019 | 0.02 | -0.002 | 0.024 |
| rs342469 | 4 | 88050554 | Yes | No | A | G | 0.602 | -0.015 | 0 | 0.013 | 0.017 | 0.012 | 0.02 |
| rs2167750 | 4 | 89730074 | Yes | No | T | C | 0.464 | -0.019 | 0 | -0.005 | 0.017 | -0.034 | 0.02 |
| rs547953616 | 4 | 100020668 | No | No | - | - | - | - | - | - | - | - | - |
| rs951941 | 4 | 100541411 | Yes | No | A | G | 0.035 | 0.029 | 0 | -0.025 | 0.044 | -0.017 | 0.053 |
| rs12511373 | 4 | 102666785 | No | No | - | - | - | - | - | - | - | - | - |
| rs13107325 | 4 | 103188709 | Yes | No | T | C | 0.064 | -0.083 | 0 | -0.037 | 0.04 | -0.011 | 0.048 |
| rs74332078 | 4 | 103766720 | Yes | No | A | G | 0.032 | -0.035 | 0 | 0.001 | 0.061 | 0.007 | 0.073 |
| rs9884482 | 4 | 106081636 | Yes | No | C | T | 0.378 | -0.012 | 0 | 0.014 | 0.017 | -0.01 | 0.021 |
| rs138307849 | 4 | 110095620 | No | No | - | - | - | - | - | - | - | - | - |
| rs114816312 | 4 | 110638824 | No | No | - | - | - | - | - | - | - | - | - |
| rs62328321 | 4 | 143319721 | Yes | No | T | C | 0.629 | 0.011 | 0 | -0.004 | 0.018 | -0.012 | 0.021 |
| rs72729623 | 4 | 154208278 | Yes | No | T | C | 0.145 | -0.016 | 0 | 0.022 | 0.022 | -0.035 | 0.027 |
| rs1425485 | 4 | 157681890 | Yes | No | T | C | 0.228 | 0.019 | 0 | -0.014 | 0.02 | -0.039 | 0.024 |
| rs76086106 | 5 | 39491186 | Yes | No | A | G | 0.042 | 0.027 | 0 | -0.005 | 0.038 | 0.045 | 0.045 |
| rs6866562 | 5 | 52697383 | Yes | No | A | G | 0.539 | -0.011 | 0 | -0.023 | 0.017 | -0.017 | 0.02 |
| rs1664781 | 5 | 53276301 | Yes | No | A | G | 0.688 | -0.014 | 0 | 0.038 | 0.018 | 0.016 | 0.022 |
| rs28650790 | 5 | 55861464 | Yes | No | T | C | 0.18 | -0.03 | 0 | 0.031 | 0.023 | -0.043 | 0.028 |
| rs4976033 | 5 | 67714246 | Yes | No | G | A | 0.405 | -0.014 | 0 | 0.007 | 0.017 | -0.025 | 0.02 |
| rs970305 | 5 | 72942296 | Yes | No | G | A | 0.716 | 0.013 | 0 | -0.034 | 0.019 | -0.004 | 0.022 |
| rs2307111 | 5 | 75003678 | Yes | No | C | T | 0.394 | 0.016 | 0 | -0.003 | 0.017 | 0.002 | 0.021 |
| rs9293683 | 5 | 75700310 | Yes | Yes | T | C | 0.661 | 0.011 | 0 | -0.013 | 0.018 | -0.015 | 0.022 |
| rs115912456 | 5 | 82815158 | Yes | No | G | A | 0.042 | 0.029 | 0 | -0.023 | 0.043 | 0.047 | 0.052 |
| rs12234017 | 5 | 118711834 | Yes | No | G | A | 0.257 | 0.016 | 0 | -0.017 | 0.019 | 0.018 | 0.023 |
| rs35897671 | 5 | 127349745 | Yes | No | T | C | 0.335 | 0.011 | 0 | -0.011 | 0.018 | -0.05 | 0.021 |
| rs10479001 | 5 | 131607721 | Yes | No | T | C | 0.042 | 0.024 | 0 | 0.003 | 0.046 | -0.074 | 0.055 |
| rs72801474 | 5 | 132444128 | Yes | No | A | G | 0.096 | 0.026 | 0 | -0.044 | 0.027 | 0.069 | 0.032 |
| rs254559 | 5 | 134444982 | Yes | No | A | C | 0.408 | -0.011 | 0 | -0.009 | 0.017 | -0.041 | 0.021 |
| rs12523418 | 5 | 141707225 | Yes | No | G | A | 0.32 | 0.012 | 0 | -0.018 | 0.018 | -0.014 | 0.022 |
| rs1438588 | 5 | 153444950 | Yes | No | G | A | 0.633 | -0.012 | 0 | 0.002 | 0.018 | -0.003 | 0.021 |
| rs1650527 | 5 | 158022724 | Yes | No | T | C | 0.228 | -0.021 | 0 | 0.009 | 0.02 | 0.002 | 0.024 |
| rs1473247 | 5 | 158603571 | Yes | Yes | C | T | 0.254 | 0.012 | 0 | -0.018 | 0.02 | 0.034 | 0.023 |
| rs72812818 | 5 | 173356752 | Yes | No | C | G | 0.311 | -0.011 | 0 | -0.024 | 0.018 | 0.012 | 0.021 |
| rs6882591 | 5 | 176546460 | Yes | No | A | G | 0.147 | 0.017 | 0 | -0.018 | 0.023 | 0.008 | 0.028 |
| rs138692142 | 5 | 180349778 | No | No | - | - | - | - | - | - | - | - | - |
| rs1240811 | 6 | 16818805 | Yes | No | T | C | 0.309 | 0.011 | 0 | -0.018 | 0.019 | -0.016 | 0.022 |
| rs806794 | 6 | 26200677 | Yes | No | G | A | 0.289 | 0.011 | 0 | 0.021 | 0.019 | -0.046 | 0.022 |
| rs9368561 | 6 | 28168343 | Yes | No | T | C | 0.132 | -0.016 | 0 | -0.011 | 0.025 | -0.021 | 0.03 |
| rs1611282 | 6 | 29730002 | Yes | No | T | G | 0.58 | 0.011 | 0 | -0.027 | 0.017 | 0.006 | 0.02 |
| rs1264377 | 6 | 30764907 | Yes | No | A | G | 0.155 | -0.015 | 0 | -0.017 | 0.024 | -0.044 | 0.029 |
| rs76716928 | 6 | 31295941 | No | No | - | - | - | - | - | - | - | - | - |
| rs115003989 | 6 | 31925921 | Yes | No | A | G | 0.023 | -0.042 | 0 | -0.024 | 0.041 | 0.046 | 0.049 |
| rs17209803 | 6 | 32443061 | Yes | No | A | C | 0.047 | -0.037 | 0 | -0.036 | 0.036 | 0.065 | 0.044 |
| rs115675705 | 6 | 34094919 | Yes | No | G | A | 0.031 | -0.041 | 0 | 0.028 | 0.051 | 0.081 | 0.061 |
| rs2263329 | 6 | 34595543 | Yes | No | C | T | 0.358 | -0.029 | 0 | -0.018 | 0.018 | -0.038 | 0.021 |
| rs11754612 | 6 | 35476562 | Yes | No | A | T | 0.115 | -0.017 | 0 | -0.009 | 0.026 | 0.027 | 0.031 |
| rs142449754 | 6 | 41986427 | Yes | No | T | C | 0.249 | -0.019 | 0 | 0.007 | 0.019 | 0.008 | 0.023 |
| rs9296406 | 6 | 42940810 | Yes | No | C | T | 0.539 | 0.017 | 0 | 0.023 | 0.017 | -0.033 | 0.02 |
| rs998584 | 6 | 43757896 | Yes | No | A | C | 0.478 | -0.031 | 0 | 0.01 | 0.017 | -0.008 | 0.02 |
| rs2608629 | 6 | 52625794 | Yes | No | A | G | 0.648 | 0.013 | 0 | 0.011 | 0.018 | -0.006 | 0.021 |
| rs71721600 | 6 | 98584717 | No | No | - | - | - | - | - | - | - | - | - |
| rs565216100 | 6 | 109064220 | No | No | - | - | - | - | - | - | - | - | - |
| rs11759230 | 6 | 109640321 | Yes | No | C | A | 0.342 | -0.017 | 0 | -0.022 | 0.018 | -0.028 | 0.021 |
| rs1338668 | 6 | 116394809 | Yes | No | A | G | 0.412 | 0.015 | 0 | 0.003 | 0.017 | -0.015 | 0.02 |
| rs72959041 | 6 | 127454893 | Yes | No | A | G | 0.052 | -0.047 | 0 | -0.066 | 0.035 | -0.027 | 0.042 |
| rs2245133 | 6 | 131931092 | Yes | No | C | T | 0.167 | -0.014 | 0 | 0.03 | 0.022 | -0.033 | 0.026 |
| rs9399136 | 6 | 135402339 | Yes | No | C | T | 0.26 | -0.012 | 0 | -0.026 | 0.019 | -0.001 | 0.022 |
| rs6924387 | 6 | 137082948 | Yes | No | G | A | 0.414 | -0.013 | 0 | -0.002 | 0.017 | 0.02 | 0.02 |
| rs2750415 | 6 | 139261477 | Yes | No | A | G | 0.825 | -0.016 | 0 | 0.01 | 0.022 | 0.004 | 0.027 |
| rs17585887 | 6 | 139835498 | Yes | No | C | T | 0.582 | 0.021 | 0 | -0.02 | 0.017 | 0.007 | 0.02 |
| rs672341 | 6 | 153455994 | Yes | No | A | G | 0.41 | 0.014 | 0 | -0.007 | 0.017 | -0.042 | 0.02 |
| rs78425119 | 6 | 160501825 | Yes | No | A | G | 0.066 | -0.026 | 0 | -0.013 | 0.032 | 0.067 | 0.039 |
| rs41272086 | 6 | 161008646 | Yes | No | A | G | 0.101 | -0.058 | 0 | -0.043 | 0.025 | -0.004 | 0.03 |
| rs147829643 | 6 | 161588372 | Yes | No | T | C | 0.037 | -0.03 | 0 | 0.032 | 0.037 | -0.066 | 0.045 |
| rs9347737 | 6 | 163740322 | Yes | No | G | A | 0.423 | -0.012 | 0 | -0.005 | 0.017 | 0.021 | 0.02 |
| rs34409228 | 7 | 1102674 | Yes | No | T | C | 0.126 | 0.028 | 0 | 0.032 | 0.025 | 0.011 | 0.03 |
| rs144787122 | 7 | 2296552 | No | No | - | - | - | - | - | - | - | - | - |
| rs79949326 | 7 | 6461310 | Yes | No | T | C | 0.252 | 0.028 | 0 | 0.003 | 0.019 | 0.003 | 0.023 |
| rs1990622 | 7 | 12283787 | Yes | Yes | G | A | 0.411 | -0.012 | 0 | -0.009 | 0.017 | -0.031 | 0.02 |
| rs6968554 | 7 | 17287106 | Yes | No | G | A | 0.635 | -0.013 | 0 | -0.012 | 0.018 | 0.008 | 0.021 |
| rs10242866 | 7 | 17920613 | Yes | Yes | T | C | 0.393 | -0.023 | 0 | -0.011 | 0.017 | -0.021 | 0.021 |
| rs1534696 | 7 | 26397239 | Yes | No | A | C | 0.552 | 0.016 | 0 | -0.015 | 0.017 | 0.012 | 0.02 |
| rs12533197 | 7 | 36192606 | Yes | No | G | T | 0.443 | -0.015 | 0 | -0.011 | 0.017 | -0.001 | 0.02 |
| rs138994411 | 7 | 38265179 | No | No | - | - | - | - | - | - | - | - | - |
| rs876039 | 7 | 50308811 | Yes | Yes | C | G | 0.313 | 0.015 | 0 | 0.022 | 0.018 | 0.019 | 0.022 |
| rs55747707 | 7 | 73037366 | Yes | No | A | G | 0.196 | 0.038 | 0 | -0.027 | 0.021 | -0.008 | 0.025 |
| rs117649635 | 7 | 74297908 | Yes | No | G | C | 0.054 | -0.024 | 0 | 0.033 | 0.043 | 0.058 | 0.05 |
| rs1761661 | 7 | 80238041 | Yes | No | C | A | 0.455 | 0.011 | 0 | 0.008 | 0.017 | 0.012 | 0.02 |
| rs705379 | 7 | 94953895 | Yes | No | A | G | 0.464 | -0.01 | 0 | -0.002 | 0.017 | 0.02 | 0.02 |
| rs4730222 | 7 | 106810293 | Yes | No | C | G | 0.2 | 0.014 | 0 | -0.014 | 0.021 | -0.027 | 0.025 |
| rs10229792 | 7 | 116982907 | Yes | No | A | G | 0.362 | -0.012 | 0 | 0.017 | 0.017 | -0.05 | 0.021 |
| rs11762784 | 7 | 130424646 | Yes | No | A | G | 0.495 | 0.03 | 0 | -0.005 | 0.017 | 0.03 | 0.02 |
| rs10273527 | 7 | 139036598 | Yes | No | T | G | 0.234 | 0.012 | 0 | 0.014 | 0.021 | -0.03 | 0.025 |
| rs7794796 | 7 | 150540196 | Yes | No | T | C | 0.332 | -0.017 | 0 | -0.02 | 0.018 | -0.044 | 0.021 |
| rs11783088 | 8 | 6620392 | Yes | No | G | A | 0.25 | -0.012 | 0 | 0.002 | 0.02 | 0.041 | 0.024 |
| rs9987289 | 8 | 9183358 | Yes | No | G | A | 0.908 | 0.093 | 0 | -0.005 | 0.027 | 0.043 | 0.033 |
| rs4645567 | 8 | 9723964 | Yes | No | A | T | 0.786 | -0.015 | 0 | -0.008 | 0.02 | 0.023 | 0.024 |
| rs9657541 | 8 | 10643164 | Yes | No | T | C | 0.202 | -0.018 | 0 | 0.013 | 0.021 | 0.046 | 0.025 |
| rs113920320 | 8 | 19292422 | No | No | - | - | - | - | - | - | - | - | - |
| rs15285 | 8 | 19824667 | Yes | Yes | T | C | 0.287 | 0.107 | 0 | 0.004 | 0.019 | -0.018 | 0.023 |
| rs73221948 | 8 | 25464670 | No | No | - | - | - | - | - | - | - | - | - |
| rs75772194 | 8 | 36846435 | Yes | No | G | A | 0.166 | -0.013 | 0 | 0.022 | 0.024 | -0.026 | 0.029 |
| rs6994609 | 8 | 64799004 | Yes | No | G | T | 0.474 | 0.011 | 0 | 0.025 | 0.017 | -0.039 | 0.02 |
| rs34246353 | 8 | 71138299 | Yes | No | C | T | 0.093 | -0.025 | 0 | -0.058 | 0.028 | -0.001 | 0.033 |
| rs28471687 | 8 | 72459582 | Yes | No | G | A | 0.073 | -0.022 | 0 | -0.021 | 0.034 | 0.057 | 0.041 |
| rs35910594 | 8 | 103924469 | No | No | - | - | - | - | - | - | - | - | - |
| rs2941657 | 8 | 106372180 | Yes | No | G | A | 0.531 | -0.011 | 0 | 0.021 | 0.017 | 0.029 | 0.02 |
| rs3808460 | 8 | 116597635 | Yes | Yes | C | T | 0.574 | 0.033 | 0 | -0.004 | 0.017 | -0.013 | 0.02 |
| rs10955991 | 8 | 121867780 | Yes | Yes | C | T | 0.669 | -0.02 | 0 | -0.003 | 0.018 | 0.024 | 0.021 |
| rs2954038 | 8 | 126507389 | Yes | No | A | C | 0.697 | 0.043 | 0 | -0.011 | 0.019 | 0.017 | 0.022 |
| rs7817574 | 8 | 144302570 | Yes | No | C | T | 0.195 | 0.028 | 0 | -0.007 | 0.02 | -0.042 | 0.024 |
| rs4741016 | 9 | 1036132 | Yes | No | C | T | 0.23 | -0.014 | 0 | -0.021 | 0.02 | 0 | 0.024 |
| rs77375493 | 9 | 5073770 | No | No | - | - | - | - | - | - | - | - | - |
| rs543458 | 9 | 14068327 | Yes | No | C | A | 0.19 | 0.013 | 0 | -0.029 | 0.022 | -0.003 | 0.027 |
| rs686030 | 9 | 15304782 | Yes | Yes | A | C | 0.858 | 0.05 | 0 | -0.031 | 0.024 | 0.004 | 0.028 |
| rs62543565 | 9 | 16901067 | Yes | No | A | C | 0.637 | 0.011 | 0 | -0.03 | 0.017 | -0.02 | 0.021 |
| rs1412234 | 9 | 28410683 | Yes | No | C | T | 0.32 | -0.013 | 0 | -0.011 | 0.018 | 0.021 | 0.022 |
| rs11795343 | 9 | 32523737 | Yes | Yes | C | T | 0.404 | 0.01 | 0 | -0.002 | 0.017 | 0.03 | 0.02 |
| rs746125435 | 9 | 105805994 | No | No | - | - | - | - | - | - | - | - | - |
| rs549466628 | 9 | 106532723 | No | No | - | - | - | - | - | - | - | - | - |
| rs186793936 | 9 | 107093784 | No | No | - | - | - | - | - | - | - | - | - |
| rs2740488 | 9 | 107661742 | Yes | No | C | A | 0.261 | -0.069 | 0 | -0.001 | 0.019 | 0.045 | 0.023 |
| rs577703186 | 9 | 108359753 | No | No | - | - | - | - | - | - | - | - | - |
| rs535372564 | 9 | 109062494 | No | No | - | - | - | - | - | - | - | - | - |
| rs10733608 | 9 | 117148430 | Yes | No | T | G | 0.476 | 0.012 | 0 | -0.006 | 0.017 | -0.009 | 0.02 |
| rs4620350 | 9 | 126539944 | Yes | No | G | A | 0.065 | -0.02 | 0 | 0.087 | 0.032 | -0.068 | 0.038 |
| rs2519093 | 9 | 136141870 | Yes | No | T | C | 0.187 | 0.017 | 0 | 0.022 | 0.02 | 0.01 | 0.024 |
| rs75406471 | 10 | 5257647 | Yes | No | A | G | 0.156 | -0.014 | 0 | -0.014 | 0.022 | -0.023 | 0.026 |
| rs11254464 | 10 | 17265447 | Yes | No | C | T | 0.421 | 0.014 | 0 | -0.003 | 0.017 | 0.002 | 0.021 |
| rs12358424 | 10 | 33641149 | Yes | No | G | T | 0.09 | -0.026 | 0 | -0.024 | 0.029 | -0.053 | 0.035 |
| rs55665473 | 10 | 46022005 | Yes | No | A | G | 0.239 | 0.026 | 0 | 0.006 | 0.02 | 0.02 | 0.024 |
| rs10826337 | 10 | 61409469 | Yes | No | A | G | 0.417 | -0.012 | 0 | -0.022 | 0.017 | -0.012 | 0.02 |
| rs7924036 | 10 | 65191645 | Yes | No | T | G | 0.513 | 0.011 | 0 | -0.052 | 0.017 | 0.013 | 0.02 |
| rs703966 | 10 | 80954251 | Yes | Yes | A | G | 0.418 | 0.012 | 0 | -0.022 | 0.017 | -0.009 | 0.02 |
| rs17875327 | 10 | 94274809 | Yes | No | G | A | 0.11 | -0.018 | 0 | -0.013 | 0.025 | -0.024 | 0.03 |
| rs4418728 | 10 | 94839724 | Yes | No | T | G | 0.451 | 0.019 | 0 | -0.002 | 0.017 | 0 | 0.02 |
| rs7091593 | 10 | 95342772 | Yes | No | T | A | 0.113 | -0.021 | 0 | -0.022 | 0.027 | 0.024 | 0.033 |
| rs2862954 | 10 | 101912064 | Yes | No | C | T | 0.482 | 0.015 | 0 | 0.012 | 0.017 | -0.007 | 0.02 |
| rs2792751 | 10 | 113940329 | Yes | No | C | T | 0.719 | -0.031 | 0 | 0.027 | 0.019 | 0.005 | 0.023 |
| rs72823014 | 10 | 115786236 | Yes | No | A | G | 0.118 | 0.031 | 0 | 0.02 | 0.026 | 0.024 | 0.031 |
| rs11197783 | 10 | 118407116 | No | No | - | - | - | - | - | - | - | - | - |
| rs12415055 | 10 | 122869552 | Yes | No | G | A | 0.112 | -0.018 | 0 | 0.016 | 0.027 | -0.01 | 0.033 |
| rs10901854 | 10 | 126733643 | Yes | No | C | T | 0.271 | -0.011 | 0 | -0.018 | 0.019 | 0.003 | 0.023 |
| rs140201358 | 11 | 823586 | Yes | No | G | C | 0.013 | -0.059 | 0 | -0.102 | 0.068 | -0.014 | 0.082 |
| rs1661052 | 11 | 2942593 | Yes | No | A | G | 0.908 | 0.026 | 0 | -0.001 | 0.031 | 0.065 | 0.037 |
| rs2218793 | 11 | 10380828 | Yes | No | A | C | 0.286 | -0.013 | 0 | 0 | 0.019 | 0.006 | 0.022 |
| rs150090666 | 11 | 14865399 | No | No | - | - | - | - | - | - | - | - | - |
| rs7944119 | 11 | 27722298 | Yes | No | T | G | 0.315 | -0.015 | 0 | 0.022 | 0.018 | 0.011 | 0.021 |
| rs11031791 | 11 | 32469729 | Yes | Yes | T | A | 0.394 | -0.012 | 0 | -0.077 | 0.017 | 0.007 | 0.021 |
| rs77403571 | 11 | 45913607 | Yes | No | A | G | 0.06 | 0.036 | 0 | -0.018 | 0.036 | -0.038 | 0.043 |
| rs10838684 | 11 | 47283862 | Yes | No | G | A | 0.311 | 0.042 | 0 | -0.029 | 0.018 | -0.017 | 0.022 |
| rs11229252 | 11 | 55129640 | Yes | Yes | T | C | 0.113 | 0.039 | 0 | -0.008 | 0.025 | 0.004 | 0.03 |
| rs35366909 | 11 | 57065586 | Yes | No | T | C | 0.047 | 0.029 | 0 | -0.001 | 0.037 | 0.034 | 0.044 |
| rs12575459 | 11 | 58162382 | Yes | No | A | G | 0.077 | 0.019 | 0 | 0.039 | 0.029 | -0.022 | 0.035 |
| rs174564 | 11 | 61588305 | Yes | No | G | A | 0.351 | -0.046 | 0 | 0.002 | 0.018 | 0.011 | 0.021 |
| rs56271783 | 11 | 64004723 | Yes | No | C | G | 0.047 | -0.05 | 0 | -0.064 | 0.038 | -0.06 | 0.044 |
| rs10750766 | 11 | 65473798 | Yes | No | A | C | 0.71 | -0.018 | 0 | -0.025 | 0.019 | 0.014 | 0.022 |
| rs4930352 | 11 | 66066993 | Yes | No | T | G | 0.491 | 0.011 | 0 | -0.001 | 0.017 | -0.034 | 0.02 |
| rs12794995 | 11 | 67372946 | Yes | No | A | G | 0.258 | 0.012 | 0 | -0.008 | 0.019 | -0.022 | 0.023 |
| rs2003892 | 11 | 68593929 | Yes | No | A | G | 0.334 | -0.014 | 0 | 0 | 0.017 | 0.029 | 0.021 |
| rs673335 | 11 | 75450576 | Yes | No | C | T | 0.174 | -0.03 | 0 | 0.006 | 0.021 | -0.008 | 0.026 |
| rs10891054 | 11 | 109966513 | Yes | No | C | A | 0.695 | -0.016 | 0 | 0.034 | 0.018 | 0.007 | 0.022 |
| rs964184 | 11 | 116648917 | Yes | Yes | C | G | 0.865 | 0.108 | 0 | 0.033 | 0.025 | 0.003 | 0.03 |
| rs192489212 | 11 | 117220429 | No | No | - | - | - | - | - | - | - | - | - |
| rs3825061 | 11 | 118944675 | Yes | No | T | C | 0.386 | -0.016 | 0 | 0.006 | 0.017 | 0.019 | 0.021 |
| rs6589939 | 11 | 122518525 | Yes | Yes | G | A | 0.383 | 0.024 | 0 | -0.006 | 0.017 | -0.018 | 0.021 |
| rs73632737 | 11 | 126226775 | Yes | No | C | G | 0.072 | -0.037 | 0 | -0.021 | 0.031 | 0.053 | 0.037 |
| rs56196860 | 12 | 2908330 | No | No | - | - | - | - | - | - | - | - | - |
| rs7298463 | 12 | 6663964 | Yes | No | C | G | 0.171 | -0.018 | 0 | 0.029 | 0.022 | -0.016 | 0.026 |
| rs7305678 | 12 | 7681181 | Yes | No | G | T | 0.859 | -0.02 | 0 | -0.014 | 0.023 | -0.038 | 0.028 |
| rs4883201 | 12 | 9082581 | Yes | No | G | A | 0.105 | -0.027 | 0 | -0.001 | 0.029 | -0.043 | 0.034 |
| rs11045172 | 12 | 20470221 | Yes | Yes | C | A | 0.199 | 0.027 | 0 | 0.004 | 0.021 | -0.01 | 0.025 |
| rs10842703 | 12 | 26456188 | Yes | No | T | A | 0.251 | -0.012 | 0 | 0.004 | 0.019 | 0.036 | 0.022 |
| rs1126930 | 12 | 49399132 | Yes | No | C | G | 0.034 | -0.029 | 0 | 0.015 | 0.047 | -0.112 | 0.055 |
| rs7132908 | 12 | 50263148 | Yes | No | A | G | 0.385 | -0.011 | 0 | 0 | 0.017 | -0.016 | 0.02 |
| rs9943712 | 12 | 51160715 | Yes | No | T | C | 0.391 | 0.011 | 0 | -0.029 | 0.017 | 0.011 | 0.021 |
| rs11170538 | 12 | 53796744 | No | No | - | - | - | - | - | - | - | - | - |
| rs11171739 | 12 | 56470625 | Yes | No | T | C | 0.578 | -0.011 | 0 | 0 | 0.017 | -0.021 | 0.02 |
| rs73119306 | 12 | 57826982 | Yes | No | G | A | 0.249 | 0.027 | 0 | 0.028 | 0.019 | 0.034 | 0.022 |
| rs12368865 | 12 | 58422642 | Yes | No | G | A | 0.096 | 0.021 | 0 | -0.001 | 0.025 | -0.035 | 0.03 |
| rs1012306 | 12 | 101888063 | Yes | Yes | T | C | 0.558 | 0.01 | 0 | 0.005 | 0.017 | 0.024 | 0.02 |
| rs11114022 | 12 | 109050204 | Yes | No | A | G | 0.36 | -0.012 | 0 | -0.011 | 0.017 | -0.028 | 0.021 |
| rs2338104 | 12 | 109895168 | No | No | - | - | - | - | - | - | - | - | - |
| rs3184504 | 12 | 111884608 | Yes | No | C | T | 0.527 | 0.022 | 0 | 0.006 | 0.017 | 0.028 | 0.02 |
| rs10774579 | 12 | 121405210 | Yes | No | C | T | 0.481 | -0.015 | 0 | -0.024 | 0.017 | -0.007 | 0.02 |
| rs12369179 | 12 | 122963550 | Yes | No | T | C | 0.083 | -0.032 | 0 | -0.002 | 0.031 | -0.037 | 0.037 |
| rs12366872 | 12 | 123804721 | Yes | Yes | C | G | 0.095 | 0.043 | 0 | -0.014 | 0.028 | -0.006 | 0.035 |
| rs7133378 | 12 | 124409502 | Yes | No | A | G | 0.32 | 0.033 | 0 | -0.005 | 0.018 | -0.012 | 0.022 |
| rs61941677 | 12 | 125325010 | Yes | No | G | C | 0.13 | -0.062 | 0 | 0.041 | 0.024 | 0.001 | 0.029 |
| rs76428106 | 13 | 28604007 | No | No | - | - | - | - | - | - | - | - | - |
| rs78424108 | 13 | 41673457 | Yes | No | G | C | 0.071 | -0.026 | 0 | -0.024 | 0.034 | 0.101 | 0.04 |
| rs549058 | 13 | 51201045 | Yes | No | T | G | 0.125 | 0.018 | 0 | -0.025 | 0.023 | 0.02 | 0.028 |
| rs9537803 | 13 | 58366634 | Yes | No | T | C | 0.721 | 0.011 | 0 | 0.02 | 0.018 | 0.004 | 0.022 |
| rs200748895 | 13 | 108960380 | No | No | - | - | - | - | - | - | - | - | - |
| rs9604045 | 13 | 113927208 | No | No | - | - | - | - | - | - | - | - | - |
| rs1955512 | 14 | 33175822 | Yes | No | A | G | 0.571 | 0.01 | 0 | -0.026 | 0.017 | -0.002 | 0.021 |
| rs8022053 | 14 | 65867947 | Yes | No | G | T | 0.666 | 0.011 | 0 | -0.008 | 0.018 | -0.022 | 0.021 |
| rs72729582 | 14 | 69149372 | Yes | No | G | A | 0.073 | 0.023 | 0 | -0.03 | 0.031 | -0.033 | 0.037 |
| rs1044527 | 14 | 70833819 | No | No | - | - | - | - | - | - | - | - | - |
| rs13379043 | 14 | 74250126 | Yes | No | C | T | 0.271 | 0.019 | 0 | 0.035 | 0.019 | 0.013 | 0.023 |
| rs8021280 | 14 | 75290018 | No | No | - | - | - | - | - | - | - | - | - |
| rs2371562 | 14 | 81619474 | Yes | No | A | C | 0.263 | 0.011 | 0 | 0.015 | 0.019 | -0.015 | 0.022 |
| rs3825667 | 14 | 89804373 | Yes | No | A | T | 0.805 | 0.015 | 0 | -0.036 | 0.022 | -0.008 | 0.026 |
| rs113953439 | 14 | 100380290 | Yes | No | A | G | 0.175 | -0.014 | 0 | 0.02 | 0.022 | 0.035 | 0.027 |
| rs12888955 | 14 | 103256877 | Yes | No | A | G | 0.654 | 0.013 | 0 | 0.017 | 0.018 | 0.021 | 0.021 |
| rs2494747 | 14 | 105258437 | Yes | No | T | G | 0.615 | -0.026 | 0 | -0.036 | 0.018 | 0.005 | 0.021 |
| rs275179 | 15 | 39447529 | Yes | No | G | A | 0.158 | 0.015 | 0 | 0.005 | 0.023 | -0.028 | 0.028 |
| rs3803357 | 15 | 40751555 | Yes | No | A | C | 0.493 | 0.011 | 0 | -0.002 | 0.017 | -0.014 | 0.02 |
| rs7170463 | 15 | 41888918 | Yes | No | G | A | 0.312 | 0.013 | 0 | 0.003 | 0.018 | -0.064 | 0.022 |
| rs55707100 | 15 | 43820717 | Yes | No | T | C | 0.026 | -0.102 | 0 | 0.043 | 0.056 | -0.036 | 0.066 |
| rs1901529 | 15 | 45001900 | Yes | No | G | A | 0.12 | -0.017 | 0 | 0.047 | 0.027 | 0.023 | 0.032 |
| rs261290 | 15 | 58678720 | Yes | No | C | T | 0.655 | -0.106 | 0 | -0.017 | 0.018 | -0.031 | 0.021 |
| rs112147665 | 15 | 59277019 | No | No | - | - | - | - | - | - | - | - | - |
| rs187359403 | 15 | 59780362 | No | No | - | - | - | - | - | - | - | - | - |
| rs10851698 | 15 | 61947694 | Yes | No | T | C | 0.264 | -0.013 | 0 | 0.027 | 0.019 | -0.01 | 0.023 |
| rs4619348 | 15 | 63359564 | Yes | Yes | C | A | 0.565 | 0.016 | 0 | -0.003 | 0.017 | 0.013 | 0.02 |
| rs58633453 | 15 | 63939033 | No | No | - | - | - | - | - | - | - | - | - |
| rs2469128 | 15 | 66877172 | Yes | No | A | G | 0.44 | 0.01 | 0 | -0.026 | 0.017 | 0.023 | 0.02 |
| rs56332871 | 15 | 96714816 | Yes | No | A | C | 0.27 | 0.011 | 0 | 0.021 | 0.02 | 0.036 | 0.024 |
| rs7404696 | 16 | 1012393 | Yes | No | C | T | 0.274 | 0.014 | 0 | -0.014 | 0.019 | 0.014 | 0.022 |
| rs62025680 | 16 | 11464263 | Yes | No | A | G | 0.203 | 0.016 | 0 | -0.019 | 0.021 | 0.015 | 0.025 |
| rs12928099 | 16 | 15150505 | Yes | No | A | C | 0.297 | 0.018 | 0 | 0.03 | 0.019 | 0.027 | 0.022 |
| rs9938120 | 16 | 19977733 | Yes | No | T | C | 0.137 | 0.014 | 0 | -0.016 | 0.026 | -0.037 | 0.031 |
| rs3814883 | 16 | 29994922 | Yes | No | T | C | 0.469 | -0.013 | 0 | -0.017 | 0.017 | 0.014 | 0.02 |
| rs41440449 | 16 | 31337677 | Yes | No | G | A | 0.34 | 0.01 | 0 | 0.014 | 0.018 | 0.005 | 0.021 |
| rs56094641 | 16 | 53806453 | Yes | No | G | A | 0.41 | -0.019 | 0 | 0.004 | 0.017 | 0.009 | 0.02 |
| rs532566544 | 16 | 55793904 | No | No | - | - | - | - | - | - | - | - | - |
| rs560117104 | 16 | 56393734 | No | No | - | - | - | - | - | - | - | - | - |
| rs183130 | 16 | 56991363 | Yes | No | T | C | 0.321 | 0.229 | 0 | 0.02 | 0.018 | -0.009 | 0.021 |
| rs12924311 | 16 | 57551366 | Yes | No | T | C | 0.033 | -0.053 | 0 | 0.034 | 0.038 | -0.054 | 0.046 |
| rs567124999 | 16 | 58191848 | No | No | - | - | - | - | - | - | - | - | - |
| rs117365265 | 16 | 66461927 | Yes | No | A | G | 0.021 | -0.044 | 0 | -0.02 | 0.064 | 0.038 | 0.076 |
| rs55781197 | 16 | 67940350 | Yes | No | G | A | 0.122 | 0.07 | 0 | -0.04 | 0.025 | 0.002 | 0.03 |
| rs571298027 | 16 | 68866671 | No | No | - | - | - | - | - | - | - | - | - |
| rs141904578 | 16 | 69538239 | No | No | - | - | - | - | - | - | - | - | - |
| rs940922629 | 16 | 70587724 | No | No | - | - | - | - | - | - | - | - | - |
| rs745977021 | 16 | 72852502 | No | No | - | - | - | - | - | - | - | - | - |
| rs11149803 | 16 | 75195765 | Yes | No | G | C | 0.311 | 0.011 | 0 | 0.018 | 0.018 | 0.024 | 0.022 |
| rs2925979 | 16 | 81534790 | Yes | Yes | C | T | 0.699 | 0.035 | 0 | 0.006 | 0.018 | 0.009 | 0.022 |
| rs896263 | 16 | 85927519 | Yes | Yes | G | C | 0.241 | 0.013 | 0 | 0.01 | 0.02 | -0.019 | 0.024 |
| rs12931143 | 16 | 88006065 | Yes | No | C | T | 0.478 | -0.012 | 0 | 0.002 | 0.017 | 0.013 | 0.02 |
| rs36049560 | 16 | 88535620 | Yes | No | G | A | 0.31 | -0.013 | 0 | -0.005 | 0.019 | -0.031 | 0.022 |
| rs11870735 | 17 | 481604 | Yes | No | T | C | 0.176 | -0.016 | 0 | 0.03 | 0.023 | -0.06 | 0.027 |
| rs12449758 | 17 | 3884026 | Yes | No | G | A | 0.305 | -0.011 | 0 | -0.001 | 0.018 | -0.016 | 0.022 |
| rs3803800 | 17 | 7462969 | Yes | No | G | A | 0.783 | 0.019 | 0 | -0.026 | 0.02 | -0.005 | 0.024 |
| rs6503091 | 17 | 8109598 | Yes | No | A | G | 0.61 | -0.011 | 0 | -0.014 | 0.017 | -0.008 | 0.021 |
| rs28583584 | 17 | 17483297 | Yes | No | A | G | 0.061 | -0.025 | 0 | -0.037 | 0.035 | -0.004 | 0.042 |
| rs881844 | 17 | 37810218 | Yes | Yes | G | C | 0.663 | 0.03 | 0 | 0.016 | 0.018 | 0.001 | 0.022 |
| rs2586116 | 17 | 38505824 | Yes | No | G | C | 0.253 | -0.013 | 0 | 0.011 | 0.019 | 0.04 | 0.023 |
| rs180705493 | 17 | 40359483 | No | No | - | - | - | - | - | - | - | - | - |
| rs200576810 | 17 | 41166479 | No | No | - | - | - | - | - | - | - | - | - |
| rs72836561 | 17 | 41926126 | Yes | No | T | C | 0.03 | -0.187 | 0 | 0.025 | 0.049 | 0.034 | 0.058 |
| rs72828813 | 17 | 42984085 | Yes | No | T | C | 0.014 | -0.062 | 0 | 0.034 | 0.057 | 0.057 | 0.068 |
| rs4264433 | 17 | 45737275 | No | No | - | - | - | - | - | - | - | - | - |
| rs115271198 | 17 | 46319476 | Yes | No | T | C | 0.071 | 0.028 | 0 | 0.004 | 0.034 | -0.046 | 0.04 |
| rs35895680 | 17 | 47060322 | Yes | No | A | C | 0.314 | 0.012 | 0 | 0 | 0.018 | -0.058 | 0.022 |
| rs12940636 | 17 | 53400110 | Yes | No | C | T | 0.345 | 0.012 | 0 | -0.011 | 0.018 | 0 | 0.021 |
| rs9915591 | 17 | 65826090 | Yes | No | G | C | 0.202 | -0.014 | 0 | 0.024 | 0.021 | -0.005 | 0.025 |
| rs34931250 | 17 | 66879927 | No | No | - | - | - | - | - | - | - | - | - |
| rs7214563 | 17 | 74214276 | Yes | No | T | C | 0.076 | -0.019 | 0 | 0.018 | 0.034 | -0.016 | 0.042 |
| rs2376583 | 17 | 76402105 | No | No | - | - | - | - | - | - | - | - | - |
| rs6506032 | 18 | 287534 | No | No | - | - | - | - | - | - | - | - | - |
| rs1652343 | 18 | 21131929 | Yes | No | C | T | 0.493 | 0.013 | 0 | 0.011 | 0.017 | -0.009 | 0.02 |
| rs144242131 | 18 | 29769910 | No | No | - | - | - | - | - | - | - | - | - |
| rs1518166 | 18 | 40705900 | Yes | No | G | A | 0.379 | 0.011 | 0 | 0.016 | 0.017 | -0.021 | 0.02 |
| rs538969346 | 18 | 46545976 | No | No | - | - | - | - | - | - | - | - | - |
| rs1943977 | 18 | 47173181 | Yes | No | G | A | 0.826 | 0.075 | 0 | 0.055 | 0.022 | -0.009 | 0.026 |
| rs76511906 | 18 | 47698555 | No | No | - | - | - | - | - | - | - | - | - |
| NA | 18 | 48570324 | No | No | - | - | - | - | - | - | - | - | - |
| rs41292412 | 18 | 56118358 | No | No | - | - | - | - | - | - | - | - | - |
| rs35693910 | 18 | 57913965 | Yes | No | A | G | 0.271 | -0.02 | 0 | 0.007 | 0.019 | 0.039 | 0.022 |
| rs12454712 | 18 | 60845884 | No | No | - | - | - | - | - | - | - | - | - |
| rs12975319 | 19 | 3414088 | Yes | Yes | A | G | 0.305 | -0.015 | 0 | -0.015 | 0.018 | 0.014 | 0.022 |
| rs2289863 | 19 | 4028783 | No | No | - | - | - | - | - | - | - | - | - |
| rs10853981 | 19 | 4965064 | Yes | No | A | G | 0.333 | -0.011 | 0 | -0.009 | 0.018 | 0.002 | 0.021 |
| rs112659531 | 19 | 7238475 | Yes | No | C | T | 0.188 | -0.017 | 0 | 0.021 | 0.022 | 0.005 | 0.026 |
| rs116843064 | 19 | 8429323 | Yes | No | A | G | 0.02 | 0.215 | 0 | 0.039 | 0.053 | 0.069 | 0.065 |
| rs555628099 | 19 | 8945880 | No | No | - | - | - | - | - | - | - | - | - |
| rs56322906 | 19 | 11346155 | Yes | No | A | G | 0.039 | -0.079 | 0 | 0.02 | 0.038 | -0.006 | 0.046 |
| rs60570301 | 19 | 18597196 | Yes | No | A | G | 0.121 | 0.016 | 0 | -0.006 | 0.025 | -0.029 | 0.03 |
| rs4805881 | 19 | 33896432 | Yes | No | C | A | 0.666 | 0.02 | 0 | 0.005 | 0.018 | -0.016 | 0.021 |
| rs62122148 | 19 | 35492575 | Yes | No | A | G | 0.318 | 0.011 | 0 | 0.005 | 0.019 | 0.011 | 0.022 |
| rs56254331 | 19 | 41826020 | Yes | No | C | A | 0.163 | 0.016 | 0 | 0.021 | 0.022 | 0.011 | 0.027 |
| rs4760 | 19 | 44153100 | No | No | - | - | - | - | - | - | - | - | - |
| rs141226346 | 19 | 44890969 | No | No | - | - | - | - | - | - | - | - | - |
| rs429358 | 19 | 45411941 | No | No | - | - | - | - | - | - | - | - | - |
| rs34514836 | 19 | 46385438 | Yes | No | C | A | 0.111 | -0.031 | 0 | -0.002 | 0.028 | 0.055 | 0.034 |
| rs12609461 | 19 | 47581464 | Yes | No | C | A | 0.683 | -0.016 | 0 | 0 | 0.018 | 0.047 | 0.022 |
| rs3112494 | 19 | 48105826 | Yes | No | T | C | 0.867 | 0.018 | 0 | 0.025 | 0.024 | -0.052 | 0.029 |
| rs28873836 | 19 | 52314655 | Yes | No | C | G | 0.265 | -0.021 | 0 | -0.001 | 0.019 | -0.014 | 0.023 |
| rs367070 | 19 | 54800500 | Yes | No | G | A | 0.219 | 0.056 | 0 | -0.008 | 0.019 | -0.061 | 0.023 |
| rs151235402 | 20 | 569164 | Yes | No | T | C | 0.017 | -0.068 | 0 | -0.032 | 0.055 | -0.046 | 0.065 |
| rs1054787 | 20 | 17600114 | Yes | No | C | G | 0.156 | -0.018 | 0 | 0.032 | 0.022 | -0.035 | 0.026 |
| rs7266471 | 20 | 30220599 | Yes | No | A | T | 0.164 | 0.015 | 0 | -0.008 | 0.022 | 0.03 | 0.027 |
| rs12624640 | 20 | 32952125 | Yes | Yes | A | G | 0.429 | -0.018 | 0 | -0.004 | 0.017 | 0.002 | 0.02 |
| rs1800961 | 20 | 43042364 | Yes | Yes | T | C | 0.034 | -0.134 | 0 | 0.02 | 0.039 | -0.075 | 0.047 |
| rs2743402 | 20 | 43985204 | Yes | No | C | T | 0.762 | 0.013 | 0 | -0.015 | 0.02 | 0.004 | 0.023 |
| rs6065908 | 20 | 44569930 | Yes | No | T | C | 0.187 | -0.06 | 0 | 0.027 | 0.022 | -0.03 | 0.026 |
| rs55966194 | 20 | 45599090 | Yes | No | G | C | 0.274 | 0.012 | 0 | 0.02 | 0.019 | -0.014 | 0.023 |
| rs4239651 | 20 | 46340596 | Yes | Yes | C | T | 0.797 | 0.025 | 0 | -0.035 | 0.02 | -0.022 | 0.024 |
| rs6096939 | 20 | 51034268 | Yes | No | C | G | 0.362 | 0.011 | 0 | -0.017 | 0.017 | 0 | 0.021 |
| rs6123685 | 20 | 55836040 | No | No | - | - | - | - | - | - | - | - | - |
| rs8126001 | 20 | 62711459 | Yes | No | T | C | 0.484 | 0.014 | 0 | 0.025 | 0.017 | 0.006 | 0.021 |
| rs4818226 | 21 | 42633065 | Yes | No | G | A | 0.677 | -0.011 | 0 | 0.007 | 0.018 | 0.011 | 0.021 |
| rs235314 | 21 | 46271452 | Yes | Yes | T | C | 0.523 | -0.018 | 0 | 0.001 | 0.017 | -0.006 | 0.02 |
| rs9976784 | 21 | 46907479 | Yes | No | A | G | 0.201 | -0.019 | 0 | 0.02 | 0.021 | -0.007 | 0.025 |
| rs2070512 | 22 | 21949411 | Yes | No | C | A | 0.205 | -0.036 | 0 | -0.004 | 0.02 | -0.037 | 0.024 |
| rs9608956 | 22 | 30901592 | Yes | Yes | C | T | 0.251 | -0.017 | 0 | -0.008 | 0.019 | -0.048 | 0.023 |
| rs9610329 | 22 | 36042986 | No | No | - | - | - | - | - | - | - | - | - |
| rs4820325 | 22 | 38599978 | Yes | No | A | G | 0.582 | -0.02 | 0 | 0.001 | 0.017 | 0.015 | 0.021 |
| rs4821815 | 22 | 39105707 | Yes | No | A | G | 0.333 | -0.012 | 0 | -0.033 | 0.018 | -0.011 | 0.022 |
| rs2294915 | 22 | 44340904 | Yes | No | T | C | 0.239 | -0.013 | 0 | 0.012 | 0.02 | 0.004 | 0.024 |
