# Supplementary material for: Impaired glucose tolerance and cardiovascular risk factors in relation to infertility: a Mendelian randomization analysis in the Norwegian Mother, Father, and Child Cohort Study
Source: Hum Reprod. 2023 Nov 8;39(2):436–41. doi: 10.1093/humrep/dead234 (PMC10833082; doi:10.1093/humrep/dead234)
Supplement: dead234_Supplementary_Table_S6 [file dead234_supplementary_table_s6.docx]

**Supplementary Table S6.** SNPs included in triglyceride-related analyses.

| **RSID** | **Chrom.** | **Position** | **Used in MR** | **Used in MR**  **+ Steiger filt.** | **Effect**  **allele** | **Other**  **allele** | **Effect**  **allele**  **freq.** | **Exposure:**  **beta** | **Exposure:**  **SE** | **Outcome**  **(women):**  **beta** | **Outcome**  **(women):**  **SE** | **Outcome**  **(men):**  **beta** | **Outcome**  **(men):**  **SE** |
| --- | --- | --- | --- | --- | --- | --- | --- | --- | --- | --- | --- | --- | --- |
| rs6605083 | 1 | 2147162 | Yes | No | C | T | 0.233 | 0.012 | 0 | 0.024 | 0.021 | 0.013 | 0.025 |
| rs7538833 | 1 | 16504381 | Yes | No | C | T | 0.628 | 0.011 | 0 | 0.013 | 0.018 | -0.005 | 0.021 |
| rs7551124 | 1 | 23785760 | Yes | No | T | C | 0.874 | 0.017 | 0 | 0.002 | 0.024 | 0.008 | 0.029 |
| rs213641 | 1 | 26232356 | Yes | No | A | C | 0.593 | 0.01 | 0 | -0.023 | 0.017 | -0.042 | 0.021 |
| rs114165349 | 1 | 27021913 | Yes | No | C | G | 0.023 | 0.068 | 0 | 0.012 | 0.049 | -0.002 | 0.058 |
| rs4660690 | 1 | 39819503 | Yes | No | A | G | 0.215 | 0.021 | 0 | -0.012 | 0.021 | 0.018 | 0.025 |
| rs61781290 | 1 | 40393160 | Yes | No | G | A | 0.262 | 0.013 | 0 | 0.06 | 0.019 | 0.033 | 0.023 |
| rs12078980 | 1 | 51508526 | Yes | No | C | T | 0.092 | -0.022 | 0 | 0.048 | 0.027 | -0.037 | 0.032 |
| rs1883783 | 1 | 54890956 | Yes | Yes | G | T | 0.581 | 0.014 | 0 | 0 | 0.017 | -0.033 | 0.021 |
| rs188503412 | 1 | 61680217 | No | No | - | - | - | - | - | - | - | - | - |
| rs10889333 | 1 | 62957030 | Yes | Yes | A | G | 0.334 | -0.071 | 0 | 0.018 | 0.018 | 0.006 | 0.021 |
| rs574313788 | 1 | 63679932 | No | No | - | - | - | - | - | - | - | - | - |
| rs2613505 | 1 | 72835410 | Yes | No | T | C | 0.811 | 0.014 | 0 | -0.025 | 0.021 | -0.036 | 0.026 |
| rs165316 | 1 | 91533297 | Yes | No | G | A | 0.2 | -0.013 | 0 | -0.016 | 0.021 | -0.05 | 0.025 |
| rs12133907 | 1 | 93850283 | Yes | No | A | C | 0.609 | 0.012 | 0 | -0.013 | 0.017 | -0.001 | 0.021 |
| rs4378243 | 1 | 98395881 | Yes | No | T | G | 0.826 | -0.015 | 0 | 0.024 | 0.022 | 0.026 | 0.026 |
| rs646776 | 1 | 109818530 | Yes | No | T | C | 0.78 | 0.011 | 0 | -0.002 | 0.02 | -0.015 | 0.024 |
| rs10494363 | 1 | 149909495 | Yes | No | A | G | 0.075 | -0.027 | 0 | 0.006 | 0.03 | -0.013 | 0.037 |
| rs12750321 | 1 | 154251626 | Yes | No | G | A | 0.292 | -0.015 | 0 | 0.016 | 0.019 | -0.025 | 0.022 |
| rs7539464 | 1 | 172359576 | No | No | - | - | - | - | - | - | - | - | - |
| rs115276619 | 1 | 184865132 | Yes | No | A | T | 0.016 | -0.05 | 0 | -0.069 | 0.063 | -0.12 | 0.075 |
| rs2821228 | 1 | 203517723 | No | No | - | - | - | - | - | - | - | - | - |
| rs11240358 | 1 | 205070573 | Yes | Yes | A | G | 0.397 | 0.013 | 0 | -0.011 | 0.017 | -0.001 | 0.021 |
| rs1337101 | 1 | 219726100 | Yes | No | T | G | 0.313 | -0.02 | 0 | -0.019 | 0.018 | 0.011 | 0.021 |
| rs2642438 | 1 | 220970028 | Yes | No | G | A | 0.706 | -0.016 | 0 | -0.006 | 0.019 | 0.008 | 0.022 |
| rs2281721 | 1 | 230297136 | Yes | No | T | C | 0.61 | -0.044 | 0 | -0.004 | 0.017 | -0.024 | 0.021 |
| rs75054794 | 1 | 230823363 | Yes | No | G | A | 0.052 | 0.023 | 0 | -0.076 | 0.037 | 0.035 | 0.044 |
| rs17713879 | 2 | 254215 | Yes | No | A | G | 0.368 | -0.011 | 0 | -0.008 | 0.017 | 0.025 | 0.02 |
| rs3820897 | 2 | 3642361 | Yes | No | C | T | 0.807 | 0.016 | 0 | 0.012 | 0.021 | 0.022 | 0.025 |
| rs12478327 | 2 | 20374249 | Yes | No | C | A | 0.487 | -0.017 | 0 | -0.018 | 0.017 | 0.037 | 0.02 |
| rs676210 | 2 | 21231524 | Yes | No | A | G | 0.214 | -0.07 | 0 | 0.005 | 0.021 | -0.061 | 0.025 |
| rs62128360 | 2 | 26309420 | Yes | No | G | T | 0.07 | 0.021 | 0 | -0.003 | 0.034 | 0.019 | 0.041 |
| rs57035494 | 2 | 26924018 | Yes | No | G | C | 0.038 | -0.038 | 0 | 0.027 | 0.042 | 0.111 | 0.05 |
| rs1260326 | 2 | 27730940 | Yes | No | C | T | 0.609 | -0.111 | 0 | 0.001 | 0.018 | 0.003 | 0.021 |
| rs72780006 | 2 | 28599749 | No | No | - | - | - | - | - | - | - | - | - |
| rs72616903 | 2 | 43743012 | Yes | No | C | T | 0.165 | 0.015 | 0 | -0.002 | 0.023 | 0.009 | 0.028 |
| rs17326656 | 2 | 48962291 | Yes | No | T | G | 0.231 | 0.013 | 0 | 0.041 | 0.02 | 0.039 | 0.024 |
| rs1861410 | 2 | 58933591 | Yes | No | T | C | 0.551 | -0.011 | 0 | -0.016 | 0.017 | 0.017 | 0.02 |
| rs3087898 | 2 | 61765074 | Yes | No | A | G | 0.424 | -0.01 | 0 | -0.012 | 0.017 | 0.011 | 0.02 |
| rs2540949 | 2 | 65284231 | Yes | No | T | A | 0.383 | -0.02 | 0 | 0.001 | 0.017 | -0.011 | 0.021 |
| rs939136 | 2 | 66677531 | No | No | - | - | - | - | - | - | - | - | - |
| rs6708784 | 2 | 111927379 | Yes | Yes | G | A | 0.496 | -0.013 | 0 | -0.001 | 0.017 | -0.018 | 0.02 |
| rs13409360 | 2 | 113838102 | Yes | No | A | G | 0.398 | -0.01 | 0 | 0.007 | 0.017 | 0.008 | 0.021 |
| rs78198921 | 2 | 119749146 | Yes | No | G | C | 0.014 | 0.044 | 0 | -0.039 | 0.068 | 0.031 | 0.08 |
| rs7578604 | 2 | 121308660 | Yes | No | T | G | 0.248 | 0.016 | 0 | 0.007 | 0.019 | -0.005 | 0.023 |
| rs6430090 | 2 | 146347459 | Yes | No | G | A | 0.598 | -0.01 | 0 | 0.029 | 0.017 | -0.009 | 0.021 |
| rs6743795 | 2 | 161122134 | Yes | No | G | A | 0.723 | 0.011 | 0 | 0.021 | 0.019 | 0.012 | 0.022 |
| rs13389219 | 2 | 165528876 | Yes | No | T | C | 0.4 | -0.034 | 0 | -0.011 | 0.017 | -0.008 | 0.02 |
| rs12472667 | 2 | 171629063 | Yes | No | G | C | 0.361 | 0.01 | 0 | -0.001 | 0.017 | -0.001 | 0.021 |
| rs148566631 | 2 | 203447875 | Yes | No | A | G | 0.117 | 0.018 | 0 | -0.004 | 0.027 | 0.039 | 0.032 |
| rs78058190 | 2 | 219699999 | No | No | - | - | - | - | - | - | - | - | - |
| rs1024137 | 2 | 227095876 | Yes | No | T | G | 0.637 | 0.034 | 0 | 0.001 | 0.017 | 0.032 | 0.021 |
| rs79639690 | 2 | 227605895 | Yes | No | C | T | 0.046 | 0.024 | 0 | -0.088 | 0.044 | 0.078 | 0.053 |
| rs9812100 | 3 | 4763301 | Yes | No | A | G | 0.471 | -0.012 | 0 | -0.027 | 0.017 | -0.014 | 0.02 |
| rs62246311 | 3 | 9498143 | Yes | No | A | G | 0.102 | 0.016 | 0 | 0.043 | 0.027 | 0.024 | 0.031 |
| rs1801282 | 3 | 12393125 | Yes | No | G | C | 0.125 | -0.027 | 0 | -0.044 | 0.024 | -0.046 | 0.029 |
| rs6792725 | 3 | 24520283 | No | No | - | - | - | - | - | - | - | - | - |
| rs144323903 | 3 | 36981990 | No | No | - | - | - | - | - | - | - | - | - |
| rs4955411 | 3 | 49145304 | No | No | - | - | - | - | - | - | - | - | - |
| rs17052061 | 3 | 52346240 | Yes | No | G | T | 0.169 | -0.02 | 0 | -0.016 | 0.023 | -0.03 | 0.028 |
| rs12486424 | 3 | 66874481 | Yes | No | C | A | 0.289 | 0.012 | 0 | 0.004 | 0.019 | 0.009 | 0.022 |
| rs13066793 | 3 | 87037543 | No | No | - | - | - | - | - | - | - | - | - |
| rs684773 | 3 | 135956305 | Yes | No | C | A | 0.791 | 0.025 | 0 | 0.047 | 0.02 | 0.037 | 0.024 |
| rs111834982 | 3 | 142019751 | Yes | No | G | A | 0.129 | -0.014 | 0 | 0.012 | 0.025 | 0.052 | 0.029 |
| rs9653945 | 3 | 142660706 | Yes | No | A | G | 0.347 | -0.013 | 0 | -0.016 | 0.018 | -0.027 | 0.021 |
| rs62271373 | 3 | 150066540 | Yes | No | A | T | 0.053 | 0.035 | 0 | 0.056 | 0.04 | -0.091 | 0.047 |
| rs6787781 | 3 | 155535351 | Yes | No | C | G | 0.27 | 0.013 | 0 | -0.018 | 0.019 | 0.025 | 0.023 |
| rs9817452 | 3 | 156795414 | Yes | No | T | G | 0.383 | -0.018 | 0 | 0.006 | 0.017 | -0.021 | 0.021 |
| rs11924648 | 3 | 170717996 | Yes | No | G | A | 0.128 | 0.02 | 0 | 0.017 | 0.025 | 0.009 | 0.03 |
| rs79287178 | 3 | 172294500 | Yes | No | A | G | 0.029 | 0.06 | 0 | -0.058 | 0.059 | 0.073 | 0.068 |
| rs34311866 | 4 | 951947 | Yes | No | C | T | 0.182 | 0.017 | 0 | 0.009 | 0.022 | -0.028 | 0.026 |
| rs3775067 | 4 | 2888622 | Yes | Yes | A | G | 0.386 | 0.014 | 0 | 0.011 | 0.017 | -0.003 | 0.021 |
| rs13108218 | 4 | 3443931 | Yes | No | G | A | 0.614 | -0.029 | 0 | -0.009 | 0.017 | -0.027 | 0.021 |
| rs4450871 | 4 | 4990298 | No | No | - | - | - | - | - | - | - | - | - |
| rs9942171 | 4 | 18032487 | Yes | No | A | G | 0.13 | 0.021 | 0 | 0.03 | 0.027 | -0.043 | 0.032 |
| rs6448429 | 4 | 26066863 | Yes | No | T | C | 0.164 | 0.022 | 0 | 0.024 | 0.024 | -0.002 | 0.029 |
| rs35484700 | 4 | 55498781 | No | No | - | - | - | - | - | - | - | - | - |
| rs9884390 | 4 | 69373407 | Yes | No | C | T | 0.223 | 0.017 | 0 | 0.008 | 0.02 | -0.01 | 0.024 |
| rs573930512 | 4 | 74512280 | No | No | - | - | - | - | - | - | - | - | - |
| rs10008637 | 4 | 77414144 | Yes | No | C | T | 0.456 | 0.01 | 0 | 0.013 | 0.017 | -0.013 | 0.02 |
| rs17449582 | 4 | 87240157 | Yes | No | T | C | 0.358 | 0.016 | 0 | 0.03 | 0.018 | 0.001 | 0.021 |
| rs17605615 | 4 | 87996745 | Yes | Yes | A | G | 0.394 | 0.027 | 0 | -0.007 | 0.017 | 0.031 | 0.021 |
| rs2167750 | 4 | 89730074 | Yes | No | T | C | 0.465 | 0.012 | 0 | -0.005 | 0.017 | -0.034 | 0.02 |
| rs1126673 | 4 | 100045616 | Yes | No | T | C | 0.694 | 0.014 | 0 | -0.023 | 0.019 | -0.011 | 0.022 |
| rs13107325 | 4 | 103188709 | Yes | No | T | C | 0.064 | 0.037 | 0 | -0.037 | 0.04 | -0.011 | 0.048 |
| rs10017313 | 4 | 103861380 | Yes | No | G | A | 0.486 | -0.01 | 0 | 0.027 | 0.017 | 0.03 | 0.02 |
| rs138307849 | 4 | 110095620 | No | No | - | - | - | - | - | - | - | - | - |
| rs114816312 | 4 | 110638824 | No | No | - | - | - | - | - | - | - | - | - |
| rs192929239 | 4 | 124641827 | Yes | No | G | A | 0.013 | 0.046 | 0 | 0.087 | 0.072 | -0.169 | 0.087 |
| rs7696969 | 4 | 143326714 | Yes | No | G | T | 0.632 | -0.012 | 0 | -0.007 | 0.018 | -0.015 | 0.021 |
| rs6054 | 4 | 155489608 | No | No | - | - | - | - | - | - | - | - | - |
| rs6822892 | 4 | 157734675 | Yes | No | G | A | 0.33 | -0.014 | 0 | -0.028 | 0.018 | -0.033 | 0.021 |
| rs79760705 | 5 | 53298716 | Yes | No | T | G | 0.11 | 0.026 | 0 | 0.025 | 0.026 | 0.002 | 0.032 |
| rs28650790 | 5 | 55861464 | Yes | No | T | C | 0.181 | 0.038 | 0 | 0.031 | 0.023 | -0.043 | 0.028 |
| rs151912 | 5 | 57607142 | No | No | - | - | - | - | - | - | - | - | - |
| rs4976033 | 5 | 67714246 | Yes | No | G | A | 0.406 | 0.018 | 0 | 0.007 | 0.017 | -0.025 | 0.02 |
| rs10052346 | 5 | 78472599 | Yes | No | T | G | 0.39 | -0.011 | 0 | 0.011 | 0.017 | -0.017 | 0.021 |
| rs115912456 | 5 | 82815158 | Yes | No | G | A | 0.042 | -0.027 | 0 | -0.023 | 0.043 | 0.047 | 0.052 |
| rs6595187 | 5 | 118723609 | Yes | No | G | A | 0.283 | -0.019 | 0 | -0.022 | 0.019 | 0.005 | 0.023 |
| rs17764730 | 5 | 127357526 | Yes | No | T | C | 0.232 | -0.011 | 0 | 0.004 | 0.02 | -0.05 | 0.024 |
| rs253942 | 5 | 131320462 | Yes | No | T | C | 0.062 | 0.025 | 0 | -0.072 | 0.034 | -0.1 | 0.04 |
| rs72801474 | 5 | 132444128 | Yes | No | A | G | 0.095 | -0.03 | 0 | -0.044 | 0.027 | 0.069 | 0.032 |
| rs111998037 | 5 | 139798007 | Yes | No | C | A | 0.257 | -0.011 | 0 | 0.007 | 0.019 | -0.013 | 0.022 |
| rs12523418 | 5 | 141707225 | Yes | No | G | A | 0.32 | -0.01 | 0 | -0.018 | 0.018 | -0.014 | 0.022 |
| rs245078 | 5 | 149353598 | Yes | No | G | A | 0.406 | -0.01 | 0 | -0.007 | 0.017 | -0.005 | 0.021 |
| rs6882076 | 5 | 156390297 | Yes | No | C | T | 0.635 | 0.034 | 0 | -0.016 | 0.018 | 0.01 | 0.021 |
| rs2914231 | 5 | 158011435 | Yes | No | C | G | 0.23 | 0.022 | 0 | 0.008 | 0.02 | 0.005 | 0.024 |
| rs72812818 | 5 | 173356752 | Yes | No | C | G | 0.311 | 0.011 | 0 | -0.024 | 0.018 | 0.012 | 0.021 |
| rs62397245 | 5 | 176750688 | Yes | No | G | C | 0.231 | 0.015 | 0 | -0.008 | 0.02 | -0.008 | 0.024 |
| rs6924805 | 6 | 18747705 | Yes | No | T | G | 0.592 | -0.01 | 0 | -0.024 | 0.017 | -0.003 | 0.021 |
| rs7451008 | 6 | 20673880 | Yes | No | C | T | 0.267 | 0.012 | 0 | 0.008 | 0.019 | -0.022 | 0.023 |
| rs9379828 | 6 | 26167951 | Yes | No | G | C | 0.355 | -0.01 | 0 | 0 | 0.018 | -0.036 | 0.021 |
| rs56114371 | 6 | 27274834 | No | No | - | - | - | - | - | - | - | - | - |
| rs3131337 | 6 | 28796071 | No | No | - | - | - | - | - | - | - | - | - |
| rs9258375 | 6 | 29752808 | Yes | No | G | A | 0.102 | -0.022 | 0 | 0.001 | 0.027 | -0.035 | 0.032 |
| rs3094034 | 6 | 30363351 | No | No | - | - | - | - | - | - | - | - | - |
| rs9468937 | 6 | 31270118 | Yes | No | A | C | 0.435 | 0.028 | 0 | 0.024 | 0.017 | 0.015 | 0.02 |
| rs622871 | 6 | 31878495 | Yes | No | G | A | 0.703 | 0.028 | 0 | -0.009 | 0.018 | 0.037 | 0.022 |
| rs9461755 | 6 | 32441046 | Yes | No | A | G | 0.046 | 0.076 | 0 | -0.033 | 0.036 | 0.068 | 0.044 |
| rs144607208 | 6 | 32947516 | Yes | No | G | A | 0.023 | 0.065 | 0 | -0.081 | 0.053 | 0.066 | 0.064 |
| rs75507056 | 6 | 33457187 | Yes | No | T | C | 0.016 | 0.056 | 0 | -0.053 | 0.066 | -0.037 | 0.081 |
| rs6934083 | 6 | 35237153 | Yes | No | T | C | 0.034 | 0.026 | 0 | 0.004 | 0.05 | 0.061 | 0.06 |
| rs3176320 | 6 | 36646788 | Yes | No | G | A | 0.339 | -0.01 | 0 | -0.01 | 0.018 | 0.012 | 0.021 |
| rs742493 | 6 | 40998167 | Yes | No | C | T | 0.116 | -0.017 | 0 | 0.002 | 0.025 | 0.033 | 0.03 |
| rs7757363 | 6 | 42876386 | Yes | Yes | T | C | 0.309 | 0.01 | 0 | 0.04 | 0.019 | 0.001 | 0.022 |
| rs998584 | 6 | 43757896 | Yes | No | A | C | 0.479 | 0.036 | 0 | 0.01 | 0.017 | -0.008 | 0.02 |
| rs2749005 | 6 | 52621433 | Yes | No | G | T | 0.648 | -0.019 | 0 | 0.012 | 0.018 | -0.006 | 0.021 |
| rs398085093 | 6 | 86296925 | No | No | - | - | - | - | - | - | - | - | - |
| rs9496567 | 6 | 100602753 | Yes | No | A | G | 0.236 | -0.012 | 0 | -0.038 | 0.02 | -0.002 | 0.024 |
| rs6913325 | 6 | 106378009 | Yes | No | T | G | 0.441 | -0.01 | 0 | -0.017 | 0.017 | -0.028 | 0.02 |
| rs35045014 | 6 | 107432157 | Yes | No | A | C | 0.462 | 0.014 | 0 | 0.042 | 0.017 | 0.016 | 0.02 |
| rs9480889 | 6 | 109189021 | Yes | No | G | C | 0.788 | 0.015 | 0 | -0.006 | 0.021 | -0.004 | 0.025 |
| rs577721086 | 6 | 127440047 | Yes | No | C | T | 0.052 | 0.053 | 0 | -0.065 | 0.035 | -0.025 | 0.042 |
| rs7740188 | 6 | 130346105 | Yes | No | A | G | 0.705 | 0.014 | 0 | 0.007 | 0.018 | -0.004 | 0.022 |
| rs672457 | 6 | 139836562 | Yes | No | A | G | 0.582 | -0.026 | 0 | -0.02 | 0.017 | 0.007 | 0.02 |
| rs9321765 | 6 | 140595744 | No | No | - | - | - | - | - | - | - | - | - |
| rs607335 | 6 | 153465230 | Yes | No | A | C | 0.412 | -0.01 | 0 | -0.007 | 0.017 | -0.042 | 0.02 |
| rs78425119 | 6 | 160501825 | Yes | No | A | G | 0.066 | 0.035 | 0 | -0.013 | 0.032 | 0.067 | 0.039 |
| rs41272086 | 6 | 161008646 | Yes | No | A | G | 0.102 | 0.039 | 0 | -0.043 | 0.025 | -0.004 | 0.03 |
| rs1464780 | 6 | 161520837 | Yes | No | A | G | 0.945 | 0.023 | 0 | 0.005 | 0.035 | 0.097 | 0.041 |
| rs73029263 | 6 | 164113762 | Yes | No | G | A | 0.13 | -0.019 | 0 | -0.017 | 0.025 | -0.029 | 0.03 |
| rs5011439 | 7 | 12268811 | No | No | - | - | - | - | - | - | - | - | - |
| rs38205 | 7 | 15913588 | Yes | Yes | C | A | 0.624 | -0.013 | 0 | -0.009 | 0.018 | -0.022 | 0.021 |
| rs4410790 | 7 | 17284577 | Yes | No | C | T | 0.632 | 0.013 | 0 | -0.01 | 0.018 | 0.009 | 0.021 |
| rs4142995 | 7 | 17919258 | Yes | No | T | G | 0.392 | 0.013 | 0 | -0.011 | 0.017 | -0.02 | 0.021 |
| rs55696093 | 7 | 21605973 | Yes | No | G | A | 0.21 | 0.012 | 0 | 0.02 | 0.02 | -0.016 | 0.024 |
| rs4722551 | 7 | 25991826 | Yes | No | C | T | 0.163 | -0.028 | 0 | 0.02 | 0.023 | 0.056 | 0.027 |
| rs849336 | 7 | 28224053 | Yes | No | G | A | 0.649 | -0.01 | 0 | 0.033 | 0.018 | 0.026 | 0.021 |
| rs2070971 | 7 | 44197583 | Yes | Yes | T | G | 0.14 | 0.029 | 0 | -0.016 | 0.024 | -0.035 | 0.029 |
| rs71551223 | 7 | 71256489 | Yes | No | C | A | 0.03 | -0.037 | 0 | -0.018 | 0.046 | 0.01 | 0.055 |
| rs36104871 | 7 | 71808047 | Yes | No | T | G | 0.029 | -0.058 | 0 | 0.048 | 0.046 | 0.092 | 0.056 |
| rs35753501 | 7 | 72330303 | Yes | No | T | C | 0.026 | -0.094 | 0 | 0.022 | 0.047 | -0.014 | 0.056 |
| rs3812316 | 7 | 73020337 | Yes | No | G | C | 0.124 | -0.122 | 0 | 0.006 | 0.025 | -0.066 | 0.03 |
| rs3135688 | 7 | 73651743 | Yes | Yes | C | T | 0.04 | -0.03 | 0 | 0.018 | 0.037 | -0.005 | 0.044 |
| rs1057868 | 7 | 75615006 | Yes | No | T | C | 0.288 | 0.012 | 0 | 0.004 | 0.018 | -0.015 | 0.022 |
| rs1229499 | 7 | 81573045 | Yes | Yes | A | T | 0.706 | -0.011 | 0 | 0.004 | 0.019 | 0.002 | 0.023 |
| rs2283038 | 7 | 106835410 | Yes | No | T | C | 0.235 | -0.011 | 0 | -0.032 | 0.02 | -0.007 | 0.024 |
| rs10215153 | 7 | 116399131 | Yes | Yes | A | G | 0.311 | 0.012 | 0 | -0.016 | 0.018 | -0.022 | 0.022 |
| rs12534129 | 7 | 117018135 | Yes | No | C | T | 0.392 | 0.012 | 0 | 0.017 | 0.017 | -0.04 | 0.02 |
| rs4731702 | 7 | 130433384 | Yes | No | T | C | 0.495 | -0.025 | 0 | -0.006 | 0.017 | 0.029 | 0.02 |
| rs7808446 | 7 | 150214465 | Yes | No | G | T | 0.754 | -0.016 | 0 | -0.016 | 0.019 | 0.041 | 0.023 |
| rs2921060 | 8 | 8317817 | Yes | No | C | A | 0.458 | -0.016 | 0 | 0.002 | 0.017 | -0.027 | 0.02 |
| rs17149279 | 8 | 9195638 | Yes | No | T | C | 0.231 | -0.019 | 0 | -0.014 | 0.02 | -0.01 | 0.023 |
| rs615632 | 8 | 9796321 | Yes | No | T | C | 0.525 | 0.015 | 0 | 0.009 | 0.017 | 0.024 | 0.02 |
| rs7821812 | 8 | 10644101 | Yes | No | C | G | 0.205 | 0.028 | 0 | 0.016 | 0.021 | 0.045 | 0.025 |
| rs4320509 | 8 | 11528339 | Yes | No | C | G | 0.127 | 0.032 | 0 | 0.034 | 0.028 | 0.023 | 0.033 |
| rs28415552 | 8 | 13511219 | Yes | No | A | C | 0.631 | -0.01 | 0 | -0.02 | 0.018 | -0.003 | 0.021 |
| rs1968041 | 8 | 17616562 | Yes | No | T | C | 0.358 | -0.01 | 0 | -0.03 | 0.018 | 0.005 | 0.021 |
| rs35246381 | 8 | 18272535 | Yes | No | T | C | 0.778 | -0.034 | 0 | 0.025 | 0.02 | -0.001 | 0.024 |
| rs2958557 | 8 | 19251679 | No | No | - | - | - | - | - | - | - | - | - |
| rs328 | 8 | 19819724 | Yes | No | G | C | 0.1 | -0.18 | 0 | 0.007 | 0.028 | 0.021 | 0.033 |
| rs2616213 | 8 | 20610866 | Yes | No | C | T | 0.544 | -0.01 | 0 | -0.012 | 0.017 | 0.004 | 0.02 |
| rs56193509 | 8 | 23525075 | Yes | No | A | T | 0.244 | 0.011 | 0 | 0.011 | 0.019 | 0.031 | 0.023 |
| rs73221948 | 8 | 25464670 | No | No | - | - | - | - | - | - | - | - | - |
| rs7007256 | 8 | 26203081 | Yes | No | A | G | 0.712 | 0.01 | 0 | -0.004 | 0.018 | 0.02 | 0.022 |
| rs7816345 | 8 | 36846109 | Yes | No | T | C | 0.169 | 0.014 | 0 | 0.022 | 0.024 | -0.027 | 0.029 |
| rs4647906 | 8 | 38325411 | Yes | No | A | G | 0.402 | 0.013 | 0 | 0.004 | 0.017 | 0.017 | 0.02 |
| rs2081687 | 8 | 59388565 | Yes | No | C | T | 0.659 | -0.023 | 0 | 0.001 | 0.018 | 0.016 | 0.022 |
| rs745578 | 8 | 72466324 | Yes | Yes | A | G | 0.245 | 0.017 | 0 | 0.004 | 0.019 | -0.009 | 0.023 |
| rs7830852 | 8 | 106415088 | Yes | No | G | A | 0.226 | 0.012 | 0 | 0.011 | 0.02 | 0.057 | 0.025 |
| rs2737246 | 8 | 116659578 | Yes | No | C | G | 0.277 | -0.012 | 0 | 0.031 | 0.018 | 0.026 | 0.022 |
| rs28601761 | 8 | 126500031 | No | No | - | - | - | - | - | - | - | - | - |
| rs1561929 | 8 | 129567373 | Yes | Yes | T | C | 0.883 | 0.016 | 0 | 0.009 | 0.026 | 0.047 | 0.032 |
| rs7832515 | 8 | 144306379 | Yes | No | G | A | 0.197 | -0.014 | 0 | -0.014 | 0.02 | -0.044 | 0.024 |
| rs7856817 | 9 | 1054587 | No | No | - | - | - | - | - | - | - | - | - |
| rs1658970 | 9 | 6664853 | Yes | Yes | A | C | 0.139 | 0.014 | 0 | 0.04 | 0.025 | -0.014 | 0.03 |
| rs7041117 | 9 | 13679856 | Yes | No | T | A | 0.583 | -0.011 | 0 | -0.009 | 0.017 | -0.033 | 0.021 |
| rs686030 | 9 | 15304782 | Yes | No | A | C | 0.859 | 0.017 | 0 | -0.031 | 0.024 | 0.004 | 0.028 |
| rs62543565 | 9 | 16901067 | Yes | No | A | C | 0.637 | -0.016 | 0 | -0.03 | 0.017 | -0.02 | 0.021 |
| rs2383766 | 9 | 28412500 | Yes | No | T | G | 0.319 | 0.01 | 0 | -0.009 | 0.018 | 0.022 | 0.022 |
| rs10971957 | 9 | 34151463 | Yes | No | T | C | 0.598 | -0.011 | 0 | -0.031 | 0.017 | -0.009 | 0.02 |
| rs296883 | 9 | 86578925 | Yes | No | T | A | 0.264 | -0.011 | 0 | -0.008 | 0.019 | 0.035 | 0.023 |
| rs17055001 | 9 | 92195246 | Yes | No | A | G | 0.311 | 0.012 | 0 | -0.008 | 0.018 | 0.003 | 0.022 |
| rs1800978 | 9 | 107665978 | Yes | No | G | C | 0.123 | -0.025 | 0 | -0.004 | 0.026 | 0.042 | 0.031 |
| rs11789974 | 9 | 110488875 | Yes | No | A | C | 0.032 | -0.029 | 0 | 0.004 | 0.041 | -0.015 | 0.048 |
| rs75113691 | 9 | 112245773 | No | No | - | - | - | - | - | - | - | - | - |
| rs150611042 | 9 | 117083803 | Yes | No | A | C | 0.069 | -0.02 | 0 | -0.069 | 0.038 | -0.002 | 0.045 |
| rs2416797 | 9 | 123480600 | Yes | No | A | C | 0.699 | 0.011 | 0 | -0.009 | 0.018 | -0.007 | 0.022 |
| rs142122182 | 10 | 5263341 | Yes | No | G | A | 0.155 | -0.021 | 0 | -0.015 | 0.022 | -0.023 | 0.026 |
| rs4934927 | 10 | 33628062 | Yes | No | C | G | 0.09 | 0.018 | 0 | -0.022 | 0.03 | -0.053 | 0.035 |
| rs41274050 | 10 | 52573772 | No | No | - | - | - | - | - | - | - | - | - |
| rs1171616 | 10 | 61468589 | Yes | Yes | T | G | 0.775 | 0.016 | 0 | -0.005 | 0.02 | -0.016 | 0.024 |
| rs35706909 | 10 | 63922233 | Yes | No | C | T | 0.099 | -0.018 | 0 | 0.041 | 0.027 | 0.005 | 0.033 |
| rs7924036 | 10 | 65191645 | Yes | No | T | G | 0.512 | -0.029 | 0 | -0.052 | 0.017 | 0.013 | 0.02 |
| rs7904738 | 10 | 74710484 | Yes | No | C | G | 0.086 | 0.024 | 0 | 0.007 | 0.029 | -0.061 | 0.034 |
| rs149590572 | 10 | 93575831 | Yes | No | A | G | 0.064 | 0.025 | 0 | 0.038 | 0.033 | -0.014 | 0.04 |
| rs17875327 | 10 | 94274809 | Yes | No | G | A | 0.111 | 0.024 | 0 | -0.013 | 0.025 | -0.024 | 0.03 |
| rs2068888 | 10 | 94839642 | Yes | Yes | A | G | 0.451 | -0.029 | 0 | -0.003 | 0.017 | -0.001 | 0.02 |
| rs7091593 | 10 | 95342772 | Yes | No | T | A | 0.113 | 0.018 | 0 | -0.022 | 0.027 | 0.024 | 0.033 |
| rs3891783 | 10 | 96015793 | No | No | - | - | - | - | - | - | - | - | - |
| rs1408579 | 10 | 101912194 | Yes | No | T | C | 0.482 | 0.01 | 0 | 0.012 | 0.017 | -0.008 | 0.02 |
| rs75398587 | 10 | 103946480 | Yes | No | G | C | 0.062 | -0.027 | 0 | 0.029 | 0.036 | 0.032 | 0.043 |
| rs2803619 | 10 | 113934384 | Yes | No | C | G | 0.718 | 0.018 | 0 | 0.027 | 0.019 | 0.005 | 0.023 |
| rs2484294 | 10 | 115792062 | Yes | No | A | G | 0.737 | -0.011 | 0 | -0.005 | 0.019 | -0.005 | 0.023 |
| rs12772930 | 10 | 120493295 | Yes | No | A | G | 0.353 | 0.01 | 0 | -0.005 | 0.018 | 0.02 | 0.021 |
| rs1873449 | 10 | 122931652 | Yes | No | T | G | 0.022 | -0.037 | 0 | -0.047 | 0.057 | -0.083 | 0.069 |
| rs1133400 | 10 | 134459388 | Yes | No | G | A | 0.213 | 0.013 | 0 | -0.009 | 0.02 | 0.036 | 0.025 |
| rs537132753 | 10 | 135440683 | No | No | - | - | - | - | - | - | - | - | - |
| rs117739035 | 11 | 408174 | Yes | No | T | G | 0.036 | 0.025 | 0 | -0.031 | 0.038 | -0.053 | 0.046 |
| rs4909945 | 11 | 10673739 | Yes | No | C | T | 0.68 | 0.013 | 0 | 0.027 | 0.018 | -0.003 | 0.021 |
| rs1037169 | 11 | 13361005 | Yes | No | C | T | 0.683 | 0.018 | 0 | 0 | 0.018 | -0.021 | 0.021 |
| rs150090666 | 11 | 14865399 | No | No | - | - | - | - | - | - | - | - | - |
| rs142108391 | 11 | 26216166 | Yes | No | A | G | 0.063 | 0.021 | 0 | -0.024 | 0.035 | 0.041 | 0.042 |
| rs1519480 | 11 | 27675712 | Yes | No | T | C | 0.679 | -0.015 | 0 | -0.023 | 0.018 | -0.008 | 0.021 |
| rs75160368 | 11 | 30402714 | Yes | No | A | G | 0.088 | -0.017 | 0 | -0.05 | 0.031 | 0.036 | 0.037 |
| rs117847116 | 11 | 46048240 | Yes | No | C | T | 0.04 | -0.027 | 0 | 0 | 0.045 | 0.058 | 0.055 |
| rs3758669 | 11 | 47272579 | Yes | No | G | A | 0.31 | -0.023 | 0 | -0.028 | 0.018 | -0.017 | 0.022 |
| rs541958245 | 11 | 56385221 | No | No | - | - | - | - | - | - | - | - | - |
| rs174562 | 11 | 61585144 | Yes | No | G | A | 0.346 | 0.052 | 0 | 0.002 | 0.018 | 0.009 | 0.021 |
| rs11231161 | 11 | 62378221 | Yes | Yes | G | A | 0.358 | 0.011 | 0 | -0.02 | 0.017 | -0.037 | 0.021 |
| rs56271783 | 11 | 64004723 | Yes | No | C | G | 0.047 | 0.053 | 0 | -0.064 | 0.038 | -0.06 | 0.044 |
| rs10750766 | 11 | 65473798 | Yes | No | A | C | 0.71 | 0.017 | 0 | -0.025 | 0.019 | 0.014 | 0.022 |
| rs10896373 | 11 | 68614810 | No | No | - | - | - | - | - | - | - | - | - |
| rs10796821 | 11 | 69305417 | Yes | No | G | A | 0.513 | 0.01 | 0 | 0.026 | 0.017 | 0.003 | 0.02 |
| rs2063724 | 11 | 78133077 | Yes | No | C | T | 0.173 | -0.014 | 0 | 0.022 | 0.023 | 0.033 | 0.028 |
| rs759831202 | 11 | 115554537 | No | No | - | - | - | - | - | - | - | - | - |
| rs117288140 | 11 | 116139965 | Yes | No | T | C | 0.017 | 0.04 | 0 | 0.123 | 0.058 | -0.165 | 0.069 |
| rs964184 | 11 | 116648917 | Yes | No | C | G | 0.865 | -0.246 | 0 | 0.033 | 0.025 | 0.003 | 0.03 |
| rs192489212 | 11 | 117220429 | No | No | - | - | - | - | - | - | - | - | - |
| rs747437837 | 11 | 117849058 | No | No | - | - | - | - | - | - | - | - | - |
| rs749804194 | 11 | 118574327 | No | No | - | - | - | - | - | - | - | - | - |
| rs76895963 | 12 | 4384844 | No | No | - | - | - | - | - | - | - | - | - |
| rs73047887 | 12 | 6736843 | Yes | No | C | T | 0.255 | 0.011 | 0 | 0.032 | 0.02 | 0.012 | 0.023 |
| rs11045171 | 12 | 20470199 | Yes | No | G | A | 0.2 | -0.02 | 0 | 0.004 | 0.021 | -0.009 | 0.025 |
| rs4149056 | 12 | 21331549 | Yes | Yes | C | T | 0.158 | 0.027 | 0 | -0.013 | 0.023 | 0.029 | 0.027 |
| rs11046481 | 12 | 22747967 | Yes | Yes | G | T | 0.235 | -0.015 | 0 | -0.015 | 0.02 | 0.042 | 0.024 |
| rs17389465 | 12 | 25414408 | Yes | No | A | T | 0.069 | -0.02 | 0 | -0.026 | 0.034 | -0.059 | 0.041 |
| rs4963975 | 12 | 26443030 | Yes | No | A | G | 0.25 | 0.015 | 0 | 0.008 | 0.019 | 0.036 | 0.022 |
| rs10082956 | 12 | 29504556 | Yes | No | G | A | 0.29 | -0.013 | 0 | -0.018 | 0.019 | -0.001 | 0.022 |
| rs11183212 | 12 | 46213867 | Yes | Yes | G | A | 0.202 | 0.016 | 0 | -0.01 | 0.021 | 0.021 | 0.025 |
| rs145878042 | 12 | 48143315 | Yes | No | G | A | 0.011 | 0.046 | 0 | -0.16 | 0.076 | 0.047 | 0.09 |
| rs1126930 | 12 | 49399132 | Yes | No | C | G | 0.034 | 0.028 | 0 | 0.015 | 0.047 | -0.112 | 0.055 |
| rs7312441 | 12 | 56941146 | Yes | No | A | C | 0.345 | -0.011 | 0 | -0.005 | 0.017 | 0.023 | 0.021 |
| rs79395356 | 12 | 57738600 | Yes | No | G | T | 0.248 | -0.025 | 0 | 0.027 | 0.019 | 0.034 | 0.022 |
| rs8756 | 12 | 66359752 | Yes | No | A | C | 0.509 | 0.01 | 0 | -0.02 | 0.017 | -0.027 | 0.02 |
| rs11615712 | 12 | 69619736 | No | No | - | - | - | - | - | - | - | - | - |
| rs11113118 | 12 | 107199142 | Yes | No | A | G | 0.235 | 0.017 | 0 | -0.008 | 0.021 | 0.053 | 0.025 |
| rs4964736 | 12 | 109100503 | Yes | No | T | C | 0.359 | 0.01 | 0 | -0.011 | 0.017 | -0.029 | 0.021 |
| rs149793040 | 12 | 109661672 | No | No | - | - | - | - | - | - | - | - | - |
| rs2009170 | 12 | 111703313 | Yes | No | C | T | 0.255 | -0.013 | 0 | 0.038 | 0.019 | 0.038 | 0.023 |
| rs580063 | 12 | 123206340 | Yes | No | C | T | 0.212 | -0.017 | 0 | -0.002 | 0.021 | 0 | 0.025 |
| rs12303671 | 12 | 124492610 | Yes | No | G | T | 0.337 | -0.026 | 0 | -0.013 | 0.018 | -0.018 | 0.021 |
| rs11057840 | 12 | 125316055 | Yes | No | C | A | 0.138 | 0.019 | 0 | 0.014 | 0.025 | -0.036 | 0.03 |
| rs4770433 | 13 | 23903791 | Yes | Yes | G | A | 0.449 | -0.011 | 0 | -0.028 | 0.017 | -0.002 | 0.02 |
| rs1340819 | 13 | 29145323 | Yes | No | C | A | 0.337 | -0.01 | 0 | -0.023 | 0.018 | -0.017 | 0.021 |
| rs398098735 | 13 | 31001091 | No | No | - | - | - | - | - | - | - | - | - |
| rs138358301 | 13 | 45970147 | No | No | - | - | - | - | - | - | - | - | - |
| rs2812208 | 13 | 50707087 | Yes | No | C | G | 0.023 | -0.048 | 0 | -0.01 | 0.048 | 0.005 | 0.056 |
| rs797482 | 13 | 51214613 | Yes | No | G | A | 0.875 | 0.015 | 0 | 0.024 | 0.023 | -0.022 | 0.028 |
| rs2298058 | 13 | 95248566 | Yes | Yes | T | C | 0.316 | 0.018 | 0 | 0.042 | 0.018 | 0.009 | 0.022 |
| rs6602909 | 13 | 114551993 | Yes | No | C | T | 0.33 | 0.026 | 0 | -0.003 | 0.018 | 0.005 | 0.022 |
| rs72681869 | 14 | 50655357 | No | No | - | - | - | - | - | - | - | - | - |
| rs12878001 | 14 | 64239629 | Yes | No | G | T | 0.161 | 0.021 | 0 | -0.016 | 0.023 | 0.015 | 0.027 |
| rs3825669 | 14 | 89804276 | Yes | No | G | A | 0.805 | -0.012 | 0 | -0.035 | 0.022 | -0.009 | 0.026 |
| rs61993685 | 14 | 100765823 | Yes | No | C | T | 0.071 | -0.019 | 0 | -0.005 | 0.032 | -0.02 | 0.038 |
| rs12891399 | 14 | 104293533 | Yes | No | C | T | 0.339 | 0.011 | 0 | -0.014 | 0.018 | -0.005 | 0.021 |
| rs28624578 | 15 | 31637666 | Yes | No | C | T | 0.166 | 0.013 | 0 | -0.019 | 0.022 | -0.044 | 0.026 |
| rs275179 | 15 | 39447529 | Yes | No | G | A | 0.158 | -0.015 | 0 | 0.005 | 0.023 | -0.028 | 0.028 |
| rs34245505 | 15 | 40397191 | Yes | No | G | C | 0.188 | 0.015 | 0 | 0.021 | 0.023 | 0.002 | 0.027 |
| rs72735627 | 15 | 41057507 | Yes | No | T | C | 0.088 | -0.021 | 0 | 0.05 | 0.029 | -0.038 | 0.034 |
| rs8025212 | 15 | 41880619 | Yes | No | G | A | 0.324 | -0.011 | 0 | -0.003 | 0.018 | -0.066 | 0.022 |
| rs139661283 | 15 | 42384229 | Yes | No | A | G | 0.014 | 0.06 | 0 | -0.084 | 0.081 | 0.073 | 0.096 |
| rs139974673 | 15 | 44027885 | Yes | No | C | T | 0.026 | 0.134 | 0 | 0.03 | 0.056 | -0.047 | 0.066 |
| rs1901529 | 15 | 45001900 | Yes | No | G | A | 0.121 | 0.018 | 0 | 0.047 | 0.027 | 0.023 | 0.032 |
| rs72749502 | 15 | 57297078 | Yes | No | T | A | 0.07 | 0.03 | 0 | 0.036 | 0.033 | 0.081 | 0.04 |
| rs1077835 | 15 | 58723426 | Yes | No | G | A | 0.216 | 0.045 | 0 | -0.025 | 0.021 | -0.01 | 0.025 |
| rs112147665 | 15 | 59277019 | No | No | - | - | - | - | - | - | - | - | - |
| rs12438742 | 15 | 61947280 | No | No | - | - | - | - | - | - | - | - | - |
| rs11635675 | 15 | 63793238 | Yes | Yes | G | T | 0.352 | 0.024 | 0 | 0.002 | 0.018 | -0.005 | 0.021 |
| rs12442852 | 15 | 64677413 | Yes | No | T | C | 0.881 | 0.015 | 0 | 0 | 0.025 | -0.05 | 0.03 |
| rs2218181 | 15 | 66872325 | Yes | Yes | C | T | 0.346 | 0.014 | 0 | 0.017 | 0.018 | -0.029 | 0.021 |
| rs11072332 | 15 | 72108307 | Yes | No | T | G | 0.214 | -0.012 | 0 | -0.004 | 0.021 | -0.021 | 0.025 |
| rs7164727 | 15 | 73093991 | Yes | No | T | C | 0.681 | 0.011 | 0 | 0.022 | 0.018 | 0.015 | 0.022 |
| rs1037117 | 15 | 102068658 | Yes | Yes | A | G | 0.253 | 0.014 | 0 | 0.012 | 0.02 | 0.019 | 0.024 |
| rs12600110 | 16 | 962154 | Yes | No | C | T | 0.375 | -0.015 | 0 | -0.017 | 0.017 | 0.034 | 0.021 |
| rs12921195 | 16 | 4677604 | Yes | No | A | C | 0.129 | 0.017 | 0 | -0.005 | 0.028 | 0.021 | 0.033 |
| rs12928099 | 16 | 15150505 | Yes | No | A | C | 0.297 | -0.026 | 0 | 0.03 | 0.019 | 0.027 | 0.022 |
| rs7196161 | 16 | 31110981 | Yes | No | A | G | 0.624 | 0.015 | 0 | 0.032 | 0.017 | 0.015 | 0.021 |
| rs62033400 | 16 | 53811788 | Yes | No | G | A | 0.401 | 0.016 | 0 | 0.003 | 0.017 | 0.007 | 0.02 |
| rs17231506 | 16 | 56994528 | Yes | No | T | C | 0.32 | -0.033 | 0 | 0.017 | 0.018 | -0.009 | 0.021 |
| rs73597575 | 16 | 67677001 | Yes | No | T | C | 0.085 | -0.017 | 0 | -0.039 | 0.027 | 0.014 | 0.032 |
| rs6499240 | 16 | 69686912 | Yes | No | G | A | 0.582 | 0.016 | 0 | 0.024 | 0.017 | 0.029 | 0.02 |
| rs12445401 | 16 | 72148419 | Yes | No | G | A | 0.192 | 0.022 | 0 | 0.031 | 0.021 | 0.019 | 0.025 |
| rs76862947 | 16 | 79746461 | Yes | No | C | T | 0.289 | 0.012 | 0 | 0.002 | 0.018 | 0.011 | 0.021 |
| rs2925979 | 16 | 81534790 | Yes | No | C | T | 0.699 | -0.026 | 0 | 0.006 | 0.018 | 0.009 | 0.022 |
| rs11641586 | 16 | 85270707 | Yes | No | T | G | 0.198 | -0.014 | 0 | 0.019 | 0.021 | 0.001 | 0.025 |
| rs8054124 | 16 | 86434553 | Yes | No | C | T | 0.231 | 0.012 | 0 | -0.016 | 0.02 | 0.006 | 0.024 |
| rs11870735 | 17 | 481604 | Yes | No | T | C | 0.176 | 0.013 | 0 | 0.03 | 0.023 | -0.06 | 0.027 |
| rs11078597 | 17 | 1618363 | Yes | Yes | C | T | 0.187 | 0.017 | 0 | -0.003 | 0.021 | -0.037 | 0.025 |
| rs79202680 | 17 | 4692640 | No | No | - | - | - | - | - | - | - | - | - |
| rs571475342 | 17 | 5207882 | No | No | - | - | - | - | - | - | - | - | - |
| rs200489612 | 17 | 7106378 | No | No | - | - | - | - | - | - | - | - | - |
| rs9905032 | 17 | 17457768 | Yes | No | A | G | 0.063 | 0.029 | 0 | -0.044 | 0.034 | -0.018 | 0.041 |
| rs704 | 17 | 26694861 | Yes | No | A | G | 0.477 | -0.011 | 0 | 0.028 | 0.017 | 0.006 | 0.02 |
| rs17767256 | 17 | 28581043 | Yes | No | G | T | 0.337 | -0.011 | 0 | 0.034 | 0.018 | -0.031 | 0.021 |
| rs650558 | 17 | 40721042 | Yes | No | T | C | 0.242 | 0.015 | 0 | -0.045 | 0.019 | -0.043 | 0.023 |
| rs186398347 | 17 | 41335716 | No | No | - | - | - | - | - | - | - | - | - |
| rs72836561 | 17 | 41926126 | Yes | No | T | C | 0.03 | 0.15 | 0 | 0.025 | 0.049 | 0.034 | 0.058 |
| rs72824773 | 17 | 42626279 | Yes | No | A | G | 0.006 | 0.074 | 0 | 0.078 | 0.074 | 0.079 | 0.087 |
| rs7207542 | 17 | 45697549 | No | No | - | - | - | - | - | - | - | - | - |
| rs10775406 | 17 | 46197755 | Yes | No | G | A | 0.752 | 0.015 | 0 | -0.037 | 0.02 | -0.038 | 0.023 |
| rs28394864 | 17 | 47450775 | Yes | No | A | G | 0.462 | 0.016 | 0 | -0.008 | 0.017 | -0.054 | 0.02 |
| rs1292072 | 17 | 57925649 | Yes | No | G | A | 0.211 | 0.016 | 0 | 0.008 | 0.021 | 0.046 | 0.025 |
| rs1801689 | 17 | 64210580 | Yes | No | C | A | 0.026 | -0.057 | 0 | 0.021 | 0.058 | 0.105 | 0.068 |
| rs11656743 | 17 | 65980289 | Yes | No | A | G | 0.788 | -0.024 | 0 | -0.028 | 0.021 | 0.02 | 0.024 |
| rs77542162 | 17 | 67081278 | Yes | No | G | A | 0.02 | -0.049 | 0 | -0.001 | 0.062 | -0.015 | 0.073 |
| rs717118 | 17 | 68464817 | No | No | - | - | - | - | - | - | - | - | - |
| rs3744231 | 17 | 73268801 | Yes | No | T | C | 0.701 | 0.01 | 0 | 0.01 | 0.019 | -0.001 | 0.023 |
| rs193220 | 17 | 74268619 | Yes | No | T | C | 0.309 | -0.012 | 0 | -0.012 | 0.018 | 0.034 | 0.022 |
| rs7212201 | 17 | 76393896 | Yes | No | A | C | 0.611 | -0.02 | 0 | -0.004 | 0.017 | 0.033 | 0.021 |
| rs62078746 | 17 | 80053590 | Yes | No | A | G | 0.553 | -0.011 | 0 | -0.003 | 0.017 | 0.021 | 0.021 |
| rs8083730 | 18 | 293519 | No | No | - | - | - | - | - | - | - | - | - |
| rs11664106 | 18 | 2846812 | No | No | - | - | - | - | - | - | - | - | - |
| rs35321365 | 18 | 19905141 | Yes | No | T | C | 0.516 | -0.011 | 0 | -0.018 | 0.017 | -0.008 | 0.02 |
| rs1623060 | 18 | 21143183 | Yes | No | T | C | 0.493 | -0.015 | 0 | 0.011 | 0.017 | -0.009 | 0.02 |
| rs150261265 | 18 | 29772887 | No | No | - | - | - | - | - | - | - | - | - |
| rs143393480 | 18 | 56089858 | No | No | - | - | - | - | - | - | - | - | - |
| rs1457489 | 18 | 57861961 | Yes | No | A | G | 0.27 | 0.013 | 0 | 0.012 | 0.019 | 0.04 | 0.022 |
| rs12454712 | 18 | 60845884 | No | No | - | - | - | - | - | - | - | - | - |
| rs1135908 | 19 | 808742 | Yes | No | T | G | 0.146 | 0.013 | 0 | 0.031 | 0.023 | 0.024 | 0.028 |
| rs350885 | 19 | 4147445 | Yes | Yes | C | A | 0.742 | 0.017 | 0 | 0.012 | 0.019 | -0.014 | 0.023 |
| rs35502362 | 19 | 4966041 | Yes | No | T | C | 0.338 | -0.015 | 0 | -0.009 | 0.018 | -0.001 | 0.022 |
| rs4804414 | 19 | 7223785 | Yes | No | T | C | 0.427 | 0.02 | 0 | -0.02 | 0.017 | -0.008 | 0.02 |
| rs11672003 | 19 | 7909765 | Yes | No | A | G | 0.276 | 0.014 | 0 | -0.013 | 0.019 | -0.007 | 0.022 |
| rs116843064 | 19 | 8429323 | Yes | No | A | G | 0.02 | -0.236 | 0 | 0.039 | 0.053 | 0.069 | 0.065 |
| rs555628099 | 19 | 8945880 | No | No | - | - | - | - | - | - | - | - | - |
| rs145464906 | 19 | 11350874 | No | No | - | - | - | - | - | - | - | - | - |
| rs1966500 | 19 | 18735666 | Yes | No | C | T | 0.452 | -0.012 | 0 | -0.024 | 0.017 | -0.018 | 0.02 |
| rs58542926 | 19 | 19379549 | Yes | No | T | C | 0.075 | -0.108 | 0 | 0.054 | 0.029 | 0.049 | 0.034 |
| rs75916629 | 19 | 20213781 | Yes | No | G | A | 0.025 | -0.057 | 0 | 0.003 | 0.043 | 0.008 | 0.051 |
| rs7256564 | 19 | 33889593 | Yes | No | G | A | 0.689 | -0.015 | 0 | 0.01 | 0.018 | -0.022 | 0.022 |
| rs58895965 | 19 | 35551428 | Yes | No | A | C | 0.165 | 0.024 | 0 | -0.002 | 0.022 | 0.02 | 0.026 |
| rs35538872 | 19 | 41754430 | No | No | - | - | - | - | - | - | - | - | - |
| rs141226346 | 19 | 44890969 | No | No | - | - | - | - | - | - | - | - | - |
| rs483082 | 19 | 45416178 | Yes | No | T | G | 0.229 | 0.096 | 0 | -0.009 | 0.019 | 0.036 | 0.023 |
| rs12459222 | 19 | 46859301 | Yes | No | C | G | 0.301 | -0.013 | 0 | 0.019 | 0.018 | 0.029 | 0.022 |
| rs10408163 | 19 | 47597102 | Yes | No | C | T | 0.709 | 0.012 | 0 | 0.007 | 0.019 | 0.026 | 0.022 |
| rs62129968 | 19 | 48383542 | Yes | No | A | C | 0.162 | -0.016 | 0 | 0.035 | 0.024 | 0.004 | 0.028 |
| rs838133 | 19 | 49259529 | No | No | - | - | - | - | - | - | - | - | - |
| rs113886122 | 19 | 50044741 | Yes | No | G | C | 0.151 | 0.02 | 0 | -0.023 | 0.024 | 0.014 | 0.028 |
| rs798889 | 19 | 54793250 | Yes | No | T | G | 0.22 | -0.013 | 0 | 0.012 | 0.019 | -0.051 | 0.023 |
| rs7251733 | 19 | 56099951 | Yes | No | A | G | 0.161 | 0.021 | 0 | 0.017 | 0.023 | -0.012 | 0.028 |
| rs8102873 | 19 | 57488423 | Yes | No | T | C | 0.581 | 0.01 | 0 | 0.026 | 0.017 | -0.027 | 0.02 |
| rs151235402 | 20 | 569164 | Yes | No | T | C | 0.017 | 0.048 | 0 | -0.032 | 0.055 | -0.046 | 0.065 |
| rs6120879 | 20 | 30202961 | Yes | No | C | T | 0.164 | -0.013 | 0 | -0.008 | 0.022 | 0.032 | 0.027 |
| rs67611724 | 20 | 32308275 | Yes | No | T | C | 0.147 | 0.016 | 0 | -0.003 | 0.025 | -0.013 | 0.03 |
| rs78185617 | 20 | 34124603 | Yes | No | C | G | 0.132 | -0.018 | 0 | -0.009 | 0.027 | -0.021 | 0.032 |
| rs1883711 | 20 | 39179822 | Yes | No | C | G | 0.032 | 0.059 | 0 | 0.03 | 0.037 | 0.015 | 0.043 |
| rs6102322 | 20 | 39872768 | Yes | No | T | C | 0.298 | 0.016 | 0 | -0.026 | 0.019 | -0.053 | 0.022 |
| rs6073958 | 20 | 44551855 | Yes | No | C | T | 0.202 | 0.049 | 0 | 0.021 | 0.021 | -0.035 | 0.025 |
| rs6066138 | 20 | 45594711 | Yes | No | A | G | 0.274 | -0.017 | 0 | 0.02 | 0.019 | -0.014 | 0.023 |
| rs6068174 | 20 | 51046309 | Yes | No | T | C | 0.637 | 0.011 | 0 | 0.016 | 0.017 | 0.003 | 0.021 |
| rs6070138 | 20 | 56118558 | Yes | Yes | A | C | 0.594 | 0.015 | 0 | 0.019 | 0.017 | 0.011 | 0.021 |
| rs8126001 | 20 | 62711459 | Yes | No | T | C | 0.484 | -0.016 | 0 | 0.025 | 0.017 | 0.006 | 0.021 |
| rs8134638 | 21 | 40644170 | Yes | No | C | T | 0.384 | 0.012 | 0 | 0.005 | 0.017 | 0.008 | 0.021 |
| rs960230 | 21 | 42622479 | Yes | No | G | A | 0.786 | 0.012 | 0 | -0.015 | 0.02 | -0.005 | 0.024 |
| rs200559406 | 21 | 46875817 | No | No | - | - | - | - | - | - | - | - | - |
| rs35665085 | 22 | 17625915 | Yes | No | A | G | 0.056 | 0.023 | 0 | -0.022 | 0.035 | 0.014 | 0.041 |
| rs4822458 | 22 | 24265659 | Yes | No | T | C | 0.546 | -0.011 | 0 | -0.032 | 0.017 | -0.02 | 0.02 |
| rs4352308 | 22 | 29131788 | Yes | No | T | C | 0.708 | 0.012 | 0 | 0.019 | 0.018 | -0.026 | 0.022 |
| rs2530679 | 22 | 30082569 | No | No | - | - | - | - | - | - | - | - | - |
| rs9610329 | 22 | 36042986 | No | No | - | - | - | - | - | - | - | - | - |
| rs4820325 | 22 | 38599978 | Yes | No | A | G | 0.582 | 0.021 | 0 | 0.001 | 0.017 | 0.015 | 0.021 |
| rs4821815 | 22 | 39105707 | Yes | Yes | A | G | 0.334 | 0.015 | 0 | -0.033 | 0.018 | -0.011 | 0.022 |
| rs3747207 | 22 | 44324855 | Yes | No | A | G | 0.222 | -0.012 | 0 | 0.002 | 0.021 | 0.011 | 0.025 |
| rs4253766 | 22 | 46623905 | Yes | Yes | T | C | 0.107 | 0.023 | 0 | -0.031 | 0.029 | 0.009 | 0.034 |
| rs140947018 | 22 | 50165082 | Yes | No | A | G | 0.029 | -0.031 | 0 | 0.081 | 0.056 | 0.035 | 0.066 |
| rs73188911 | 22 | 50746706 | Yes | No | T | C | 0.082 | -0.018 | 0 | -0.026 | 0.031 | 0.008 | 0.036 |
