# Supplementary material for: Impaired glucose tolerance and cardiovascular risk factors in relation to infertility: a Mendelian randomization analysis in the Norwegian Mother, Father, and Child Cohort Study
Source: Hum Reprod. 2023 Nov 8;39(2):436–41. doi: 10.1093/humrep/dead234 (PMC10833082; doi:10.1093/humrep/dead234)
Supplement: dead234_Supplementary_Table_S7 [file dead234_supplementary_table_s7.docx]

**Supplementary Table S7.** SNPs included in systolic blood pressure-related analyses.

| **RSID** | **Chrom.** | **Position** | **Used in MR** | **Used in MR**  **+ Steiger filt.** | **Effect**  **allele** | **Other**  **allele** | **Effect**  **allele**  **freq.** | **Exposure:**  **beta** | **Exposure:**  **SE** | **Outcome**  **(women):**  **beta** | **Outcome**  **(women):**  **SE** | **Outcome**  **(men):**  **beta** | **Outcome**  **(men):**  **SE** |
| --- | --- | --- | --- | --- | --- | --- | --- | --- | --- | --- | --- | --- | --- |
| rs2076328 | 1 | 1687482 | Yes | No | T | G | 0.489 | -0.334 | 0.031 | -0.016 | 0.017 | 0.003 | 0.02 |
| rs260508 | 1 | 2187085 | Yes | No | T | G | 0.615 | 0.158 | 0.031 | 0.015 | 0.017 | 0.017 | 0.021 |
| rs2493292 | 1 | 3328659 | Yes | No | T | C | 0.145 | 0.408 | 0.044 | -0.021 | 0.024 | -0.023 | 0.028 |
| rs709209 | 1 | 6278414 | Yes | No | A | G | 0.658 | 0.09 | 0.033 | 0.022 | 0.018 | -0.007 | 0.022 |
| rs4908678 | 1 | 7739250 | Yes | No | T | C | 0.625 | -0.134 | 0.032 | -0.007 | 0.017 | 0.021 | 0.021 |
| rs2252865 | 1 | 8422676 | Yes | No | T | C | 0.348 | 0.202 | 0.032 | 0.013 | 0.017 | 0.001 | 0.021 |
| rs9662255 | 1 | 9441949 | Yes | No | A | C | 0.427 | -0.229 | 0.031 | 0 | 0.017 | 0.028 | 0.02 |
| rs880315 | 1 | 10796866 | Yes | Yes | T | C | 0.656 | -0.508 | 0.032 | 0.013 | 0.018 | 0.019 | 0.021 |
| rs17367504 | 1 | 11862778 | Yes | Yes | A | G | 0.841 | 0.897 | 0.041 | 0.003 | 0.023 | 0.022 | 0.028 |
| rs3820068 | 1 | 15798197 | Yes | No | A | G | 0.804 | 0.294 | 0.039 | 0.023 | 0.021 | 0.02 | 0.025 |
| rs2807337 | 1 | 22577371 | Yes | Yes | T | C | 0.366 | 0.186 | 0.031 | 0.022 | 0.017 | -0.027 | 0.021 |
| rs150266910 | 1 | 23442265 | Yes | No | T | C | 0.177 | 0.115 | 0.039 | -0.021 | 0.023 | 0.01 | 0.028 |
| rs6686889 | 1 | 25030470 | Yes | No | T | C | 0.253 | 0.205 | 0.035 | -0.004 | 0.019 | -0.014 | 0.023 |
| rs79598313 | 1 | 27284913 | Yes | No | T | C | 0.025 | 0.665 | 0.1 | 0.016 | 0.048 | -0.005 | 0.057 |
| rs3737801 | 1 | 27960832 | Yes | No | C | G | 0.922 | 0.389 | 0.06 | -0.002 | 0.034 | -0.035 | 0.042 |
| rs143167197 | 1 | 28734372 | Yes | No | A | G | 0.929 | -0.434 | 0.062 | -0.047 | 0.031 | -0.034 | 0.038 |
| rs1565716 | 1 | 29549216 | Yes | No | A | G | 0.07 | 0.247 | 0.059 | -0.037 | 0.036 | -0.046 | 0.043 |
| rs4652875 | 1 | 33868469 | No | No | - | - | - | - | - | - | - | - | - |
| rs9729719 | 1 | 38298207 | Yes | No | A | G | 0.293 | 0.153 | 0.034 | -0.011 | 0.019 | 0.003 | 0.022 |
| rs11210029 | 1 | 41865293 | Yes | No | A | G | 0.632 | -0.203 | 0.031 | 0.029 | 0.017 | -0.019 | 0.021 |
| rs7515635 | 1 | 42408070 | Yes | No | T | C | 0.462 | 0.251 | 0.03 | 0 | 0.017 | 0.033 | 0.02 |
| rs72659998 | 1 | 43037556 | Yes | No | T | C | 0.151 | -0.181 | 0.043 | -0.05 | 0.024 | 0.036 | 0.029 |
| rs839755 | 1 | 43856410 | Yes | No | A | C | 0.615 | -0.266 | 0.031 | 0.022 | 0.017 | -0.006 | 0.021 |
| rs512083 | 1 | 46027355 | Yes | No | T | C | 0.535 | -0.111 | 0.03 | 0.015 | 0.017 | 0.02 | 0.02 |
| rs12142296 | 1 | 46541679 | Yes | No | T | G | 0.864 | -0.112 | 0.044 | 0.035 | 0.025 | 0.003 | 0.03 |
| rs4926923 | 1 | 48109225 | Yes | No | T | C | 0.911 | 0.32 | 0.054 | 0.02 | 0.03 | -0.034 | 0.036 |
| rs11579440 | 1 | 49052423 | Yes | Yes | T | C | 0.848 | 0.267 | 0.043 | -0.025 | 0.025 | -0.001 | 0.029 |
| rs147696085 | 1 | 51021867 | Yes | No | A | G | 0.094 | -0.084 | 0.053 | 0.054 | 0.027 | -0.021 | 0.032 |
| rs6681713 | 1 | 51527684 | Yes | No | T | G | 0.981 | 0.318 | 0.114 | 0.035 | 0.05 | -0.076 | 0.059 |
| rs112557609 | 1 | 56576924 | Yes | No | A | G | 0.344 | 0.284 | 0.032 | -0.016 | 0.018 | 0.001 | 0.022 |
| rs2404715 | 1 | 57008778 | Yes | No | T | C | 0.092 | -0.305 | 0.052 | -0.002 | 0.03 | 0.041 | 0.036 |
| rs60199046 | 1 | 59663341 | Yes | No | A | G | 0.711 | 0.272 | 0.033 | -0.008 | 0.018 | -0.008 | 0.021 |
| rs20354 | 1 | 67071356 | Yes | No | T | G | 0.134 | 0.201 | 0.044 | 0.007 | 0.024 | -0.009 | 0.028 |
| rs34517439 | 1 | 78450517 | No | No | - | - | - | - | - | - | - | - | - |
| rs12034319 | 1 | 86040107 | Yes | No | A | G | 0.22 | 0.127 | 0.036 | 0.016 | 0.02 | 0.031 | 0.024 |
| rs385437 | 1 | 86822231 | Yes | No | A | G | 0.86 | 0.183 | 0.044 | -0.029 | 0.024 | 0.03 | 0.029 |
| rs10923038 | 1 | 88651771 | Yes | Yes | A | C | 0.618 | 0.201 | 0.031 | -0.011 | 0.017 | 0.001 | 0.02 |
| rs10922502 | 1 | 89360158 | Yes | No | A | G | 0.628 | -0.286 | 0.031 | -0.024 | 0.018 | -0.008 | 0.021 |
| rs2065152 | 1 | 90228519 | Yes | No | T | C | 0.357 | 0.155 | 0.031 | -0.033 | 0.018 | -0.023 | 0.021 |
| rs17516329 | 1 | 92319781 | Yes | No | A | T | 0.685 | 0.097 | 0.033 | -0.006 | 0.018 | 0.041 | 0.022 |
| rs7514579 | 1 | 94051350 | Yes | No | A | C | 0.771 | 0.224 | 0.036 | 0.007 | 0.02 | 0.007 | 0.023 |
| rs17396055 | 1 | 94730954 | Yes | No | A | G | 0.332 | -0.176 | 0.032 | -0.026 | 0.018 | -0.033 | 0.022 |
| rs17030613 | 1 | 113190807 | Yes | No | A | C | 0.79 | -0.405 | 0.037 | 0.002 | 0.02 | 0.009 | 0.024 |
| rs2932538 | 1 | 113216543 | Yes | No | A | G | 0.257 | -0.339 | 0.034 | 0.022 | 0.019 | -0.025 | 0.023 |
| rs12078697 | 1 | 117015118 | Yes | No | C | G | 0.211 | -0.146 | 0.037 | -0.012 | 0.021 | 0.004 | 0.026 |
| rs7553422 | 1 | 119540719 | Yes | No | T | C | 0.412 | -0.101 | 0.031 | 0.009 | 0.017 | -0.009 | 0.021 |
| rs72704264 | 1 | 145713305 | Yes | No | C | G | 0.216 | 0.093 | 0.037 | 0.019 | 0.02 | -0.011 | 0.025 |
| rs11585169 | 1 | 150572037 | No | No | - | - | - | - | - | - | - | - | - |
| rs13796 | 1 | 154245917 | Yes | No | T | C | 0.864 | -0.053 | 0.045 | 0.027 | 0.026 | 0.012 | 0.031 |
| rs76719272 | 1 | 156129796 | Yes | No | T | C | 0.131 | -0.274 | 0.046 | 0.022 | 0.024 | -0.021 | 0.028 |
| rs2171690 | 1 | 164740099 | Yes | No | T | C | 0.535 | 0.153 | 0.03 | -0.02 | 0.017 | 0.038 | 0.02 |
| rs7524019 | 1 | 167367193 | Yes | No | T | C | 0.491 | 0.131 | 0.03 | -0.01 | 0.017 | -0.029 | 0.02 |
| rs2157597 | 1 | 169201567 | Yes | No | T | C | 0.355 | -0.143 | 0.032 | -0.008 | 0.018 | 0.02 | 0.021 |
| rs12405515 | 1 | 172357441 | Yes | No | T | G | 0.571 | -0.141 | 0.03 | -0.022 | 0.017 | 0.017 | 0.02 |
| rs12118102 | 1 | 176634724 | Yes | No | A | G | 0.947 | 0.192 | 0.068 | -0.088 | 0.036 | -0.02 | 0.043 |
| rs150816167 | 1 | 179571862 | Yes | No | T | C | 0.955 | -0.237 | 0.078 | 0.064 | 0.043 | 0.067 | 0.052 |
| rs10913934 | 1 | 180131640 | Yes | No | T | G | 0.593 | 0.102 | 0.031 | 0.003 | 0.017 | -0.033 | 0.021 |
| rs1043069 | 1 | 180859368 | Yes | Yes | T | G | 0.616 | 0.234 | 0.031 | -0.006 | 0.017 | -0.005 | 0.021 |
| rs41475048 | 1 | 183058452 | Yes | No | A | G | 0.745 | -0.123 | 0.035 | -0.022 | 0.02 | 0.013 | 0.023 |
| rs4651224 | 1 | 184585182 | Yes | No | T | C | 0.447 | 0.199 | 0.031 | 0.007 | 0.017 | -0.002 | 0.02 |
| rs12042924 | 1 | 197297417 | Yes | No | T | C | 0.528 | -0.181 | 0.03 | 0.014 | 0.017 | -0.003 | 0.02 |
| rs882624 | 1 | 201735913 | No | No | - | - | - | - | - | - | - | - | - |
| rs33996239 | 1 | 203109801 | Yes | No | T | C | 0.06 | -0.366 | 0.066 | -0.037 | 0.038 | -0.043 | 0.046 |
| rs4245739 | 1 | 204518842 | Yes | No | A | C | 0.728 | 0.095 | 0.034 | 0.026 | 0.019 | -0.035 | 0.023 |
| rs2629665 | 1 | 207220800 | Yes | No | A | C | 0.41 | -0.18 | 0.031 | -0.017 | 0.017 | -0.014 | 0.02 |
| rs2761436 | 1 | 207919748 | Yes | No | T | C | 0.537 | 0.184 | 0.03 | 0.005 | 0.017 | 0.024 | 0.02 |
| rs7555285 | 1 | 209970355 | Yes | No | C | G | 0.801 | 0.229 | 0.038 | -0.008 | 0.02 | -0.036 | 0.024 |
| rs12408022 | 1 | 217718789 | Yes | No | T | C | 0.259 | 0.194 | 0.035 | -0.017 | 0.019 | 0.018 | 0.022 |
| rs35981664 | 1 | 218549354 | Yes | No | A | T | 0.688 | -0.156 | 0.033 | -0.035 | 0.018 | -0.009 | 0.022 |
| rs2820443 | 1 | 219753509 | Yes | No | T | C | 0.709 | 0.16 | 0.033 | 0.022 | 0.018 | -0.018 | 0.022 |
| rs9431431 | 1 | 221358796 | Yes | No | A | G | 0.294 | -0.211 | 0.033 | -0.033 | 0.018 | -0.062 | 0.022 |
| rs73091767 | 1 | 227250775 | Yes | No | T | C | 0.734 | -0.179 | 0.034 | 0.024 | 0.019 | 0.011 | 0.023 |
| rs2004776 | 1 | 230848702 | Yes | No | T | C | 0.24 | 0.379 | 0.035 | 0.046 | 0.02 | -0.054 | 0.024 |
| rs6429422 | 1 | 243472801 | Yes | No | T | G | 0.677 | -0.153 | 0.032 | 0.003 | 0.018 | 0.029 | 0.022 |
| rs4926499 | 1 | 249155909 | No | No | - | - | - | - | - | - | - | - | - |
| rs4850047 | 2 | 3634753 | Yes | No | T | C | 0.147 | -0.132 | 0.045 | 0.003 | 0.024 | -0.02 | 0.029 |
| rs2175337 | 2 | 9298590 | Yes | No | A | C | 0.61 | 0.146 | 0.031 | -0.01 | 0.017 | -0.02 | 0.021 |
| rs67720684 | 2 | 18975439 | Yes | No | A | C | 0.238 | 0.194 | 0.035 | -0.029 | 0.021 | 0.001 | 0.025 |
| rs1344653 | 2 | 19730845 | Yes | No | A | G | 0.499 | -0.253 | 0.03 | -0.025 | 0.017 | -0.004 | 0.02 |
| rs7255 | 2 | 20878820 | Yes | No | T | C | 0.454 | -0.152 | 0.03 | -0.005 | 0.017 | -0.009 | 0.02 |
| rs66774912 | 2 | 21423532 | Yes | No | A | G | 0.137 | -0.164 | 0.044 | -0.033 | 0.027 | -0.013 | 0.033 |
| rs10779936 | 2 | 23950200 | Yes | No | A | G | 0.713 | -0.141 | 0.033 | 0.027 | 0.019 | 0.028 | 0.022 |
| rs55701159 | 2 | 25139596 | Yes | No | T | G | 0.887 | 0.396 | 0.048 | 0 | 0.024 | 0.076 | 0.029 |
| rs1275988 | 2 | 26914364 | Yes | Yes | T | C | 0.611 | -0.541 | 0.031 | -0.014 | 0.017 | -0.008 | 0.021 |
| rs9678851 | 2 | 27887034 | Yes | No | A | C | 0.568 | -0.172 | 0.031 | 0.002 | 0.017 | 0.002 | 0.02 |
| rs7562 | 2 | 28635740 | Yes | Yes | T | C | 0.521 | 0.231 | 0.03 | -0.009 | 0.017 | -0.001 | 0.02 |
| rs1607644 | 2 | 34679626 | Yes | No | A | G | 0.366 | -0.127 | 0.031 | 0.015 | 0.017 | 0.003 | 0.021 |
| rs13420463 | 2 | 37517566 | Yes | No | A | G | 0.773 | 0.314 | 0.036 | -0.009 | 0.02 | 0.056 | 0.024 |
| rs2707238 | 2 | 38094149 | Yes | No | C | G | 0.284 | 0.093 | 0.034 | 0 | 0.019 | 0.062 | 0.022 |
| rs4952611 | 2 | 40567743 | Yes | No | T | C | 0.58 | -0.211 | 0.032 | -0.011 | 0.017 | 0.007 | 0.021 |
| rs11681462 | 2 | 42352567 | Yes | No | A | C | 0.789 | -0.178 | 0.037 | 0 | 0.021 | -0.006 | 0.025 |
| rs76326501 | 2 | 43167878 | Yes | No | A | C | 0.909 | 0.568 | 0.053 | -0.036 | 0.03 | -0.01 | 0.036 |
| rs35590893 | 2 | 43716933 | Yes | No | A | G | 0.277 | -0.237 | 0.034 | -0.007 | 0.019 | 0.042 | 0.023 |
| rs11690961 | 2 | 46363336 | Yes | No | A | C | 0.883 | 0.139 | 0.047 | -0.005 | 0.027 | 0.027 | 0.032 |
| rs6545155 | 2 | 50429861 | Yes | Yes | T | C | 0.782 | 0.182 | 0.036 | 0.002 | 0.022 | 0.015 | 0.026 |
| rs10189186 | 2 | 53025757 | Yes | No | A | G | 0.53 | 0.189 | 0.03 | 0.002 | 0.017 | 0.004 | 0.02 |
| rs2920899 | 2 | 55279681 | Yes | No | T | G | 0.786 | 0.199 | 0.037 | 0.018 | 0.02 | 0.021 | 0.024 |
| rs1975487 | 2 | 55809054 | Yes | No | A | G | 0.481 | -0.199 | 0.031 | -0.005 | 0.017 | -0.019 | 0.02 |
| rs6730325 | 2 | 59315828 | Yes | No | A | G | 0.605 | -0.127 | 0.031 | -0.005 | 0.017 | -0.013 | 0.021 |
| rs72816333 | 2 | 60096560 | Yes | No | A | T | 0.83 | 0.234 | 0.04 | -0.023 | 0.022 | 0.036 | 0.026 |
| rs925484 | 2 | 60611437 | Yes | No | C | G | 0.6 | -0.17 | 0.031 | 0.022 | 0.017 | 0.002 | 0.021 |
| rs7608483 | 2 | 61836235 | Yes | No | A | C | 0.418 | 0.138 | 0.031 | -0.002 | 0.017 | 0.004 | 0.021 |
| rs13014371 | 2 | 64217786 | Yes | No | T | C | 0.571 | -0.171 | 0.03 | 0.02 | 0.017 | 0.02 | 0.02 |
| rs2631669 | 2 | 66104881 | Yes | No | T | C | 0.471 | 0.043 | 0.03 | 0.016 | 0.017 | -0.034 | 0.02 |
| rs2300481 | 2 | 66782467 | Yes | Yes | T | C | 0.386 | 0.198 | 0.031 | -0.009 | 0.017 | -0.003 | 0.021 |
| rs6731373 | 2 | 68503044 | Yes | No | A | G | 0.349 | 0.191 | 0.033 | 0.006 | 0.018 | -0.01 | 0.021 |
| rs12052761 | 2 | 69065841 | Yes | No | A | G | 0.394 | -0.134 | 0.031 | -0.007 | 0.017 | 0.035 | 0.021 |
| rs3771371 | 2 | 71627539 | Yes | No | T | C | 0.569 | -0.149 | 0.03 | 0.033 | 0.017 | 0 | 0.02 |
| rs10193543 | 2 | 72483329 | Yes | No | T | G | 0.837 | 0.202 | 0.041 | 0.007 | 0.023 | -0.008 | 0.027 |
| rs1876487 | 2 | 73114352 | Yes | No | A | C | 0.294 | -0.128 | 0.034 | 0.001 | 0.018 | -0.025 | 0.022 |
| rs11689667 | 2 | 85491365 | Yes | No | T | C | 0.545 | 0.198 | 0.03 | -0.028 | 0.017 | -0.029 | 0.02 |
| rs72847885 | 2 | 86326717 | Yes | No | A | G | 0.663 | 0.241 | 0.032 | -0.008 | 0.018 | -0.03 | 0.021 |
| rs2579519 | 2 | 96675166 | Yes | No | T | C | 0.616 | -0.009 | 0.031 | 0 | 0.017 | -0.017 | 0.021 |
| rs4851462 | 2 | 98357163 | No | No | - | - | - | - | - | - | - | - | - |
| rs150194832 | 2 | 106126880 | Yes | No | C | G | 0.093 | -0.29 | 0.052 | -0.019 | 0.032 | -0.014 | 0.039 |
| rs28377357 | 2 | 112769721 | No | No | - | - | - | - | - | - | - | - | - |
| rs62158170 | 2 | 114082175 | Yes | No | A | G | 0.783 | 0.237 | 0.037 | -0.008 | 0.021 | -0.016 | 0.025 |
| rs10864859 | 2 | 121440218 | Yes | No | T | G | 0.915 | 0.309 | 0.057 | -0.003 | 0.033 | -0.027 | 0.04 |
| rs6723509 | 2 | 122000745 | Yes | No | T | C | 0.861 | 0.253 | 0.044 | -0.001 | 0.026 | 0.036 | 0.031 |
| rs13001283 | 2 | 127183454 | Yes | No | A | G | 0.16 | 0.182 | 0.042 | -0.016 | 0.022 | -0.064 | 0.026 |
| rs4954192 | 2 | 135632981 | Yes | No | T | C | 0.391 | -0.18 | 0.031 | 0.012 | 0.018 | 0.021 | 0.022 |
| rs72844590 | 2 | 138421227 | Yes | No | T | G | 0.15 | 0.217 | 0.043 | 0.024 | 0.022 | 0.029 | 0.027 |
| rs7606205 | 2 | 144146311 | Yes | No | A | C | 0.702 | -0.184 | 0.033 | 0.017 | 0.019 | 0.023 | 0.023 |
| rs1438896 | 2 | 145646072 | Yes | No | T | C | 0.298 | 0.208 | 0.033 | -0.02 | 0.018 | 0.038 | 0.022 |
| rs34570306 | 2 | 146272860 | Yes | No | T | C | 0.528 | -0.152 | 0.031 | 0.026 | 0.017 | -0.023 | 0.02 |
| rs62169544 | 2 | 146950908 | Yes | No | A | G | 0.442 | -0.188 | 0.03 | 0.039 | 0.017 | 0.007 | 0.02 |
| rs12990959 | 2 | 148572160 | Yes | No | T | C | 0.687 | -0.097 | 0.033 | -0.005 | 0.018 | 0.001 | 0.021 |
| rs4664080 | 2 | 152978341 | Yes | No | A | G | 0.398 | -0.145 | 0.031 | -0.003 | 0.017 | 0.026 | 0.021 |
| rs3175 | 2 | 153618773 | Yes | No | A | G | 0.652 | 0.16 | 0.032 | 0.011 | 0.018 | -0.025 | 0.022 |
| rs1220128 | 2 | 158499902 | Yes | No | C | G | 0.852 | 0.203 | 0.043 | -0.015 | 0.026 | -0.012 | 0.031 |
| rs79523138 | 2 | 161368213 | Yes | No | A | G | 0.881 | -0.247 | 0.047 | -0.017 | 0.026 | -0.045 | 0.031 |
| rs55732192 | 2 | 162278233 | Yes | No | T | G | 0.095 | -0.336 | 0.052 | -0.018 | 0.031 | -0.052 | 0.037 |
| rs1446468 | 2 | 164963486 | Yes | Yes | T | C | 0.454 | -0.506 | 0.03 | -0.007 | 0.017 | -0.002 | 0.02 |
| rs6712203 | 2 | 165557318 | Yes | No | T | C | 0.372 | -0.209 | 0.031 | 0 | 0.017 | -0.016 | 0.021 |
| rs2390258 | 2 | 166250129 | Yes | No | A | G | 0.307 | -0.131 | 0.033 | -0.025 | 0.019 | -0.012 | 0.023 |
| rs560887 | 2 | 169763148 | Yes | No | T | C | 0.299 | -0.157 | 0.033 | 0.022 | 0.019 | -0.014 | 0.022 |
| rs151054210 | 2 | 172381487 | Yes | No | A | G | 0.182 | 0.182 | 0.039 | 0.02 | 0.021 | 0.003 | 0.026 |
| rs6758859 | 2 | 173965056 | Yes | No | T | C | 0.636 | 0.09 | 0.031 | -0.037 | 0.018 | 0 | 0.021 |
| rs11694601 | 2 | 174949358 | Yes | No | A | G | 0.597 | -0.191 | 0.031 | 0.03 | 0.017 | -0.012 | 0.02 |
| rs72914576 | 2 | 175529967 | Yes | No | C | G | 0.81 | -0.292 | 0.039 | 0.015 | 0.021 | 0.011 | 0.025 |
| rs60148403 | 2 | 177989414 | Yes | No | A | T | 0.196 | -0.204 | 0.038 | -0.019 | 0.022 | 0.008 | 0.026 |
| rs1837164 | 2 | 178716601 | Yes | Yes | A | T | 0.37 | 0.182 | 0.031 | -0.01 | 0.017 | -0.008 | 0.021 |
| rs79146658 | 2 | 179786068 | Yes | No | T | C | 0.915 | -0.074 | 0.054 | -0.009 | 0.032 | -0.046 | 0.038 |
| rs1486236 | 2 | 180739450 | Yes | Yes | A | C | 0.37 | -0.234 | 0.032 | -0.004 | 0.018 | -0.018 | 0.021 |
| rs10184839 | 2 | 181946115 | Yes | No | A | T | 0.291 | -0.185 | 0.033 | -0.008 | 0.019 | -0.002 | 0.023 |
| rs16823124 | 2 | 183224127 | Yes | No | A | G | 0.307 | 0.236 | 0.033 | 0.004 | 0.018 | 0.04 | 0.021 |
| rs12473688 | 2 | 185033470 | Yes | No | A | G | 0.284 | 0.18 | 0.033 | 0.005 | 0.019 | -0.023 | 0.023 |
| rs28558491 | 2 | 187816321 | Yes | No | T | C | 0.734 | -0.211 | 0.034 | 0.02 | 0.019 | 0.02 | 0.023 |
| rs11901929 | 2 | 189643316 | Yes | No | A | G | 0.346 | 0.091 | 0.032 | -0.012 | 0.018 | 0.036 | 0.022 |
| rs7592578 | 2 | 191439591 | Yes | No | T | G | 0.195 | -0.286 | 0.039 | -0.001 | 0.023 | 0.014 | 0.027 |
| rs296797 | 2 | 201102905 | Yes | Yes | T | C | 0.405 | 0.216 | 0.031 | 0.031 | 0.017 | -0.005 | 0.02 |
| rs1469760 | 2 | 204125426 | Yes | No | T | C | 0.584 | -0.248 | 0.031 | -0.019 | 0.017 | 0.043 | 0.02 |
| rs2162003 | 2 | 205077128 | No | No | - | - | - | - | - | - | - | - | - |
| rs1263671 | 2 | 207996447 | Yes | No | T | C | 0.837 | -0.13 | 0.042 | 0.036 | 0.024 | -0.014 | 0.028 |
| rs55780018 | 2 | 208526140 | Yes | No | T | C | 0.543 | -0.278 | 0.031 | -0.001 | 0.017 | -0.043 | 0.02 |
| rs1047891 | 2 | 211540507 | Yes | No | A | C | 0.319 | -0.253 | 0.033 | 0.003 | 0.018 | -0.039 | 0.022 |
| rs12694277 | 2 | 213188795 | Yes | No | T | C | 0.295 | -0.202 | 0.034 | 0.002 | 0.019 | 0.008 | 0.023 |
| rs4674114 | 2 | 217659266 | Yes | No | A | G | 0.201 | -0.177 | 0.038 | -0.003 | 0.022 | 0.003 | 0.026 |
| rs1063281 | 2 | 218668732 | Yes | No | T | C | 0.603 | -0.258 | 0.031 | -0.026 | 0.017 | -0.016 | 0.021 |
| rs1996992 | 2 | 219651349 | Yes | No | T | G | 0.051 | -0.125 | 0.069 | 0.042 | 0.038 | 0 | 0.046 |
| rs12474050 | 2 | 220362557 | Yes | No | T | C | 0.343 | 0.065 | 0.032 | 0.004 | 0.018 | -0.015 | 0.021 |
| rs2972146 | 2 | 227100698 | Yes | No | T | G | 0.642 | 0.253 | 0.031 | 0.001 | 0.017 | 0.032 | 0.021 |
| rs1044822 | 2 | 230629138 | Yes | No | T | C | 0.148 | -0.248 | 0.042 | -0.027 | 0.024 | -0.061 | 0.029 |
| rs12052878 | 2 | 238227594 | Yes | No | A | G | 0.32 | -0.116 | 0.032 | 0.009 | 0.018 | -0.001 | 0.022 |
| rs4507125 | 2 | 239864732 | Yes | No | A | C | 0.786 | -0.121 | 0.037 | -0.033 | 0.021 | -0.005 | 0.025 |
| rs139354822 | 2 | 242344695 | Yes | No | T | C | 0.97 | 0.612 | 0.098 | 0.059 | 0.05 | 0.041 | 0.059 |
| rs9865843 | 3 | 7489993 | Yes | No | A | G | 0.518 | -0.137 | 0.031 | 0.009 | 0.017 | -0.041 | 0.02 |
| rs347591 | 3 | 11290122 | Yes | Yes | T | G | 0.662 | 0.318 | 0.032 | 0.038 | 0.018 | -0.001 | 0.022 |
| rs729639 | 3 | 13826854 | Yes | No | T | C | 0.345 | -0.113 | 0.032 | -0.016 | 0.018 | 0.005 | 0.021 |
| rs11128722 | 3 | 14958126 | Yes | No | A | G | 0.57 | -0.286 | 0.031 | -0.009 | 0.017 | -0.006 | 0.021 |
| rs189267552 | 3 | 20073193 | Yes | No | A | T | 0.013 | -0.866 | 0.139 | -0.002 | 0.077 | 0.01 | 0.093 |
| rs4634143 | 3 | 23163749 | Yes | No | T | C | 0.3 | 0.148 | 0.033 | 0.004 | 0.018 | -0.023 | 0.022 |
| rs13082711 | 3 | 27537909 | Yes | No | T | C | 0.762 | -0.309 | 0.035 | 0.019 | 0.02 | -0.02 | 0.024 |
| rs72851229 | 3 | 29374219 | Yes | No | C | G | 0.175 | -0.179 | 0.04 | 0.004 | 0.023 | -0.009 | 0.027 |
| rs12638085 | 3 | 30405936 | Yes | Yes | A | T | 0.356 | 0.219 | 0.032 | -0.007 | 0.018 | 0.016 | 0.021 |
| rs4678915 | 3 | 36964583 | Yes | No | A | G | 0.432 | -0.138 | 0.031 | 0.007 | 0.017 | 0.035 | 0.021 |
| rs267517 | 3 | 37539090 | Yes | No | A | G | 0.592 | -0.261 | 0.031 | -0.009 | 0.017 | 0.045 | 0.021 |
| rs6801957 | 3 | 38767315 | Yes | No | T | C | 0.41 | 0.087 | 0.031 | -0.006 | 0.017 | 0.008 | 0.021 |
| rs6788984 | 3 | 41107173 | Yes | Yes | A | G | 0.856 | 0.3 | 0.043 | -0.033 | 0.025 | -0.015 | 0.03 |
| rs9815354 | 3 | 41912651 | Yes | No | A | G | 0.164 | -0.217 | 0.041 | -0.051 | 0.023 | 0.006 | 0.028 |
| rs141979279 | 3 | 44858131 | Yes | No | T | C | 0.951 | 0.288 | 0.07 | -0.094 | 0.04 | -0.065 | 0.048 |
| rs113134141 | 3 | 46861939 | Yes | No | A | G | 0.898 | -0.273 | 0.05 | -0.011 | 0.028 | 0.011 | 0.034 |
| rs6797587 | 3 | 48197614 | Yes | No | A | G | 0.328 | -0.327 | 0.032 | 0.03 | 0.018 | -0.025 | 0.022 |
| rs73082337 | 3 | 49009570 | Yes | No | C | G | 0.879 | 0.28 | 0.048 | 0.03 | 0.026 | -0.05 | 0.031 |
| rs36022378 | 3 | 49913705 | Yes | No | T | C | 0.8 | -0.192 | 0.038 | -0.01 | 0.022 | 0.02 | 0.026 |
| rs13303 | 3 | 52558008 | Yes | No | T | C | 0.436 | -0.096 | 0.031 | 0.038 | 0.017 | 0.016 | 0.02 |
| rs9810888 | 3 | 53635595 | Yes | No | T | G | 0.498 | -0.172 | 0.03 | 0.014 | 0.017 | 0.01 | 0.02 |
| rs9827472 | 3 | 56726646 | No | No | - | - | - | - | - | - | - | - | - |
| rs12486605 | 3 | 57706503 | Yes | No | T | C | 0.573 | -0.083 | 0.031 | -0.006 | 0.017 | 0.013 | 0.02 |
| rs3774702 | 3 | 63856870 | Yes | No | A | G | 0.177 | 0.051 | 0.04 | 0.003 | 0.023 | 0.01 | 0.027 |
| rs918466 | 3 | 64710253 | Yes | No | A | G | 0.41 | -0.067 | 0.031 | 0.001 | 0.017 | 0.039 | 0.021 |
| rs7630745 | 3 | 66427029 | Yes | No | T | C | 0.659 | 0.169 | 0.032 | 0.002 | 0.018 | -0.033 | 0.021 |
| rs4499560 | 3 | 70920485 | Yes | No | A | T | 0.317 | -0.22 | 0.033 | 0.015 | 0.018 | 0.036 | 0.022 |
| rs729448 | 3 | 73260545 | Yes | No | A | G | 0.547 | -0.121 | 0.03 | -0.026 | 0.017 | -0.01 | 0.02 |
| rs9857362 | 3 | 74710462 | Yes | No | A | C | 0.529 | 0.173 | 0.031 | 0.013 | 0.017 | -0.003 | 0.02 |
| rs1375564 | 3 | 85656311 | Yes | No | T | C | 0.639 | 0.258 | 0.032 | 0.009 | 0.018 | -0.004 | 0.021 |
| rs9860290 | 3 | 99839106 | Yes | No | A | G | 0.209 | -0.139 | 0.037 | 0.042 | 0.02 | -0.023 | 0.024 |
| rs11923667 | 3 | 101268080 | No | No | - | - | - | - | - | - | - | - | - |
| rs28675079 | 3 | 111500002 | Yes | No | A | G | 0.188 | -0.176 | 0.039 | 0.002 | 0.021 | 0.017 | 0.025 |
| rs1882289 | 3 | 114461208 | Yes | No | A | G | 0.885 | -0.209 | 0.047 | 0.046 | 0.026 | -0.001 | 0.031 |
| rs6806529 | 3 | 123049938 | Yes | No | A | C | 0.434 | 0.177 | 0.031 | -0.007 | 0.017 | -0.001 | 0.02 |
| rs6438857 | 3 | 124557643 | Yes | No | T | C | 0.577 | 0.274 | 0.03 | -0.017 | 0.017 | -0.007 | 0.021 |
| rs62270945 | 3 | 128201889 | Yes | No | T | C | 0.029 | 0.436 | 0.096 | -0.033 | 0.052 | -0.066 | 0.062 |
| rs9875380 | 3 | 132780356 | Yes | No | T | C | 0.464 | -0.175 | 0.03 | -0.017 | 0.017 | -0.003 | 0.02 |
| rs6783086 | 3 | 133959552 | Yes | No | T | C | 0.395 | 0.305 | 0.031 | 0.006 | 0.017 | -0.008 | 0.021 |
| rs590198 | 3 | 135953729 | Yes | No | A | G | 0.529 | 0.187 | 0.03 | 0.01 | 0.017 | 0.026 | 0.02 |
| rs2306374 | 3 | 138119952 | Yes | No | T | C | 0.838 | -0.282 | 0.041 | 0.004 | 0.023 | -0.03 | 0.028 |
| rs16851397 | 3 | 141134818 | Yes | No | A | G | 0.953 | -0.529 | 0.073 | 0.023 | 0.037 | 0.004 | 0.044 |
| rs62278541 | 3 | 142631909 | Yes | No | A | G | 0.647 | 0.137 | 0.032 | -0.002 | 0.018 | 0.007 | 0.021 |
| rs6772704 | 3 | 149524692 | Yes | No | A | C | 0.684 | -0.124 | 0.032 | 0.008 | 0.018 | 0.014 | 0.021 |
| rs73158427 | 3 | 153721493 | Yes | No | A | T | 0.162 | 0.28 | 0.041 | 0.008 | 0.023 | -0.012 | 0.027 |
| rs143112823 | 3 | 154707967 | Yes | No | A | G | 0.088 | -0.417 | 0.056 | -0.021 | 0.03 | -0.066 | 0.036 |
| rs9833313 | 3 | 157576791 | Yes | Yes | A | T | 0.242 | -0.191 | 0.036 | -0.007 | 0.02 | 0.012 | 0.024 |
| rs78151625 | 3 | 158316726 | Yes | No | T | C | 0.834 | -0.245 | 0.041 | -0.003 | 0.024 | -0.001 | 0.028 |
| rs419076 | 3 | 169100886 | Yes | No | T | C | 0.473 | 0.405 | 0.03 | 0.018 | 0.017 | -0.01 | 0.02 |
| rs4894535 | 3 | 171995605 | Yes | No | T | C | 0.16 | 0.188 | 0.041 | 0 | 0.022 | -0.039 | 0.026 |
| rs73171158 | 3 | 176927949 | No | No | - | - | - | - | - | - | - | - | - |
| rs7611674 | 3 | 179169230 | Yes | No | T | G | 0.803 | 0.118 | 0.039 | -0.011 | 0.022 | -0.003 | 0.026 |
| rs262986 | 3 | 183435713 | Yes | No | A | G | 0.47 | -0.237 | 0.03 | 0.013 | 0.017 | -0.04 | 0.02 |
| rs12374077 | 3 | 185317674 | Yes | No | C | G | 0.344 | 0.214 | 0.032 | -0.03 | 0.018 | -0.003 | 0.022 |
| rs1706003 | 3 | 194299967 | No | No | - | - | - | - | - | - | - | - | - |
| rs6777317 | 3 | 197070959 | No | No | - | - | - | - | - | - | - | - | - |
| rs1250129 | 4 | 1254930 | Yes | No | A | G | 0.115 | -0.206 | 0.047 | 0.039 | 0.026 | -0.03 | 0.031 |
| rs55829085 | 4 | 2165493 | Yes | No | A | C | 0.955 | -0.431 | 0.074 | -0.013 | 0.041 | -0.074 | 0.05 |
| rs231708 | 4 | 2694773 | Yes | No | C | G | 0.692 | -0.282 | 0.033 | -0.006 | 0.018 | -0.031 | 0.022 |
| rs2498323 | 4 | 3451109 | Yes | No | A | G | 0.098 | 0.317 | 0.052 | -0.048 | 0.031 | 0.016 | 0.037 |
| rs3822239 | 4 | 10095539 | Yes | No | A | T | 0.697 | 0.147 | 0.033 | -0.003 | 0.018 | 0.008 | 0.022 |
| rs13122790 | 4 | 15356795 | Yes | No | A | G | 0.732 | 0.116 | 0.034 | 0.011 | 0.019 | -0.011 | 0.022 |
| rs11730129 | 4 | 16032948 | Yes | No | T | C | 0.218 | -0.082 | 0.037 | -0.005 | 0.02 | 0.036 | 0.024 |
| rs2610990 | 4 | 18008232 | Yes | No | A | G | 0.264 | -0.29 | 0.034 | -0.032 | 0.019 | -0.019 | 0.023 |
| rs28667801 | 4 | 26785356 | No | No | - | - | - | - | - | - | - | - | - |
| rs1878825 | 4 | 36091370 | Yes | No | C | G | 0.642 | -0.122 | 0.032 | 0.005 | 0.017 | -0.035 | 0.021 |
| rs2291435 | 4 | 38387395 | Yes | Yes | T | C | 0.533 | -0.262 | 0.03 | 0.001 | 0.017 | 0.006 | 0.02 |
| rs12511987 | 4 | 46595623 | Yes | No | T | G | 0.823 | -0.233 | 0.04 | 0.027 | 0.021 | 0.014 | 0.026 |
| rs871606 | 4 | 54799245 | Yes | No | T | C | 0.895 | 0.37 | 0.05 | 0.027 | 0.03 | 0.02 | 0.036 |
| rs1718845 | 4 | 57943153 | Yes | No | A | G | 0.298 | -0.094 | 0.033 | 0.011 | 0.019 | 0.01 | 0.022 |
| rs6551716 | 4 | 63575696 | Yes | No | A | T | 0.856 | 0.214 | 0.044 | -0.01 | 0.026 | 0.005 | 0.031 |
| rs10008637 | 4 | 77414144 | Yes | No | T | C | 0.54 | 0.216 | 0.03 | -0.013 | 0.017 | 0.013 | 0.02 |
| rs1458038 | 4 | 81164723 | Yes | No | T | C | 0.294 | 0.823 | 0.033 | 0.02 | 0.017 | -0.017 | 0.021 |
| rs6823199 | 4 | 83925895 | Yes | No | T | C | 0.744 | 0.209 | 0.035 | -0.019 | 0.02 | -0.003 | 0.024 |
| rs2014912 | 4 | 86715670 | Yes | Yes | T | C | 0.153 | 0.477 | 0.042 | 0.013 | 0.022 | -0.019 | 0.027 |
| rs13149209 | 4 | 89750668 | Yes | No | T | C | 0.777 | 0.281 | 0.037 | -0.032 | 0.02 | 0.022 | 0.024 |
| rs7694000 | 4 | 95324968 | No | No | - | - | - | - | - | - | - | - | - |
| rs1347345 | 4 | 95938386 | Yes | Yes | A | G | 0.618 | -0.181 | 0.031 | -0.001 | 0.017 | -0.02 | 0.021 |
| rs17248480 | 4 | 102435265 | Yes | No | A | G | 0.028 | -0.844 | 0.097 | -0.129 | 0.07 | 0.052 | 0.082 |
| rs13107325 | 4 | 103188709 | Yes | No | T | C | 0.074 | -0.909 | 0.059 | -0.037 | 0.04 | -0.011 | 0.048 |
| rs223361 | 4 | 103769304 | Yes | No | T | C | 0.665 | 0.048 | 0.032 | -0.016 | 0.018 | -0.014 | 0.021 |
| rs4699165 | 4 | 106109381 | Yes | No | A | G | 0.356 | 0.107 | 0.032 | -0.01 | 0.018 | 0.002 | 0.021 |
| rs13112725 | 4 | 106911742 | Yes | No | C | G | 0.763 | 0.414 | 0.036 | 0.016 | 0.02 | 0.083 | 0.025 |
| rs7694643 | 4 | 109017528 | Yes | No | A | G | 0.645 | -0.195 | 0.032 | -0.033 | 0.018 | -0.009 | 0.021 |
| rs6825911 | 4 | 111381638 | Yes | No | T | C | 0.791 | -0.307 | 0.038 | -0.012 | 0.021 | -0.005 | 0.026 |
| rs4834735 | 4 | 119958809 | Yes | No | T | C | 0.137 | 0.184 | 0.044 | 0.022 | 0.025 | 0.016 | 0.029 |
| rs66887589 | 4 | 120509279 | Yes | No | T | C | 0.522 | -0.195 | 0.03 | 0.009 | 0.017 | 0.002 | 0.02 |
| rs3097937 | 4 | 124794644 | Yes | No | A | T | 0.803 | 0.223 | 0.038 | 0.024 | 0.022 | -0.055 | 0.027 |
| rs7439567 | 4 | 138464842 | Yes | Yes | T | C | 0.411 | 0.254 | 0.031 | 0.017 | 0.017 | 0.027 | 0.02 |
| rs72719160 | 4 | 144051276 | Yes | No | A | T | 0.683 | -0.224 | 0.032 | 0.001 | 0.018 | -0.02 | 0.022 |
| rs4292285 | 4 | 145271954 | Yes | No | A | T | 0.402 | -0.14 | 0.031 | 0.024 | 0.017 | -0.027 | 0.02 |
| rs4835266 | 4 | 146821725 | Yes | No | T | C | 0.522 | 0.216 | 0.031 | 0.009 | 0.017 | -0.002 | 0.02 |
| rs10305838 | 4 | 148400256 | Yes | No | T | C | 0.859 | -0.266 | 0.043 | -0.022 | 0.027 | 0.033 | 0.032 |
| rs6823767 | 4 | 151295085 | Yes | No | T | C | 0.723 | -0.213 | 0.034 | 0.021 | 0.019 | 0.019 | 0.022 |
| rs13139571 | 4 | 156645513 | Yes | No | A | C | 0.237 | -0.33 | 0.035 | 0.019 | 0.02 | 0.029 | 0.024 |
| rs17035181 | 4 | 157678511 | Yes | No | T | G | 0.855 | 0.307 | 0.043 | 0.025 | 0.023 | 0.037 | 0.027 |
| rs869396 | 4 | 169688000 | Yes | No | A | C | 0.466 | -0.212 | 0.03 | -0.016 | 0.017 | -0.011 | 0.02 |
| rs4957026 | 5 | 361148 | Yes | No | A | G | 0.34 | 0.198 | 0.032 | 0.001 | 0.018 | 0.008 | 0.022 |
| rs10069690 | 5 | 1279790 | Yes | Yes | T | C | 0.258 | 0.31 | 0.037 | 0.001 | 0.019 | 0.011 | 0.023 |
| rs954767 | 5 | 3706050 | Yes | No | A | C | 0.741 | -0.213 | 0.035 | -0.002 | 0.019 | -0.009 | 0.023 |
| rs1173771 | 5 | 32815028 | Yes | Yes | A | G | 0.399 | -0.632 | 0.031 | 0.026 | 0.017 | 0.003 | 0.021 |
| rs74774746 | 5 | 33411769 | Yes | No | C | G | 0.261 | -0.19 | 0.035 | -0.017 | 0.019 | 0.018 | 0.023 |
| rs7710854 | 5 | 43824677 | Yes | No | A | G | 0.883 | 0.224 | 0.048 | -0.017 | 0.025 | 0.023 | 0.03 |
| rs6867399 | 5 | 52135543 | Yes | No | A | C | 0.267 | 0.158 | 0.036 | -0.035 | 0.02 | 0.028 | 0.023 |
| rs1694068 | 5 | 53283630 | Yes | No | A | T | 0.614 | 0.266 | 0.031 | 0.031 | 0.017 | 0.019 | 0.021 |
| rs13179413 | 5 | 55868097 | Yes | No | T | C | 0.282 | 0.224 | 0.035 | -0.009 | 0.019 | -0.011 | 0.023 |
| rs12515541 | 5 | 57095011 | Yes | No | T | G | 0.608 | 0.158 | 0.031 | 0.016 | 0.017 | -0.01 | 0.021 |
| rs1848510 | 5 | 57754005 | Yes | No | A | G | 0.362 | 0.184 | 0.032 | 0.009 | 0.018 | -0.003 | 0.021 |
| rs10062049 | 5 | 61553881 | Yes | No | T | C | 0.136 | 0.273 | 0.044 | -0.017 | 0.024 | -0.008 | 0.029 |
| rs6875372 | 5 | 64079015 | No | No | - | - | - | - | - | - | - | - | - |
| rs3121685 | 5 | 65662133 | Yes | Yes | T | C | 0.484 | -0.156 | 0.03 | 0.003 | 0.017 | -0.023 | 0.02 |
| rs4286632 | 5 | 66291370 | Yes | No | A | G | 0.731 | 0.211 | 0.034 | 0.001 | 0.019 | 0.004 | 0.023 |
| rs246973 | 5 | 68007803 | Yes | Yes | T | C | 0.288 | 0.248 | 0.034 | 0.002 | 0.019 | 0.008 | 0.023 |
| rs72761109 | 5 | 71506529 | Yes | No | T | C | 0.303 | 0.15 | 0.033 | 0.006 | 0.019 | 0.015 | 0.022 |
| rs4443403 | 5 | 72654304 | Yes | No | T | C | 0.903 | -0.25 | 0.051 | -0.024 | 0.028 | 0.028 | 0.034 |
| rs10078021 | 5 | 75038431 | Yes | No | T | G | 0.627 | -0.048 | 0.032 | 0.014 | 0.017 | -0.002 | 0.021 |
| rs10057188 | 5 | 77837789 | Yes | No | A | G | 0.458 | -0.228 | 0.031 | -0.024 | 0.017 | 0.009 | 0.02 |
| rs10059921 | 5 | 87514515 | Yes | No | T | G | 0.082 | -0.425 | 0.059 | -0.083 | 0.035 | 0.07 | 0.043 |
| rs62380354 | 5 | 89484911 | Yes | No | A | C | 0.892 | 0.216 | 0.051 | 0.037 | 0.028 | -0.007 | 0.034 |
| rs709668 | 5 | 96174186 | Yes | Yes | A | G | 0.2 | -0.294 | 0.038 | -0.025 | 0.021 | -0.007 | 0.025 |
| rs1871190 | 5 | 97953719 | Yes | Yes | T | G | 0.335 | 0.195 | 0.032 | -0.001 | 0.018 | -0.009 | 0.021 |
| rs286809 | 5 | 107458637 | Yes | No | A | G | 0.172 | -0.175 | 0.04 | -0.023 | 0.022 | 0.025 | 0.026 |
| rs79409628 | 5 | 108113740 | Yes | No | T | G | 0.084 | -0.258 | 0.054 | 0.014 | 0.034 | -0.008 | 0.04 |
| rs10077885 | 5 | 114390121 | Yes | Yes | A | C | 0.508 | -0.259 | 0.031 | 0.001 | 0.017 | -0.013 | 0.02 |
| rs1432457 | 5 | 119781569 | Yes | No | A | G | 0.715 | -0.156 | 0.033 | -0.018 | 0.019 | -0.008 | 0.022 |
| rs9885577 | 5 | 121194226 | Yes | No | T | C | 0.371 | 0.231 | 0.032 | -0.003 | 0.017 | -0.022 | 0.021 |
| rs13359291 | 5 | 122476457 | Yes | Yes | A | G | 0.157 | 0.433 | 0.042 | 0.012 | 0.022 | -0.039 | 0.026 |
| rs6891344 | 5 | 123136656 | Yes | No | A | G | 0.818 | 0.271 | 0.04 | -0.014 | 0.023 | -0.017 | 0.027 |
| rs62373688 | 5 | 127352807 | Yes | No | A | T | 0.131 | 0.274 | 0.045 | -0.011 | 0.023 | -0.002 | 0.028 |
| rs6595838 | 5 | 127868199 | Yes | No | A | G | 0.298 | 0.323 | 0.033 | 0.028 | 0.018 | -0.001 | 0.022 |
| rs12521868 | 5 | 131784393 | Yes | No | T | G | 0.422 | -0.087 | 0.031 | 0.004 | 0.017 | -0.006 | 0.021 |
| rs55747751 | 5 | 132397351 | Yes | No | A | G | 0.081 | -0.278 | 0.058 | -0.045 | 0.028 | 0.064 | 0.033 |
| rs702395 | 5 | 140086677 | Yes | No | T | C | 0.437 | 0.232 | 0.03 | -0.016 | 0.017 | 0.002 | 0.02 |
| rs1650911 | 5 | 141740620 | Yes | No | C | G | 0.765 | 0.255 | 0.037 | -0.006 | 0.02 | 0.009 | 0.024 |
| rs2400509 | 5 | 147696018 | Yes | No | A | G | 0.258 | -0.167 | 0.034 | 0.004 | 0.019 | -0.048 | 0.023 |
| rs9687065 | 5 | 148391140 | Yes | No | A | G | 0.81 | 0.336 | 0.039 | 0.019 | 0.021 | 0.024 | 0.025 |
| rs157678 | 5 | 156145654 | Yes | No | A | T | 0.669 | -0.183 | 0.033 | -0.008 | 0.018 | 0.006 | 0.022 |
| rs11953630 | 5 | 157845402 | Yes | No | T | C | 0.366 | -0.453 | 0.032 | 0.023 | 0.018 | -0.007 | 0.021 |
| rs62385385 | 5 | 158367249 | Yes | No | A | T | 0.383 | 0.283 | 0.031 | -0.004 | 0.017 | -0.021 | 0.021 |
| rs114503346 | 5 | 172192350 | Yes | No | T | C | 0.046 | -0.18 | 0.075 | -0.008 | 0.043 | 0.005 | 0.05 |
| rs72812846 | 5 | 173377636 | Yes | No | A | T | 0.278 | -0.258 | 0.034 | -0.018 | 0.018 | 0.001 | 0.022 |
| rs28362590 | 5 | 176731452 | Yes | No | T | G | 0.755 | 0.13 | 0.035 | -0.021 | 0.02 | 0.002 | 0.024 |
| rs12153395 | 5 | 179411477 | Yes | No | A | G | 0.115 | -0.33 | 0.049 | -0.006 | 0.026 | 0.009 | 0.031 |
| rs2745599 | 6 | 1613686 | Yes | No | A | G | 0.552 | 0.216 | 0.032 | 0.003 | 0.017 | -0.004 | 0.02 |
| rs1334576 | 6 | 7211818 | Yes | No | A | G | 0.42 | -0.071 | 0.031 | 0.002 | 0.017 | -0.029 | 0.02 |
| rs9392172 | 6 | 7723962 | No | No | - | - | - | - | - | - | - | - | - |
| rs114275780 | 6 | 8224648 | Yes | No | A | T | 0.059 | 0.304 | 0.068 | -0.02 | 0.033 | -0.037 | 0.04 |
| rs1630736 | 6 | 12295987 | No | No | - | - | - | - | - | - | - | - | - |
| rs9349379 | 6 | 12903957 | Yes | No | A | G | 0.593 | 0.266 | 0.031 | 0 | 0.017 | -0.041 | 0.02 |
| rs12216497 | 6 | 19028623 | Yes | No | T | C | 0.562 | 0.155 | 0.03 | -0.01 | 0.017 | -0.002 | 0.02 |
| rs9368222 | 6 | 20686996 | Yes | No | A | C | 0.269 | 0.228 | 0.034 | 0.007 | 0.019 | -0.026 | 0.023 |
| rs6911827 | 6 | 22130601 | Yes | Yes | T | C | 0.458 | 0.238 | 0.031 | -0.007 | 0.017 | -0.012 | 0.02 |
| rs1799945 | 6 | 26091179 | Yes | No | C | G | 0.851 | -0.544 | 0.043 | 0.005 | 0.027 | -0.058 | 0.032 |
| rs409558 | 6 | 31708147 | Yes | No | T | C | 0.849 | 0.384 | 0.043 | 0.014 | 0.024 | 0.028 | 0.029 |
| rs115245297 | 6 | 34244132 | Yes | No | T | C | 0.956 | -0.445 | 0.077 | -0.092 | 0.044 | -0.043 | 0.053 |
| rs3176336 | 6 | 36648816 | Yes | No | A | T | 0.609 | 0.108 | 0.031 | 0.008 | 0.017 | -0.002 | 0.021 |
| rs4714224 | 6 | 39186743 | Yes | No | C | G | 0.278 | -0.081 | 0.034 | -0.041 | 0.019 | 0.033 | 0.023 |
| rs1563788 | 6 | 43308363 | Yes | No | T | C | 0.287 | 0.338 | 0.033 | 0.002 | 0.019 | -0.029 | 0.022 |
| rs9472135 | 6 | 43809802 | Yes | No | T | C | 0.699 | 0.047 | 0.033 | 0.038 | 0.018 | -0.013 | 0.022 |
| rs78648104 | 6 | 50683009 | Yes | No | T | C | 0.907 | -0.429 | 0.054 | 0.046 | 0.03 | 0.053 | 0.036 |
| rs13205180 | 6 | 51832494 | Yes | No | T | C | 0.488 | 0.124 | 0.03 | -0.007 | 0.017 | -0.024 | 0.02 |
| rs631441 | 6 | 53994626 | Yes | No | T | G | 0.695 | -0.169 | 0.033 | -0.02 | 0.018 | -0.001 | 0.022 |
| rs1925153 | 6 | 56102780 | Yes | No | T | C | 0.446 | -0.119 | 0.031 | -0.004 | 0.017 | 0.002 | 0.021 |
| rs504691 | 6 | 72206620 | Yes | No | A | C | 0.4 | -0.036 | 0.031 | -0.027 | 0.017 | -0.01 | 0.02 |
| rs12195276 | 6 | 73657714 | Yes | No | T | C | 0.725 | -0.167 | 0.034 | 0.008 | 0.019 | -0.025 | 0.023 |
| rs10943605 | 6 | 79655477 | Yes | No | A | G | 0.489 | 0.239 | 0.03 | 0.008 | 0.017 | 0.017 | 0.02 |
| rs7753695 | 6 | 80818531 | Yes | No | T | C | 0.437 | 0.098 | 0.031 | 0.016 | 0.017 | 0.021 | 0.02 |
| rs9449350 | 6 | 82281417 | Yes | No | T | C | 0.679 | -0.219 | 0.032 | -0.018 | 0.018 | -0.009 | 0.021 |
| rs60255247 | 6 | 85283253 | Yes | No | A | C | 0.887 | 0.219 | 0.049 | 0.021 | 0.029 | -0.059 | 0.034 |
| rs35410524 | 6 | 96885405 | Yes | No | T | C | 0.188 | 0.337 | 0.039 | 0.019 | 0.022 | 0.002 | 0.027 |
| rs9486916 | 6 | 109013930 | Yes | No | T | C | 0.198 | 0.266 | 0.038 | -0.033 | 0.022 | -0.011 | 0.026 |
| rs3822857 | 6 | 116313931 | Yes | No | C | G | 0.366 | -0.084 | 0.031 | 0.018 | 0.018 | 0.018 | 0.022 |
| rs2693560 | 6 | 117523671 | Yes | No | A | G | 0.366 | -0.206 | 0.032 | -0.015 | 0.018 | 0.012 | 0.021 |
| rs2498586 | 6 | 118026126 | Yes | Yes | T | C | 0.165 | -0.207 | 0.041 | 0.002 | 0.022 | 0.05 | 0.027 |
| rs9372498 | 6 | 118572486 | Yes | No | A | T | 0.081 | 0.397 | 0.056 | -0.045 | 0.032 | -0.057 | 0.038 |
| rs9401090 | 6 | 119113317 | Yes | No | T | C | 0.746 | 0.158 | 0.035 | -0.024 | 0.019 | -0.004 | 0.023 |
| rs11154027 | 6 | 121781390 | Yes | No | T | C | 0.461 | 0.063 | 0.031 | 0.018 | 0.017 | 0.005 | 0.02 |
| rs6925750 | 6 | 122287990 | Yes | No | T | C | 0.886 | 0.09 | 0.048 | -0.042 | 0.027 | -0.041 | 0.033 |
| rs10782230 | 6 | 126228512 | Yes | Yes | A | G | 0.484 | 0.211 | 0.03 | -0.012 | 0.017 | 0.008 | 0.02 |
| rs13209747 | 6 | 127115454 | Yes | No | T | C | 0.441 | 0.512 | 0.03 | 0.004 | 0.017 | -0.024 | 0.02 |
| rs9885632 | 6 | 131311909 | Yes | Yes | T | C | 0.731 | 0.236 | 0.034 | 0.026 | 0.02 | 0.013 | 0.024 |
| rs4896104 | 6 | 135119089 | Yes | No | T | C | 0.559 | -0.07 | 0.031 | 0.023 | 0.017 | -0.011 | 0.02 |
| rs668459 | 6 | 139835689 | Yes | No | T | C | 0.586 | -0.129 | 0.03 | -0.02 | 0.017 | 0.007 | 0.02 |
| rs7763294 | 6 | 140383733 | Yes | No | T | G | 0.316 | -0.2 | 0.032 | -0.018 | 0.018 | -0.014 | 0.022 |
| rs6941056 | 6 | 143591821 | No | No | - | - | - | - | - | - | - | - | - |
| rs7765526 | 6 | 147713764 | Yes | Yes | A | G | 0.463 | 0.201 | 0.031 | -0.017 | 0.017 | -0.004 | 0.02 |
| rs17080102 | 6 | 151004770 | Yes | No | C | G | 0.069 | -0.808 | 0.059 | 0.008 | 0.033 | 0.072 | 0.039 |
| rs9479200 | 6 | 152398505 | Yes | No | A | G | 0.886 | -0.256 | 0.048 | 0.054 | 0.025 | 0.038 | 0.03 |
| rs9479509 | 6 | 153427265 | Yes | No | A | G | 0.296 | -0.145 | 0.033 | 0.025 | 0.018 | 0.001 | 0.022 |
| rs598682 | 6 | 154418759 | Yes | No | A | G | 0.251 | -0.157 | 0.035 | 0.006 | 0.019 | -0.017 | 0.023 |
| rs449789 | 6 | 159699125 | Yes | No | C | G | 0.139 | 0.323 | 0.044 | 0.013 | 0.025 | 0.001 | 0.03 |
| rs555754 | 6 | 160769423 | Yes | No | A | G | 0.475 | -0.156 | 0.03 | 0.013 | 0.017 | -0.006 | 0.02 |
| rs9456648 | 6 | 161712235 | Yes | No | T | C | 0.323 | -0.177 | 0.032 | 0.005 | 0.018 | 0.054 | 0.021 |
| rs73030266 | 6 | 166179459 | Yes | No | A | T | 0.933 | 0.439 | 0.062 | 0.099 | 0.036 | 0.036 | 0.043 |
| rs4342401 | 6 | 169016615 | No | No | - | - | - | - | - | - | - | - | - |
| rs1322639 | 6 | 169587103 | Yes | No | A | G | 0.776 | 0.154 | 0.036 | 0.041 | 0.02 | -0.006 | 0.024 |
| rs6959688 | 7 | 1966831 | Yes | No | A | G | 0.598 | -0.234 | 0.031 | -0.003 | 0.017 | 0 | 0.021 |
| rs2969070 | 7 | 2512545 | Yes | No | A | G | 0.631 | -0.273 | 0.031 | -0.031 | 0.018 | -0.007 | 0.021 |
| rs73049928 | 7 | 4669949 | Yes | No | A | G | 0.806 | -0.238 | 0.039 | 0.023 | 0.02 | -0.038 | 0.024 |
| rs1468520 | 7 | 7290732 | Yes | No | A | G | 0.832 | -0.187 | 0.041 | -0.015 | 0.022 | -0.02 | 0.027 |
| rs13240040 | 7 | 14375977 | Yes | No | A | G | 0.684 | 0.175 | 0.033 | -0.008 | 0.019 | 0.026 | 0.022 |
| rs2107595 | 7 | 19049388 | Yes | No | A | G | 0.159 | 0.418 | 0.042 | -0.017 | 0.022 | 0.022 | 0.027 |
| rs4507656 | 7 | 22156538 | No | No | - | - | - | - | - | - | - | - | - |
| rs2069833 | 7 | 22767664 | Yes | No | T | C | 0.575 | -0.199 | 0.031 | 0.007 | 0.017 | 0.044 | 0.02 |
| rs12979 | 7 | 24738164 | Yes | Yes | C | G | 0.87 | 0.274 | 0.045 | 0.005 | 0.024 | -0.039 | 0.029 |
| rs1055144 | 7 | 25871109 | Yes | No | T | C | 0.193 | 0.11 | 0.038 | -0.008 | 0.022 | -0.019 | 0.026 |
| rs6969780 | 7 | 27159136 | Yes | No | C | G | 0.092 | 0.296 | 0.053 | 0.047 | 0.028 | -0.051 | 0.033 |
| rs7777128 | 7 | 27337113 | Yes | No | C | G | 0.08 | 0.516 | 0.056 | 0.049 | 0.032 | 0.035 | 0.038 |
| rs10274928 | 7 | 28142088 | Yes | No | A | G | 0.484 | 0.164 | 0.031 | -0.004 | 0.017 | -0.009 | 0.02 |
| rs917275 | 7 | 28658522 | Yes | No | A | G | 0.61 | -0.173 | 0.031 | -0.003 | 0.017 | -0.008 | 0.021 |
| rs10233127 | 7 | 30933453 | Yes | No | A | T | 0.108 | 0.256 | 0.051 | -0.01 | 0.028 | 0.053 | 0.033 |
| rs342989 | 7 | 35467896 | Yes | No | A | G | 0.228 | 0.091 | 0.036 | 0.002 | 0.021 | -0.007 | 0.025 |
| rs2052263 | 7 | 36225818 | Yes | No | A | G | 0.118 | 0.254 | 0.048 | 0.012 | 0.026 | 0.038 | 0.032 |
| rs76206723 | 7 | 40447971 | Yes | No | A | G | 0.107 | -0.404 | 0.049 | 0.007 | 0.026 | -0.014 | 0.031 |
| rs12701929 | 7 | 41722494 | Yes | No | T | C | 0.25 | 0.108 | 0.035 | 0.011 | 0.02 | 0.006 | 0.024 |
| rs1004558 | 7 | 44240407 | Yes | No | T | C | 0.178 | 0.164 | 0.04 | 0.015 | 0.024 | 0.022 | 0.028 |
| rs73105827 | 7 | 45036785 | Yes | No | T | G | 0.09 | -0.223 | 0.055 | -0.069 | 0.029 | 0.062 | 0.035 |
| rs11977526 | 7 | 46008110 | Yes | No | A | G | 0.401 | -0.321 | 0.031 | -0.025 | 0.017 | -0.009 | 0.021 |
| rs71543920 | 7 | 46554358 | Yes | No | T | G | 0.94 | -0.214 | 0.064 | -0.074 | 0.036 | 0.002 | 0.044 |
| rs12668436 | 7 | 47548893 | Yes | No | T | C | 0.754 | -0.215 | 0.035 | 0.018 | 0.02 | 0.011 | 0.024 |
| rs17454517 | 7 | 50915776 | Yes | No | A | G | 0.493 | 0.028 | 0.03 | -0.013 | 0.017 | -0.006 | 0.02 |
| rs6593297 | 7 | 56122058 | Yes | No | A | T | 0.303 | 0.167 | 0.034 | 0 | 0.019 | -0.027 | 0.023 |
| rs2222544 | 7 | 69769369 | Yes | No | T | C | 0.737 | -0.142 | 0.034 | 0.011 | 0.019 | -0.003 | 0.023 |
| rs1091811 | 7 | 73491212 | Yes | No | A | G | 0.169 | -0.155 | 0.04 | 0.016 | 0.023 | 0.004 | 0.028 |
| rs34324971 | 7 | 74107374 | Yes | No | A | G | 0.185 | 0.152 | 0.041 | -0.038 | 0.023 | -0.068 | 0.027 |
| rs6963105 | 7 | 75097488 | Yes | No | A | G | 0.439 | -0.189 | 0.032 | -0.009 | 0.017 | 0.018 | 0.02 |
| rs848445 | 7 | 77572461 | Yes | No | T | C | 0.285 | -0.202 | 0.034 | -0.026 | 0.018 | 0.017 | 0.022 |
| rs11770630 | 7 | 89805241 | Yes | No | T | C | 0.535 | 0.039 | 0.03 | 0.019 | 0.017 | 0.024 | 0.02 |
| rs10245696 | 7 | 90449362 | Yes | No | A | C | 0.4 | 0.17 | 0.031 | 0 | 0.017 | -0.033 | 0.02 |
| rs2282978 | 7 | 92264410 | Yes | No | T | C | 0.668 | 0.28 | 0.032 | 0.008 | 0.018 | 0.04 | 0.021 |
| rs1947228 | 7 | 96461649 | Yes | No | T | C | 0.418 | -0.176 | 0.031 | -0.008 | 0.017 | 0.015 | 0.02 |
| rs12705090 | 7 | 100467700 | Yes | No | T | C | 0.19 | -0.07 | 0.039 | 0.01 | 0.021 | 0.039 | 0.024 |
| rs17477177 | 7 | 106411858 | Yes | Yes | T | C | 0.796 | -0.735 | 0.038 | 0.018 | 0.02 | -0.011 | 0.024 |
| rs115172170 | 7 | 107019947 | Yes | No | T | C | 0.944 | -0.314 | 0.066 | -0.009 | 0.04 | 0.006 | 0.049 |
| rs1997571 | 7 | 116198621 | Yes | No | A | G | 0.591 | -0.155 | 0.031 | 0.043 | 0.017 | 0.059 | 0.02 |
| rs4728142 | 7 | 128573967 | Yes | Yes | A | G | 0.444 | -0.181 | 0.03 | -0.008 | 0.017 | 0.002 | 0.02 |
| rs11556924 | 7 | 129663496 | Yes | No | T | C | 0.382 | -0.21 | 0.032 | 0.001 | 0.018 | 0.029 | 0.021 |
| rs34072724 | 7 | 130432469 | Yes | No | A | G | 0.489 | -0.242 | 0.03 | -0.006 | 0.017 | 0.03 | 0.02 |
| rs13238550 | 7 | 131059056 | Yes | No | A | G | 0.396 | 0.257 | 0.031 | -0.011 | 0.017 | -0.003 | 0.02 |
| rs1722886 | 7 | 134215259 | No | No | - | - | - | - | - | - | - | - | - |
| rs10267979 | 7 | 136618188 | Yes | No | A | T | 0.324 | -0.12 | 0.032 | -0.016 | 0.018 | 0.025 | 0.021 |
| rs7810028 | 7 | 139461616 | Yes | No | C | G | 0.807 | 0.284 | 0.038 | -0.013 | 0.022 | -0.015 | 0.026 |
| rs12703989 | 7 | 140238048 | Yes | No | A | G | 0.499 | 0.153 | 0.031 | 0.006 | 0.017 | 0.006 | 0.02 |
| rs73727605 | 7 | 149474622 | Yes | No | A | G | 0.066 | 0.362 | 0.062 | -0.032 | 0.037 | -0.059 | 0.044 |
| rs11771693 | 7 | 150050111 | Yes | No | A | G | 0.675 | 0.183 | 0.033 | 0.01 | 0.018 | -0.013 | 0.022 |
| rs3918226 | 7 | 150690176 | Yes | No | T | C | 0.081 | 0.664 | 0.058 | -0.018 | 0.031 | -0.017 | 0.037 |
| rs891511 | 7 | 150704843 | Yes | No | A | G | 0.334 | -0.351 | 0.033 | -0.027 | 0.018 | 0.011 | 0.021 |
| rs10224002 | 7 | 151415041 | Yes | Yes | A | G | 0.714 | -0.367 | 0.034 | -0.023 | 0.019 | -0.017 | 0.023 |
| rs1870735 | 7 | 155744303 | No | No | - | - | - | - | - | - | - | - | - |
| rs9638084 | 7 | 156311745 | Yes | No | A | G | 0.398 | 0.158 | 0.031 | -0.008 | 0.017 | 0.018 | 0.02 |
| rs4875958 | 8 | 1721090 | Yes | No | A | G | 0.712 | 0.226 | 0.034 | -0.002 | 0.019 | 0.01 | 0.022 |
| rs2922895 | 8 | 6379932 | No | No | - | - | - | - | - | - | - | - | - |
| rs61040371 | 8 | 8503700 | Yes | No | T | C | 0.629 | 0.184 | 0.031 | -0.003 | 0.017 | -0.035 | 0.021 |
| rs62491354 | 8 | 9730663 | Yes | No | A | G | 0.134 | 0.309 | 0.044 | 0.023 | 0.024 | -0.041 | 0.029 |
| rs1986971 | 8 | 10268736 | Yes | Yes | A | G | 0.706 | 0.256 | 0.033 | -0.005 | 0.019 | 0.02 | 0.022 |
| rs2898290 | 8 | 11433909 | Yes | No | T | C | 0.476 | 0.312 | 0.03 | -0.003 | 0.017 | -0.042 | 0.02 |
| rs75902664 | 8 | 17427186 | Yes | No | A | G | 0.974 | -0.251 | 0.1 | 0.135 | 0.057 | 0.073 | 0.068 |
| rs1047030 | 8 | 22428708 | Yes | No | A | G | 0.804 | 0.207 | 0.041 | -0.015 | 0.021 | 0.074 | 0.025 |
| rs62503324 | 8 | 23400615 | Yes | No | T | C | 0.239 | 0.264 | 0.036 | 0.002 | 0.021 | -0.018 | 0.025 |
| rs6557876 | 8 | 25900675 | Yes | No | T | C | 0.25 | -0.416 | 0.035 | -0.003 | 0.019 | -0.012 | 0.023 |
| rs17321041 | 8 | 26445194 | Yes | No | T | C | 0.063 | 0.294 | 0.063 | -0.035 | 0.032 | -0.012 | 0.039 |
| rs2979470 | 8 | 30288272 | Yes | Yes | T | C | 0.49 | 0.199 | 0.03 | -0.011 | 0.017 | 0.003 | 0.02 |
| rs11991469 | 8 | 32413280 | No | No | - | - | - | - | - | - | - | - | - |
| rs7845722 | 8 | 33309993 | Yes | No | A | G | 0.601 | -0.127 | 0.031 | 0.031 | 0.017 | 0.025 | 0.021 |
| rs1906672 | 8 | 38130025 | Yes | Yes | A | G | 0.232 | 0.297 | 0.036 | 0.016 | 0.02 | -0.008 | 0.023 |
| rs2978456 | 8 | 42324765 | No | No | - | - | - | - | - | - | - | - | - |
| rs4873492 | 8 | 51947549 | Yes | Yes | T | C | 0.172 | 0.343 | 0.04 | -0.004 | 0.022 | 0.038 | 0.027 |
| rs6996733 | 8 | 60535824 | Yes | No | T | C | 0.849 | 0.211 | 0.043 | 0.006 | 0.023 | -0.002 | 0.028 |
| rs2354862 | 8 | 64501744 | Yes | No | A | C | 0.641 | 0.251 | 0.032 | 0.011 | 0.018 | 0.005 | 0.021 |
| rs13253358 | 8 | 68920135 | Yes | No | T | C | 0.298 | 0.213 | 0.033 | 0.014 | 0.019 | 0.038 | 0.022 |
| rs1350100 | 8 | 76054904 | Yes | No | A | G | 0.445 | 0.103 | 0.031 | -0.016 | 0.017 | 0.011 | 0.02 |
| rs1449544 | 8 | 76591880 | Yes | No | A | C | 0.544 | 0.212 | 0.03 | -0.002 | 0.017 | -0.033 | 0.02 |
| rs72688070 | 8 | 81393697 | Yes | No | T | C | 0.169 | -0.27 | 0.041 | -0.032 | 0.024 | 0.011 | 0.029 |
| rs56345595 | 8 | 82814156 | Yes | No | A | G | 0.585 | 0.207 | 0.031 | -0.001 | 0.017 | -0.036 | 0.02 |
| rs2142141 | 8 | 90940205 | No | No | - | - | - | - | - | - | - | - | - |
| rs7009170 | 8 | 92149429 | Yes | No | T | C | 0.319 | -0.215 | 0.032 | -0.009 | 0.018 | -0.042 | 0.021 |
| rs62526122 | 8 | 92769569 | Yes | No | A | G | 0.29 | 0.211 | 0.034 | 0.004 | 0.019 | -0.007 | 0.022 |
| rs4582532 | 8 | 95969257 | Yes | No | A | G | 0.506 | -0.2 | 0.03 | 0.001 | 0.017 | -0.007 | 0.02 |
| rs2978098 | 8 | 101676675 | Yes | No | A | C | 0.547 | 0.223 | 0.031 | -0.013 | 0.017 | -0.031 | 0.02 |
| rs142449193 | 8 | 102750597 | Yes | No | T | C | 0.046 | -0.455 | 0.074 | -0.052 | 0.046 | -0.005 | 0.054 |
| rs2513877 | 8 | 103883630 | Yes | No | A | G | 0.192 | -0.159 | 0.038 | 0.04 | 0.022 | 0.015 | 0.026 |
| rs35783704 | 8 | 105966258 | Yes | No | A | G | 0.104 | -0.462 | 0.051 | 0.043 | 0.026 | 0.022 | 0.031 |
| rs28499085 | 8 | 110107161 | Yes | No | A | G | 0.725 | 0.21 | 0.034 | 0.004 | 0.019 | 0.025 | 0.022 |
| rs2205260 | 8 | 116959837 | Yes | No | A | C | 0.168 | 0.067 | 0.04 | 0.012 | 0.022 | -0.017 | 0.026 |
| rs2071518 | 8 | 120435812 | Yes | No | T | C | 0.261 | 0.276 | 0.034 | 0.004 | 0.019 | 0.002 | 0.023 |
| rs62523863 | 8 | 126520544 | Yes | No | A | G | 0.22 | 0.269 | 0.037 | -0.028 | 0.02 | -0.003 | 0.024 |
| rs4598218 | 8 | 129483956 | Yes | Yes | T | C | 0.616 | 0.191 | 0.031 | 0.012 | 0.018 | 0.016 | 0.021 |
| rs894344 | 8 | 135612745 | Yes | No | A | G | 0.593 | -0.214 | 0.031 | -0.004 | 0.017 | -0.005 | 0.02 |
| rs4454254 | 8 | 141060027 | Yes | No | A | G | 0.631 | -0.218 | 0.031 | -0.006 | 0.018 | -0.024 | 0.021 |
| rs10087782 | 8 | 141858620 | Yes | No | T | C | 0.447 | 0.225 | 0.03 | -0.011 | 0.017 | 0.02 | 0.02 |
| rs34591516 | 8 | 142367087 | Yes | No | T | C | 0.049 | 0.486 | 0.071 | 0.022 | 0.035 | 0.041 | 0.041 |
| rs4129585 | 8 | 143312933 | Yes | Yes | A | C | 0.441 | 0.186 | 0.03 | 0.022 | 0.017 | 0.002 | 0.02 |
| rs62524579 | 8 | 144060955 | Yes | No | A | G | 0.533 | -0.268 | 0.032 | 0.01 | 0.017 | -0.007 | 0.02 |
| rs56233017 | 8 | 144981488 | Yes | No | A | G | 0.046 | -0.158 | 0.077 | 0.108 | 0.046 | 0.009 | 0.055 |
| rs520015 | 9 | 211762 | No | No | - | - | - | - | - | - | - | - | - |
| rs60191654 | 9 | 753648 | Yes | No | A | G | 0.812 | -0.238 | 0.038 | -0.033 | 0.022 | 0.012 | 0.026 |
| rs12216886 | 9 | 2493751 | Yes | No | T | G | 0.807 | 0.19 | 0.038 | 0.022 | 0.022 | 0 | 0.026 |
| rs28558845 | 9 | 4334791 | Yes | No | C | G | 0.155 | -0.257 | 0.042 | -0.028 | 0.025 | -0.009 | 0.03 |
| rs1332813 | 9 | 9350706 | Yes | No | T | C | 0.351 | 0.22 | 0.031 | 0.001 | 0.018 | 0.012 | 0.021 |
| rs35287509 | 9 | 10594635 | Yes | No | T | C | 0.663 | -0.164 | 0.032 | -0.007 | 0.018 | -0.005 | 0.022 |
| rs11789875 | 9 | 16872323 | Yes | No | A | G | 0.152 | 0.161 | 0.042 | 0.03 | 0.023 | 0.003 | 0.027 |
| rs4977492 | 9 | 19057551 | Yes | No | T | C | 0.662 | -0.124 | 0.032 | 0.015 | 0.018 | -0.022 | 0.021 |
| rs4364717 | 9 | 21801530 | Yes | No | A | G | 0.548 | -0.099 | 0.03 | -0.013 | 0.017 | -0.033 | 0.02 |
| rs9886665 | 9 | 22942770 | Yes | Yes | T | C | 0.267 | 0.205 | 0.034 | 0.015 | 0.019 | -0.005 | 0.023 |
| rs4553000 | 9 | 34223553 | Yes | No | T | C | 0.514 | -0.203 | 0.03 | 0.013 | 0.017 | 0.005 | 0.02 |
| rs76452347 | 9 | 35906471 | No | No | - | - | - | - | - | - | - | - | - |
| rs11139596 | 9 | 85128518 | Yes | No | T | G | 0.776 | -0.117 | 0.037 | -0.026 | 0.02 | -0.011 | 0.024 |
| rs11141731 | 9 | 89888472 | Yes | No | T | C | 0.227 | -0.194 | 0.036 | 0.024 | 0.02 | 0.004 | 0.024 |
| rs7045409 | 9 | 95201540 | Yes | No | A | T | 0.367 | -0.186 | 0.031 | 0 | 0.017 | -0.014 | 0.021 |
| rs10988442 | 9 | 101739709 | Yes | No | A | G | 0.619 | 0.148 | 0.031 | 0.026 | 0.017 | -0.003 | 0.021 |
| rs7020564 | 9 | 109670016 | Yes | No | A | T | 0.701 | -0.074 | 0.033 | 0.018 | 0.018 | 0.017 | 0.022 |
| rs7043304 | 9 | 112358150 | Yes | No | T | C | 0.855 | 0.198 | 0.043 | 0.017 | 0.024 | 0.031 | 0.029 |
| rs111245230 | 9 | 113169775 | Yes | No | T | C | 0.965 | -0.749 | 0.083 | -0.121 | 0.053 | 0.034 | 0.064 |
| rs13290326 | 9 | 116696625 | Yes | No | T | C | 0.501 | -0.212 | 0.03 | -0.008 | 0.017 | -0.024 | 0.02 |
| rs10982910 | 9 | 118534500 | Yes | No | T | G | 0.904 | -0.12 | 0.052 | -0.02 | 0.029 | -0.014 | 0.034 |
| rs1861881 | 9 | 119312256 | Yes | No | T | G | 0.316 | 0.128 | 0.032 | -0.009 | 0.018 | -0.012 | 0.022 |
| rs1953126 | 9 | 123640500 | Yes | No | T | C | 0.354 | 0.158 | 0.032 | -0.009 | 0.017 | -0.016 | 0.021 |
| rs10818775 | 9 | 125755571 | Yes | No | T | C | 0.121 | -0.302 | 0.046 | -0.039 | 0.025 | -0.033 | 0.03 |
| rs7861040 | 9 | 127044135 | Yes | No | C | G | 0.367 | -0.189 | 0.031 | -0.018 | 0.017 | 0.011 | 0.021 |
| rs72765298 | 9 | 127900996 | Yes | No | T | C | 0.875 | -0.368 | 0.046 | -0.026 | 0.027 | -0.061 | 0.032 |
| rs7023828 | 9 | 128498594 | Yes | No | T | C | 0.418 | -0.26 | 0.031 | 0.003 | 0.017 | 0.001 | 0.021 |
| rs1891730 | 9 | 130309028 | Yes | Yes | T | C | 0.617 | -0.181 | 0.031 | -0.014 | 0.017 | -0.027 | 0.021 |
| rs7869756 | 9 | 131210410 | Yes | No | A | G | 0.81 | -0.072 | 0.039 | -0.014 | 0.021 | 0.008 | 0.025 |
| rs184457 | 9 | 131940019 | Yes | No | A | G | 0.308 | -0.172 | 0.033 | -0.016 | 0.018 | -0.012 | 0.022 |
| rs687621 | 9 | 136137065 | Yes | No | A | G | 0.672 | 0.103 | 0.032 | -0.024 | 0.017 | -0.009 | 0.021 |
| rs6271 | 9 | 136522274 | No | No | - | - | - | - | - | - | - | - | - |
| rs11145807 | 9 | 139520789 | No | No | - | - | - | - | - | - | - | - | - |
| rs56352451 | 10 | 5804865 | Yes | No | T | C | 0.132 | 0.222 | 0.044 | 0.001 | 0.024 | 0.017 | 0.029 |
| rs36006409 | 10 | 10876943 | Yes | No | T | G | 0.794 | -0.159 | 0.038 | -0.011 | 0.021 | -0.028 | 0.025 |
| rs10906391 | 10 | 13523937 | Yes | No | T | C | 0.319 | 0.125 | 0.033 | -0.013 | 0.018 | -0.005 | 0.021 |
| rs4373814 | 10 | 18419972 | No | No | - | - | - | - | - | - | - | - | - |
| rs1813353 | 10 | 18707448 | Yes | No | T | C | 0.662 | 0.52 | 0.032 | -0.01 | 0.018 | -0.016 | 0.022 |
| rs11010905 | 10 | 19934813 | No | No | - | - | - | - | - | - | - | - | - |
| rs72795925 | 10 | 20531420 | Yes | No | T | C | 0.221 | 0.125 | 0.036 | 0.037 | 0.02 | 0.006 | 0.024 |
| rs10732433 | 10 | 21037294 | Yes | No | T | C | 0.421 | 0.155 | 0.031 | 0 | 0.017 | -0.003 | 0.021 |
| rs3802517 | 10 | 28233469 | No | No | - | - | - | - | - | - | - | - | - |
| rs1265842 | 10 | 28924901 | Yes | No | T | C | 0.484 | 0.128 | 0.03 | 0.013 | 0.017 | -0.016 | 0.02 |
| rs9337951 | 10 | 30317073 | No | No | - | - | - | - | - | - | - | - | - |
| rs11008355 | 10 | 31412561 | Yes | No | C | G | 0.239 | -0.056 | 0.035 | 0.002 | 0.02 | 0.037 | 0.024 |
| rs10826995 | 10 | 32082658 | Yes | No | T | C | 0.716 | -0.194 | 0.034 | -0.017 | 0.019 | -0.024 | 0.023 |
| rs76164690 | 10 | 32590362 | Yes | No | T | G | 0.86 | -0.197 | 0.044 | -0.031 | 0.024 | 0.006 | 0.029 |
| rs2246438 | 10 | 45273079 | Yes | No | A | G | 0.276 | -0.045 | 0.034 | -0.001 | 0.019 | 0.017 | 0.022 |
| rs34130368 | 10 | 48411796 | No | No | - | - | - | - | - | - | - | - | - |
| rs10761530 | 10 | 62390726 | Yes | No | T | C | 0.499 | 0.2 | 0.03 | 0.01 | 0.017 | 0.044 | 0.02 |
| rs1530440 | 10 | 63524591 | Yes | No | T | C | 0.188 | -0.567 | 0.039 | -0.01 | 0.022 | -0.008 | 0.026 |
| rs7090758 | 10 | 65335315 | Yes | No | T | C | 0.528 | -0.161 | 0.03 | -0.034 | 0.017 | 0.019 | 0.02 |
| rs7914287 | 10 | 69350563 | Yes | No | T | C | 0.779 | -0.189 | 0.037 | 0.029 | 0.02 | 0.045 | 0.024 |
| rs10823136 | 10 | 69855363 | Yes | No | T | C | 0.921 | -0.35 | 0.058 | 0.03 | 0.032 | 0.023 | 0.038 |
| rs10998362 | 10 | 70404159 | Yes | No | T | C | 0.317 | 0.107 | 0.034 | -0.068 | 0.018 | -0.039 | 0.022 |
| rs12572586 | 10 | 74751579 | Yes | No | T | C | 0.938 | -0.39 | 0.064 | -0.066 | 0.033 | 0.063 | 0.039 |
| rs10887914 | 10 | 82215288 | Yes | No | T | C | 0.462 | 0.201 | 0.03 | -0.008 | 0.017 | -0.019 | 0.02 |
| rs77413490 | 10 | 89681688 | Yes | No | T | G | 0.042 | 0.449 | 0.076 | 0.013 | 0.045 | 0.029 | 0.054 |
| rs11187142 | 10 | 94468685 | Yes | No | T | C | 0.105 | 0.331 | 0.05 | 0.024 | 0.028 | -0.032 | 0.034 |
| rs932764 | 10 | 95895940 | Yes | Yes | A | G | 0.569 | -0.43 | 0.031 | 0.026 | 0.017 | -0.027 | 0.02 |
| rs4494250 | 10 | 96563757 | Yes | No | A | G | 0.362 | 0.304 | 0.032 | -0.009 | 0.017 | 0.017 | 0.021 |
| rs603424 | 10 | 102075479 | Yes | No | A | G | 0.178 | 0.233 | 0.04 | 0.017 | 0.025 | 0.009 | 0.03 |
| rs112184198 | 10 | 102604514 | Yes | No | A | G | 0.105 | -0.663 | 0.05 | -0.029 | 0.029 | 0.009 | 0.034 |
| rs72847884 | 10 | 103115345 | Yes | No | A | G | 0.953 | 0.104 | 0.074 | 0.024 | 0.038 | 0.002 | 0.045 |
| rs11191156 | 10 | 103702763 | Yes | No | A | G | 0.648 | -0.155 | 0.032 | -0.019 | 0.018 | 0.031 | 0.021 |
| rs11191548 | 10 | 104846178 | Yes | Yes | T | C | 0.919 | 1.098 | 0.055 | -0.008 | 0.03 | -0.047 | 0.036 |
| rs4387287 | 10 | 105677897 | Yes | No | A | C | 0.165 | 0.228 | 0.041 | 0.011 | 0.024 | 0.02 | 0.028 |
| rs191784289 | 10 | 106894942 | Yes | No | T | C | 0.012 | 1.229 | 0.143 | 0.133 | 0.058 | -0.059 | 0.07 |
| rs111777102 | 10 | 111965826 | Yes | No | T | C | 0.066 | 0.291 | 0.062 | -0.012 | 0.038 | 0.035 | 0.045 |
| rs34872471 | 10 | 114754071 | Yes | No | T | C | 0.708 | -0.22 | 0.033 | 0.017 | 0.019 | -0.002 | 0.023 |
| rs2782980 | 10 | 115781527 | Yes | No | T | C | 0.284 | -0.425 | 0.034 | 0.011 | 0.019 | 0.027 | 0.022 |
| rs11197813 | 10 | 118523933 | Yes | Yes | A | G | 0.699 | -0.182 | 0.033 | 0.017 | 0.018 | -0.004 | 0.022 |
| rs72842207 | 10 | 121433675 | Yes | No | T | C | 0.214 | -0.203 | 0.037 | -0.002 | 0.02 | -0.021 | 0.024 |
| rs11592107 | 10 | 122968964 | Yes | Yes | A | G | 0.31 | 0.302 | 0.033 | 0 | 0.019 | -0.021 | 0.022 |
| rs72834453 | 10 | 124235226 | Yes | No | T | G | 0.876 | -0.325 | 0.046 | 0.025 | 0.027 | 0.006 | 0.032 |
| rs4411245 | 10 | 126712781 | Yes | No | A | G | 0.293 | 0.125 | 0.033 | -0.029 | 0.018 | 0.031 | 0.022 |
| rs7096563 | 10 | 133770229 | Yes | No | A | G | 0.355 | 0.21 | 0.032 | 0.021 | 0.018 | 0.011 | 0.021 |
| rs1133400 | 10 | 134459388 | Yes | Yes | A | G | 0.786 | -0.298 | 0.038 | 0.009 | 0.02 | -0.036 | 0.025 |
| rs7126805 | 11 | 828916 | Yes | No | A | G | 0.728 | 0.16 | 0.035 | 0.015 | 0.019 | -0.03 | 0.022 |
| rs661348 | 11 | 1905292 | Yes | Yes | T | C | 0.575 | -0.445 | 0.032 | -0.012 | 0.017 | -0.024 | 0.02 |
| rs17224476 | 11 | 4673788 | Yes | No | A | G | 0.111 | 0.208 | 0.048 | 0.006 | 0.03 | 0.021 | 0.035 |
| rs2929184 | 11 | 6289118 | Yes | No | A | G | 0.771 | 0.129 | 0.037 | 0.007 | 0.02 | -0.016 | 0.024 |
| rs110419 | 11 | 8252853 | Yes | No | A | G | 0.478 | 0.113 | 0.03 | -0.018 | 0.017 | 0.008 | 0.02 |
| rs10743086 | 11 | 8774923 | Yes | No | A | G | 0.207 | -0.205 | 0.037 | -0.019 | 0.021 | 0.007 | 0.025 |
| rs360153 | 11 | 9762274 | Yes | No | T | C | 0.417 | -0.344 | 0.031 | -0.009 | 0.017 | -0.028 | 0.021 |
| rs7129220 | 11 | 10350538 | Yes | Yes | A | G | 0.119 | 0.5 | 0.047 | 0.016 | 0.029 | 0.027 | 0.034 |
| rs900145 | 11 | 13293905 | Yes | No | T | C | 0.704 | 0.179 | 0.033 | 0.018 | 0.018 | -0.05 | 0.022 |
| rs4757391 | 11 | 16302939 | Yes | No | T | C | 0.798 | -0.511 | 0.038 | -0.013 | 0.021 | 0.016 | 0.025 |
| rs381815 | 11 | 16902268 | Yes | No | T | C | 0.275 | 0.301 | 0.034 | 0.006 | 0.019 | 0.006 | 0.023 |
| rs5219 | 11 | 17409572 | Yes | No | T | C | 0.366 | 0.347 | 0.031 | 0.019 | 0.017 | 0.019 | 0.021 |
| rs10766533 | 11 | 19224677 | Yes | Yes | A | T | 0.711 | 0.21 | 0.034 | 0.011 | 0.019 | -0.007 | 0.023 |
| rs11026586 | 11 | 22515533 | Yes | No | A | G | 0.07 | 0.434 | 0.06 | -0.042 | 0.032 | 0.016 | 0.039 |
| rs11030119 | 11 | 27728102 | Yes | No | A | G | 0.301 | -0.222 | 0.033 | 0.019 | 0.018 | 0.021 | 0.022 |
| rs871004 | 11 | 28512458 | Yes | Yes | A | G | 0.348 | 0.234 | 0.032 | -0.02 | 0.018 | 0.008 | 0.021 |
| rs11031051 | 11 | 30355707 | Yes | No | A | C | 0.69 | -0.224 | 0.033 | 0.01 | 0.018 | -0.03 | 0.022 |
| rs919045 | 11 | 31111810 | Yes | No | T | C | 0.628 | 0.17 | 0.031 | -0.003 | 0.017 | -0.004 | 0.021 |
| rs4922591 | 11 | 32374199 | Yes | No | T | C | 0.387 | -0.189 | 0.031 | 0.034 | 0.017 | 0.009 | 0.021 |
| rs190194639 | 11 | 34068037 | Yes | No | T | C | 0.077 | 0.302 | 0.058 | -0.005 | 0.028 | 0.062 | 0.034 |
| rs7480089 | 11 | 45207851 | Yes | No | A | G | 0.118 | -0.261 | 0.047 | -0.049 | 0.027 | -0.035 | 0.032 |
| rs1585453 | 11 | 46884713 | Yes | No | A | T | 0.887 | -0.382 | 0.049 | -0.02 | 0.032 | 0.001 | 0.038 |
| rs7103648 | 11 | 47461783 | Yes | No | A | G | 0.613 | -0.386 | 0.031 | 0.002 | 0.018 | 0.002 | 0.021 |
| rs11537751 | 11 | 47587452 | Yes | No | T | C | 0.055 | 0.44 | 0.067 | 0.028 | 0.039 | -0.007 | 0.047 |
| rs2688716 | 11 | 54835623 | No | No | - | - | - | - | - | - | - | - | - |
| rs75905900 | 11 | 55113534 | Yes | No | A | C | 0.866 | 0.417 | 0.045 | 0.012 | 0.024 | 0.007 | 0.028 |
| rs11607056 | 11 | 57496820 | Yes | No | T | C | 0.328 | -0.251 | 0.032 | -0.002 | 0.018 | 0.003 | 0.021 |
| rs11229457 | 11 | 58207203 | Yes | Yes | T | C | 0.213 | -0.317 | 0.037 | 0.028 | 0.019 | -0.018 | 0.023 |
| rs751984 | 11 | 61278246 | Yes | No | T | C | 0.882 | 0.441 | 0.048 | -0.023 | 0.024 | -0.075 | 0.029 |
| rs4980515 | 11 | 63744609 | Yes | No | T | C | 0.499 | 0.225 | 0.03 | 0 | 0.017 | -0.007 | 0.02 |
| rs3741378 | 11 | 65408937 | Yes | Yes | T | C | 0.135 | -0.409 | 0.045 | 0.036 | 0.025 | 0.008 | 0.03 |
| rs67976715 | 11 | 68023742 | Yes | No | C | G | 0.234 | 0.208 | 0.036 | 0.015 | 0.02 | 0.066 | 0.024 |
| rs67330701 | 11 | 69079707 | Yes | No | T | C | 0.094 | -0.391 | 0.056 | 0.019 | 0.03 | 0.064 | 0.036 |
| rs875106 | 11 | 70005641 | Yes | No | A | G | 0.522 | -0.082 | 0.03 | -0.012 | 0.017 | 0.013 | 0.02 |
| rs504217 | 11 | 72006086 | Yes | No | T | C | 0.074 | 0.386 | 0.058 | 0.067 | 0.034 | 0.018 | 0.04 |
| rs2298807 | 11 | 73068571 | Yes | No | T | C | 0.784 | 0.15 | 0.037 | 0.012 | 0.02 | 0.033 | 0.024 |
| rs4420291 | 11 | 74374950 | Yes | No | A | G | 0.506 | 0.074 | 0.03 | -0.006 | 0.017 | 0.002 | 0.02 |
| rs7927515 | 11 | 76125330 | Yes | Yes | A | C | 0.346 | 0.227 | 0.032 | -0.038 | 0.018 | 0.012 | 0.021 |
| rs59986178 | 11 | 77359909 | Yes | No | C | G | 0.104 | 0.128 | 0.052 | -0.031 | 0.027 | -0.015 | 0.032 |
| rs2450128 | 11 | 77940075 | Yes | No | A | G | 0.154 | -0.173 | 0.042 | 0.032 | 0.024 | 0.045 | 0.029 |
| rs2289125 | 11 | 89224453 | Yes | No | A | C | 0.22 | -0.259 | 0.038 | 0.014 | 0.02 | 0.018 | 0.024 |
| rs10830963 | 11 | 92708710 | Yes | No | C | G | 0.724 | -0.132 | 0.035 | -0.028 | 0.019 | 0.024 | 0.023 |
| rs11021221 | 11 | 95308854 | Yes | No | A | T | 0.166 | 0.002 | 0.041 | 0.003 | 0.022 | 0.023 | 0.027 |
| rs633185 | 11 | 100593538 | Yes | No | C | G | 0.713 | 0.637 | 0.033 | -0.039 | 0.018 | -0.029 | 0.022 |
| rs61892344 | 11 | 101100768 | Yes | No | T | C | 0.174 | -0.095 | 0.04 | -0.032 | 0.024 | -0.02 | 0.029 |
| rs12807220 | 11 | 102077200 | Yes | No | A | G | 0.359 | -0.072 | 0.032 | 0.015 | 0.018 | 0.004 | 0.022 |
| rs4754196 | 11 | 107096777 | Yes | No | A | G | 0.522 | -0.349 | 0.03 | 0.022 | 0.017 | -0.046 | 0.02 |
| rs12362593 | 11 | 111586091 | Yes | No | C | G | 0.729 | -0.2 | 0.035 | 0.011 | 0.019 | -0.006 | 0.023 |
| rs17119370 | 11 | 116097136 | Yes | Yes | A | T | 0.693 | 0.221 | 0.033 | -0.02 | 0.019 | -0.022 | 0.023 |
| rs1076485 | 11 | 116772441 | Yes | No | T | C | 0.13 | 0.339 | 0.046 | 0.008 | 0.025 | -0.002 | 0.03 |
| rs8258 | 11 | 117283676 | Yes | No | T | C | 0.374 | 0.165 | 0.031 | 0.002 | 0.017 | 0.013 | 0.021 |
| rs12574332 | 11 | 122521123 | Yes | No | T | C | 0.123 | 0.245 | 0.046 | 0.014 | 0.025 | 0.019 | 0.029 |
| rs11222386 | 11 | 130779068 | Yes | No | C | G | 0.197 | 0.153 | 0.038 | -0.022 | 0.022 | 0.029 | 0.026 |
| rs78998485 | 12 | 434755 | Yes | No | C | G | 0.744 | -0.245 | 0.035 | -0.013 | 0.02 | 0.037 | 0.024 |
| rs11571376 | 12 | 1059556 | Yes | No | C | G | 0.706 | -0.18 | 0.033 | 0.007 | 0.018 | 0.008 | 0.022 |
| rs55935819 | 12 | 2521579 | Yes | No | A | G | 0.363 | 0.174 | 0.031 | -0.011 | 0.018 | -0.005 | 0.021 |
| rs117233107 | 12 | 4328521 | Yes | No | A | G | 0.016 | -0.637 | 0.137 | 0.021 | 0.065 | 0.03 | 0.077 |
| rs75507123 | 12 | 5417856 | Yes | No | T | G | 0.128 | -0.232 | 0.045 | -0.004 | 0.027 | -0.034 | 0.032 |
| rs7132012 | 12 | 8832203 | Yes | No | A | G | 0.675 | 0.2 | 0.032 | -0.003 | 0.018 | 0.015 | 0.022 |
| rs2024385 | 12 | 12888438 | No | No | - | - | - | - | - | - | - | - | - |
| rs28621435 | 12 | 13860990 | Yes | No | A | G | 0.115 | -0.298 | 0.048 | -0.005 | 0.03 | 0.035 | 0.035 |
| rs7313556 | 12 | 15297359 | Yes | No | A | G | 0.348 | -0.048 | 0.031 | -0.02 | 0.018 | 0.015 | 0.022 |
| rs61912333 | 12 | 19554817 | No | No | - | - | - | - | - | - | - | - | - |
| rs12579720 | 12 | 20173764 | Yes | No | C | G | 0.243 | -0.258 | 0.035 | -0.006 | 0.02 | 0.006 | 0.024 |
| rs73080726 | 12 | 20754154 | Yes | No | T | C | 0.102 | -0.244 | 0.05 | -0.005 | 0.025 | -0.053 | 0.03 |
| rs704191 | 12 | 22015022 | Yes | No | T | C | 0.463 | 0.153 | 0.03 | -0.009 | 0.017 | -0.015 | 0.02 |
| rs7976167 | 12 | 24210599 | Yes | Yes | T | C | 0.689 | 0.178 | 0.032 | 0.001 | 0.018 | 0.004 | 0.022 |
| rs17287293 | 12 | 24770878 | Yes | No | A | G | 0.851 | -0.07 | 0.042 | -0.019 | 0.024 | -0.015 | 0.028 |
| rs6487543 | 12 | 26438189 | Yes | No | A | G | 0.77 | 0.19 | 0.037 | -0.005 | 0.02 | -0.009 | 0.024 |
| rs1098708 | 12 | 27321112 | Yes | No | A | G | 0.542 | -0.197 | 0.03 | -0.004 | 0.017 | -0.032 | 0.02 |
| rs10842991 | 12 | 27962103 | Yes | No | T | C | 0.198 | -0.091 | 0.038 | 0.009 | 0.021 | -0.019 | 0.025 |
| rs7965392 | 12 | 42540280 | Yes | No | A | G | 0.387 | 0.094 | 0.031 | -0.008 | 0.017 | 0.023 | 0.021 |
| rs11168245 | 12 | 48204499 | No | No | - | - | - | - | - | - | - | - | - |
| rs2261608 | 12 | 48721634 | Yes | No | A | T | 0.351 | 0.172 | 0.031 | 0.009 | 0.018 | 0.013 | 0.021 |
| rs1126930 | 12 | 49399132 | Yes | No | C | G | 0.035 | 0.638 | 0.086 | 0.015 | 0.047 | -0.112 | 0.055 |
| rs7977389 | 12 | 49981722 | Yes | No | T | C | 0.894 | 0.258 | 0.049 | -0.024 | 0.026 | 0.01 | 0.032 |
| rs7302981 | 12 | 50537815 | Yes | No | A | G | 0.379 | 0.376 | 0.031 | 0.014 | 0.018 | 0.006 | 0.021 |
| rs61926181 | 12 | 50767037 | Yes | No | A | G | 0.038 | -0.647 | 0.085 | 0.006 | 0.043 | -0.02 | 0.05 |
| rs73099903 | 12 | 53440779 | Yes | No | T | C | 0.082 | 0.469 | 0.056 | 0.005 | 0.03 | 0.038 | 0.035 |
| rs7297416 | 12 | 54443090 | Yes | Yes | A | C | 0.696 | 0.376 | 0.033 | -0.017 | 0.018 | -0.023 | 0.021 |
| rs7137749 | 12 | 57098040 | Yes | No | T | C | 0.368 | 0.078 | 0.031 | -0.008 | 0.018 | 0.009 | 0.021 |
| rs10437954 | 12 | 58003922 | Yes | No | A | G | 0.907 | -0.41 | 0.053 | 0.018 | 0.028 | 0.008 | 0.034 |
| rs4143175 | 12 | 67782397 | Yes | Yes | T | C | 0.241 | 0.219 | 0.035 | 0.022 | 0.02 | 0.009 | 0.024 |
| rs513177 | 12 | 69950545 | No | No | - | - | - | - | - | - | - | - | - |
| rs7963801 | 12 | 79685226 | No | No | - | - | - | - | - | - | - | - | - |
| rs17249754 | 12 | 90060586 | Yes | Yes | A | G | 0.168 | -0.845 | 0.04 | 0.053 | 0.024 | 0.025 | 0.029 |
| rs10858966 | 12 | 90567026 | Yes | No | C | G | 0.299 | 0.256 | 0.033 | -0.006 | 0.018 | -0.009 | 0.022 |
| rs76785029 | 12 | 94882905 | Yes | No | T | C | 0.079 | -0.19 | 0.058 | 0.009 | 0.032 | 0.026 | 0.039 |
| rs7977311 | 12 | 95487226 | Yes | No | T | C | 0.115 | -0.23 | 0.047 | -0.012 | 0.027 | 0.012 | 0.033 |
| rs11108209 | 12 | 96109855 | Yes | No | T | C | 0.907 | -0.185 | 0.052 | -0.036 | 0.031 | 0.029 | 0.037 |
| rs7134060 | 12 | 96717095 | Yes | No | A | G | 0.446 | -0.111 | 0.03 | 0.011 | 0.017 | 0.024 | 0.02 |
| rs10778174 | 12 | 102838996 | Yes | No | A | G | 0.248 | -0.221 | 0.035 | 0.02 | 0.02 | -0.018 | 0.024 |
| rs11112548 | 12 | 105871914 | Yes | Yes | A | T | 0.956 | 0.509 | 0.077 | 0.008 | 0.042 | 0.009 | 0.051 |
| rs12184466 | 12 | 111281636 | No | No | - | - | - | - | - | - | - | - | - |
| rs3184504 | 12 | 111884608 | Yes | No | T | C | 0.48 | 0.578 | 0.03 | -0.006 | 0.017 | -0.028 | 0.02 |
| rs10850411 | 12 | 115387796 | Yes | No | T | C | 0.697 | 0.291 | 0.033 | 0.011 | 0.019 | 0.011 | 0.022 |
| rs35444 | 12 | 115552437 | Yes | No | A | G | 0.614 | 0.437 | 0.031 | -0.008 | 0.017 | 0.011 | 0.02 |
| rs11067763 | 12 | 116198341 | Yes | No | A | G | 0.898 | 0.307 | 0.05 | -0.01 | 0.027 | 0.04 | 0.032 |
| rs11615689 | 12 | 116699675 | Yes | No | T | C | 0.82 | -0.151 | 0.039 | -0.009 | 0.022 | 0.043 | 0.026 |
| rs3898618 | 12 | 120813921 | Yes | No | T | C | 0.945 | -0.309 | 0.066 | 0.013 | 0.037 | -0.037 | 0.044 |
| rs28498002 | 12 | 122599796 | Yes | No | T | C | 0.475 | -0.16 | 0.031 | -0.005 | 0.017 | 0.033 | 0.02 |
| rs1060105 | 12 | 123806219 | Yes | No | T | C | 0.203 | -0.102 | 0.038 | 0.018 | 0.021 | 0.023 | 0.024 |
| rs1271309 | 12 | 124820705 | Yes | No | A | G | 0.162 | -0.161 | 0.042 | -0.037 | 0.023 | -0.038 | 0.027 |
| rs117206641 | 12 | 133086888 | Yes | No | T | C | 0.111 | 0.315 | 0.05 | -0.013 | 0.027 | 0.015 | 0.032 |
| rs2480171 | 13 | 21559858 | Yes | No | T | C | 0.122 | 0.291 | 0.047 | -0.065 | 0.026 | -0.028 | 0.031 |
| rs606950 | 13 | 22298923 | Yes | No | A | G | 0.616 | 0.27 | 0.031 | 0.003 | 0.017 | 0.032 | 0.021 |
| rs55641580 | 13 | 25257917 | Yes | No | T | C | 0.126 | 0.268 | 0.046 | -0.02 | 0.025 | -0.034 | 0.03 |
| rs1331012 | 13 | 27115424 | Yes | No | T | G | 0.27 | 0.204 | 0.034 | -0.012 | 0.019 | -0.043 | 0.023 |
| rs63418562 | 13 | 30146201 | No | No | - | - | - | - | - | - | - | - | - |
| rs9532243 | 13 | 32191408 | Yes | No | A | C | 0.484 | 0.224 | 0.03 | 0.014 | 0.017 | -0.03 | 0.02 |
| rs9549297 | 13 | 41397482 | Yes | No | A | G | 0.821 | -0.11 | 0.04 | -0.02 | 0.022 | 0.006 | 0.026 |
| rs4274337 | 13 | 41967193 | Yes | No | A | G | 0.17 | -0.297 | 0.041 | 0.038 | 0.023 | -0.013 | 0.027 |
| rs73187288 | 13 | 42738672 | Yes | No | A | C | 0.891 | -0.277 | 0.048 | -0.012 | 0.027 | 0.015 | 0.032 |
| rs912434 | 13 | 47189928 | Yes | Yes | T | G | 0.759 | 0.234 | 0.035 | -0.013 | 0.019 | 0.018 | 0.022 |
| rs12583615 | 13 | 50564085 | Yes | No | A | G | 0.132 | 0.146 | 0.045 | -0.04 | 0.025 | -0.01 | 0.03 |
| rs9526707 | 13 | 51489186 | Yes | No | A | G | 0.322 | -0.204 | 0.032 | -0.033 | 0.018 | -0.017 | 0.022 |
| rs75961402 | 13 | 56398286 | Yes | No | A | G | 0.153 | 0.266 | 0.042 | -0.013 | 0.024 | 0.012 | 0.029 |
| rs9563529 | 13 | 58316637 | Yes | No | T | G | 0.204 | 0.186 | 0.037 | 0.017 | 0.021 | 0.032 | 0.025 |
| rs3861113 | 13 | 72364382 | Yes | No | A | C | 0.084 | 0.223 | 0.056 | -0.021 | 0.029 | -0.004 | 0.034 |
| rs78474310 | 13 | 73826901 | Yes | No | A | G | 0.955 | -0.47 | 0.073 | -0.031 | 0.039 | -0.018 | 0.046 |
| rs4304924 | 13 | 79238925 | Yes | No | A | G | 0.567 | -0.093 | 0.031 | -0.005 | 0.017 | -0.008 | 0.02 |
| rs7988232 | 13 | 79808655 | Yes | No | A | G | 0.411 | 0.152 | 0.031 | -0.017 | 0.017 | -0.051 | 0.02 |
| rs1215469 | 13 | 80707408 | Yes | No | A | C | 0.23 | -0.212 | 0.036 | -0.004 | 0.02 | 0.012 | 0.025 |
| rs55684003 | 13 | 97988689 | Yes | No | A | G | 0.696 | 0.117 | 0.033 | -0.009 | 0.018 | 0.015 | 0.022 |
| rs3742182 | 13 | 111375132 | Yes | No | T | C | 0.81 | -0.206 | 0.038 | 0.007 | 0.022 | 0.001 | 0.026 |
| rs9549328 | 13 | 113636156 | Yes | No | T | C | 0.23 | 0.292 | 0.036 | -0.012 | 0.02 | 0.004 | 0.024 |
| rs7331680 | 13 | 115000650 | Yes | No | T | G | 0.149 | 0.41 | 0.042 | -0.003 | 0.024 | 0.009 | 0.028 |
| rs17880989 | 14 | 23313633 | No | No | - | - | - | - | - | - | - | - | - |
| rs452036 | 14 | 23865885 | Yes | No | A | G | 0.355 | -0.222 | 0.032 | -0.022 | 0.018 | 0.005 | 0.022 |
| rs17115145 | 14 | 30122409 | Yes | Yes | T | C | 0.397 | 0.178 | 0.031 | 0 | 0.017 | 0.039 | 0.021 |
| rs4424827 | 14 | 35110857 | Yes | No | T | C | 0.567 | -0.127 | 0.03 | 0.008 | 0.017 | 0.036 | 0.021 |
| rs8904 | 14 | 35871217 | Yes | No | A | G | 0.368 | 0.306 | 0.031 | 0.007 | 0.018 | -0.06 | 0.021 |
| rs34983854 | 14 | 39858442 | Yes | No | A | G | 0.603 | -0.206 | 0.031 | -0.016 | 0.017 | -0.007 | 0.02 |
| rs72683923 | 14 | 50735947 | No | No | - | - | - | - | - | - | - | - | - |
| rs9888615 | 14 | 53377540 | Yes | Yes | T | C | 0.291 | -0.274 | 0.033 | 0.027 | 0.019 | 0.034 | 0.023 |
| rs210381 | 14 | 54107791 | Yes | No | A | G | 0.565 | -0.145 | 0.03 | 0.001 | 0.017 | -0.033 | 0.02 |
| rs7144602 | 14 | 55285588 | Yes | No | T | G | 0.649 | -0.158 | 0.032 | 0.005 | 0.018 | 0.036 | 0.021 |
| rs11628933 | 14 | 60700903 | Yes | No | C | G | 0.234 | -0.102 | 0.036 | -0.012 | 0.02 | -0.008 | 0.024 |
| rs731681 | 14 | 68010224 | No | No | - | - | - | - | - | - | - | - | - |
| rs57786342 | 14 | 69260028 | Yes | No | A | G | 0.206 | 0.232 | 0.037 | -0.031 | 0.021 | 0.01 | 0.026 |
| rs11623535 | 14 | 72462381 | Yes | Yes | A | G | 0.744 | 0.21 | 0.034 | -0.001 | 0.019 | 0.012 | 0.023 |
| rs4903064 | 14 | 73279420 | Yes | No | T | C | 0.764 | -0.028 | 0.036 | 0.025 | 0.021 | 0.001 | 0.025 |
| rs11159091 | 14 | 75074316 | Yes | Yes | A | G | 0.462 | 0.198 | 0.03 | 0.004 | 0.017 | -0.002 | 0.02 |
| rs11627326 | 14 | 85785251 | Yes | No | C | G | 0.287 | 0.094 | 0.033 | -0.009 | 0.019 | 0.005 | 0.023 |
| rs4904503 | 14 | 89565130 | Yes | No | T | C | 0.298 | 0.088 | 0.033 | 0.004 | 0.018 | -0.017 | 0.022 |
| rs11160085 | 14 | 93112102 | Yes | No | T | C | 0.704 | 0.156 | 0.034 | -0.021 | 0.019 | 0.063 | 0.022 |
| rs8013933 | 14 | 94465789 | Yes | No | T | C | 0.691 | 0.203 | 0.033 | 0.016 | 0.018 | -0.003 | 0.022 |
| rs9323988 | 14 | 98587630 | Yes | No | T | C | 0.615 | -0.249 | 0.031 | -0.008 | 0.018 | -0.001 | 0.021 |
| rs1475130 | 14 | 100225144 | Yes | No | T | C | 0.346 | -0.198 | 0.032 | -0.016 | 0.018 | -0.013 | 0.021 |
| rs28470843 | 14 | 100742658 | Yes | No | T | C | 0.597 | 0.118 | 0.031 | 0.003 | 0.017 | 0.014 | 0.02 |
| rs11626434 | 14 | 101998443 | Yes | No | C | G | 0.362 | -0.109 | 0.032 | 0.024 | 0.017 | 0.005 | 0.021 |
| rs8014182 | 14 | 103859962 | Yes | No | T | C | 0.132 | -0.334 | 0.044 | -0.021 | 0.024 | 0.034 | 0.029 |
| rs34161718 | 14 | 104620193 | No | No | - | - | - | - | - | - | - | - | - |
| rs10873612 | 15 | 26105602 | Yes | No | T | C | 0.596 | -0.105 | 0.031 | -0.002 | 0.017 | 0.004 | 0.021 |
| rs11629850 | 15 | 40317075 | Yes | No | A | G | 0.529 | 0.23 | 0.03 | 0.009 | 0.017 | -0.017 | 0.02 |
| rs2925345 | 15 | 41311799 | Yes | No | T | C | 0.468 | 0.274 | 0.03 | -0.003 | 0.017 | 0.029 | 0.02 |
| rs4924570 | 15 | 41974660 | Yes | No | T | C | 0.629 | -0.211 | 0.031 | 0.008 | 0.017 | -0.034 | 0.021 |
| rs1036477 | 15 | 48914926 | Yes | No | A | G | 0.897 | 0.384 | 0.049 | 0.007 | 0.028 | -0.026 | 0.034 |
| rs3098186 | 15 | 50810621 | Yes | No | T | C | 0.516 | -0.242 | 0.03 | -0.004 | 0.017 | -0.011 | 0.02 |
| rs3191402 | 15 | 59429160 | No | No | - | - | - | - | - | - | - | - | - |
| rs956006 | 15 | 62808539 | Yes | No | T | C | 0.331 | -0.096 | 0.032 | -0.019 | 0.018 | 0.002 | 0.022 |
| rs832890 | 15 | 65166309 | Yes | No | T | C | 0.465 | 0.094 | 0.03 | -0.013 | 0.017 | 0.003 | 0.02 |
| rs7178615 | 15 | 66869072 | Yes | No | A | G | 0.377 | -0.165 | 0.031 | 0.011 | 0.017 | 0.006 | 0.021 |
| rs2289261 | 15 | 67457485 | Yes | No | C | G | 0.652 | -0.11 | 0.032 | -0.031 | 0.018 | -0.017 | 0.021 |
| rs62004794 | 15 | 68454523 | Yes | No | A | G | 0.439 | -0.097 | 0.03 | 0.004 | 0.017 | 0.037 | 0.02 |
| rs11853359 | 15 | 71621524 | Yes | No | A | G | 0.332 | -0.024 | 0.032 | 0.005 | 0.018 | 0.016 | 0.022 |
| rs61653296 | 15 | 74557817 | Yes | No | A | G | 0.801 | -0.184 | 0.038 | -0.014 | 0.021 | 0.005 | 0.025 |
| rs1378942 | 15 | 75077367 | Yes | No | A | C | 0.668 | -0.508 | 0.032 | -0.004 | 0.018 | 0.048 | 0.021 |
| rs11634028 | 15 | 76276150 | Yes | No | A | T | 0.214 | 0.191 | 0.038 | 0.031 | 0.021 | 0.048 | 0.026 |
| rs62011052 | 15 | 79156983 | Yes | No | T | C | 0.849 | -0.11 | 0.042 | 0.038 | 0.023 | -0.043 | 0.028 |
| rs2759308 | 15 | 81016227 | Yes | Yes | A | G | 0.475 | 0.316 | 0.03 | 0.022 | 0.017 | -0.009 | 0.02 |
| rs2034618 | 15 | 83799632 | Yes | No | T | C | 0.222 | 0.01 | 0.036 | 0.001 | 0.02 | 0.006 | 0.024 |
| rs899927 | 15 | 84561879 | Yes | No | T | C | 0.318 | 0.084 | 0.033 | 0.001 | 0.018 | 0.002 | 0.022 |
| rs7180952 | 15 | 85162551 | Yes | No | T | C | 0.538 | -0.072 | 0.03 | -0.029 | 0.017 | 0.002 | 0.02 |
| rs3743157 | 15 | 85680532 | Yes | Yes | A | C | 0.167 | 0.293 | 0.04 | -0.05 | 0.023 | 0.001 | 0.028 |
| rs11632436 | 15 | 86295286 | No | No | - | - | - | - | - | - | - | - | - |
| rs28611491 | 15 | 90641809 | Yes | No | T | C | 0.081 | 0.334 | 0.058 | 0.027 | 0.034 | 0.028 | 0.04 |
| rs2521501 | 15 | 91437388 | No | No | - | - | - | - | - | - | - | - | - |
| rs873122 | 15 | 92702020 | Yes | No | C | G | 0.72 | 0.137 | 0.034 | 0.012 | 0.019 | -0.004 | 0.023 |
| rs11632112 | 15 | 93468276 | Yes | No | C | G | 0.24 | -0.177 | 0.035 | 0.017 | 0.019 | 0.04 | 0.023 |
| rs12906962 | 15 | 95312071 | Yes | No | T | C | 0.676 | -0.265 | 0.032 | -0.031 | 0.018 | -0.011 | 0.022 |
| rs4984496 | 15 | 96635898 | Yes | No | T | G | 0.336 | 0.074 | 0.032 | 0.02 | 0.018 | -0.003 | 0.022 |
| rs34756251 | 15 | 100192540 | Yes | No | T | C | 0.171 | -0.262 | 0.04 | -0.044 | 0.023 | 0.022 | 0.027 |
| rs9932866 | 16 | 706067 | Yes | No | A | G | 0.367 | 0.16 | 0.032 | -0.011 | 0.017 | -0.011 | 0.021 |
| rs11248862 | 16 | 1344291 | Yes | No | A | G | 0.125 | 0.28 | 0.046 | 0.007 | 0.027 | 0.097 | 0.033 |
| rs28590346 | 16 | 2080653 | Yes | No | A | T | 0.66 | -0.271 | 0.033 | -0.005 | 0.018 | 0.01 | 0.021 |
| rs2379829 | 16 | 3538873 | Yes | Yes | C | G | 0.73 | -0.268 | 0.034 | 0.021 | 0.019 | 0.012 | 0.023 |
| rs4785955 | 16 | 4297651 | Yes | No | T | G | 0.218 | 0.138 | 0.038 | 0.029 | 0.02 | 0.002 | 0.024 |
| rs12921187 | 16 | 4943019 | Yes | No | T | G | 0.428 | -0.292 | 0.03 | -0.016 | 0.017 | -0.007 | 0.02 |
| rs35450617 | 16 | 6889675 | Yes | No | T | G | 0.699 | -0.177 | 0.033 | -0.023 | 0.018 | -0.015 | 0.022 |
| rs11642631 | 16 | 11198835 | Yes | No | T | C | 0.562 | -0.094 | 0.03 | -0.044 | 0.017 | -0.006 | 0.02 |
| rs57327054 | 16 | 14487036 | Yes | No | T | C | 0.308 | -0.076 | 0.033 | -0.01 | 0.019 | 0.002 | 0.022 |
| rs3915425 | 16 | 15912544 | Yes | No | T | C | 0.682 | 0.206 | 0.032 | 0.016 | 0.018 | -0.015 | 0.022 |
| rs4782211 | 16 | 19152219 | No | No | - | - | - | - | - | - | - | - | - |
| rs13333226 | 16 | 20365654 | Yes | No | A | G | 0.816 | 0.377 | 0.039 | 0.003 | 0.022 | 0.04 | 0.027 |
| rs11639856 | 16 | 24788645 | Yes | No | A | T | 0.193 | -0.32 | 0.038 | 0.025 | 0.021 | 0.005 | 0.025 |
| rs6565174 | 16 | 30111904 | Yes | No | A | C | 0.112 | -0.288 | 0.048 | 0.053 | 0.027 | -0.018 | 0.033 |
| rs72799341 | 16 | 30936743 | Yes | No | A | G | 0.24 | 0.057 | 0.035 | 0 | 0.02 | 0.008 | 0.024 |
| rs10468291 | 16 | 49768046 | Yes | No | A | C | 0.569 | -0.187 | 0.03 | 0.013 | 0.017 | -0.002 | 0.02 |
| rs34941092 | 16 | 50550137 | Yes | No | A | G | 0.15 | -0.322 | 0.043 | -0.022 | 0.024 | -0.015 | 0.028 |
| rs9932220 | 16 | 51758116 | Yes | No | A | G | 0.218 | -0.229 | 0.036 | 0.01 | 0.02 | 0.014 | 0.023 |
| rs37060 | 16 | 58566304 | Yes | No | A | G | 0.247 | 0.11 | 0.035 | -0.01 | 0.02 | 0.05 | 0.024 |
| rs28633979 | 16 | 65282820 | Yes | No | A | C | 0.43 | -0.179 | 0.03 | -0.016 | 0.017 | -0.013 | 0.02 |
| rs45474499 | 16 | 66914492 | Yes | No | T | C | 0.047 | 0.443 | 0.072 | 0.002 | 0.034 | 0.06 | 0.041 |
| rs7185555 | 16 | 69131281 | Yes | No | C | G | 0.153 | -0.013 | 0.042 | 0.001 | 0.023 | 0.009 | 0.028 |
| rs33063 | 16 | 69640217 | Yes | No | A | G | 0.148 | 0.224 | 0.042 | 0.037 | 0.023 | 0.018 | 0.027 |
| rs62053102 | 16 | 71654365 | Yes | No | A | T | 0.953 | -0.569 | 0.075 | -0.031 | 0.039 | -0.048 | 0.047 |
| rs1012089 | 16 | 74171973 | No | No | - | - | - | - | - | - | - | - | - |
| rs35261357 | 16 | 75444572 | Yes | No | T | C | 0.585 | 0.35 | 0.031 | -0.041 | 0.017 | -0.014 | 0.021 |
| rs56844452 | 16 | 80864776 | Yes | No | T | C | 0.071 | -0.451 | 0.06 | 0.059 | 0.034 | -0.014 | 0.042 |
| rs8059962 | 16 | 81574197 | Yes | No | T | C | 0.421 | -0.197 | 0.031 | -0.009 | 0.017 | 0.022 | 0.02 |
| rs7500448 | 16 | 83045790 | Yes | No | A | G | 0.747 | 0.223 | 0.035 | 0.002 | 0.02 | 0.025 | 0.024 |
| rs7187540 | 16 | 85318302 | No | No | - | - | - | - | - | - | - | - | - |
| rs3851018 | 16 | 86437811 | No | No | - | - | - | - | - | - | - | - | - |
| rs6540125 | 16 | 87993889 | Yes | No | T | G | 0.341 | 0.204 | 0.032 | -0.016 | 0.018 | -0.011 | 0.021 |
| rs1126464 | 16 | 89704365 | Yes | No | C | G | 0.243 | 0.128 | 0.036 | 0.004 | 0.019 | 0.042 | 0.023 |
| rs12941318 | 17 | 1333598 | Yes | No | T | C | 0.496 | -0.235 | 0.031 | 0.007 | 0.017 | -0.023 | 0.02 |
| rs4480845 | 17 | 1958609 | Yes | No | T | C | 0.365 | 0.316 | 0.032 | 0.011 | 0.018 | 0.027 | 0.021 |
| rs7215084 | 17 | 3880148 | Yes | No | T | C | 0.513 | 0.095 | 0.03 | 0.016 | 0.017 | 0.017 | 0.02 |
| rs28427409 | 17 | 6473882 | Yes | No | T | C | 0.417 | 0.219 | 0.031 | 0.016 | 0.017 | 0.002 | 0.021 |
| rs78378222 | 17 | 7571752 | Yes | No | T | G | 0.986 | 0.455 | 0.138 | -0.24 | 0.063 | -0.012 | 0.075 |
| rs8069739 | 17 | 8078765 | Yes | No | T | C | 0.322 | -0.159 | 0.032 | 0.026 | 0.018 | -0.004 | 0.022 |
| rs4925159 | 17 | 18185510 | Yes | Yes | A | G | 0.425 | 0.217 | 0.03 | -0.014 | 0.017 | -0.019 | 0.021 |
| rs7502046 | 17 | 19196440 | Yes | No | T | C | 0.81 | 0.177 | 0.039 | 0.002 | 0.022 | -0.002 | 0.026 |
| rs138285687 | 17 | 27195674 | No | No | - | - | - | - | - | - | - | - | - |
| rs11080134 | 17 | 29161503 | Yes | Yes | A | G | 0.643 | -0.168 | 0.032 | -0.065 | 0.018 | -0.032 | 0.021 |
| rs1551355 | 17 | 30032420 | Yes | Yes | T | C | 0.233 | 0.21 | 0.036 | 0.015 | 0.02 | -0.047 | 0.024 |
| rs9899540 | 17 | 30777924 | Yes | No | A | T | 0.4 | 0.201 | 0.032 | -0.019 | 0.017 | 0.009 | 0.021 |
| rs3135967 | 17 | 33313729 | Yes | No | A | G | 0.524 | 0.064 | 0.03 | 0.021 | 0.017 | -0.01 | 0.02 |
| rs79089478 | 17 | 40317241 | Yes | No | T | C | 0.973 | 0.333 | 0.095 | 0.036 | 0.051 | 0.027 | 0.062 |
| rs56228409 | 17 | 40919596 | Yes | No | A | C | 0.846 | -0.185 | 0.044 | -0.015 | 0.022 | 0.004 | 0.026 |
| rs9904409 | 17 | 42680402 | Yes | No | A | G | 0.097 | 0.368 | 0.051 | -0.046 | 0.029 | -0.024 | 0.035 |
| rs12946454 | 17 | 43208121 | Yes | Yes | A | T | 0.735 | -0.412 | 0.034 | 0.009 | 0.02 | -0.014 | 0.023 |
| rs115231027 | 17 | 44199290 | No | No | - | - | - | - | - | - | - | - | - |
| rs17608766 | 17 | 45013271 | Yes | No | T | C | 0.856 | -0.69 | 0.043 | -0.028 | 0.025 | -0.002 | 0.029 |
| rs62076103 | 17 | 45888374 | Yes | No | A | G | 0.931 | -0.58 | 0.06 | -0.034 | 0.032 | 0.054 | 0.039 |
| rs7406910 | 17 | 46688256 | Yes | Yes | T | C | 0.088 | -0.435 | 0.053 | 0.003 | 0.029 | 0.009 | 0.035 |
| rs12940887 | 17 | 47402807 | Yes | No | T | C | 0.367 | 0.26 | 0.031 | -0.023 | 0.017 | -0.059 | 0.021 |
| rs12325702 | 17 | 55446364 | No | No | - | - | - | - | - | - | - | - | - |
| rs34430710 | 17 | 56876627 | Yes | No | A | T | 0.676 | -0.212 | 0.032 | 0.028 | 0.018 | 0.006 | 0.022 |
| rs2645466 | 17 | 57853214 | Yes | No | A | C | 0.705 | -0.16 | 0.033 | 0.014 | 0.019 | -0.062 | 0.022 |
| rs1036902 | 17 | 58950791 | Yes | No | T | C | 0.847 | -0.254 | 0.042 | -0.018 | 0.023 | 0.015 | 0.028 |
| rs2240736 | 17 | 59485393 | Yes | Yes | T | C | 0.734 | 0.409 | 0.034 | -0.034 | 0.019 | -0.002 | 0.023 |
| rs740698 | 17 | 60767151 | Yes | No | T | C | 0.565 | -0.21 | 0.031 | -0.004 | 0.017 | -0.002 | 0.02 |
| rs4308 | 17 | 61559625 | Yes | No | A | G | 0.378 | 0.273 | 0.031 | 0.029 | 0.017 | -0.03 | 0.021 |
| rs6504213 | 17 | 62381714 | Yes | No | T | C | 0.418 | -0.298 | 0.031 | 0.034 | 0.017 | 0.016 | 0.02 |
| rs112260610 | 17 | 64252393 | Yes | No | T | C | 0.14 | 0.259 | 0.044 | 0.014 | 0.024 | 0.026 | 0.029 |
| rs2467099 | 17 | 73949045 | Yes | No | T | C | 0.223 | -0.246 | 0.036 | 0.006 | 0.021 | -0.028 | 0.025 |
| rs35504735 | 17 | 74686809 | Yes | No | A | G | 0.558 | 0.024 | 0.03 | -0.034 | 0.017 | 0.002 | 0.02 |
| rs57927100 | 17 | 75317300 | Yes | No | C | G | 0.737 | 0.315 | 0.035 | 0.003 | 0.02 | -0.007 | 0.023 |
| rs9302885 | 17 | 76799898 | Yes | Yes | A | G | 0.445 | 0.224 | 0.03 | 0.015 | 0.017 | -0.013 | 0.02 |
| rs112280096 | 17 | 79367409 | No | No | - | - | - | - | - | - | - | - | - |
| rs34413141 | 18 | 777282 | Yes | No | A | T | 0.182 | -0.353 | 0.039 | 0.016 | 0.022 | 0.007 | 0.026 |
| rs11665020 | 18 | 10879503 | Yes | No | C | G | 0.321 | -0.13 | 0.032 | 0.032 | 0.018 | -0.025 | 0.022 |
| rs963920 | 18 | 12711052 | Yes | No | T | G | 0.675 | -0.102 | 0.032 | -0.029 | 0.018 | 0.023 | 0.021 |
| rs4800420 | 18 | 20158965 | Yes | No | A | G | 0.292 | 0.094 | 0.033 | 0.006 | 0.018 | -0.01 | 0.022 |
| rs1154214 | 18 | 24546824 | Yes | No | T | G | 0.396 | -0.203 | 0.031 | -0.002 | 0.017 | -0.01 | 0.021 |
| rs10164193 | 18 | 31161426 | Yes | No | T | G | 0.922 | -0.278 | 0.057 | 0.014 | 0.036 | 0.056 | 0.043 |
| rs61735998 | 18 | 34289285 | Yes | No | T | G | 0.024 | 0.557 | 0.103 | 0.067 | 0.057 | 0.023 | 0.07 |
| rs12958173 | 18 | 42141977 | Yes | Yes | A | C | 0.297 | 0.295 | 0.033 | -0.001 | 0.019 | -0.034 | 0.022 |
| rs7236548 | 18 | 43097750 | Yes | No | A | C | 0.185 | 0.343 | 0.039 | -0.021 | 0.023 | 0.012 | 0.027 |
| rs745821 | 18 | 48142854 | Yes | No | T | G | 0.755 | 0.221 | 0.035 | 0.016 | 0.019 | 0 | 0.023 |
| rs36010659 | 18 | 48283949 | Yes | No | T | C | 0.859 | 0.291 | 0.043 | -0.005 | 0.025 | -0.009 | 0.031 |
| rs11876341 | 18 | 48799991 | Yes | No | A | G | 0.692 | -0.213 | 0.033 | -0.018 | 0.019 | -0.009 | 0.022 |
| rs34163044 | 18 | 51851616 | Yes | No | A | C | 0.42 | 0.175 | 0.031 | 0.013 | 0.017 | 0.014 | 0.02 |
| rs72930904 | 18 | 52607301 | Yes | No | T | C | 0.16 | -0.232 | 0.041 | 0.003 | 0.023 | 0.006 | 0.028 |
| rs12605156 | 18 | 53498114 | Yes | No | A | T | 0.808 | 0.204 | 0.038 | 0.01 | 0.021 | 0.031 | 0.025 |
| rs10048404 | 18 | 54578482 | No | No | - | - | - | - | - | - | - | - | - |
| rs7235890 | 18 | 55732115 | Yes | No | T | G | 0.896 | -0.212 | 0.05 | 0.038 | 0.028 | 0.029 | 0.033 |
| rs6567160 | 18 | 57829135 | Yes | No | T | C | 0.766 | 0.224 | 0.036 | -0.01 | 0.019 | -0.047 | 0.023 |
| rs12172847 | 18 | 60223017 | Yes | No | A | G | 0.323 | -0.144 | 0.032 | 0.034 | 0.018 | -0.002 | 0.022 |
| rs12454712 | 18 | 60845884 | No | No | - | - | - | - | - | - | - | - | - |
| rs10460108 | 18 | 73034151 | Yes | No | A | G | 0.48 | 0.214 | 0.03 | -0.008 | 0.017 | 0.017 | 0.02 |
| rs1047922 | 18 | 74070562 | No | No | - | - | - | - | - | - | - | - | - |
| rs7250835 | 19 | 670234 | Yes | No | T | C | 0.165 | 0.207 | 0.043 | 0.011 | 0.023 | -0.021 | 0.028 |
| rs3760994 | 19 | 1435771 | No | No | - | - | - | - | - | - | - | - | - |
| rs740406 | 19 | 2232221 | Yes | No | A | G | 0.94 | -0.591 | 0.066 | 0.025 | 0.035 | -0.059 | 0.041 |
| rs2656523 | 19 | 4085896 | Yes | No | C | G | 0.773 | 0.14 | 0.037 | -0.038 | 0.02 | 0.026 | 0.024 |
| rs2613765 | 19 | 5066330 | Yes | No | A | G | 0.475 | -0.235 | 0.03 | -0.004 | 0.017 | 0.028 | 0.02 |
| rs7248104 | 19 | 7224431 | Yes | No | A | G | 0.412 | -0.233 | 0.031 | 0.003 | 0.017 | -0.007 | 0.02 |
| rs4247374 | 19 | 7252756 | No | No | - | - | - | - | - | - | - | - | - |
| rs2009733 | 19 | 8398714 | Yes | No | A | G | 0.5 | 0.115 | 0.031 | 0.024 | 0.017 | -0.042 | 0.02 |
| rs10409243 | 19 | 10332988 | Yes | No | T | C | 0.59 | -0.308 | 0.031 | -0.02 | 0.017 | 0.041 | 0.02 |
| rs1529744 | 19 | 10841472 | Yes | No | T | C | 0.318 | -0.154 | 0.032 | -0.023 | 0.019 | 0.012 | 0.022 |
| rs167479 | 19 | 11526765 | No | No | - | - | - | - | - | - | - | - | - |
| rs17638167 | 19 | 11584818 | Yes | No | T | C | 0.044 | -0.452 | 0.074 | 0.021 | 0.044 | -0.084 | 0.053 |
| rs10418305 | 19 | 15278808 | Yes | No | C | G | 0.099 | -0.162 | 0.051 | 0.04 | 0.032 | -0.008 | 0.038 |
| rs3745318 | 19 | 16436262 | No | No | - | - | - | - | - | - | - | - | - |
| rs1077795 | 19 | 17222584 | Yes | No | A | G | 0.739 | 0.251 | 0.034 | -0.01 | 0.019 | -0.046 | 0.023 |
| rs8111708 | 19 | 18558876 | Yes | No | A | G | 0.653 | 0.064 | 0.032 | -0.006 | 0.018 | 0.007 | 0.021 |
| rs2304130 | 19 | 19789528 | Yes | No | A | G | 0.916 | -0.339 | 0.055 | -0.041 | 0.028 | -0.023 | 0.034 |
| rs6511291 | 19 | 21950402 | Yes | No | T | C | 0.436 | -0.169 | 0.031 | -0.007 | 0.017 | -0.005 | 0.02 |
| rs62104477 | 19 | 30294991 | Yes | No | T | G | 0.33 | 0.108 | 0.032 | -0.009 | 0.018 | -0.014 | 0.022 |
| rs8105753 | 19 | 31927547 | No | No | - | - | - | - | - | - | - | - | - |
| rs1821295 | 19 | 32590773 | Yes | No | T | C | 0.699 | -0.225 | 0.033 | -0.008 | 0.018 | 0.03 | 0.021 |
| rs7256564 | 19 | 33889593 | Yes | No | A | G | 0.312 | 0.196 | 0.032 | -0.01 | 0.018 | 0.022 | 0.022 |
| rs12983238 | 19 | 39438532 | Yes | No | A | G | 0.306 | -0.129 | 0.035 | -0.036 | 0.019 | -0.004 | 0.022 |
| rs1800470 | 19 | 41858921 | Yes | No | A | G | 0.624 | -0.101 | 0.031 | 0.002 | 0.018 | 0.01 | 0.021 |
| rs7412 | 19 | 45412079 | Yes | No | T | C | 0.082 | -0.434 | 0.057 | 0.002 | 0.033 | 0.08 | 0.039 |
| rs34783010 | 19 | 46180414 | Yes | No | T | G | 0.2 | 0.221 | 0.038 | 0.03 | 0.02 | 0.011 | 0.024 |
| rs73046792 | 19 | 49605705 | Yes | No | A | G | 0.159 | -0.355 | 0.043 | 0.031 | 0.023 | -0.079 | 0.027 |
| rs138877676 | 19 | 50935809 | Yes | No | T | G | 0.019 | -0.66 | 0.124 | -0.126 | 0.063 | 0.044 | 0.075 |
| rs2143635 | 20 | 2793063 | Yes | No | T | C | 0.9 | -0.168 | 0.051 | 0 | 0.029 | -0.009 | 0.035 |
| rs1764975 | 20 | 4101290 | Yes | No | A | T | 0.795 | 0.282 | 0.038 | -0.004 | 0.02 | 0.018 | 0.024 |
| rs11087740 | 20 | 6657554 | Yes | No | T | C | 0.512 | -0.163 | 0.03 | -0.005 | 0.017 | 0.017 | 0.02 |
| rs6108168 | 20 | 8626271 | Yes | No | A | C | 0.254 | -0.296 | 0.034 | -0.04 | 0.019 | -0.025 | 0.023 |
| rs680515 | 20 | 10458688 | Yes | No | A | G | 0.389 | -0.088 | 0.031 | -0.02 | 0.017 | -0.051 | 0.021 |
| rs1327235 | 20 | 10969030 | Yes | No | A | G | 0.529 | -0.426 | 0.03 | -0.015 | 0.017 | 0.03 | 0.02 |
| rs1232482 | 20 | 11886643 | Yes | No | T | C | 0.402 | -0.138 | 0.031 | 0.01 | 0.017 | 0.017 | 0.021 |
| rs2618647 | 20 | 17882452 | Yes | No | A | G | 0.505 | -0.188 | 0.03 | 0.006 | 0.017 | -0.003 | 0.02 |
| rs6081613 | 20 | 19465907 | Yes | No | A | G | 0.274 | 0.182 | 0.034 | -0.022 | 0.019 | 0.005 | 0.023 |
| rs6060114 | 20 | 30169673 | Yes | No | T | C | 0.842 | 0.273 | 0.041 | -0.001 | 0.022 | -0.028 | 0.027 |
| rs6141767 | 20 | 31225069 | Yes | No | C | G | 0.157 | 0.311 | 0.042 | 0.022 | 0.023 | -0.014 | 0.027 |
| rs13042148 | 20 | 32298286 | Yes | No | T | C | 0.153 | -0.134 | 0.042 | -0.004 | 0.025 | -0.014 | 0.029 |
| rs6141479 | 20 | 33121942 | Yes | No | C | G | 0.197 | 0.1 | 0.039 | -0.03 | 0.021 | -0.023 | 0.026 |
| rs4811601 | 20 | 36849007 | Yes | No | T | C | 0.459 | 0.064 | 0.031 | -0.007 | 0.017 | -0.003 | 0.02 |
| rs4810332 | 20 | 40268334 | Yes | No | A | T | 0.379 | -0.261 | 0.032 | 0.008 | 0.017 | 0.008 | 0.021 |
| rs6031435 | 20 | 42797358 | Yes | No | A | G | 0.54 | -0.259 | 0.03 | -0.004 | 0.017 | 0.012 | 0.02 |
| rs6095241 | 20 | 47308798 | Yes | No | A | G | 0.44 | -0.172 | 0.03 | -0.013 | 0.017 | 0.007 | 0.02 |
| rs237485 | 20 | 48004238 | Yes | No | A | G | 0.698 | 0.11 | 0.033 | 0.018 | 0.018 | 0.028 | 0.022 |
| rs6021247 | 20 | 50108980 | Yes | Yes | A | G | 0.529 | 0.233 | 0.03 | 0.013 | 0.017 | 0.005 | 0.02 |
| rs6015450 | 20 | 57751117 | Yes | No | A | G | 0.876 | -0.7 | 0.046 | -0.004 | 0.024 | 0.037 | 0.029 |
| rs35213536 | 20 | 62694319 | Yes | No | T | G | 0.247 | 0.294 | 0.036 | -0.021 | 0.019 | 0.007 | 0.023 |
| rs1882961 | 21 | 16556367 | Yes | Yes | T | C | 0.309 | 0.244 | 0.033 | 0.015 | 0.018 | -0.045 | 0.021 |
| rs11909120 | 21 | 30131872 | Yes | No | A | T | 0.857 | -0.092 | 0.043 | 0.02 | 0.025 | 0.051 | 0.03 |
| rs11701033 | 21 | 33788341 | Yes | No | C | G | 0.818 | -0.219 | 0.039 | 0.007 | 0.022 | -0.018 | 0.026 |
| rs9976596 | 21 | 35596842 | Yes | No | T | C | 0.843 | 0.097 | 0.042 | -0.018 | 0.023 | -0.023 | 0.027 |
| rs62229372 | 21 | 37692507 | Yes | No | T | C | 0.128 | 0.241 | 0.047 | 0.007 | 0.026 | 0.057 | 0.031 |
| rs2277788 | 21 | 40817702 | Yes | No | C | G | 0.105 | 0.23 | 0.049 | -0.025 | 0.027 | 0.027 | 0.033 |
| rs12627651 | 21 | 44760603 | Yes | No | A | G | 0.287 | 0.35 | 0.034 | 0.001 | 0.018 | -0.003 | 0.022 |
| rs9306160 | 21 | 45107562 | Yes | No | T | C | 0.411 | -0.212 | 0.031 | -0.008 | 0.017 | -0.003 | 0.021 |
| rs35796750 | 21 | 47422412 | Yes | No | T | C | 0.55 | -0.134 | 0.03 | -0.012 | 0.017 | -0.017 | 0.02 |
| rs11701512 | 21 | 47962811 | Yes | No | A | G | 0.181 | 0.196 | 0.039 | 0.012 | 0.021 | 0.015 | 0.026 |
| rs12628032 | 22 | 19967980 | Yes | No | T | C | 0.309 | 0.244 | 0.033 | -0.018 | 0.018 | -0.018 | 0.021 |
| rs134041 | 22 | 28056338 | Yes | No | T | C | 0.436 | 0.019 | 0.03 | 0.003 | 0.017 | -0.021 | 0.02 |
| rs9608690 | 22 | 28921347 | Yes | No | A | G | 0.068 | -0.371 | 0.06 | 0.008 | 0.033 | 0.017 | 0.039 |
| rs4823006 | 22 | 29451671 | Yes | No | A | G | 0.555 | 0.217 | 0.03 | -0.008 | 0.017 | 0.021 | 0.02 |
| rs737721 | 22 | 30172254 | Yes | No | C | G | 0.948 | -0.407 | 0.068 | 0.007 | 0.034 | -0.041 | 0.041 |
| rs5753103 | 22 | 30768777 | Yes | No | A | G | 0.452 | 0.13 | 0.03 | -0.007 | 0.017 | 0.041 | 0.02 |
| rs9609429 | 22 | 32517431 | Yes | No | T | C | 0.724 | 0.13 | 0.034 | 0.043 | 0.019 | 0.017 | 0.023 |
| rs5750482 | 22 | 38117943 | Yes | No | T | C | 0.381 | 0.158 | 0.031 | 0.011 | 0.017 | -0.028 | 0.021 |
| rs470113 | 22 | 40729614 | Yes | No | A | G | 0.818 | -0.185 | 0.039 | -0.001 | 0.021 | 0.003 | 0.025 |
| rs73161324 | 22 | 42038786 | Yes | No | T | C | 0.056 | 0.203 | 0.07 | -0.057 | 0.034 | -0.005 | 0.04 |
| rs77692990 | 22 | 50219952 | Yes | No | T | C | 0.08 | -0.318 | 0.057 | 0.016 | 0.032 | 0.004 | 0.038 |
| rs28578714 | 22 | 50727921 | Yes | No | T | C | 0.606 | 0.207 | 0.033 | 0.013 | 0.017 | 0.027 | 0.021 |
