# Supplementary material for: Impaired glucose tolerance and cardiovascular risk factors in relation to infertility: a Mendelian randomization analysis in the Norwegian Mother, Father, and Child Cohort Study
Source: Hum Reprod. 2023 Nov 8;39(2):436–41. doi: 10.1093/humrep/dead234 (PMC10833082; doi:10.1093/humrep/dead234)
Supplement: dead234_Supplementary_Table_S9 [file dead234_supplementary_table_s9.docx]

**Supplementary Table S9.** Number of genetic variants used in all analyses

|  | **Genetic variants in original GWAS (*n*, %)** | **Used in main**  **MR analyses**  **(*n*, %)** | **Used in MR**  **+ Steiger filt.**  **(*n*, %)** |
| --- | --- | --- | --- |
| Fasting glucose | 138 (100%) | 121 (87.7%) | 67 (48.6%) |
| Glycated hemoglobin | 146 (100%) | 124 (84.9%) | 47 (32.2%) |
| Fasting  insulin | 62 (100%) | 56 (90.3%) | 30 (48.4%) |
| LDL  cholesterol | 389 (100%) | 311 (79.9%) | 72 (18.5%) |
| HDL cholesterol | 371 (100%) | 311 (83.8%) | 34 (9.2%) |
| Triglycerides | 384 (100%) | 322 (83.9%) | 31 (8.1%) |
| Systolic blood pressure | 883 (100%) | 817 (92.5%) | 83 (9.4%) |
| Diastolic blood pressure | 885 (100%) | 818 (92.4%) | 114 (12.9%) |
