# Supplementary material for: Impaired glucose tolerance and cardiovascular risk factors in relation to infertility: a Mendelian randomization analysis in the Norwegian Mother, Father, and Child Cohort Study
Source: Hum Reprod. 2023 Nov 8;39(2):436–41. doi: 10.1093/humrep/dead234 (PMC10833082; doi:10.1093/humrep/dead234)
Supplement: dead234_Supplementary_Table_S10 [file dead234_supplementary_table_s10.docx]

**Supplementary Table S10.** Comparison of participants included (with genotype data) and not-included (without genotype data)

|  | **Women** | | **Men** | |
| --- | --- | --- | --- | --- |
|  | **Included** | **Non-included** | **Included** | **Non-included** |
| Age at delivery,  years (mean ± SD) | 30.5 ± 4.24 | 29.6 ± 5.15 | 32.8 ± 4.95 | 32.7 ± 5.79 |
| Education years  (mean ± SD) | 17.3 ± 3.20 | 16.5 ± 3.57 | 16.6 ± 3.50 | 15.7 ± 3.66 |
| BMI, kg/m^2^  (median, 1^st^-3^rd^ quartile) | 23.1  (21.2-26.0) | 23.0  (21.0-25.9) | 25.4  (23.7-27.7) | 25.3  (23.5-27.7) |
| Ever smokers  (*n*, %): | 31,472  (48.8%) | 18,566  (54.6%) | 22,265  (48.5%) | 24,926  (46.4%) |
| Trying for a first pregnancy (*n*, %): | 28,524  (43.7%) | 16,062  (46.8%) | 21,351  (46.6%) | 23,235  (43.2%) |
| Subfertility reported  (*n*, %) | 8,427  (12.9%) | 2,114  (6.12%) | 5,907  (12.9%) | 4,634  (8.57%) |
