# Supplementary material for: Impaired glucose tolerance and cardiovascular risk factors in relation to infertility: a Mendelian randomization analysis in the Norwegian Mother, Father, and Child Cohort Study
Source: Hum Reprod. 2023 Nov 8;39(2):436–41. doi: 10.1093/humrep/dead234 (PMC10833082; doi:10.1093/humrep/dead234)
Supplement: dead234_Supplementary_Table_S11 [file dead234_supplementary_table_s11.docx]

**Supplementary Table S11.** Sensitivity analyses on the verification of Mendelian randomization assumptions.

|  | **Main MR analyses**  **(inverse variance**  **weighted method;**  **(OR, 95% CI)** | **Estimated**  **F-statistic** | **MR-Egger intercept test**  **(*p*-value)** | **Between-SNP heterogeneity** | | **Sensitivity MR methods** | | | **Steiger filtering** |
| --- | --- | --- | --- | --- | --- | --- | --- | --- | --- |
|  |  |  |  | **Cochran’s *Q*** | **Rücker’s *Q’*** | **MR-Egger**  **(OR, 95% CI)** | **Weighted median**  **(OR, 95% CI)** | **Weighted mode**  **(OR, 95% CI)** | **Inverse variance**  **weighted method**  **(OR, 95% CI)** |
|  |  |  |  |  |  |  |  |  |  |
| Fasting glucose  Women | 1.14  (0.99 to 1.33) | 174 | 0.517 | 163  (*p*=0.006) | 162  (*p*=0.005) | 1.07  (0.83 to 1.38) | 1.24  (1.01 to 1.51) | 1.23  (0.98 to 1.53) | 1.09  (0.90 to 1.32) |
| Fasting glucose  Men | 1.06  (0.91 to 1.24) | 174 | 0.599 | 106  (*p*=0.821) | 105  (*p*=0.809) | 1.01  (0.78 to 1.30) | 1.15  (0.91 to 1.45) | 1.12  (0.90 to 1.39) | 1.06  (0.88 to 1.28) |
| Glycated hemoglobin  Women | 1.14  (0.92 to 1.41) | 151 | 0.426 | 137  (*p*=0.187) | 136  (*p*=0.182) | 1.28  (0.90 to 1.82) | 1.26  (0.91 to 1.76) | 1.13  (0.79 to 1.61) | 1.30  (0.90 to 1.90) |
| Glycated hemoglobin  Men | 0.98  (0.77 to 1.24) | 151 | 0.943 | 122  (*p*=0.509) | 122  (*p*=0.483) | 0.97  (0.65 to 1.44) | 0.94  (0.64 to 1.37) | 1.00  (0.69 to 1.45) | 0.90  (0.52 to 1.54) |
| Fasting insulin  Women | 1.60  (1.17 to 2.18) | 52.5 | 0.194 | 55.8  (*p*=0.445) | 54.1  (*p*=0.472) | 2.94  (1.12 to 7.69) | 1.46  (0.93 to 2.28) | 1.45  (0.71 to 2.95) | 1.57  (0.98 to 2.52) |
| Fasting insulin  Men | 1.20  (0.80 to 1.80) | 52.5 | 0.190 | 66.7  (*p*=0.135) | 64.5  (*p*=0.154) | 2.68  (0.77 to 9.41) | 1.53  (0.87 to 2.68) | 3.18  (1.16 to 8.71) | 1.06  (0.59 to 1.91) |
| LDL cholesterol  Women | 0.98  (0.91 to 1.05) | 4.50 × 10^8^ | 0.298 | 312  (*p*=0.462) | 311  (*p*=0.464) | 1.01  (0.92 to 1.10) | 1.00  (0.89 to 1.12) | 0.99  (0.91 to 1.09) | 1.00  (0.86 to 1.17) |
| LDL cholesterol  Men | 0.96  (0.88 to 1.05) | 4.50 × 10^8^ | 0.162 | 311  (*p*=0.473) | 309  (*p*=0.489) | 0.91  (0.82 to 1.02) | 0.86  (0.75 to 0.99) | 0.91  (0.82 to 1.01) | 1.02  (0.85 to 1.22) |
| HDL cholesterol  Women | 1.02  (0.95 to 1.10) | 3.79 × 10^8^ | 0.298 | 295  (*p*=0.712) | 294  (*p*=0.714) | 1.06  (0.96 to 1.18) | 1.09  (0.94 to 1.26) | 1.06  (0.94 to 1.18) | 1.08  (0.88 to 1.34) |
| HDL cholesterol  Men | 0.99  (0.90 to 1.09) | 3.79 × 10^8^ | 0.996 | 358  (*p*=0.031) | 358  (*p*=0.028) | 0.99  (0.86 to 1.14) | 0.96  (0.81 to 1.15) | 1.00  (0.87 to 1.16) | 1.08  (0.86 to 1.36) |
| Triglycerides  Women | 1.00  (0.92 to 1.09) | 3.35 × 10^8^ | <0.001 | 350  (*p*=0.129) | 335  (*p*=0.274) | 0.85  (0.76 to 0.96) | 0.93  (0.82 to 1.07) | 0.92  (0.82 to 1.03) | 0.97  (0.72 to 1.32) |
| Triglycerides  Men | 1.04  (0.93 to 1.15) | 3.35 × 10^8^ | 0.872 | 383  (*p*=0.010) | 383  (*p*=0.009) | 1.03  (0.88 to 1.20) | 0.98  (0.82 to 1.18) | 1.02  (0.88 to 1.17) | 0.90  (0.63 to 1.31) |
| Systolic blood pres.  Women | 1.00  (1.00 to 1.01) | 46.1 | 0.824 | 817  (*p*=0.486) | 817  (*p*=0.476) | 1.00  (0.99 to 1.02) | 1.00  (0.99 to 1.01) | 1.01  (0.98 to 1.05) | 0.99  (0.98 to 1.01) |
| Systolic blood pres.  Men | 1.00  (0.99 to 1.00) | 46.1 | 0.846 | 850  (*p*=0.202) | 849  (*p*=0.195) | 1.00  (0.98 to 1.01) | 0.99  (0.98 to 1.01) | 0.99  (0.81 to 1.21) | 1.00  (0.98 to 1.01) |
| Diastolic blood pres.  Women | 1.01  (1.00 to 1.01) | 45.2 | 0.584 | 819  (*p*=0.470) | 819  (*p*=0.463) | 1.01  (0.99 to 1.03) | 1.01  (0.99 to 1.02) | 1.01  (0.99 to 1.02) | 1.00  (0.98 to 1.02) |
| Diastolic blood pres.  Men | 0.99  (0.98 to 1.00) | 45.2 | 0.192 | 849  (*p*=0.216) | 847  (*p*=0.221) | 0.98  (0.96 to 1.00) | 0.98  (0.96 to 1.00) | 0.98  (0.96 to 1.00) | 0.98  (0.96 to 1.00) |
